# Triarylmethanes and their Medium‐Ring Analogues by Unactivated Truce–Smiles Rearrangement of Benzanilides

**DOI:** 10.1002/anie.202102192

**Published:** 2021-04-08

**Authors:** Roman Abrams, Mehul H. Jesani, Alex Browning, Jonathan Clayden

**Affiliations:** ^1^ School of Chemistry University of Bristol, Cantock's Close Bristol BS8 1TS UK

**Keywords:** Hammett plot, medium ring, S_N_Ar, triarylmethane, Truce–Smiles rearrangement

## Abstract

Intramolecular nucleophilic aromatic substitution (Truce–Smiles rearrangement) of the anions of 2‐benzyl benzanilides leads to triarylmethanes in an operationally simple manner. The reaction succeeds even without electronic activation of the ring that plays the role of electrophile in the S_N_Ar reaction, being accelerated instead by the preferred conformation imposed by the tertiary amide tether. The amide substituent of the product may be removed or transformed into alternative functional groups. A ring‐expanding variant (n to n+4) of the reaction provided a route to doubly benzo‐fused medium ring lactams of 10 or 11 members. Hammett analysis returned a ρ value consistent with the operation of a partially concerted reaction mechanism.

## Graphical abstract

A conformationally accelerated intramolecular arylation provides an operationally simple way to prepare triarylmethanes and their medium‐ring analogues. In situ IR spectroscopy and a Hammett plot revealed mechanistic details of the electronically unactivated S_N_Ar reaction.

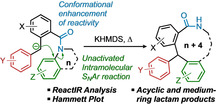

Triarylmethanes are privileged molecular structures finding application in numerous aspects of chemical science.[^1^, ^2^, ^3^, ^4^, ^5^, ^6^, ^7^, ^8^] The triarylmethane scaffold occurs in natural products,^[1]^ medicinal agents,^[2]^ building blocks in materials chemistry,^[3]^ and ligand scaffolds.^[4]^ Additionally, triarylmethanes are commonly used as dyes^[5]^ and fluorescent probes,^[6]^ culminating in their recent use as red light‐absorbing photoredox catalysts^[7]^ and red light‐fluorescing OLEDs.^[8]^

Owing to the diverse utility of triarylmethanes, various synthetic methods have been developed for their preparation.^[9]^ Common approaches include Lewis and Brønsted acid‐mediated Friedel–Crafts alkylations,^[10]^ and transition metal‐catalysed C(sp^3^)−C(sp^2^) Suzuki or Kumada cross‐couplings (Scheme 1 a).^[11]^ They may also be made by the C−H arylation of diarylmethanes that results from deprotonative transition metal‐catalysed cross‐coupling processes (Scheme 1 b),^[12]^ or by Lewis or Brønsted acid‐promoted arylations of quinone methides (Scheme 1 c).^[13]^

**Scheme 1:**
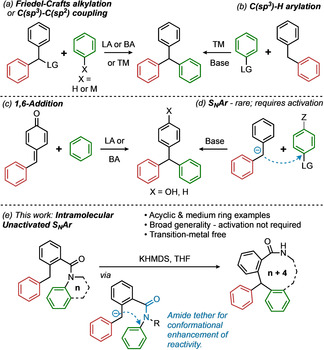
a–d) Previous approaches to triarylmethanes. e) This work: synthesis of triarylmethanes by conformationally accelerated intramolecular S_N_Ar (Truce–Smiles) rearrangement. LA: Lewis acid, BA: Brønsted acid, TM: transition metal.

Nucleophilic aromatic substitution (S_N_Ar) reactions have been used as a valuable way of making Ar−heteroatom and Ar−C bonds, but as a reaction class they have recently undergone an academic resurgence, as a result of several related discoveries in the field of concerted S_N_Ar mechanisms.[^14^, ^15^, ^16^, ^17^, ^18^] Jacobsen and co‐workers^[14]^ showed that concerted S_N_Ar reactions are far from a mechanistic rarity, paving the way for their broader application in synthesis as they provide a transition metal‐free route to arylation reactions.^[15]^ S_N_Ar reactions of diarylmethane carbanions conceptually offer a simple method for triarylmethane construction, but reports are surprisingly sparse and restricted to highly activated nitro‐containing aryl electrophiles (Scheme 1 d).^[19]^

Here we describe a much more general and operationally simple approach to triarylmethanes by the use of a conformationally enhanced intramolecular S_N_Ar reaction, or Truce–Smiles rearrangement (Scheme 1 e). Classical Truce–Smiles rearrangements proceed by 1,4‐ or 1,5‐N or O to C intramolecular S_N_Ar of electron‐deficient aryl rings, but in our conformationally accelerated variant, much greater substrate generality is possible because no electronic activation is required.[^20^, ^21^]

*N*‐Methylated benzanilides such as **1** prefer to adopt *E* conformations in which the *N*‐aryl ring lies *trans* to the carbonyl group,^[22]^ and with the amide carbonyl group twisted more or less perpendicular to the benzamide ring.^[23]^ This conformational bias can be used to promote intramolecular S_N_Ar for unactivated and even moderately electron‐rich aryl rings, by pre‐organisation of the nucleophilic and electrophilic components for reaction.^[18]^ With the aim of exploiting such a reaction for the synthesis of triarylmethanes, 2‐benzylbenzamide **1** was treated with base with the aim of generating a nucleophilic benzylic anion (Table 1).

**Table 1:** Optimisation studies. 
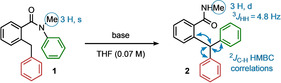

Entry^[a]^	Base (equiv)	*T* [°C]^[b]^	*t* [h]	Yield [%]^[c]^
1	NaHMDS (2.0)	20	16	<5
2	NaHMDS (2.0)	60	16	49
3	NaHMDS (2.0)	100	1	73
4	LiHMDS (2.0)	100	1	62
**5**	**KHMDS (2.0)**	**100**	**1**	**81 (67)**
6	KHMDS (1.1)	100	1	70
7	KHMDS (2.0)	120	1	69

[a] Reactions performed on a 0.1 mmol scale. KHMDS (1.0 m in THF)=potassium bis(trimethylsilyl)amide, NaHMDS (1.0 m in THF)=sodium bis(trimethylsilyl)amide, LiHMDS (1.0 m in THF)=lithium bis(trimethylsilyl)amide. [b] Reactions requiring 60 °C and below were heated conventionally. Reactions requiring heating above 60 °C were performed under microwave irradiation. [c] Yield determined by ^1^H NMR using 1,3,5‐trimethoxybenzene as an internal standard. Isolated yield in parentheses (0.2 mmol scale reaction).

Initial attempts with NaHMDS at ambient temperature only afforded trace amounts of product (Table 1, entry 1). However, at elevated temperatures 2‐benzylbenzamide **1** underwent Truce–Smiles rearrangement to give triarylmethane **2** (Table 1, entries 2, 3). A base screen identified KHMDS as the optimal choice (Table 1, entries 3–5), with THF and Et_2_O as the best performing solvents (See SI). Slightly lower yields were obtained when the KHMDS stoichiometry was reduced from 2.0 or if the reaction temperature was elevated to 120 °C (Table 1, entries 6, 7). Key indicators of the spectroscopic assignment of the structure of the product are shown in the scheme in Table 1.

With optimised conditions identified for the rearrangement of **1** we investigated the generality of the reaction. Firstly, we explored the scope of aromatic rings that undergo N to C migration (Scheme 2). As with other conformationally accelerated Truce–Smiles rearrangements,[^16^, ^17^] migration even of electron‐rich (**4 a** and **4 b**) and *ortho*‐functionalised (**4 c** and **4 d**) aromatic rings proceeded cleanly at elevated temperatures. Fluorinated aromatic rings (**4 e** and **4 f**) were likewise successfully incorporated into their triarylmethanes, with **4 f** forming even at ambient temperature. The migrations of both chlorinated and brominated aromatics required lower reaction temperatures to avoid protodehalogenation, but were all ultimately successful (**4 g**–**i**). Heterocyclic triarylmethanes could also be formed, with 4‐, 3‐, and 2‐substituted pyridines all migrating in excellent yield (**4 j**–**l**).

**Scheme 2:**
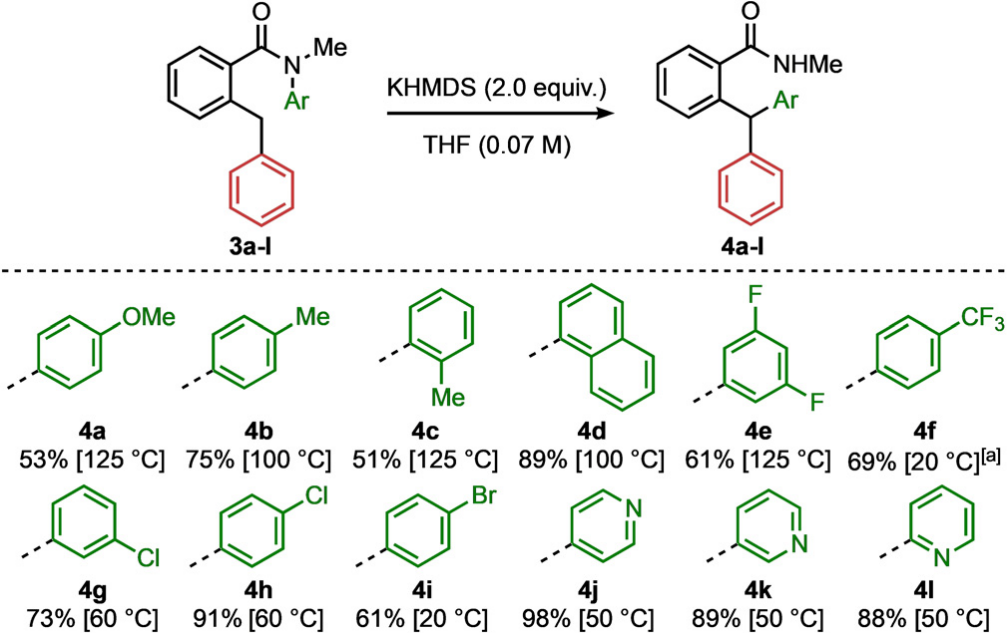
Scope of migrating aryl group (green aryl group) for the Truce–Smiles rearrangement. Reactions were carried out on a 0.20 mmol scale. Yields of product after isolation by chromatography, and temperatures of reaction are quoted. [a] With LiHMDS used in place of KHMDS.

The ring coloured red in the schemes plays the role of an anion‐stabilising group, and extensive variation in this ring was explored, as indicated in Scheme 3. The reaction was unaffected by variation in this position, tolerating a variety of substituents, including methoxy, methyl, phenyl, and fluoro groups (**6 a**–**6 d**). Substitution in the *ortho* position was not detrimental, with products containing more hindered *ortho*‐tolyl and 1‐naphthyl groups forming readily (**6 e** and **6 f**). Triarylmethanes containing 3,4‐disubstituted phenyl rings bearing methyl, fluoro, and a dibenzothiophene heterocycle were likewise all formed in good yield (**6 g**–**6 i**). Substitution on the third ring, the benzamide ring itself (coloured black), was also possible—significantly even with the substituent *ortho* to the amide, a position which imposes a significant conformational restraint (**6 j**).^[23]^

**Scheme 3:**
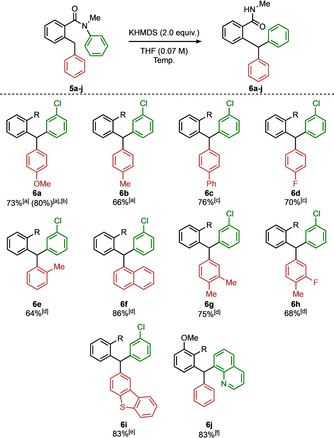
Scope of benzylic aryl groups (red and black aryl groups) for the Truce–Smiles rearrangement reaction. Reactions were carried out on a 0.20 mmol scale. Yields of product after isolation by chromatography. R=‐C(O)NHMe. Reactions requiring heating at 60 °C and below were performed thermally. Reactions requiring heating above 60 °C were performed in a microwave oven. [a] Performed at 50 °C. [b] Performed on a 3.7 mmol scale. [c] Performed at 55 °C. [d] Performed at 20 °C. [e] Performed at 30 °C. [f] Performed at 100 °C.

Alternative starting materials in which the migrating ring formed part of a heterocyclic precursor allowed the rearrangement to form products containing medium ring lactams (Scheme 4). Thus the tetrahydroquinoline **7 a** was readily expanded to the 10‐membered lactam **8 a** upon treatment of base. Similar reactivity was shown with the tetrahydronaphthyridine **7 b**, and with the benzo‐fused 7‐ring starting material **7 c**, as well as with the dihydro dibenzazepine‐derived **7 d**, each forming their respective 10 and 11‐membered lactams (**8 b**–**8 d**). The conformational restriction characteristic of medium rings gives them particular interest in medicinal chemistry,^[24]^ and ring expansion has proved to be a viable method for the preparation of medium‐ring heterocycles,^[25a]^ circumventing the difficulties associated with entropically and enthalpically unfavourable cyclisations.[^18b^, ^25^, ^26^, ^27^] **8 a**–**d** constitute the first reported examples of medium ring‐containing triarylmethane analogues.

**Scheme 4:**
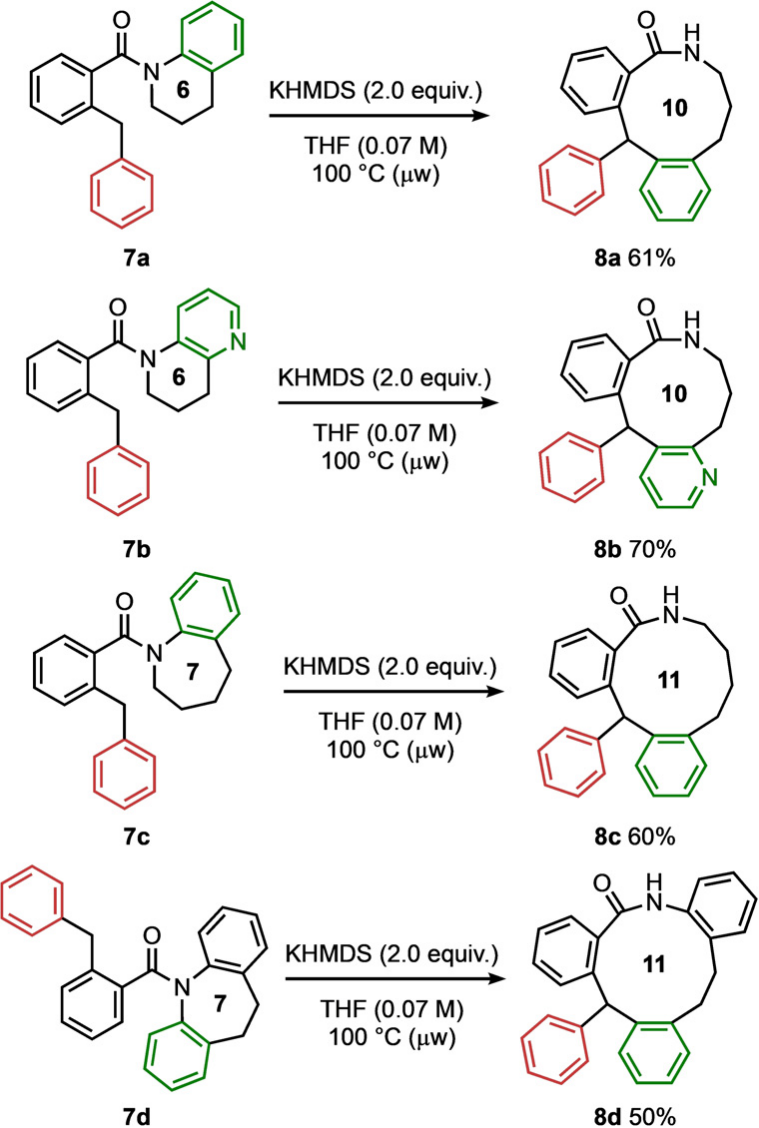
Scope of the ring‐expanding rearrangement reaction. Reactions were carried out on a 0.80 mmol scale. Yields of product after isolation by chromatography.

The rearrangement leads to products that carry a secondary amide substituent. To explore the potential broadening of this synthetic approach to a more general range of targets, the transformation of this group to other substituents (or none) was explored (Scheme 5). *N*‐Nitrosation of triarylmethane **6 a** formed *N*‐nitrosoamide **9**,^[28]^ converting the amide to an activated acyl group. In one pot, **6 a** was readily converted onwards to carboxylic acid **10** by basic hydrolysis. The carboxylic acid of **10** was removed by a copper‐catalysed decarboxylation,^[29]^ revealing the “traceless” triarylmethane product **11**. Alternatively, the *N*‐methylbenzamide of **6 a** could be used as a useful synthetic handle in, for example, an unoptimised palladium‐catalysed annulation to yield the *N*‐methyl isoquinolone **12**.^[30]^

**Scheme 5:**
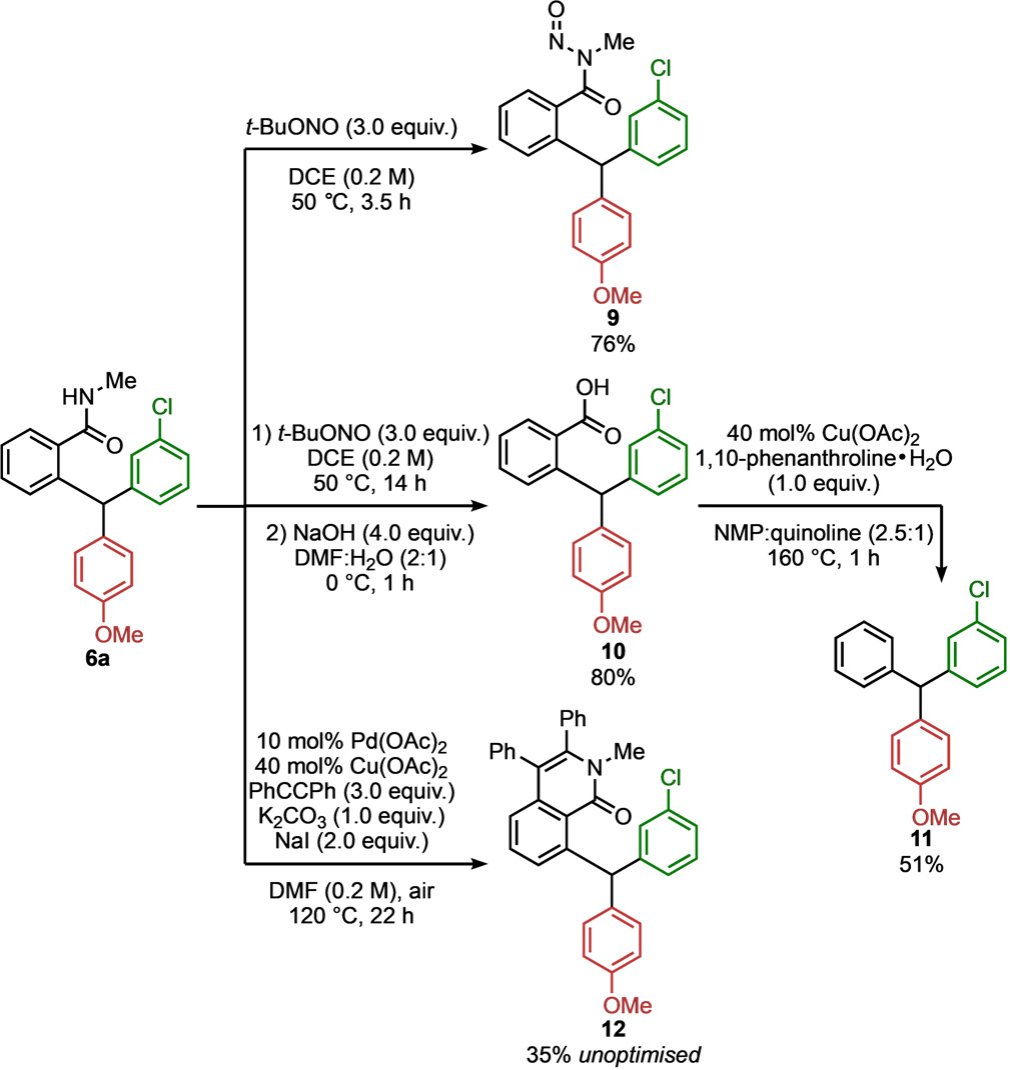
Synthetic modifications of rearrangement product **6 a**. Yields of product after isolation by chromatography.

Our proposed mechanism for the rearrangement that leads to the triarylmethane products starts with deprotonation of the 2‐benzylbenzamide **1** by KHMDS (Scheme 6 a). The DMSO *p*K_a_ values of HMDS (26)^[31]^ and diarylmethane (33.5–28)^[31]^ suggest that the equilibrium between 2‐benzylbenzamide **1** and carbanion **13** may lie in favour of the protonated form, although the carbanion is additionally stabilised by the amide group. Nonetheless, enough carbanion must be formed to allow the rate of attack on the migrating ring, which initiates an intramolecular S_N_Ar reaction, to be sufficiently high that it is synthetically useful. Protonation of product amide anion **14** by the aqueous quench yields the desired triarylmethane product.

**Scheme 6:**
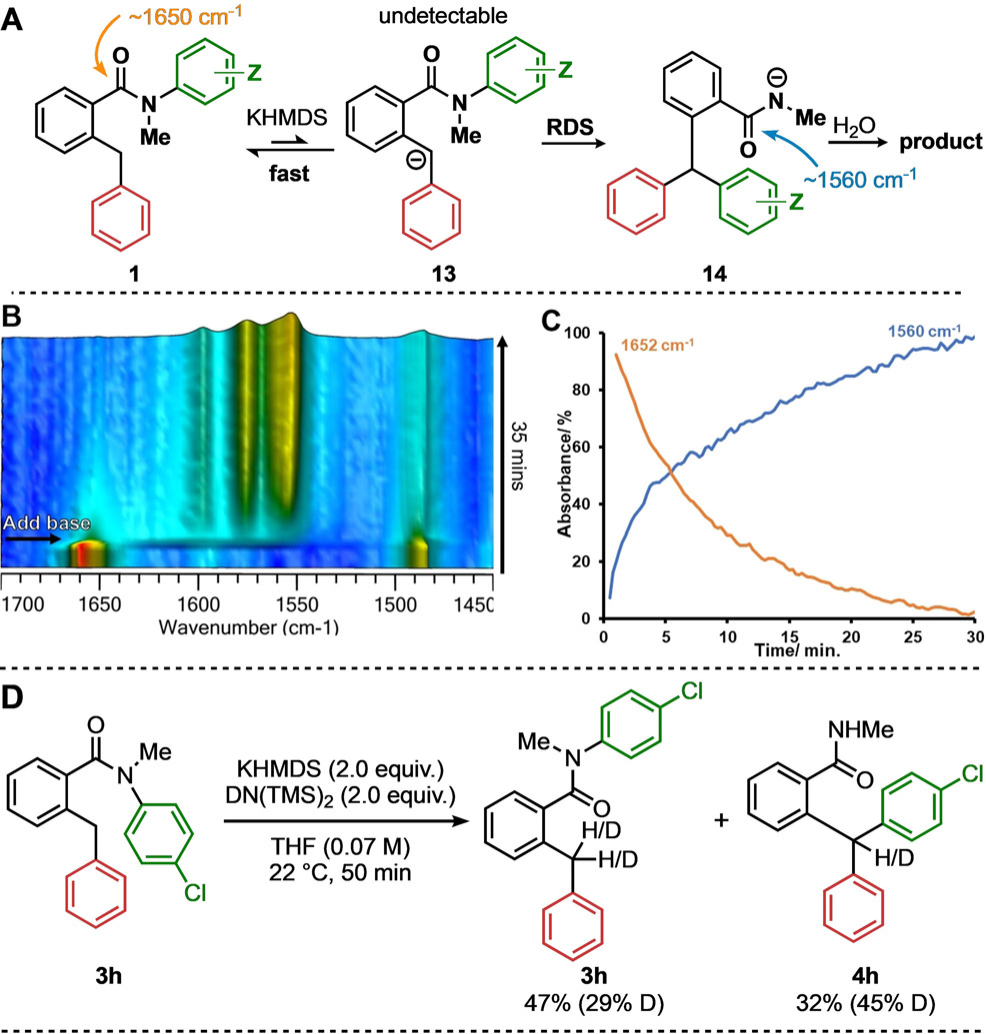
A) Proposed reaction pathway, with approximate C=O stretching frequencies. B) In situ infra‐red trace of the reaction of *N*‐aryl 2‐benzylbenzamide **3 h**, showing diagnostic changes in carbonyl‐stretching frequencies. C) Plot of absorbance against time for peaks at 1652 cm^−1^ (**3 h**, starting material) and 1560 cm^−1^ (**4 h**, product anion). D) Deuterium‐exchange study with *N*‐aryl 2‐benzylbenzamide **3 h**.

Further details of the reaction mechanism were explored by in situ infra‐red spectroscopy (React‐IR). Addition of KHMDS to a solution of **3 h** in THF (Scheme 6 b) led to decay of the amide carbonyl stretching frequency (1652 cm^−1^) as a new absorption appeared at 1560 cm^−1^ (Scheme 6 c). This band was assigned to the carbonyl group of the amide anion of **4 h**, confirmed by addition of KHMDS to triarylmethane **4 h** to generate an authentic sample of its amide anion (See SI). No C=O signal corresponding to reaction intermediate **13** was seen, an observation consistent either with rate limiting C‐deprotonation, or with rate limiting rearrangement from an undetectably low concentration of the anion. These two possibilities were distinguished by carrying out the rearrangement of **3 h** in the presence of deuterated HMDS, quenching the reaction before complete consumption of starting material (Scheme 6 d). Deuterium incorporation at the benzylic position of **3 h** indicated that deprotonation is fast and reversible, confirming that the rearrangement (S_N_Ar) step is rate‐determining.

Classical nucleophilic aromatic substitution reactions require electron‐deficient migrating groups, yet the results presented in Schemes 2 and 4 suggest that the rate of this reaction is largely independent of the nature of the electrophilic ring. The role of electronic effects in this reaction was therefore investigated using a Hammett plot. KHMDS was added at 22 °C to a series of *N*‐aryl 2‐benzylbenzamides, with the formation of triarylmethane amide anion monitored by React‐IR. Under these conditions, the formation of the triarylmethane amide anion from the 2‐benzylbenzamide followed first‐order kinetics, and the linear section of a plot of ln([**14**]_∞_−[**14**]_*t*_) against time gave a rate constant *k*_obs_ for each substrate (Figure 1). A Hammett plot of log (*k*_R_/*k*_H_) versus the substituent constant *σ* ^−^ yielded a value for *ρ* of +4.0, indicating substantial build‐up of negative charge on the migrating ring during the reaction. This significant dependence on *σ* ^−^ explains why the products in Scheme 2 may be formed in good yield at much lower temperatures when the migrating ring is electron‐deficient. Nonetheless, the facility with which even the *p*‐OMe group of **4 a** undergoes intramolecular S_N_Ar points to the importance of the conformational restriction inherent in the amide starting materials as a major factor in accelerating the absolute values of these rate constants over corresponding intermolecular variants.^[22]^ Murphy and Tuttle^[32]^ have shown that the *ρ* value is insufficient as an indicator of whether a reaction proceeds by a Meisenheimer complex or by a concerted S_N_Ar reaction, but given the lack of stabilisation offered to any intermediate anion in most substrates the reaction very likely falls into the latter class, along with other similar recently described reactions.[^14^, ^15^, ^16^, ^17^, ^18^]

**Figure 1:**
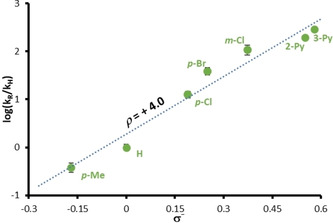
Hammett plot of log(*k*_R_/*k*_H_) against *σ* ^−^ at 22 °C, consistent with rate‐determining rearrangement. The gradient (*ρ* = +4.0) is consistent with substantial charge build‐up on the aryl substituent during the rearrangement. Error bars represent 1 standard deviation in a single direction.

In summary, a base‐mediated intramolecular S_N_Ar reaction of *N*‐aryl 2‐benzylbenzamides to form triarylmethanes of varied structure is made possible by conformationally induced rate acceleration of an otherwise apparently unfavourable intramolecular S_N_Ar reaction. This methodology is tolerant of a range of functional groups and aromatic rings, with the resulting triarylmethane products amenable to subsequent constructive or defunctionalising transformations. Novel medium ring‐containing triarylmethane scaffolds may be prepared by an *n* to *n*+4 ring expanding variant of the rearrangement. React‐IR, deuteration, and Hammett analysis support an empirical mechanistic model in which reversible deprotonation generated a low concentration of a benzyl anion which undergoes rate‐determining nucleophilic substitution on the migrating ring.

## Conflict of interest

The authors declare no conflict of interest.

## Supporting information

As a service to our authors and readers, this journal provides supporting information supplied by the authors. Such materials are peer reviewed and may be re‐organized for online delivery, but are not copy‐edited or typeset. Technical support issues arising from supporting information (other than missing files) should be addressed to the authors.

Supplementary
