# Supplementary material for: Triarylmethanes and their Medium‐Ring Analogues by Unactivated Truce–Smiles Rearrangement of Benzanilides
Source: Angew Chem Int Ed Engl. 2021 Apr 8;60(20):11272–7. doi: 10.1002/anie.202102192 (PMC8252078; doi:10.1002/anie.202102192)

Angewandte Chemie
Eine Zeitschrift der Gesellschaft Deutscher Chemiker

Supporting Information

Triarylmethanes and their Medium-Ring Analogues by Unactivated Truce–Smiles Rearrangement of Benzanilides

*Roman Abrams, Mehul H. Jesani, Alex Browning, and Jonathan Clayden**

anie_202102192_sm_miscellaneous_information.pdf

Contents

1. General Information....................2

1.1. Solvents, Reagents and Starting Materials....................2

1.2 Analytical Directions....................2

2. General Procedures....................4

3. Reaction Optimisation....................6

4. Synthetic Procedures for Starting Materials....................9

5. Synthetic Procedures for Products....................36

6. Mechanistic Studies....................66

6.1 Deuterium Labelling Studies....................66

6.2 Hammett Plot....................68

7. References....................105

8. NMR Spectra....................106

1. General Information

1.1. Solvents, Reagents and Starting Materials

Reactions requiring anhydrous conditions (where specified) were performed under an atmosphere of dry nitrogen in glassware that were dried by simultaneous use of vacuum and heat gun. Air- and moisture-sensitive liquids and solutions were transferred by syringe or cannula into reaction vessels through rubber septa. Reactions run in a microwave oven were completed on a Biotage Initiator+. All reagents were purchased at highest commercial quality and used as received. Non-anhydrous solvents were purchased at the highest commercial quality and used as received. Anhydrous toluene, diethyl ether, THF and DCM were purified by filtration over a column of activated alumina (A-2). Room temperature defines a temperature range of 20-23 °C.

1.2 Analytical Directions

Chromatography: Flash chromatography was performed on an automated Biotage IsoleraTM Spektra Four using gradient elution on pre-packed silica gel Biotage® SNAP Ultra or Biotage® Sfär columns. THF used in chromatography was distilled before use to remove BHT inhibitor.

Mp: Melting points were measured on a Scientific SMP10 melting point apparatus and are uncorrected.

IR spectroscopy: IR spectra were recorded on films of neat compounds deposited as a solution in DCM or $CDCl_3$, using a Perkin Elmer (Spectrum One) FT-IR spectrometer. Only strong and selected absorbances (ν_{max} expressed in cm^{-1}) are reported.

^{1}H NMR: Spectra were recorded on Bruker Avance (400 MHz or 500 MHz). Chemical shifts (δ_H) are quoted in parts per million (ppm) and referenced to the appropriate NMR solvent peak(s) and are assigned ArC*H*, C*H*, A of AB or B of AB (diastereotopic protons), CH_2, CH_3 and N*H*. 2D NMR experiments COSY, HSQC and HMBC were used where necessary in assigning NMR spectra. Spin-spin coupling constants (*J*) are reported in Hertz (Hz).

^{13}C NMR: Spectra were recorded on Bruker Avance (101 MHz or 126 MHz). Chemical shifts (δ_C) are quoted in parts per million (ppm) and referenced to the appropriate solvent peak(s) and are assigned C_{quat}, *C*H, CH_2, CH_3, CF_3, and Ar*C*X as determined using 2D NMR experiments HSQC and HMBC where necessary. Spin-spin coupling constants (*J*) are reported in Hertz (Hz).

^{19}F NMR: Spectra were recorded on Bruker Avance (376 MHz) instruments. Chemical shifts (δ_F) are quoted in parts per million (ppm) and referenced to an external standard.

HRMS: High resolution mass spectra were recorded on a Bruker Daltronics MicrOTOF 2 mass spectrometer (ESI).

In situ IR spectroscopy: Spectra were recorded on a ReactIR 15 instrument, under an inert atmosphere in anhydrous THF. Reported spectra and kinetic runs were allowed to stabilise for at least 20 minutes before being recorded.

2. General Procedures

General Procedure 1 (GP1): Synthesis of *N*-arylbenzamide analogues via acyl chloride formation.

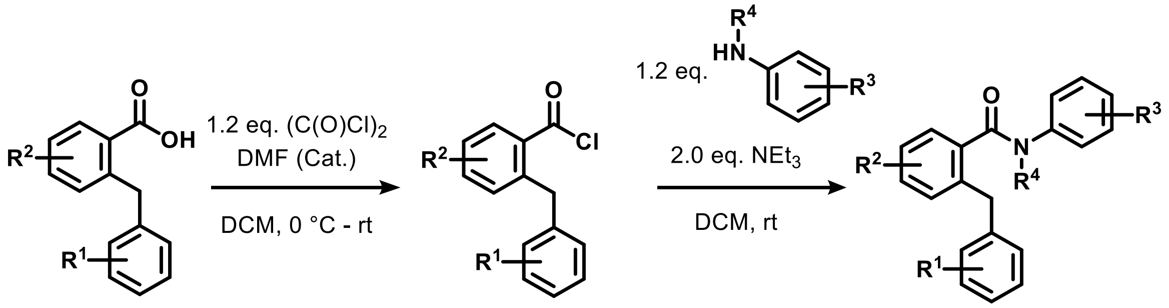

Scheme S1. Preparation of *N*-arylbenzamide derivatives.

A flame dried round bottom flask (RBF) was sequentially charged with nitrogen, benzoic acid derivative, anhydrous DCM (0.5 M) and a few drops of anhydrous DMF. The benzoic acid derivative solution was cooled to 0 °C then oxalyl chloride (2 M in DCM, 1.2 eq.) was added. The reaction mixture was warmed to room temperature and stirred for 18 hours. The reaction mixture was concentrated under reduced pressure to give crude acyl chloride which was used without further purification.

A flame dried round bottom flask was sequentially charged with nitrogen, aniline derivative (1.2 eq.), anhydrous DCM (0.5 M) and triethylamine (2.0 eq.). The crude acyl chloride was dissolved in DCM (0.5 M) and transferred to the aniline solution at room temperature by slow addition. The resulting reaction mixture was stirred for a defined amount of time, then diluted with DCM (50 mL) and sequentially washed with 1 M HCl (50 mL), 1 M NaOH (50 mL), then brine (50 mL). The organic extract was dried ($MgSO_4$), concentrated under reduced pressure and purified by flash chromatography (SiO_2) to give the desired product.

General Procedure 2 (GP2): Synthesis of triarylmethanes by Truce-Smiles rearrangement.

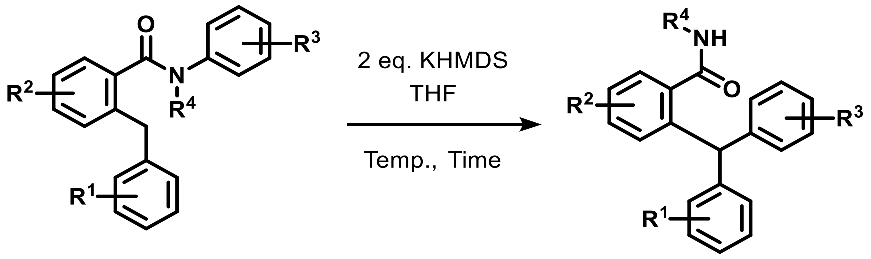

Scheme S2. Preparation of triarylmethane derivatives.

A flame dried microwave vial was charged sequentially with *N*-arylbenzamide derivative, nitrogen and anhydrous THF (0.08 M). The reaction mixture was degassed by sparging with nitrogen for 5 minutes, followed by addition of KHMDS (1 M in THF, 2.0 eq.). The reaction mixture was heated to a defined temperature (If 60 °C or below, performed thermally. If above 60 °C, performed in a microwave oven) and stirred for a defined amount of time. The reaction mixture was quenched with water and extracted with ethyl acetate (3 times). The combined organics were concentrated under reduced pressure and purified by flash column chromatography to yield the desired material.

3. Reaction Optimisation

2-Benzyl-*N*-methyl-*N*-phenylbenzamide (1)

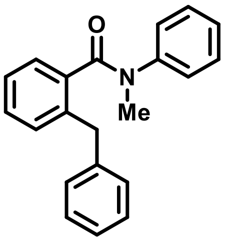

By following GP1, using 2-benzylbenzoic acid (1.06 g, 5.0 mmol), the acyl chloride intermediate was stirred with *N*-methylaniline (0.64 mL, 0.643 g, 6.0 mmol) for 5 hours and purified by flash chromatography (SiO_2, 0-100% ethyl acetate in 40-60 petroleum ether), affording the title compound (1.03 g, 69%) as a pale-yellow solid.

M.p. 107-108 °C (CH_2Cl_2). **IR (neat, cm^{-1}) ν_{max}** = 3071, 2922, 1635, 1494, 740, 701. **^{1}H NMR (400 MHz, $CDCl_3$, mixture of rotamers A:B in a 5:1 ratio)** δ 7.45-7.27 (3.02 H, m, 2 × ArC*H* rot. A + 8 × ArC*H* rot. B), 7.27-7.19 (3.34 H, m, 3 × ArC*H* rot. A + 5 × ArC*H* rot. B), 7.09 (4.32 H, br. s, 5 × ArC*H* rot. A + ArC*H* rot. B), 6.98 (1.66 H, br. s, 2 × ArC*H* rot. A), 6.65 (1.66 H, br. s, 2 × ArC*H* rot. A), 4.14 (2 H, s, CH_2), 3.49 (2.5 H, br. s, CH_3 rot. A), 2.84 (0.5 H, br. s, CH_3 rot. B). **^{13}C NMR (101 MHz, $CDCl_3$)** δ 170.7 (*C*O), 143.9 (C_{quat}), 140.3 (C_{quat}), 138.7 (C_{quat}), 136.1 (C_{quat}), 130.2 (Ar*C*H), 129.6 (2 × Ar*C*H), 128.9 (2 × Ar*C*H + Ar*C*H), 128.4 (2 × Ar*C*H), 128.3 (Ar*C*H), 126.7 (2 × Ar*C*H), 126.6 (Ar*C*H), 126.3 (Ar*C*H), 125.4 (Ar*C*H), 39.0 (*C*H_2), 37.5 (*C*H_3). **HRMS (ESI$^+$)** *m/z* calcd for $C_{21}H_{19}NONa^+$ $[M+Na]^+$ 324.1359; found 324.1371.

2-Benzhydryl-*N*-methylbenzamide (2)

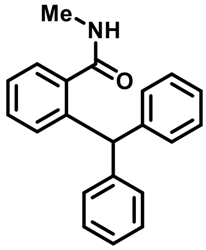

By following GP2, using 2-benzyl-*N*-methyl-*N*-phenylbenzamide (**1**) (60 mg, 0.2 mmol) as the aryl amide, heating the reaction mixture at 100 °C (μwave) for 1 hour, and purifying by flash chromatography (SiO_2, 0-100% diethyl ether in *n*-pentane), afforded the title compound (41 mg, 67%) as a white solid.

M.p. 139-140 °C (CH_2Cl_2). **IR (film, $CDCl_3$, cm^{-1}) ν_{max}** = 3291, 3025, 1635, 699. **^{1}H NMR (400 MHz, $CDCl_3$)** δ 7.50-7.19 (9 H, m, 2 × ArC*H* + 4 x ArC*H* + 3 × ArC*H*), 7.19-7.08 (4 H, m, 4 × ArC*H*), 7.03 (1 H, d, *J* 7.9, ArC*H*), 6.11 (1 H, s, C*H*), 5.24 (1 H, br. s, N*H*), 2.72 (3 H, br. d, *J* 4.8, C*H*$_3$). **^{13}C NMR (101 MHz, $CDCl_3$)** δ 170.8 (*C*O), 143.4 (2 × C_{quat}), 141.5 (C_{quat}), 137.6 (C_{quat}), 130.3 (Ar*C*H), 129.6 (4 × Ar*C*H), 129.5 (Ar*C*H), 128.4 (4 × Ar*C*H), 127.1 (Ar*C*H), 126.4 (2 × Ar*C*H + Ar*C*H), 52.6 (*C*H), 26.4 (*C*H$_3$). **HRMS (ESI$^+$)** *m/z* calcd for $C_{21}H_{20}NO^+$ $[M+H]^+$ 302.1539; found 302.1527.

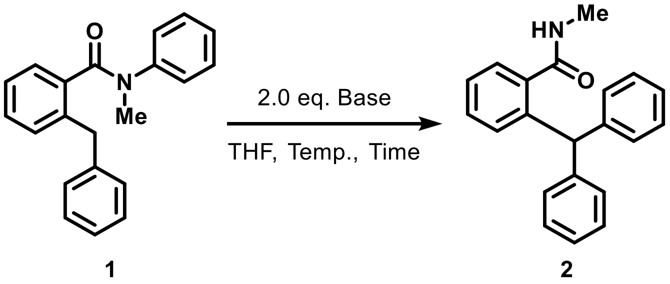

Entry	Base (equiv.)	Solvent	Temp./ °C	Time/ hr	Yield/ %[a]
1	NaHMDS (2)	THF	–50	16	0
2	NaHMDS (2)	THF	0	16	0
3	NaHMDS (2)	THF	20	16	<5
4	NaHMDS (2)	THF	40	16	12
5	NaHMDS (2)	THF	60	16	49
6	NaHMDS (2)	THF	100[b]	1	62
7	NaHMDS (2)	THF	100[b]	1	73[c]
8	LiHMDS (2)	THF	100[b]	1	62[c]
**9**	**KHMDS (2)**	**THF**	**100[b]**	**1**	**81[c]**
10	KHMDS (2)[d]	THF	100[b]	1	54[c]
11	KHMDS (2)	1,4-dioxane	100[b]	1	27[c]
12	KHMDS (2)	TMBE	100[b]	1	30[c]
13	KHMDS (2)	Et_2O	100[b]	1	80[c]
14	KHMDS (2)	Toluene	100[b]	1	39[c]
15	KHMDS (1.5)	THF	100[b]	1	72[c]
16	KHMDS (1.1)	THF	100[b]	1	70[c]
17	KHMDS (2)	THF	120[b]	1	69[c]
18	KHMDS (2)	THF	140[b]	1	75[c]
19[e]	**KHMDS (2)**	**THF**	**100[b]**	**1**	**67**

Table S1. Optimisation studies. All reactions were performed on a 0.1 mmol scale following GP2. [a] Isolated yield. [b] Reaction performed in a microwave oven. [c] Yields calculated from ^{1}H NMR using 1,3,5-trimethoxybenzene as an internal standard. [d] with 18-crown-6 (0.2 mmol). [e] 0.2 mmol reaction scale.

4. Synthetic Procedures for Starting Materials

2-Benzyl-*N*-(4-methoxyphenyl)-*N*-methylbenzamide (3a)

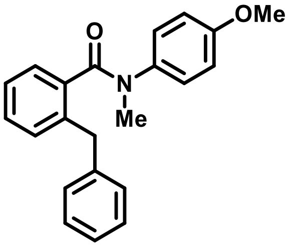

By following GP1, using 2-benzylbenzoic acid (1.06 g, 5.0 mmol), the acyl chloride intermediate was stirred with 4-methoxy-*N*-methylaniline (823 mg, 6.0 mmol) for 16 hours and purified by flash chromatography (SiO_2, 0-100% ethyl acetate in 40-60 petroleum ether), affording the title compound (1.30 g, 79%) as a brown solid.

M.p. 88-89 °C (CH_2Cl_2). **IR (neat, cm^{-1}) ν_{max}** = 3024, 2933, 1638, 1509, 1247, 742. **^{1}H NMR (400 MHz, $CDCl_3$, mixture of rotamers A:B in a 5:1 ratio)** δ 7.39-7.27 (3.18 H, m, 2 × ArC*H* rot. A + 9 × ArC*H* rot. B), 7.27-7.19 (2.5 H, m, 3 × ArC*H* rot. A), 7.19-7.02 (2 H, m, 2 × ArC*H* rot. A + 2 × ArC*H* rot. B), 7.02-6.89 (2 H, m, 2 × ArC*H* rot. A + 2 × ArCH rot. B), 6.59 (1.66 H, d, *J* 8.6, 2 × ArC*H* rot. A), 6.54 (1.66 H, d, *J* 8.6, 2 × ArC*H* rot. A), 4.12 (2 H, s, C*H*$_2$), 3.84 (0.5 H, s, OC*H*$_3$ rot. B), 3.71 (2.5 H, s, OC*H*$_3$ rot. A), 3.44 (2.5 H, s, C*H*$_3$ rot. A), 2.78 (0.5 H, s, C*H*$_3$ rot. B). **^{13}C NMR (101 MHz, $CDCl_3$)** δ 170.8 (*C*O), 157.9 (Ar*C*O), 140.4 (C_{quat}), 138.6 (C_{quat}), 136.9 (C_{quat}), 136.3 (C_{quat}), 130.1 (Ar*C*H), 129.6 (2 × Ar*C*H), 128.7 (Ar*C*H), 128.4 (2 × Ar*C*H), 128.2 (Ar*C*H), 127.9 (2 × Ar*C*H), 126.2 (Ar*C*H), 125.4 (Ar*C*H), 114.0 (2 × Ar*C*H), 55.3 (O*C*H$_3$), 39.0 (*C*H$_2$), 37.6 (*C*H$_3$). **HRMS (ESI$^+$)** *m/z* calcd for $C_{22}H_{21}NO_2Na$+ [M+Na]$^+$ 354.1464; found 354.1450.

2-Benzyl-*N*-methyl-*N*-(*p*-tolyl)benzamide (3b)

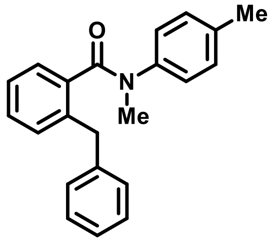

By following GP1, using 2-benzylbenzoic acid (1.06 g, 5.0 mmol), the acyl chloride intermediate was stirred with *N*,4-dimethylaniline (0.76 mL, 727 mg, 6.0 mmol) for 16 hours and purified by flash chromatography (SiO_2, 0-100% ethyl acetate in 40-60 petroleum ether), affording the title compound (900 mg, 57%) as a pale-yellow solid.

M.p. 96-98 °C (CH_2Cl_2). **IR (neat, cm^{-1}) ν_{max}** = 2978, 2913, 1631, 1375, 1075, 742. **^{1}H NMR (400 MHz, $CDCl_3$, mixture of rotamers A:B in a 5:1 ratio)** δ 7.42-7.28 (2.68 H, m, 2 × ArC*H* rot. A + 6 × ArC*H* rot. B), 7.28-7.19 (3.34 H, m, 3 × ArC*H* rot. A + 5 × ArC*H* rot. B), 7.18-7.04 (2 H, m, 2 × ArC*H* rot. A + 2 × ArC*H* rot. B), 7.04-6.93 (1.66 H, m, 2 × ArC*H* rot. A), 6.90 (1.66 H, d, *J* 7.8, 2 × ArC*H* rot. A), 6.54 (1.66 H, d, *J* 7.8, 2 × ArC*H* rot. A), 4.15 (2 H, s, CH_2), 3.47 (2.5 H, s, CH_3 rot. A), 2.81 (0.5 H, br. s, CH_3 rot. B), 2.38 (0.5 H, br. s, ArCCH_3 rot. B), 2.24 (2.5 H, s, ArCCH_3, rot. A). **^{13}C NMR (101 MHz, $CDCl_3$)** δ 170.7 (*C*O), 141.4 (C_{quat}), 140.4 (C_{quat}), 138.7 (C_{quat}), 136.4 (C_{quat}), 136.3 (C_{quat}), 130.1 (Ar*C*H), 129.6 (2 × Ar*C*H), 129.5 (2 × Ar*C*H), 128.8 (Ar*C*H), 128.4 (2 × Ar*C*H), 128.2 (Ar*C*H), 126.5 (2 × Ar*C*H), 126.3 (Ar*C*H), 125.4 (Ar*C*H), 39.1 (*C*H_2), 37.5 (*C*H_3), 20.9 (ArC*C*H_3). **HRMS (ESI$^+$)** *m/z* calcd for $C_{22}H_{21}NONa^+$ $[M+Na]^+$ 338.1515; found 338.1500.

2-Benzyl-*N*-methyl-*N*-(*o*-tolyl)benzamide (3c)

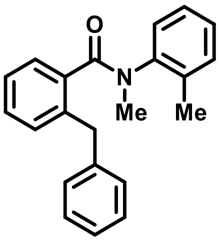

By following GP1, using 2-benzylbenzoic acid (1.06 g, 5.0 mmol), the acyl chloride intermediate was stirred with *N*,2-dimethylaniline (0.741 mL, 0.727 g, 6.0 mmol) for 16 hours and purified by flash chromatography (SiO_2, 0-30% diethyl ether in 40-60 petroleum ether), affording the title compound (0.811 g, 51%) as a white solid.

M.p. 90-92 °C ($CDCl_3$). **IR (film, CH_2Cl_2, cm^{-1})** ν_{max} = 2921, 1640, 1492, 1362. **^{1}H NMR (400 MHz, $CDCl_3$, mixture of rotamers A:B in a 0.69:0.31 ratio)** δ 7.38-7.19 (7.48 H, m, 5 x ArC*H* rot. A + 13 x ArC*H* rot. B), 7.10 (0.69 H, d, *J* 7.6, ArC*H* rot. A), 7.07-7.04 (1.38 H, m, 2 x ArC*H* rot. A), 7.00 (0.69 H, td, *J* 7.6, 1.1, ArC*H* rot. A), 6.87-6.81 (1.38 H, m, 2 x ArC*H* rot. A), 6.77 (0.69 H, br. t, *J* 7.6, ArC*H* rot. A), 6.09 (0.69 H, d, *J* 7.7, ArC*H* rot. A), 4.37 (0.69 H, d, *J* 15.0, CH_aH$_b$ rot. A), 4.29 (0.31 H, br. d, *J* 14.5, CH_aH$_b$ rot. B), 4.13-4.04 (0.31 H, m, CH$_a$$H_b$ rot. B), 4.02 (0.69 H, d, *J* 15.0, CH$_a$$H_b$ rot. A), 3.33 (2.07 H, s, NCH_3 rot. A), 2.82 (0.93 H, s, NCH_3 rot. B), 2.33 (2.07 H, s, ArCCH_3 rot. A), 2.29 (0.93 H, s, ArCCH_3 rot. B). **^{13}C NMR (101 MHz, $CDCl_3$)** δ 171.0 (*C*O rot. A), 170.7 (*C*O rot. B), 142.8 (C_{quat} rot. A), 141.6 (C_{quat} rot. B), 140.7 (C_{quat} rot. A), 140.5 (C_{quat} rot. B), 139.2 (C_{quat} rot. A + C_{quat} rot. B), 136.7 (C_{quat} rot. B), 135.9 (C_{quat} rot. A), 135.4 (Ar*C*CH$_3$ rot. B), 134.5 (Ar*C*CH$_3$ rot. A), 131.2 (Ar*C*H rot. B), 131.1 (Ar*C*H rot. A + Ar*C*H rot. B), 130.4 (Ar*C*H rot. A), 129.8 (2 x Ar*C*H rot. A), 129.5 (2 x Ar*C*H rot. B), 129.3 (Ar*C*H rot. B), 129.0 (Ar*C*H rot. A), 128.6 (Ar*C*H rot. A), 128.5 (2 x Ar*C*H rot. A + 2 x Ar*C*H rot. B), 127.9 (Ar*C*H rot. B), 127.7 (Ar*C*H rot. A), 127.3 (Ar*C*H rot. B), 126.8 (Ar*C*H rot. A + Ar*C*H rot. B), 126.7 (Ar*C*H rot. A), 126.5 (Ar*C*H rot. B), 126.5 (Ar*C*H rot. B), 126.4 (Ar*C*H rot. A + Ar*C*H rot. B), 125.2 (Ar*C*H rot. A), 39.9 (N*C*H$_3$ rot. B), 39.2 (*C*H$_2$ rot. A), 39.0 (*C*H$_2$ rot. B), 36.8 (N*C*H$_3$ rot. A), 18.1 (ArC*C*H$_3$ rot. A), 17.8 (ArC*C*H$_3$ rot. B). **HRMS (ESI$^+$)** *m/z* calcd for $C_{22}H_{22}NO^+$ [M+H]$^+$ 316.1696; found 316.1695.

2-Benzyl-*N*-methyl-*N*-(naphthalen-1-yl)benzamide (3d)

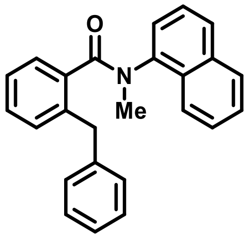

By following GP1, using 2-benzylbenzoic acid (1.06 g, 5.0 mmol), the acyl chloride intermediate was stirred with *N*-methylnaphthalen-1-amine hydrochloride (1.16 g, 6.0 mmol) and triethylamine (1.55 mL, 15.0 mmol, 3.0 eq.) for 20 hours and purified by flash chromatography (SiO_2, 0-100% ethyl acetate in 40-60 petroleum ether), affording the title compound (1.11 g, 63%) as a white solid.

M.p. 118-119 °C (CH_2Cl_2). **IR (film, $CDCl_3$, cm^{-1}) ν_{max}** = 3024, 2914, 1641, 1360, 775, 699. **^{1}H NMR (400 MHz, $CDCl_3$, mixture of rotamers A:B in a 1:0.12 ratio)** δ 8.05 (0.89 H, d, *J* 8.4, ArC*H* rot. A), 7.92 (0.11 H, d, *J* 8.4, ArC*H* rot. B), 7.84 (1 H, d, *J* 8.2, ArC*H* rot. A + ArC*H* rot. B), 7.69-7.58 (2 H, m, 2 × ArC*H* rot. A + 2 × ArC*H* rot. B), 7.58-7.49 (1.22 H, m, ArC*H* rot. A + 3 × ArC*H* rot. B), 7.47-7.23 (5.79 H, m, 6 × ArC*H* rot. A + 4 × ArC*H* rot. B), 7.09-6.95 (2.33 H, m, 2 × ArC*H* rot. A + 5 × ArC*H* rot. B), 6.83 (0.89 H, d, *J* 7.7, ArC*H* rot. A), 6.63 (0.89 H, t, *J* 7.5, ArC*H* rot. A), 6.15 (0.89 H, d, *J* 7.3, ArC*H* rot. A), 4.50 (0.89 H, d, *J* 14.8, CH_aH_b rot. A), 4.41-4.30 (0.22 H, m, CH_aH_b rot. B + CH_aH_b rot. B), 4.06 (0.89 H, d, *J* 14.9, CH_aH_b rot. A), 3.53 (2.67 H, s, CH_3 rot. A), 3.05 (0.33 H, s, CH_3, rot. B). **^{13}C NMR (101 MHz, $CDCl_3$)** δ 171.6 (*C*O rot. A), 171.5 (*C*O rot. B), 140.6 (C_{quat} rot. A), 140.5 (C_{quat} rot. B), 140.2 (C_{quat} rot. A), 139.6 (C_{quat} rot. B), 138.9 (C_{quat} rot. A), 136.6 (C_{quat} rot. B), 135.9 (C_{quat} rot. A), 134.8 (C_{quat} rot. B), 134.4 (C_{quat} rot. A), 131.3 (C_{quat} rot. B), 130.2 (Ar*C*H rot. A), 130.0 (C_{quat} rot. A), 129.7 (2 × Ar*C*H rot. A), 129.5 (C_{quat} rot. B), 129.4 (Ar*C*H rot. B), 129.3 (2 x Ar*C*H rot. B), 128.9 (Ar*C*H rot. A), 128.7 (Ar*C*H rot. A + Ar*C*H rot. B), 128.6 (2 × Ar*C*H rot. B), 128.4 (2 × Ar*C*H rot. A), 128.3 (Ar*C*H rot. B), 128.1 (Ar*C*H rot. A), 127.2 (Ar*C*H rot. A), 126.9 (Ar*C*H rot. B), 126.7 (Ar*C*H rot. B), 126.5 (Ar*C*H rot. B), 126.3 (Ar*C*H rot. B), 126.3 (3 × Ar*C*H rot. A + Ar*C*H rot. B), 126.3 (Ar*C*H rot. B), 125.9 (Ar*C*H rot. A), 125.9 (Ar*C*H rot. B), 125.4 (Ar*C*H rot. A), 125.1 (Ar*C*H rot. A), 125.0 (Ar*C*H rot. B) 122.8 (Ar*C*H rot. A), 122.3 (Ar*C*H rot. B), 40.8 (CH_3 rot. B), 39.2 (CH_2 rot. A), 39.0 (CH_2 rot. B), 37.6 (CH_3 rot. A). **HRMS (ESI$^+$)** *m/z* calcd for $C_{25}H_{22}NO^+$ $[M+H]^+$ 352.1696; found 352.1681.

2-Benzyl-*N*-(3,5-difluorophenyl)-*N*-methylbenzamide (3e)

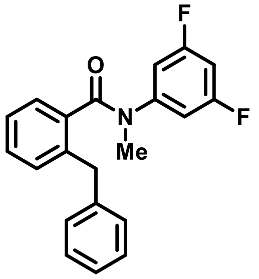

By following GP1, using 2-benzylbenzoic acid (1.06 g, 5.0 mmol), the acyl chloride intermediate was stirred with 3,5-difluoro-*N*-methylaniline (859 mg, 6.0 mmol) for 20 hours and purified by flash chromatography (SiO_2, 0-100% ethyl acetate in 40-60 petroleum ether), affording the title compound (1.41 g, 84%) as a white solid.

M.p. 126-127 °C (CH_2Cl_2). **IR (film, $CDCl_3$, cm^{-1})** ν_{max} = 3080, 3029, 1645, 1613, 1334, 748. **^{1}H NMR (400 MHz, $CDCl_3$)** δ 7.41-7.17 (7 H, m, 7 × ArC*H*), 7.08 (1 H, br. s, ArC*H*), 7.03 (1 H, br. s, ArC*H*), 6.57 (1 H, br. t, *J* 8.9, ArC*H*), 6.22 (2 H, br. s, 2 × ArC*H*), 4.17 (2 H, s, CH_2), 3.25 (3 H, br. s, CH_3). **^{13}C NMR (101 MHz, $CDCl_3$)** δ 170.4 (*C*O), 162.6 (dd, J_{CF} 248.9, 14.4, 2 x Ar*C*F), 145.8 (C_{quat}), 140.1 (C_{quat}), 139.1 (C_{quat}), 135.4 (C_{quat}), 130.8 (Ar*C*H), 129.5 (Ar*C*H), 129.5 (2 × Ar*C*H), 128.6 (2 × Ar*C*H), 127.6 (Ar*C*H), 126.5 (Ar*C*H), 125.9 (Ar*C*H), 109.7 (br. d, J_{CF} 25.3, 2 x Ar*C*H), 102.1 (t, J_{CF} 25.4, Ar*C*H), 39.1 (*C*H_2), 37.8 (*C*H_3). **^{19}F NMR (377 MHz, $CDCl_3$)** δ −108.8 (s, 2 × ArC*F*). **HRMS (ESI$^+$)** *m/z* calcd for $C_{21}H_{18}F_2NO^+$ $[M+H]^+$ 338.1351; found 338.1348.

2-Benzyl-*N*-methyl-*N*-(4-(trifluoromethyl)phenyl)benzamide (3f)

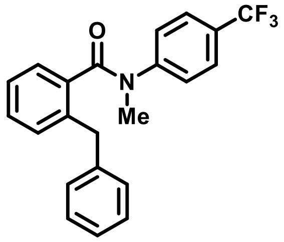

By following GP1, using 2-benzylbenzoic acid (1.06 g, 5.0 mmol), the acyl chloride intermediate was stirred with *N*-methyl-4-(trifluoromethyl)aniline (0.85 mL, 1.05 g, 6.0 mmol) for 20 hours and purified by flash chromatography (SiO_2, 0-100% ethyl acetate in 40-60 petroleum ether), affording the title compound (1.12 g, 61%) as a white solid.

M.p. 75-77 °C (CH_2Cl_2). **IR (film, $CDCl_3$, cm^{-1}) ν_{max} =** 3025, 2920, 1649, 1324, 1120, 742. **^{1}H NMR (400 MHz, $CDCl_3$)** δ 7.41 (2 H, br. s, 2 × ArC*H*), 7.37-7.28 (2 H, m, 2 × ArC*H*), 7.28-7.17 (5 H, m, 5 × ArC*H*), 7.05 (2 H, br. s, 2 × ArC*H*), 6.77 (2 H, br. s, 2 × ArC*H*), 4.18 (2 H, s, C*H*$_2$), 3.36 (3 H, br. s, C*H*$_3$). **^{13}C NMR (101 MHz, $CDCl_3$)** δ 170.6 (*C*O), 146.8 (C_{quat}), 140.1 (C_{quat}), 139.0 (C_{quat}), 135.6 (C_{quat}), 130.7 (Ar*C*H), 129.5 (2 × Ar*C*H), 129.4 (Ar*C*H), 128.5 (2 × Ar*C*H), 128.3 (q, J_{CF} 32.6, C_{quat}), 127.8 (Ar*C*H), 126.4 (3 × Ar*C*H), 126.0 (q, J_{CF} 3.7, 2 × Ar*C*H), 125.9 (Ar*C*H), 123.8 (q, J_{CF} 272.0, *C*F$_3$), 39.1 (*C*H$_2$), 37.7 (*C*H$_3$). **^{19}F NMR (377 MHz, $CDCl_3$)** δ –62.5 (s, C*F*$_3$). **HRMS (ESI$^+$)** *m/z* calcd for $C_{22}H_{18}F_3NONa^+$ $[M+Na]^+$ 392.1233; found 392.1244.

2-Benzyl-*N*-(3-chlorophenyl)-*N*-methylbenzamide (3g)

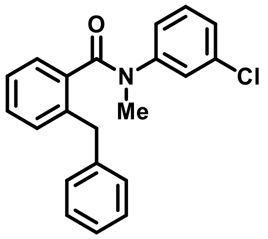

By following GP1, using 2-benzylbenzoic acid (1.06 g, 5.0 mmol), the acyl chloride intermediate was stirred with 3-chloro-*N*-methylaniline (0.73 mL, 850 mg, 6.0 mmol) for 20 hours and purified by flash chromatography (SiO_2, 0-100% ethyl acetate in 40-60 petroleum ether), affording the title compound (940 mg, 56%) as a pale brown solid.

M.p. 82-83 °C (CH_2Cl_2). **IR (film, $CDCl_3$, cm^{-1}) v_{max}** = 3025, 2922, 1646, 1590, 1357, 742, 698. **^{1}H NMR (400 MHz, $CDCl_3$, mixture of rotamers A + B in an indiscernible ratio)** δ 7.36-7.28 (2.5 H, m, 2.5 × ArC*H*), 7.28-7.14 (5 H, m, 5 × ArC*H*), 7.13-6.85 (4 H, m, 4 × ArC*H*), 6.78-6.18 (1.5 H, m, 1.5 × ArC*H*), 4.16 (2 H, s, C*H*$_2$), 3.81-2.43 (3 H, m, C*H*$_3$ rot. A + C*H*$_3$ rot. B). **^{13}C NMR (101 MHz, $CDCl_3$)** δ 170.5 (*C*O), 144.9 (C_{quat}), 140.2 (C_{quat}), 139.0 (C_{quat}), 135.6 (C_{quat}), 134.3 (Ar*C*Cl), 130.6 (Ar*C*H), 129.8 (Ar*C*H), 129.5 (2 × Ar*C*H), 129.2 (Ar*C*H), 128.6 (2 × Ar*C*H), 128.0 (Ar*C*H), 126.8 (Ar*C*H), 126.7 (Ar*C*H), 126.4 (Ar*C*H), 125.7 (Ar*C*H), 124.8 (Ar*C*H), 39.1 (*C*H$_2$), 37.5 (*C*H$_3$). **HRMS (ESI$^+$)** *m/z* calcd for $C_{21}H_{18}ClNONa^+$ $[M+Na]^+$ 358.0969; found 358.0968.

2-Benzyl-*N*-(4-chlorophenyl)-*N*-methylbenzamide (3h)

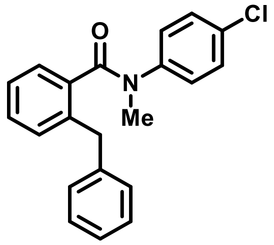

By following GP1, using 2-benzylbenzoic acid (1.06 g, 5.0 mmol), the acyl chloride intermediate was stirred with 4-chloro-*N*-methylaniline (0.73 mL, 850 mg, 6.0 mmol) for 16 hours and purified by flash chromatography (SiO_2, 0-100% ethyl acetate in 40-60 petroleum ether), affording the title compound (1.07 g, 64%) as a white solid.

M.p. 88-90 °C (CH_2Cl_2). **IR (neat, cm^{-1}) ν_{max}** = 3011, 2933, 1640, 1493, 744, 700. **^{1}H NMR (400 MHz, $CDCl_3$, mixture of rotamers A:B in a 5:1 ratio)** δ 7.44-7.29 (2.68 H, m, 2 × ArC*H* rot. A + 6 × ArC*H* rot. B), 7.28-7.21 (3.49 H, m, 4 × ArC*H* rot. A + ArC*H* rot. B), 7.21-7.11 (1.83 H, m, 2 × ArC*H* rot. A + ArC*H* rot. B), 7.11-6.76 (3.34 H, m, 3 × ArC*H* rot. A + 5 × ArC*H* rot. B), 6.46 (1.66 H, br. s, 2 × ArC*H* rot. A), 4.16 (2 H, s, CH_2), 3.45 (2.5 H, br. s, CH_3 rot. A), 2.80 (0.5 H, br. s, CH_3 rot. B). **^{13}C NMR (101 MHz, $CDCl_3$)** δ 170.5 (*C*O), 142.5 (C_{quat}), 140.2 (C_{quat}), 138.9 (C_{quat}), 135.7 (C_{quat}), 132.1 (Ar*C*Cl), 130.5 (Ar*C*H), 129.5 (2 × Ar*C*H), 129.2 (Ar*C*H), 129.0 (2 × Ar*C*H), 128.5 (2 × Ar*C*H), 128.3 (Ar*C*H), 127.9 (2 × Ar*C*H), 126.4 (Ar*C*H), 125.6 (Ar*C*H), 39.1 (CH_2), 37.5 (CH_3). **HRMS (ESI$^+$)** *m/z* calcd for $C_{21}H_{18}ClNONa^+$ [M+Na]$^+$ 358.0969; found 358.0965.

2-Benzyl-*N*-(4-bromophenyl)-*N*-methylbenzamide (3i)

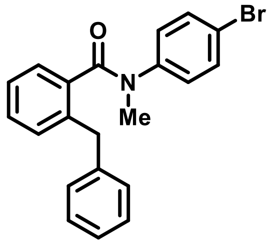

By following GP1, using 2-benzylbenzoic acid (1.06 g, 5.0 mmol), the acyl chloride intermediate was stirred with 4-bromo-*N*-methylaniline (1.00 g, 6.0 mmol) for 20 hours and purified by flash chromatography (SiO_2, 0-30% diethyl ether in 40-60 petroleum ether), affording the title compound (1.11 g, 58%) as a light brown solid.

M.p. 91-93 °C ($CDCl_3$). **IR (film, CH_2Cl_2, cm^{-1})** ν_{max} = 2918, 1640, 1489, 1359. **^{1}H NMR (400 MHz, $CDCl_3$, mixture of rotamers A:B in a 0.85:0.15 ratio)** δ 7.48-7.35 (0.45 H, m, 3 x ArC*H* rot. B), 7.34-7.23 (3 H, m, 3 x ArC*H* rot. A + 3 x ArC*H* rot. B), 7.23-7.17 (3.55 H, m, 4 x ArC*H* rot. A + ArC*H* rot. B), 7.14 (2.70 H, br. s, 3 x ArC*H* rot. A + ArC*H* rot. B), 6.92 (2 H, br. s, 2 x ArC*H* rot. A + 2 x ArC*H* rot. B), 6.34 (1.30 H, br. s, ArC*H* rot. A + 3 x ArC*H* rot. B), 4.11 (2 H, s, CH_2 rot. A + CH_2 rot. B), 3.40 (2.5 H, s, CH_3 rot. A), 2.73 (0.5 H, s, CH_3 rot. B). **^{13}C NMR (101 MHz, $CDCl_3$)** δ 170.5 (*C*O), 143.1 (C_{quat}), 140.3 (C_{quat}), 139.1 (C_{quat}), 135.7 (C_{quat}), 132.1 (2 x Ar*C*H), 130.6 (Ar*C*H), 129.6 (2 x Ar*C*H), 129.3 (Ar*C*H), 128.6 (2 x Ar*C*H), 128.3 (2 x Ar*C*H), 126.5 (Ar*C*H + Ar*C*H), 125.7 (Ar*C*H), 120.1 (Ar*C*Br), 39.1 (*C*H_2), 37.5 (*C*H_3). **HRMS (ESI$^+$)** *m/z* calcd for $C_{21}H_{19}NOBr^+$ $[M+H]^+$ 380.0645; found 380.0636.

2-Benzyl-*N*-methyl-*N*-(pyridin-4-yl)benzamide (3j)

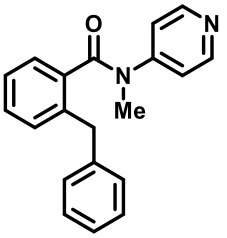

By following GP1, using 2-benzylbenzoic acid (1.06 g, 5.0 mmol), the acyl chloride intermediate was stirred with *N*-methylpyridin-4-amine (0.649 g, 6.0 mmol) for 21 hours and purified by flash chromatography (0-30% acetone in 40-60 petroleum ether), affording the title compound (995 mg, 67%) as a white solid.

M.p. 85-88 °C ($CDCl_3$). **IR (film, CH_2Cl_2, cm^{-1})** ν_{max} = 3026, 1651, 1585, 1355. **^{1}H NMR (400 MHz, $CDCl_3$)** δ 8.35 (2 H, d, *J* 5.6, 2 x ArC*H*), 7.30-7.24 (4 H, m, 2 x ArC*H* + ArC*H* + ArC*H*), 7.21-7.17 (3 H, m, 2 x ArC*H* + ArC*H*), 7.08 (1 H, t, *J* 7.3, ArC*H*), 7.02 (1 H, d, *J* 7.3, ArC*H*), 6.68 (2 H, br. s, 2 x ArC*H*), 4.10 (2 H, s, CH_2), 3.22 (3 H, s, CH_3). **^{13}C NMR (101 MHz, $CDCl_3$)** δ 170.8 (*C*O), 150.7 (C_{quat}), 150.6 (2 x Ar*C*H), 140.0 (C_{quat}), 139.1 (C_{quat}), 135.5 (C_{quat}), 130.9 (Ar*C*H), 129.9 (Ar*C*H), 129.5 (2 x Ar*C*H), 128.6 (2 x Ar*C*H), 127.7 (Ar*C*H), 126.6 (Ar*C*H), 126.2 (Ar*C*H), 119.6 (2 x Ar*C*H), 39.2 (CH_2), 37.0 (NCH_3). **HRMS (ESI$^+$)** *m/z* calcd for $C_{20}H_{19}N_2O^+$ $[M+H]^+$ 303.1492; found 303.1489.

2-Benzyl-*N*-methyl-*N*-(pyridin-3-yl)benzamide (3k)

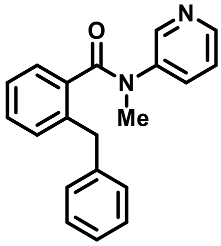

By following GP1, using 2-benzylbenzoic acid (1.06 g, 5.0 mmol), the acyl chloride intermediate was stirred with *N*-methylpyridin-3-amine (649 mg, 6.0 mmol) for 16 hours and purified by flash chromatography (SiO_2, 0-8% MeOH in DCM, then again by 0-30% acetone in 40-60 petroleum ether), affording the title compound (0.328 g, 22%) as a white solid.

M.p. 84-86 °C ($CDCl_3$). **IR (film, CH_2Cl_2, cm^{-1}) ν_{max}** = 2921, 1647, 1492, 1362. **^{1}H NMR (400 MHz, $CDCl_3$, mixture of rotamers A:B in a 0.83:0.17 ratio)** δ 8.66-8.15 (1.17 H, m, ArC*H* rot. A + 2 x ArC*H* rot. B), 7.88 (0.83 H, br. s, ArC*H* rot. A), 7.44-7.25 (3.17 H, m, 3 x ArC*H* rot. A + 4 x ArC*H* rot. B), 7.24-7.19 (3.17 H, m, 3 x ArC*H* rot. A + 4 x ArC*H* rot. B), 7.15 (1.83 H, br. s, 2 x ArC*H* rot. A + ArC*H* rot. B), 6.95 (2 H, br. s, 2 x ArC*H* rot. A + 2 x ArC*H* rot. B), 6.68 (0.83 H, br. s, ArC*H* rot. A), 4.12 (2 H, s, CH_2 rot. A + CH_2 rot. B), 3.45 (2.49 H, s, NCH_3 rot. A), 2.77 (0.51 H, s, NCH_3 rot. B). **^{13}C NMR (101 MHz, $CDCl_3$)** δ 170.7 (*C*O), 147.9 (C_{quat}), 147.5 (Ar*C*H), 140.2 (C_{quat}), 139.2 (C_{quat}), 135.0 (C_{quat}), 133.8 (Ar*C*H), 130.8 (Ar*C*H), 129.6 (2 x Ar*C*H + Ar*C*H), 129.5 (Ar*C*H), 128.6 (2 x Ar*C*H), 128.4 (Ar*C*H), 126.5 (Ar*C*H), 125.8 (Ar*C*H), 123.5 (Ar*C*H), 39.2 (*C*H_2), 37.5 (N*C*H_3). **HRMS (ESI$^+$)** *m/z* calcd for $C_{20}H_{19}N_2O^+$ $[M+H]^+$ 303.1492; found 303.1489.

2-Benzyl-*N*-methyl-*N*-(pyridin-2-yl)benzamide (3l)

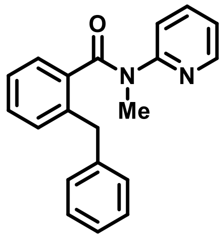

By following GP1, using 2-benzylbenzoic acid (1.06 g, 5.0 mmol), the acyl chloride intermediate was stirred with *N*-methylpyridin-2-amine (0.617 mL, 0.649 g, 6.0 mmol) for 20 hours (0-30% acetone in 40-60 petroleum ether), afforded the title compound (1.07 g, 71%) as a colourless oil.

IR (film, CH_2Cl_2, cm^{-1}) ν_{max} = 2920, 1648, 1352. **^{1}H NMR (400 MHz, $CDCl_3$)** δ 8.39 (1 H, ddd, *J* 4.9, 2.0, 0.8, ArC*H*), 7.33 (1 H, br. s, ArC*H*), 7.28-7.24 (2 H, m, 2 x ArC*H*), 7.23-7.14 (5 H, m, 5 x ArC*H*), 7.05 (2 H, br. s, 2 x ArC*H*), 6.98 (1 H, dd, *J* 7.3, 4.9, ArC*H*), 6.48 (1 H, br. s, ArC*H*), 4.12 (2 H, s, CH_2), 3.39 (3 H, s, CH_3). **^{13}C NMR (101 MHz, $CDCl_3$)** δ 171.1 (*C*O), 155.7 (C_{quat}), 148.4 (Ar*C*H), 140.2 (C_{quat}), 139.1 (C_{quat}), 137.2 (Ar*C*H), 136.4 (C_{quat}), 130.5 (Ar*C*H), 129.6 (2 x Ar*C*H), 129.5 (Ar*C*H), 128.5 (2 x Ar*C*H), 127.7 (Ar*C*H), 126.4 (Ar*C*H), 126.0 (Ar*C*H), 121.0 (Ar*C*H + ArC*H*), 39.0 (*C*H_2), 35.6 (N*C*H_3). **HRMS (ESI$^+$)** *m/z* calcd for $C_{20}H_{18}N_2ONa^+$ $[M+Na]^+$ 325.1311; found 325.1312.

***N*-(3-Chlorophenyl)-2-(4-methoxybenzyl)-*N*-methylbenzamide (5a)**

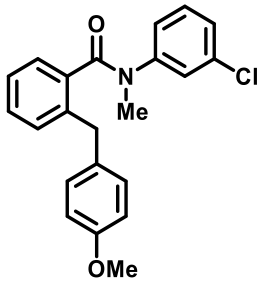

Starting α-aryl-*o*-toluic acid was synthesised from 2-(4-methoxybenzoyl)benzoic acid prepared by following literature procedure[1] then reduced by following literature procedure.[2]

By following GP1, using 2-(4-methoxybenzyl)benzoic acid (1.21 g, 5.0 mmol), the acyl chloride intermediate was stirred with 3-chloro-*N*-methylaniline (0.73 mL, 850 mg, 6.0 mmol) for 20 hours and purified by flash chromatography (SiO_2, 0-30% diethyl ether in 40-60 petroleum ether), affording the title compound (1.73 g, 95%) as a colourless oil.

IR (film, CH_2Cl_2, cm^{-1}) ν_{max} = 2933, 1646, 1590, 1517, 1356, 1243. **^{1}H NMR (400 MHz, $CDCl_3$)** δ 7.38-6.86 (9 H, m, 2 x ArC*H* + 7 x ArC*H*), 6.83 (2 H, d, *J* 8.8, 2 x ArC*H*), 6.50 (1 H, br. s, ArC*H*), 4.05 (2 H, s, CH_2), 3.77 (3 H, s, OCH_3), 3.61-2.67 (3 H, m, CH_3). **^{13}C NMR (101 MHz, $CDCl_3$)** δ 170.6 (*C*O), 158.1 (Ar*C*O), 145.1 (C_{quat}), 139.4 (C_{quat}), 135.5 (C_{quat}), 134.3 (Ar*C*Cl), 132.3 (C_{quat}), 130.4 (2 x Ar*C*H), 130.4 (Ar*C*H), 129.7 (Ar*C*H), 129.2 (Ar*C*H), 128.1 (Ar*C*H), 126.8 (Ar*C*H), 126.7 (Ar*C*H), 125.5 (Ar*C*H), 124.8 (Ar*C*H), 114.0 (2 x Ar*C*H), 55.3 (O*C*H_3), 38.2 (*C*H_2), 37.3 (*C*H_3). **HRMS (ESI$^+$)** *m/z* calcd for $C_{22}H_{20}ClNO_2Na^+$ $[M+Na]^+$ 388.1075; found 388.1074.

***N*-(3-Chlorophenyl)-*N*-methyl-2-(4-methylbenzyl)benzamide (5b)**

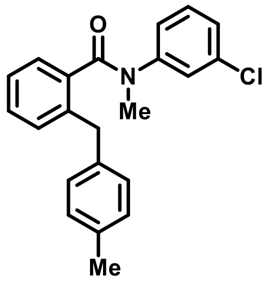

Starting α-aryl-*o*-toluic acid was synthesised from 2-(4-toluoyl)benzoic acid prepared by following literature procedure,[1] then hydrogenated by following literature procedure at 1 bar of hydrogen.[3]

By following GP1, using 2-(4-methylbenzyl)benzoic acid (952 mg, 4.2 mmol), the acyl chloride intermediate was stirred with 3-chloro-*N*-methylaniline (0.62 mL, 714 mg, 5.0 mmol) for 23 hours and purified by flash chromatography (SiO_2, 0-30% diethyl ether in 40-60 petroleum ether), affording the title compound (732 mg, 50%) as a light-brown oil.

IR (film, CH_2Cl_2, cm^{-1}) ν_{max} = 2919, 1644, 1590, 1355, 763. **^{1}H NMR (400 MHz, $CDCl_3$)** δ 7.25-6.86 (10 H, m, 2 x ArC*H* + 2 x ArC*H* + 6 x ArC*H*), 6.51 (2 H, br. s, ArC*H* + ArC*H*), 4.08 (2 H, s, C*H*$_2$), 3.40 (3 H, br. s, NC*H*$_3$), 2.32 (3 H, s, ArCC*H*$_3$). **^{13}C NMR (126 MHz, $CDCl_3$)** δ 170.7 (*C*O), 145.2 (C_{quat}), 139.4 (C_{quat}), 137.2 (C_{quat}), 136.0 (C_{quat}), 135.6 (C_{quat}), 134.4 (Ar*C*Cl), 130.6 (Ar*C*H), 129.8 (Ar*C*H), 129.4 (2 x Ar*C*H), 129.4 (2 x Ar*C*H), 129.3 (Ar*C*H), 128.2 (Ar*C*H), 126.9 (Ar*C*H), 126.9 (Ar*C*H), 125.7 (Ar*C*H), 125.0 (Ar*C*H), 38.8 (*C*H$_2$), 37.5 (N*C*H$_3$), 21.2 (ArC*C*H$_3$). **HRMS (ESI$^+$)** *m/z* calcd for $C_{22}H_{20}NOClNa^+$ $[M+Na]^+$ 372.1126; found 372.1136.

2-([1,1'-Biphenyl]-4-ylmethyl)-*N*-(3-chlorophenyl)-*N*-methylbenzamide (5c)

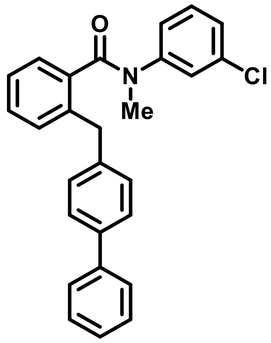

Starting α-aryl-*o*-toluic acid was synthesised from 2-(4-phenylbenzoyl)benzoic acid prepared by following literature procedure,[4] then hydrogenated by following literature procedure at 1 bar of hydrogen.[3]

By following GP1, using 2-([1,1'-biphenyl]-4-ylmethyl)benzoic acid (721 mg, 2.5 mmol), the acyl chloride intermediate was stirred with 3-chloro-*N*-methylaniline (0.37 mL, 425 mg, 3.0 mmol) for 20 hours and purified by flash chromatography (SiO_2, 0-30% diethyl ether in 40-60 petroleum ether), affording the title compound (369 mg, 36%) as a light-brown oil.

IR (film, CH_2Cl_2, cm^{-1}) ν_{max} = 3027, 1647, 1590, 1487, 1359, 758. **^{1}H NMR (400 MHz, $CDCl_3$)** δ 7.58-7.54 (2 H, m, 2 x ArC*H*), 7.52 (2 H, d, *J* 8.2, 2 x ArC*H*), 7.39 (2 H, t, *J* 7.5, 2 x ArC*H*), 7.33-7.26 (3 H, m, ArC*H* + 2 x ArC*H*), 7.18 (3 H, br. s, ArC*H* + ArC*H* + ArC*H*), 7.01 (3 H, br. s, ArC*H* + ArC*H* + ArC*H*), 6.54 (2 H, br. s, ArC*H* + ArC*H*), 4.16 (2 H, s, CH_2), 3.38 (3 H, br. s, CH_3). **^{13}C NMR (126 MHz, $CDCl_3$)** δ 170.5 (*C*O), 145.0 (C_{quat}), 140.9 (C_{quat}), 139.3 (C_{quat}), 139.3 (C_{quat}), 139.0 (C_{quat}), 135.6 (C_{quat}), 134.3 (Ar*C*Cl), 130.6 (Ar*C*H), 129.9 (2 x Ar*C*H), 129.8 (Ar*C*H), 129.3 (Ar*C*H), 128.8 (2 x Ar*C*H), 128.3 (Ar*C*H), 127.3 (2 x Ar*C*H), 127.2 (Ar*C*H), 127.0 (2 x Ar*C*H), 126.8 (Ar*C*H), 126.8 (Ar*C*H), 125.7 (Ar*C*H), 124.8 (Ar*C*H), 38.8 (*C*H_2), 37.5 (*C*H_3). **HRMS (ESI$^+$)** *m/z* calcd for $C_{27}H_{23}NOCl^+$ $[M+H]^+$ 412.1463; found 412.1463.

***N*-(3-Chlorophenyl)-2-(4-fluorobenzyl)-*N*-methylbenzamide (5d)**

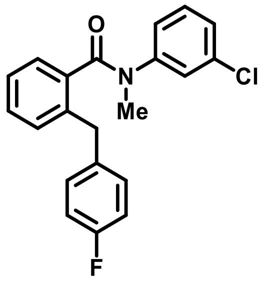

Starting α-aryl-*o*-toluic acid was synthesised from 2-(4-fluorobenzoyl)benzoic acid prepared by following literature procedure,[5] then hydrogenated by following literature procedure at 1 bar of hydrogen.[3]

By following GP1, using 2-(4-fluorobenzyl)benzoic acid (576 mg, 2.5 mmol), the acyl chloride intermediate was stirred with 3-chloro-*N*-methylaniline (0.37 mL, 425 mg, 3.0 mmol) for 24 hours and purified by flash chromatography (SiO_2, 0-30% diethyl ether in 40-60 petroleum ether), affording the title compound (566 mg, 64%) as a colourless oil.

IR (film, CH_2Cl_2, cm^{-1}) ν_{max} =3065, 2921, 1644, 1506, 1356, 1220, 773. **^{1}H NMR (400 MHz, $CDCl_3$)** δ 7.33-6.90 (10 H, m, 2 x ArC*H* + 2 x ArC*H* + 6 x ArC*H*), 6.59 (2 H, br. s, ArC*H* + ArC*H*), 4.08 (2 H, s, CH_2), 3.39 (3 H, br. s, CH_3). **^{13}C NMR (126 MHz, $CDCl_3$)** δ 170.4 (*C*O), 161.5 (Ar*C*F, d, J_{CF} 244.7), 145.0 (C_{quat}), 138.8 (C_{quat}), 135.9 (C_{quat}, d, J_{CF} 3.2), 135.5 (C_{quat}), 134.4 (Ar*C*Cl), 130.8 (2 x Ar*C*H, d, J_{CF} 7.8), 130.4 (Ar*C*H), 129.8 (Ar*C*H), 129.3 (Ar*C*H), 128.2 (Ar*C*H), 126.8 (Ar*C*H), 126.7 (Ar*C*H), 125.8 (Ar*C*H), 124.7 (Ar*C*H), 115.3 (2 x Ar*C*H, d, J_{CF} 21.1), 38.2 (*C*H_2), 37.4 (*C*H_3). **^{19}F NMR (377 MHz, $CDCl_3$)** δ –116.6 (ArC*F*, tt, *J* 8.7, 5.4). **HRMS (ESI$^+$)** *m/z* calcd for $C_{21}H_{17}ClFNONa^+$ $[M+Na]^+$ 376.0875; found 376.0876.

N-(3-Chlorophenyl)-N-methyl-2-(2-methylbenzyl)benzamide (5e)

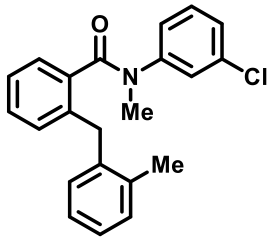

Starting α-aryl-*o*-toluic acid was synthesised from 2-(2-methylbenzoyl)benzoic acid by following literature procedure,[3] reduced to the benzylic alcohol by literature procedure,[6] then hydrogenated by following literature procedure at 1 bar of hydrogen.[3]

By following GP1, using 2-(2-methylbenzyl)benzoic acid (784 mg, 3.5 mmol), the acyl chloride intermediate was stirred with 3-chloro-*N*-methylaniline (0.51 mL, 590 mg, 4.2 mmol) for 24 hours and purified by flash chromatography (SiO_2, 12-100% diethyl ether in 40-60 petroleum ether), affording the title compound (1.05 g, 87%) as an red gum.

IR (film, $CDCl_3$, cm^{-1}) ν_{max} = 3064, 3020, 2925, 1646, 1590, 1477, 1358, 779, 744, 734, 694. **^{1}H NMR (400 MHz, $CDCl_3$)** δ 7.33-6.85 (10 H, m, 10 x ArC*H*), 6.85-6.00 (2 H, m, ArC*H* + ArC*H*), 4.10 (2 H, s, CH_2), 3.68-2.67 (3 H, m, NCH_3), 2.23 (3 H, s, ArCCH_3). **^{13}C NMR (126 MHz, $CDCl_3$)** δ 170.7 (*C*O), 145.1 (C_{quat}), 137.9 (C_{quat}), 137.8 (C_{quat}), 137.2 (C_{quat}), 136.0 (C_{quat}), 134.5 (Ar*C*Cl), 130.8 (Ar*C*H), 130.6 (Ar*C*H), 130.2 (Ar*C*H), 129.9 (Ar*C*H), 129.1 (Ar*C*H), 128.1 (Ar*C*H), 126.9 (Ar*C*H), 126.8 (Ar*C*H + Ar*C*H), 126.1 (Ar*C*H), 125.7 (Ar*C*H), 124.8 (Ar*C*H), 37.5 (NCH_3), 36.9 (CH_2), 20.0 (ArCCH_3). **HRMS (ESI$^+$)** *m/z* calcd for $C_{22}H_{20}ClNONa^+$ $[M+Na]^+$ 372.1126; found 372.1125.

_N_-(3-Chlorophenyl)-_N_-methyl-2-(naphthalen-1-ylmethyl)benzamide (5f)

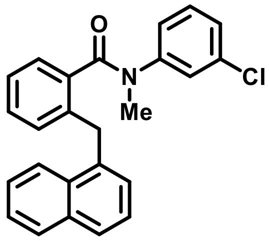

Starting α-aryl-*o*-toluic acid was synthesised from 2-(naphthalen-1-ylmethyl)benzonitrile prepared by following literature procedure,[7] then hydrolysed by following literature procedure.[8]

By following GP1, using 2-(naphthalen-1-ylmethyl)benzoic acid (680 mg, 2.6 mmol), the acyl chloride intermediate was stirred with 3-chloro-*N*-methylaniline (0.38 mL, 439 mg, 3.1 mmol) for 21 hours and purified by flash chromatography (SiO_2, 7-60% diethyl ether in 40-60 petroleum ether), affording the title compound (936 mg, 94%) as an orange gum.

IR (film, $CDCl_3$, cm^{-1}) ν_{max} = 3063, 2924, 1647, 1591, 1478, 1360, 790, 776, 733, 695. **^{1}H NMR (400 MHz, $CDCl_3$)** δ 7.94 (1 H, d, *J* 7.5, ArC*H*), 7.89-7.83 (1 H, m, ArC*H*), 7.77 (1 H, d, *J* 8.2, ArC*H*), 7.51-7.33 (3 H, m, 3 x ArC*H*), 7.31 (1 H, dd, *J* 7.1, 1.2, ArC*H*), 7.28-7.17 (1 H, m, ArC*H*), 7.15-6.84 (6 H, m, 6 x ArC*H*), 6.77-6.54 (1 H, m, ArC*H*), 4.54 (2 H, s, CH_2), 3.77-2.91 (3 H, m, NCH_3). **^{13}C NMR (126 MHz, $CDCl_3$)** δ 170.9 (*C*O), 144.9 (C_{quat}), 138.0 (C_{quat}), 135.9 (C_{quat}), 135.5 (C_{quat}), 134.5 (Ar*C*Cl), 134.0 (C_{quat}), 132.4 (C_{quat}), 130.2 (Ar*C*H), 129.9 (Ar*C*H), 129.3 (Ar*C*H), 128.8 (Ar*C*H), 128.3 (Ar*C*H), 128.0 (Ar*C*H), 127.5 (Ar*C*H), 126.9 (Ar*C*H), 126.8 (Ar*C*H), 126.3 (Ar*C*H), 125.8 (Ar*C*H + Ar*C*H), 125.6 (Ar*C*H), 124.9 (Ar*C*H), 124.5 (Ar*C*H), 36.2 (*C*H_2), 32.0 (N*C*H_3). **HRMS (ESI$^+$)** *m/z* calcd for $C_{25}H_{20}ClNONa^+$ $[M+Na]^+$ 408.1126; found 408.1131.

***N*-(3-Chlorophenyl)-2-(3,4-dimethylbenzyl)-*N*-methylbenzamide (5g)**

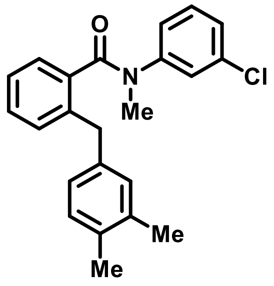

Starting α-aryl-*o*-toluic acid was synthesised by hydrogenation of 2-(3,4-dimethylbenzoyl)benzoic acid following literature procedure at 1 bar of hydrogen.[3]

By following GP1, using 2-(3,4-dimethylbenzyl)benzoic acid (1.12 g, 4.7 mmol), the acyl chloride intermediate was stirred with 3-chloro-*N*-methylaniline (0.50 mL, 578 mg, 4.1 mmol) for 3.5 hours and purified by flash chromatography (SiO_2, 12-100% diethyl ether in 40-60 petroleum ether), affording the title compound (1.03 g, 69%) as an orange gum.

IR (film, $CDCl_3$, cm^{-1}) ν_{max} = 3063, 2921, 1645, 1590, 1476, 1357, 771, 729, 694. **^{1}H NMR (400 MHz, $CDCl_3$)** δ 7.30-6.87 (9 H, m, 9 x ArC*H*), 6.83-6.20 (2 H, m, ArC*H* + ArC*H*), 4.07 (2 H, s, CH_2), 3.77-2.55 (3 H, m, NCH_3), 2.23 (6 H, s, ArCCH_3 + ArCCH_3). **^{13}C NMR (126 MHz, $CDCl_3$)** δ 170.6 (*C*O), 145.2 (C_{quat}), 139.6 (C_{quat}), 137.6 (C_{quat}), 136.7 (C_{quat}), 135.5 (C_{quat}), 134.6 (C_{quat}), 134.3 (Ar*C*Cl), 130.8 (Ar*C*H), 130.5 (Ar*C*H), 129.8 (Ar*C*H), 129.8 (Ar*C*H), 129.2 (Ar*C*H), 128.1 (Ar*C*H), 126.9 (Ar*C*H + Ar*C*H), 126.8 (Ar*C*H), 125.5 (Ar*C*H), 125.0 (Ar*C*H), 38.7 (*C*H_2), 37.5 (N*C*H_3), 19.8 (ArC*C*H_3), 19.4 (ArC*C*H_3). **HRMS (ESI$^+$)** *m/z* calcd for $C_{23}H_{22}ClNONa^+$ $[M+Na]^+$ 386.1282; found 386.1293.

***N*-(3-Chlorophenyl)-2-(3-fluoro-4-methylbenzyl)-*N*-methylbenzamide (5h)**

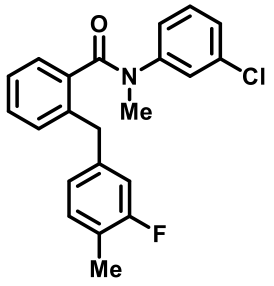

By following GP1, using 2-(3-fluoro-4-methylbenzyl)benzoic acid (989 mg, 4.0 mmol), the acyl chloride intermediate was stirred with 3-chloro-*N*-methylaniline (0.60 mL, 694 mg, 4.9 mmol) for 3.5 hours and purified by flash chromatography (SiO_2, 7-60% diethyl ether in 40-60 petroleum ether), affording the title compound (1.04 g, 70%) as a red oil.

IR (film, $CDCl_3$, cm^{-1}) ν_{max} = 3066, 2925, 1644, 1590, 1501, 1357, 1119, 908, 773, 729, 694. **^{1}H NMR (400 MHz, $CDCl_3$)** δ 7.42-6.82 (9 H, m, 9 x ArC*H*), 6.82-6.22 (2 H, m, 2 x ArC*H*), 4.05 (2 H, s, C*H*$_2$), 3.40 (3 H, m, NC*H*$_3$), 2.23 (3 H, d, *J* 2.1, ArCC*H*$_3$). **^{13}C NMR (126 MHz, $CDCl_3$)** δ 170.6 (*C*O), 160.2 (Ar*C*F, d, J_{CF} 243.5), 145.1 (C_{quat}), 139.1 (C_{quat}), 135.6 (C_{quat}, d, J_{CF} 3.7), 135.5 (C_{quat}), 134.4 (Ar*C*Cl), 132.5 (Ar*C*H, d, J_{CF} 5.0), 130.5 (Ar*C*H), 129.9 (Ar*C*H), 129.4 (Ar*C*H), 128.2 (Ar*C*H), 128.2 (Ar*C*H), 126.9 (Ar*C*H), 126.8 (Ar*C*H), 125.8 (Ar*C*H), 124.9 (Ar*C*CH$_3$, d, J_{CF} 17.2), 124.8 (Ar*C*H), 115.0 (Ar*C*H, d, J_{CF} 22.2), 38.3 (*C*H$_2$), 37.5 (N*C*H$_3$), 14.6 (ArC*C*H$_3$, d, J_{CF} 3.5). **^{19}F NMR (377 MHz, $CDCl_3$)** δ -121.1 (br. s, ArC*F*). **HRMS (ESI$^+$)** *m/z* calcd for $C_{22}H_{19}ClFNONa^+$ $[M+H]^+$ 390.1031; found 390.1039.

***N*-(3-Chlorophenyl)-2-(dibenzo[*b*,*d*]thiophen-2-ylmethyl)-*N*-methylbenzamide (5i)**

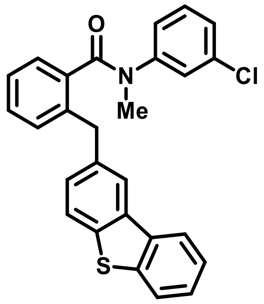

Starting α-aryl-*o*-toluic acid was synthesised from 2-(*o*-carboxybenzoyl)dibenzothiophene prepared by following literature procedure[9] then reduced by following literature procedure.[10]

By following GP1, using 2-(dibenzo[*b*,*d*]thiophen-2-ylmethyl)benzoic acid (451 mg, 1.4 mmol), the acyl chloride intermediate was stirred with 3-chloro-*N*-methylaniline (0.20 mL, 241 mg, 1.7 mmol) for 23 hours and purified by flash chromatography (SiO_2, 0-30% diethyl ether in 40-60 petroleum ether), affording the title compound (264 mg, 43%) as a pale-yellow solid.

M.p. 59-62 °C (Et_2O). **IR (film, CH_2Cl_2, cm^{-1})** ν_{max} = 3066, 2926, 1646, 1591, 1360, 764. **^{1}H NMR (400 MHz, $CDCl_3$)** δ 8.16-8.06 (1 H, m, ArC*H*), 8.03 (1 H, s, ArC*H*), 7.85-7.81 (1 H, m, ArC*H*), 7.79 (1 H, d, *J* 8.2, ArC*H*), 7.46-7.41 (2 H, m, 2 x ArC*H*), 7.32 (1 H, dd, *J* 8.2, 6.9, ArC*H*), 7.22 (2 H, br. s, 2 x ArC*H*), 6.98 (4 H, br. s, 4 x ArC*H*), 6.45 (2 H, br. s, 2 x ArC*H*), 4.32 (2 H, s, C*H*$_2$), 3.53-2.56 (3 H, m, C*H*$_3$). **^{13}C NMR (126 MHz, $CDCl_3$)** δ 170.6 (*C*O), 139.9 (C_{quat}), 139.2 (C_{quat}), 137.6 (C_{quat}), 136.6 (C_{quat}), 135.9 (C_{quat}), 135.6 (C_{quat}), 135.4 (C_{quat}), 134.4 (C_{quat}), 134.4 (Ar*C*Cl), 130.6 (Ar*C*H), 129.8 (Ar*C*H), 129.4 (Ar*C*H), 128.6 (Ar*C*H), 128.4 (Ar*C*H), 126.9 (Ar*C*H), 126.9 (Ar*C*H), 126.8 (Ar*C*H), 125.8 (Ar*C*H), 124.9 (Ar*C*H), 124.4, (Ar*C*H), 123.0 (Ar*C*H), 122.9 (Ar*C*H), 122.5 (Ar*C*H), 121.8 (Ar*C*H), 39.2 (*C*H$_2$), 37.6 (*C*H$_3$). **HRMS (ESI$^+$)** *m/z* calcd for $C_{27}H_{20}ClNOSNa^+$ $[M+Na]^+$ 464.0846; found 464.0843.

2-Benzyl-6-methoxy-*N*-methyl-*N*-(quinolin-8-yl)benzamide (5j)

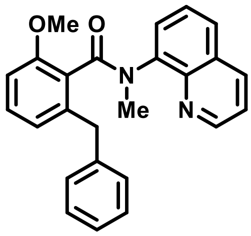

Starting 2-benzyl-6-methoxy-*N*-(quinolin-8-yl)benzamide was synthesised by following literature procedure.[11]

Sodium hydride (63 mg, 60% in mineral oil, 1.6 mmol) was suspended in anhydrous DMF (3.8 mL), and cooled to 0 °C. A solution of 2-benzyl-6-methoxy-*N*-(quinolin-8-yl)benzamide (283 mg, 0.8 mmol) in anhydrous DMF (3.8 mL) was added dropwise. The reaction mixture was stirred at room temperature for 1 hour, then methyl iodide (0.06 mL, 141 mg, 1.0 mmol) was added dropwise, and the reaction mixture was stirred at room temperature for a further 1 hour. The reaction mixture was diluted with DCM (25 mL), washed with H_2O (3 × 25 mL), dried (Na_2SO_4) and concentrated *in vacuo*. The crude residue was purified by flash chromatography (SiO_2, 7-60% ethyl acetate in 40-60 petroleum ether), affording the title compound (237 mg, 81%) as a yellow gum.

IR (film, $CDCl_3$, cm^{-1}) ν_{max} = 3060, 3028, 2933, 1642, 1596, 1581, 1496, 1469, 1390, 1261, 1067, 732. **^{1}H NMR (500 MHz, $CDCl_3$, mixture of rotamers A:B:C in a 0.55:0.25:0.20 ratio)** δ 9.08 (0.2 H, d, *J* 3.1, ArC*H* rot. C), 8.89 (0.25 H, br. s, ArC*H* rot. B), 8.77 (0.55 H, dd, *J* 4.3, 1.7, ArC*H* rot. A), 8.21 (0.25 H, d, *J* 8.2, ArC*H* rot. B), 8.13-8.04 (0.75 H, m, ArC*H* rot. A + ArC*H* rot. C), 7.84 (0.45 H, d, *J* 8.1, ArC*H* rot. B + ArC*H* rot. C), 7.76 (0.55 H, dd, *J* 7.3, 1.4, ArC*H* rot. A), 7.66 (0.55 H, dd, *J* 8.1, 1.5, ArC*H* rot. A), 7.63 (0.2 H, d, *J* 7.7, ArC*H* rot. C), 7.52 (0.2 H, d, *J* 8.2, ArC*H* rot. C), 7.49-7.39 (1.45 H, m, 3 x ArC*H* rot. B + 2 x ArC*H* rot. C), 7.38-7.27 (2.3 H, m, 2 x ArC*H* rot. A + 3 x ArC*H* rot. B + 2 x ArC*H* rot. C), 7.27-7.24 (0.2 H, m, ArCH rot. C), 7.24-7.18 (0.5 H, m, 2 x ArCH rot. B), 7.08-7.04 (1.65 H, m, 3 x ArCH rot. A), 6.98 (0.25 H, t, *J* 8.0, ArC*H* rot. B), 6.91 (0.2 H, t, *J* 7.8, ArC*H* rot. C), 6.87-6.81 (1 H, m, ArC*H* rot. A + ArC*H* rot. B + ArC*H* rot. C), 6.77 (0.2 H, d, *J* 7.6, ArC*H* rot. C), 6.62-6.57 (1.1 H, m, 2 x ArC*H* rot. A), 6.55 (0.55 H, d, *J* 8.3, ArC*H* rot. A), 6.15-6.08 (1 H, m, ArC*H* rot. A + ArC*H* rot. B + ArC*H* rot. C), 4.44 (0.45 H, d, *J* 14.9, CH_aH_b rot. B + CH_aH_b rot. C), 4.31 (0.25 H, d, *J* 15.4, CH_aH_b rot. B), 4.11 (0.2 H, d, *J* 14.5, CH_aH_b rot. C), 4.05 (0.55 H, d, *J* 16.2, CH_aH_b rot. A), 3.96 (0.75 H, s, OCH_3 rot. B), 3.94 (1.65 H, s, OCH_3 rot. A), 3.80 (0.55 H, d, *J* 16.2, CH_aH_b rot. A), 3.71 (0.6 H, s, NCH_3 rot. C), 3.70 (1.65 H, s, NCH_3 rot. A), 3.31 (0.75 H, s, NCH_3 rot. B), 2.61 (0.60, s, OCH_3 rot. C). **^{13}C NMR (101 MHz, $CDCl_3$, mixture of rotamers A:B:C in a 0.55:0.25:0.20 ratio)** δ 169.9 (*C*O rot. B), 169.1 (*C*O rot. A), 168.5 (*C*O rot. C), 155.7 (Ar*C*O rot. B), 155.5 (Ar*C*O rot. A), 155.1 (Ar*C*O rot. C), 150.3 (Ar*C*H rot. A + Ar*C*H rot. B), 149.7 (Ar*C*H rot. C), 144.4 (C_{quat}

rot. B), 144.1 (C_{quat} rot. A + C_{quat} rot. C), 141.0 (C_{quat} rot. C), 140.7 (C_{quat} rot. A + C_{quat} rot. B), 140.5 (C_{quat} rot. A), 140.4 (C_{quat} rot. C), 140.3 (C_{quat} rot. B), 140.3 (C_{quat} rot. C), 140.0 (C_{quat} rot. B), 139.5 (C_{quat} rot. A), 136.4 (Ar*C*H rot. C), 136.2 (Ar*C*H rot. B), 136.0 (Ar*C*H rot. A), 129.7 (2 x Ar*C*H rot. C), 129.7 (2 x Ar*C*H rot. B), 129.6 (Ar*C*H rot. C), 129.6 (Ar*C*H rot. B), 129.4 (Ar*C*H rot. B), 129.3 (Ar*C*H rot. C), 129.2 (2 x Ar*C*H rot. A + C_{quat} rot. A), 128.9 (Ar*C*H rot. A), 128.8 (C_{quat} rot. B), 128.5 (2 x Ar*C*H rot. C), 128.4 (2 x Ar*C*H rot. B), 128.1 (Ar*C*H rot. A), 128.0 (2 x Ar*C*H rot. A + Ar*C*H rot. B), 127.8 (Ar*C*H rot. B), 127.8 (Ar*C*H rot. A), 127.2 (C_{quat} rot. B), 127.2 (Ar*C*H rot. C), 126.6 (C_{quat} rot. A), 126.4 (Ar*C*H rot. C), 126.3 (C_{quat} rot. C), 126.0 (Ar*C*H rot. B), 125.9 (Ar*C*H rot. A), 125.6 (Ar*C*H rot. A), 125.5 (C_{quat} rot. C), 125.2 (Ar*C*H rot. C), 122.4 (Ar*C*H rot. B), 122.2 (Ar*C*H rot. C), 121.6 (Ar*C*H rot. A), 121.5 (Ar*C*H rot. A), 121.5 (Ar*C*H rot. B), 121.0 (Ar*C*H rot. C), 108.4 (Ar*C*H rot. C), 107.7 (Ar*C*H rot. A), 107.7 (Ar*C*H rot. B), 56.0 (OCH_3 rot. B), 55.6 (OCH_3 rot. A), 53.8 (OCH_3 rot. C), 39.7 (NCH_3 rot. B), 39.4 (CH_2 rot. C), 38.4 (CH_2 rot. B), 38.1 (CH_2 rot. A), 37.4 (NCH_3 rot. C), 37.3 (NCH_3 rot. A). **HRMS (ESI$^+$)** *m/z* calcd for $C_{25}H_{22}N_2O_2Na^+$ $[M+Na]^+$ 405.1573; found 405.1581.

(2-Benzylphenyl)(3,4-dihydroquinolin-1(2*H*)-yl)methanone (7a)

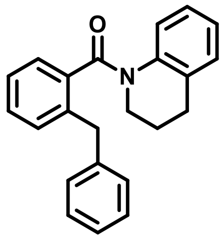

By following an adaptation of GP1, using 2-benzylbenzoic acid (1.06 g, 5.0 mmol), the acyl chloride intermediate was stirred with 1,2,3,4-tetrahydroquinoline (0.75 mL, 799 mg, 6.0 mmol) in anhydrous toluene (20 mL, 0.25 M) at 80 °C for 20 hours. After which, the toluene was removed under reduced pressure, the work-up was performed according to GP1 and the resulting crude material was purified by flash chromatography (SiO_2, 0-50% ethyl acetate in 40-60 petroleum ether), affording the title compound (1.19 g, 73%) as a yellow oil that solidified upon standing.

M.p. 76-77 °C (CH_2Cl_2). **IR (neat, cm^{-1}) ν_{max}** = 2926, 1741, 1374, 1241, 1057. **^{1}H NMR (400 MHz, $CDCl_3$)** δ 8.59-7.22 (4 H, m, 4 × ArC*H*), 7.22-7.09 (6 H, m, 6 × ArC*H*), 7.09-6.73 (2 H, m, 2 x ArC*H*), 6.21 (1 H, *br*. s, ArC*H*), 4.08 (2 H, s, C*H*$_2$), 3.75 (2 H, br. s, C*H*$_2$), 2.79 (2 H, br. t, *J* 6.7, C*H*$_2$), 1.94 (2 H, br. s, C*H*$_2$). **^{13}C NMR (126 MHz, DMSO-*d*$_6$, 100 °C)** δ 169.7 (*C*O), 140.5 (C_{quat}), 138.8 (C_{quat}), 138.5 (C_{quat}), 137.4 (C_{quat}), 131.5 (C_{quat}), 130.7 (ArCH), 129.5 (ArCH), 129.4 (2 × Ar*C*H), 129.0 (Ar*C*H), 128.7 (2 × Ar*C*H), 127.5 (Ar*C*H), 126.5 (2 × Ar*C*H), 125.7 (Ar*C*H), 124.7 (Ar*C*H), 124.7 (Ar*C*H), 45.2 (*C*H$_2$), 38.5 (*C*H$_2$), 26.7 (*C*H$_2$), 23.7 (*C*H$_2$). **HRMS (ESI$^+$)** *m/z* calcd for $C_{23}H_{21}NONa^+$ [M+Na]$^+$ 350.1515; found 350.1516.

(2-Benzylphenyl)(3,4-dihydro-1,5-naphthyridin-1(2*H*)-yl)methanone (7b)

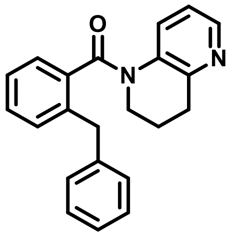

Starting 1,2,3,4-tetrahydro-1,5-naphthyridine was synthesised from 1,5-naphthyridine following a literature procedure.[12]

By following an adaptation of GP1, using 2-benzylbenzoic acid (395 mg, 1.9 mmol), the acyl chloride intermediate was stirred with 1,2,3,4-tetrahydro-1,5-naphthyridine (250 mg, 1.9 mmol) in anhydrous toluene (7.6 mL) at 80 °C for 23 hours. After which the toluene was removed under reduced pressure, the work-up was performed according to GP1 and the resulting crude material was purified by flash chromatography (SiO_2, 12-100% diethyl ether in 40-60 petroleum ether), affording the title compound (380 mg, 62%) as a yellow oil that solidified upon standing.

M.p. 101-103 °C (CH_2Cl_2). **IR (film, $CDCl_3$, cm^{-1})** ν_{max} = 3061, 3026, 2935, 1644, 1581, 1450, 1369, 1344, 908, 726, 698, 640. **^{1}H NMR (400 MHz, $CDCl_3$)** δ 8.24 (1 H, br. s, ArC*H*), 7.32 (1 H, t, *J* 7.4, ArC*H*), 7.28-7.06 (9 H, m, 9 x ArC*H*), 7.05-6.80 (1 H, m, ArC*H*), 4.07 (2 H, br. s, CH_2), 3.87-3.12 (2 H, m, CH_2), 2.92 (2 H, br. s, CH_2), 1.88 (1 H, br. s, CH_aH_b), 1.66 (1 H, br. s. CH_aH_b). **^{13}C NMR (126 MHz, $CDCl_3$)** δ 170.4 (*C*O), 149.7 (C_{quat}), 145.1 (Ar*C*H), 139.8 (C_{quat}), 138.6 (C_{quat}), 136.1 (C_{quat}), 134.7 (C_{quat}), 131.5 (Ar*C*H), 130.9 (Ar*C*H), 129.6 (Ar*C*H), 129.2 (2 x Ar*C*H), 128.5 (2 x Ar*C*H), 126.9 (Ar*C*H), 126.4 (Ar*C*H), 126.3 (Ar*C*H), 120.8 (Ar*C*H), 38.9 (CH_2), 38.9 (CH_2), 30.1 (CH_2), 22.7 (CH_2). **HRMS (ESI$^+$)** *m/z* calcd for $C_{22}H_{20}N_2ONa^+$ $[M+Na]^+$ 351.1468; found 351.1462.

(2-Benzylphenyl)(2,3,4,5-tetrahydro-1*H*-benzo[*b*]azepin-1-yl)methanone (7c)

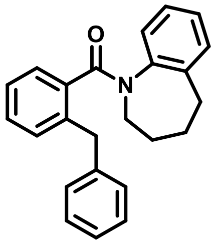

By following an adaptation of GP1, using 2-benzylbenzoic acid (1.06 g, 5.0 mmol), the acyl chloride intermediate was stirred with 2,3,4,5-tetrahydro-1*H*-benzo[*b*]azepine (883 mg, 6.0 mmol) in anhydrous toluene (20 mL) at 80 °C for 4.5 hours. After which the toluene was removed under reduced pressure, the work-up was performed according to GP1 and the resulting crude material was purified by flash chromatography (SiO_2, 7-60% diethyl ether in 40-60 petroleum ether), affording the title compound (1.41 g, 82%) as a white solid.

M.p. 107-109 °C (CH_2Cl_2). **IR (film, $CDCl_3$, cm^{-1})** ν_{max} = 3060, 3025, 2931, 2852, 1638, 1598, 1578, 1492, 1388, 1311, 737, 699. **^{1}H NMR (400 MHz, $CDCl_3$)** δ 7.46-7.17 (5 H, m, 2 x ArC*H* + 2 x ArC*H* + ArC*H*), 7.14-7.06 (3 H, m, ArC*H* + ArC*H* + ArC*H*), 6.97 (1 H, td, *J* 7.6, 1.4, ArC*H*), 6.84 (1 H, td, *J* 7.4, 1.7, ArC*H*), 6.75-6.70 (1 H, m, ArC*H*), 6.70 (1 H, td, *J* 7.6, 1.4, ArC*H*), 5.87 (1 H, d, *J* 7.6, ArC*H*), 4.98 (1 H, d, *J* 12.9, CH_aH$_b$), 4.41 (1 H, d, *J* 14.7, CH_aH$_b$), 4.06 (1 H, d, *J* 14.7, CH$_a$$H_b$), 3.00 (1 H, t, *J* 12.9, CH_aH$_b$), 2.87-2.77 (1 H, m, CH$_a$$H_b$), 2.72 (1 H, t, *J* 12.9, CH$_a$$H_b$), 2.15-1.98 (2 H, m, C$H_aH_b$ + CH_aH$_b$), 1.98-1.89 (1 H, m, CH$_a$$H_b$), 1.46 (1 H, q, *J* 12.9, CH$_a$$H_b$). **^{13}C NMR (101 MHz, $CDCl_3$)** δ 169.3 (*C*O), 143.3 (C_{quat}), 140.6 (C_{quat}), 139.2 (C_{quat} + C_{quat}), 136.3 (C_{quat}), 130.3 (Ar*C*H), 129.9 (Ar*C*H), 129.7 (2 x Ar*C*H), 128.8 (Ar*C*H), 128.5 (2 x Ar*C*H), 127.8 (Ar*C*H), 127.1 (Ar*C*H), 126.8 (Ar*C*H + Ar*C*H), 126.4 (Ar*C*H), 125.3 (Ar*C*H), 47.5 (*C*H$_2$), 39.2 (*C*H$_2$), 35.2 (*C*H$_2$), 29.5 (*C*H$_2$), 26.6 (*C*H$_2$). **HRMS (ESI$^+$)** *m/z* calcd for $C_{24}H_{23}NONa^+$ [M+Na]$^+$ 364.1672; found 364.1676.

(2-Benzylphenyl)(10,11-dihydro-5*H*-dibenzo[*b*,*f*]azepin-5-yl)methanone (7d)

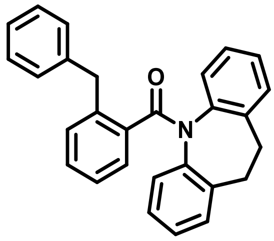

By following an adaptation of GP1, using 2-benzylbenzoic acid (1.06 g, 5.0 mmol), the acyl chloride intermediate was stirred with 10,11-dihydro-5*H*-dibenzo[*b*,*f*]azepine (1.17 g, 6.0 mmol) in anhydrous toluene (20 mL) at 80 °C for 11 days. After which the toluene was removed under reduced pressure, the work-up was performed according to GP1 and the resulting crude material was purified by flash chromatography (SiO_2, 0-25% diethyl ether in 40-60 petroleum ether), affording the title compound (1.11 g, 57%) as a cream solid.

M.p. 170-172 °C (CH_2Cl_2). **IR (film, $CDCl_3$, cm^{-1})** ν_{max} = 3061, 3025, 2922, 2858, 1651, 1601, 1574, 1489, 1339, 738. **^{1}H NMR (400 MHz, $CDCl_3$)** δ 7.48 (1 H, d, *J* 7.8, ArC*H*), 7.42-7.34 (4 H, m, 2 x ArC*H* + 2 x ArC*H*), 7.34-7.23 (4 H, m, 4 x ArC*H*), 7.21-7.13 (2 H, m, ArC*H* + ArC*H*), 7.10-6.89 (4 H, m, 4 x ArC*H*), 6.68 (1 H, t, *J* 7.4, ArC*H*), 6.09 (1 H, d, *J* 7.9, ArC*H*), 4.57 (1 H, d, *J* 15.0, CH_aH_b), 4.19 (1 H, d, *J* 15.0, CH_aH_b), 3.70-3.51 (2 H, m, CH_2), 3.02-2.83 (2 H, m, CH_2). **^{13}C NMR (101 MHz, $CDCl_3$)** δ 169.6 (*C*O), 141.5 (C_{quat}), 141.3 (C_{quat}), 140.5 (C_{quat}), 139.7 (C_{quat}), 136.0 (C_{quat}), 135.7 (C_{quat}), 135.3 (C_{quat}), 130.7 (Ar*C*H), 130.3 (Ar*C*H), 129.9 (2 x Ar*C*H + Ar*C*H), 129.1 (Ar*C*H), 128.6 (2 x Ar*C*H), 128.3 (Ar*C*H), 128.2 (Ar*C*H), 128.0 (Ar*C*H), 127.5 (Ar*C*H + Ar*C*H), 127.1 (Ar*C*H), 126.5 (Ar*C*H + Ar*C*H), 125.4 (Ar*C*H), 39.2 (CH_2), 31.5 (CH_2), 30.4 (CH_2). **HRMS (ESI$^+$)** *m/z* calcd for $C_{28}H_{23}NONa^+$ $[M+Na]^+$ 412.1672; found 412.1677.

5. Synthetic Procedures for Products

2-((4-Methoxyphenyl)(phenyl)methyl)-*N*-methylbenzamide (4a)

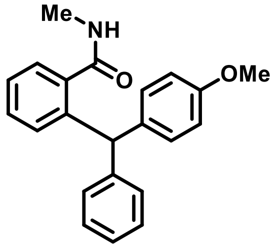

By following GP2, using 2-benzyl-*N*-(4-methoxyphenyl)-*N*-methylbenzamide (**3a**) (66 mg, 0.2 mmol) as the aryl amide, heating the reaction mixture at 125 °C (μwave) for 1 hour, and purifying by flash chromatography (SiO_2, 0-100% diethyl ether in *n*-pentane), afforded the title compound (35 mg, 53%) as a white solid.

M.p. 130-132 °C (CH_2Cl_2). **IR (film, $CDCl_3$, cm^{-1}) ν_{max}** = 3295, 3025, 1635, 1532, 700. **^1H NMR (400 MHz, $CDCl_3$)** δ 7.32-7.07 (6 H, m, 2 × ArC*H* + 4 × ArC*H*), 7.02 (2 H, d, *J* 7.5, 2 × ArC*H*), 6.98-6.87 (3 H, m, 2 × ArC*H* + ArC*H*), 6.74 (2 H, d, *J* 8.2, 2 × ArC*H*), 5.93 (1 H, s, C*H*), 5.16 (1 H, br. s, N*H*), 3.70 (3 H, s, OCH_3), 2.63 (3 H, d, *J* 4.9, CH_3). **^{13}C NMR (101 MHz, $CDCl_3$)** δ 170.8 (*C*O), 158.1 (Ar*C*O), 143.7 (C_{quat}), 141.7 (C_{quat}), 137.5 (C_{quat}), 135.4 (C_{quat}), 130.5 (2 × Ar*C*H), 130.2 (Ar*C*H), 129.5 (Ar*C*H), 129.5 (2 × Ar*C*H), 128.4 (2 × Ar*C*H), 127.2 (Ar*C*H), 126.4 (Ar*C*H), 126.4 (Ar*C*H), 113.8 (2 × Ar*C*H), 55.3 (O*C*H_3), 51.8 (*C*H), 26.4 (*C*H_3). **HRMS (ESI$^+$)** *m/z* calcd for $C_{22}H_{21}NO_2Na^+$ $[M+Na]^+$ 354.1465; found 354.1469.

***N*-Methyl-2-(phenyl(*p*-tolyl)methyl)benzamide (4b)**

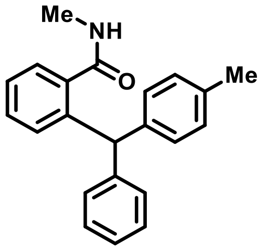

By following GP2, using 2-benzyl-*N*-methyl-*N*-(*p*-tolyl)benzamide (**3b**) (63 mg, 0.2 mmol) as the aryl amide, heating the reaction mixture at 100 °C (μwave) for 1 hour, and purified by flash chromatography (SiO_2, 0-100% diethyl ether in *n*-pentane), afforded the title compound (47 mg, 75%) as an off-white solid.

M.p. 144-145 °C (CH_2Cl_2). **IR (film, $CDCl_3$, cm^{-1}) v_{max} =** 3292, 3024, 1635, 1537, 1512, 700. **^1H NMR (400 MHz, $CDCl_3$)** δ 7.37-7.15 (6 H, m, 6 × ArC*H*), 7.13-7.03 (4 H, m, 4 × ArC*H*), 7.03-6.93 (3 H, m, 2 × ArC*H* + ArC*H*), 5.99 (1 H, s, C*H*), 5.20 (1 H, br. s, N*H*), 2.69 (3 H, d, *J* 5.0, CH_3), 2.30 (3 H, s, ArCCH_3). **^{13}C NMR (101 MHz, $CDCl_3$)** δ 170.9 (*C*O), 143.7 (C_{quat}), 141.6 (C_{quat}), 140.4 (C_{quat}), 137.6 (C_{quat}), 136.1 (C_{quat}), 130.3 (Ar*C*H), 129.6 (Ar*C*H + 2 × Ar*C*H), 129.5 (2 × Ar*C*H), 129.2 (2 × Ar*C*H), 128.4 (2 × Ar*C*H), 127.3 (Ar*C*H), 126.5 (Ar*C*H), 126.4 (Ar*C*H), 52.3 (*C*H), 26.5 (CH_3), 21.1 (ArCCH_3). **HRMS (ESI+)** *m/z* calcd for $C_{22}H_{22}NO^+$ $[M+H]^+$ 316.1696; found 316.1705.

***N*-Methyl-2-(phenyl(*o*-tolyl)methyl)benzamide (4c)**

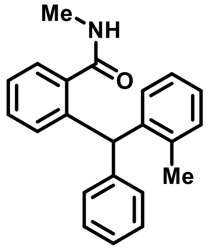

By following GP2, using 2-benzyl-*N*-methyl-*N*-(*o*-tolyl)benzamide (**3c**) (63 mg, 0.2 mmol) as the aryl amide, heating the reaction mixture at 125 °C for 1 hour, and purifying by flash chromatography (SiO_2, 0-100% diethyl ether in *n*-pentane), afforded the title compound (32 mg, 51%) as a white solid.

M.p. 135-139 °C ($CDCl_3$). **IR (film, CH_2Cl_2, cm^{-1})** ν_{max} = 3301, 2925, 1638, 1533. **^{1}H NMR (400 MHz, $CDCl_3$)** δ 7.28 (1 H, d, *J* 7.1, ArC*H*), 7.23-6.98 (10 H, m, 2 x ArC*H* + 2 x ArC*H* + 6 x ArC*H*), 6.82 (1 H, d, *J* 7.6, ArC*H*), 6.70 (1 H, d, *J* 7.5, ArC*H*), 6.07 (1 H, s, C*H*), 5.01 (1 H, br. s, N*H*), 2.55 (3 H, d, *J* 4.9, NCH_3), 2.13 (3 H, s, ArCCH_3). **^{13}C NMR (101 MHz, $CDCl_3$)** δ 170.9 (*C*O), 142.7 (C_{quat}), 142.0 (C_{quat}), 141.2 (C_{quat}), 137.7 (C_{quat}), 137.3 (Ar*C*CH_3), 130.7 (Ar*C*H), 130.1 (Ar*C*H), 129.9 (2 x Ar*C*H), 129.6 (Ar*C*H), 129.2 (Ar*C*H), 128.6 (2 x Ar*C*H), 127.4 (Ar*C*H), 126.7 (Ar*C*H), 126.6 (Ar*C*H + Ar*C*H), 125.9 (Ar*C*H), 49.8 (*C*H), 26.5 (NCH_3), 19.9 (ArCCH_3). **HRMS (ESI$^+$)** *m/z* calcd for $C_{22}H_{22}NO^+$ $[M+H]^+$ 316.1696; found 316.1704.

***N*-Methyl-2-(naphthalen-1-yl(phenyl)methyl)benzamide (4d)**

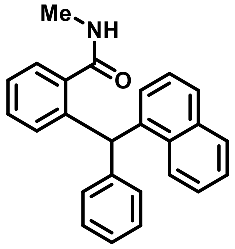

By following GP2, using 2-benzyl-*N*-methyl-*N*-(naphthalen-1-yl)benzamide (**3d**) (70 mg, 0.2 mmol) as the aryl amide, heating the reaction mixture at 100 °C (μwave) for 1 hour, and purifying by flash chromatography (SiO_2, 0-100% diethyl ether in *n*-pentane), afforded the title compound (63 mg, 89%) as a white solid.

M.p. 198-200 °C (CH_2Cl_2). **IR (film, $CDCl_3$, cm^{-1}) ν_{max}** = 3300, 3059, 1634, 1529, 727. **^{1}H NMR (400 MHz, $CDCl_3$)** δ 8.17-8.06 (1 H, m, ArC*H*), 7.91-7.81 (1 H, m, ArC*H*), 7.77 (1 H, d, *J* 8.2, ArC*H*), 7.53-7.20 (9 H, m, 9 × ArC*H*), 7.13 (2 H, d, *J* 7.4, 2 × ArC*H*), 6.98 (1 H, d, *J* 7.2, ArC*H*), 6.94 (1 H, dd, *J* 5.7, 3.4, ArC*H*), 6.81 (1 H, s, C*H*), 5.14 (1 H, br. s, N*H*), 2.55 (3 H, d, *J* 4.9, CH_3). **^{13}C NMR (101 MHz, $CDCl_3$)** δ 170.8 (*C*O), 143.2 (C_{quat}), 141.2 (C_{quat}), 139.6 (C_{quat}), 137.3 (C_{quat}), 134.0 (C_{quat}), 131.9 (C_{quat}), 130.2 (Ar*C*H), 129.8 (2 × Ar*C*H), 129.6 (Ar*C*H), 128.5 (2 × Ar*C*H), 128.5 (Ar*C*H), 127.6 (Ar*C*H), 127.4 (Ar*C*H), 127.3 (Ar*C*H), 126.6 (Ar*C*H), 126.6 (Ar*C*H), 126.6 (Ar*C*H), 125.8 (Ar*C*H), 125.0 (Ar*C*H), 124.7 (Ar*C*H), 49.2 (*C*H), 26.3 (*C*H_3). **HRMS (ESI$^+$)** *m/z* calcd for $C_{25}H_{22}NO^+$ [M+H]$^+$ 352.1696; found 352.1683.

2-((3,5-Difluorophenyl)(phenyl)methyl)-*N*-methylbenzamide (4e)

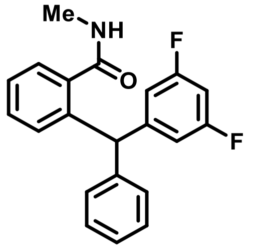

By following GP2, using 2-benzyl-*N*-(3,5-difluorophenyl)-*N*-methylbenzamide (**3e**) (67 mg, 0.2 mmol) as the aryl amide, heating the reaction mixture at 100 °C (μwave) for 1 hour, and purifying by flash chromatography (SiO_2, 0-100% diethyl ether in *n*-pentane), afforded the title compound (41 mg, 61%) as a pale-yellow oil.

IR (film, $CDCl_3$, cm^{-1}) ν_{max} = 3288, 3024, 2936, 1622, 1596, 1116, 989, 700. **^{1}H NMR (400 MHz, $CDCl_3$)** δ 7.39-7.20 (6 H, m, 4 × ArC*H* + 2 × ArC*H*), 7.14-7.06 (2 H, m, 2 × ArC*H*), 7.00 (1 H, d, *J* 7.7, ArC*H*), 6.73-6.61 (3 H, m, ArC*H* + 2 × ArC*H*), 6.17 (1 H, s, C*H*), 5.42 (1 H, br. s, N*H*), 2.74 (3 H, d, *J* 4.9, CH_3). **^{13}C NMR (101 MHz, $CDCl_3$)** δ 170.5 (*C*O), 163.9 (dd, J_{CF} 248.3, 12.9, 2 x Ar*C*F), 147.6 (t, J_{CF} 8.5, C_{quat}), 142.1 (C_{quat}), 140.7 (C_{quat}), 137.3 (C_{quat}), 130.1 (Ar*C*H), 129.9 (Ar*C*H), 129.4 (2 × Ar*C*H), 128.6 (2 × Ar*C*H), 127.1 (Ar*C*H), 126.9 (Ar*C*H), 126.9 (Ar*C*H), 112.5 (dd, J_{CF} 18.5, 6.8, 2 x Ar*C*H), 101.9 (t, J_{CF} 25.4, Ar*C*H), 51.9 (t, J_{CF} 1.9, *C*H), 26.5 (*C*H_3). **^{19}F NMR (376 MHz, $CDCl_3$)** δ -109.9 (t, *J* 8.2, 2 x ArC*F*). **HRMS (ESI$^+$)** *m/z* calcd for $C_{21}H_{18}NOF_2^+$ $[M+H]^+$ 338.1351; found 338.1357.

_N_-Methyl-2-(phenyl(4-(trifluoromethyl)phenyl)methyl)benzamide (4f)

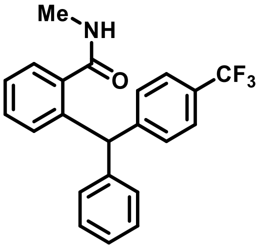

By following an adaptation of GP2, using 2-benzyl-*N*-methyl-*N*-(4-(trifluoromethyl)phenyl)benzamide (**3f**) (74 mg, 0.2 mmol) as the aryl amide, using LiHMDS (0.4 mL, 0.4 mmol, 1 M in THF, 2 eq.) in place of KHMDS, performing the reaction at 20 °C for 16 hours, and purifying by flash chromatography (SiO_2, 0-100% diethyl ether in *n*-pentane), afforded the title compound (51 mg, 69%) as a pale-yellow solid.

M.p. 129-132 °C ($CDCl_3$). **IR (film, CH_2Cl_2, cm^{-1})** ν_{max} = 3297, 1637, 1325, 1122, 1068. **^{1}H NMR (400 MHz, $CDCl_3$)** δ 7.53 (2 H, d, *J* 8.2, 2 x ArC*H*), 7.35 (1 H, dd, *J* 7.2, 1.5, ArC*H*), 7.33-7.27 (3 H, m, 2 x ArC*H* + ArC*H*), 7.27-7.21 (4 H, m, 2 x ArC*H* + ArC*H* + ArC*H*), 7.11-7.07 (2 H, m, 2 x ArC*H*), 6.97 (1 H, d, *J*, 7.9, ArC*H*), 6.22 (1 H, s, C*H*), 5.34 (1 H, br. s, N*H*), 2.70 (3 H, d, *J* 5.0, C*H*3). **^{13}C NMR (101 MHz, $CDCl_3$)** δ 170.7 (*C*O), 147.7 (C_{quat}), 142.5 (C_{quat}), 141.1 (C_{quat}), 137.4 (C_{quat}), 130.3 (Ar*C*H), 130.0 (2 x Ar*C*H), 129.9 (Ar*C*H), 129.6 (2 x Ar*C*H), 128.7 (q, J_{CF} 32.4, C_{quat}), 128.7 (2 x Ar*C*H), 127.2 (Ar*C*H), 126.9 (Ar*C*H + Ar*C*H), 125.3 (q, J_{CF} 3.8, 2 x Ar*C*H), 124.3 (q, J_{CF} 271.8, *C*F3), 52.2 (*C*H), 26.6 (*C*H3). **^{19}F NMR (377 MHz, $CDCl_3$)** δ –62.3 (s, C*F*3). **HRMS (ESI$^+$)** *m/z* calcd for $C_{22}H_{18}F_3NONa^+$ $[M+Na]^+$ 392.1233; found 392.1241.

2-((3-Chlorophenyl)(phenyl)methyl)-*N*-methylbenzamide (4g)

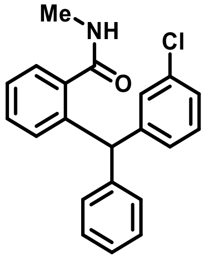

By following GP2, using 2-benzyl-*N*-(3-chlorophenyl)-*N*-methylbenzamide (**3g**) (67 mg, 0.2 mmol) as the aryl amide, heating the reaction mixture at 60 °C for 5 hours, and purifying by flash chromatography (SiO_2, 0-100% diethyl ether in *n*-pentane), afforded the title compound (49 mg, 73%) as a white solid.

M.p. 120-123 °C ($CDCl_3$). **IR (film, CH_2Cl_2, cm^{-1})** ν_{max} = 3295, 2926, 1635, 1539. **^{1}H NMR (400 MHz, $CDCl_3$)** δ 7.35-7.18 (8 H, m, 2 x ArC*H* + 6 x ArC*H*), 7.11-7.07 (3 H, m, 2 x ArC*H* + ArC*H*), 7.04-7.00 (1 H, m, ArC*H*), 6.98 (1 H, d, *J* 8.1, ArC*H*), 6.11 (1 H, s, C*H*), 5.32 (1 H, br. s, N*H*), 2.70 (3 H, d, *J* 4.9, NC*H*$_3$). **^{13}C NMR (101 MHz, $CDCl_3$)** δ 170.7 (*C*O), 145.6 (C_{quat}), 142.6 (C_{quat}), 141.1 (C_{quat}), 137.5 (C_{quat}), 134.3 (Ar*C*Cl), 130.3 (Ar*C*H), 129.8 (Ar*C*H), 129.7 (Ar*C*H), 129.6 (Ar*C*H), 129.6 (2 x Ar*C*H), 128.6 (2 x Ar*C*H), 128.0 (Ar*C*H), 127.2 (Ar*C*H), 126.8 (Ar*C*H + Ar*C*H), 126.8 (Ar*C*H), 52.1 (*C*H), 26.6 (*C*H$_3$). **HRMS (ESI$^+$)** *m/z* calcd for $C_{21}H_{19}NOCl^+$ $[M+H]^+$ 336.1150; found 336.1148.

2-((4-Chlorophenyl)(phenyl)methyl)-*N*-methylbenzamide (4h)

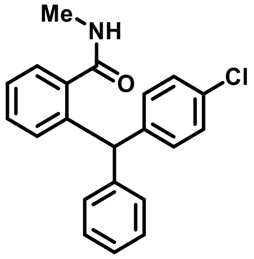

By following GP2, using 2-benzyl-*N*-(4-chlorophenyl)-*N*-methylbenzamide (**3h**) (67 mg, 0.2 mmol) as the aryl amide, heating the reaction mixture at 60 °C for 2 hours, and purifying by flash chromatography (SiO_2, 0-100% diethyl ether in *n*-pentane), afforded the title compound (61 mg, 91%) as a white solid.

M.p. 120-123 °C ($CDCl_3$). **IR (film, CH_2Cl_2, cm^{-1})** ν_{max} = 3291, 2924, 1634, 1489. **^{1}H NMR (400 MHz, $CDCl_3$)** δ 7.33 (1 H, dd, *J* 7.2, 1.6, ArC*H*), 7.31-7.21 (7 H, m, 2 x ArC*H* + 2 x ArC*H* + 3 x ArC*H*), 7.08 (2 H, d, *J* 7.3, 2 x ArC*H*), 7.04 (2 H, d, *J* 8.4, 2 x ArC*H*), 6.97 (1 H, d, *J* 7.7, ArC*H*), 6.10 (1 H, s, C*H*), 5.30 (1 H, br. s, N*H*), 2.71 (3 H, d, *J* 5.0, CH_3). **^{13}C NMR (101 MHz, $CDCl_3$)** δ 170.8 (*C*O), 143.0 (C_{quat}), 142.0 (C_{quat}), 141.4 (C_{quat}), 137.4 (C_{quat}), 132.3 (Ar*C*Cl), 131.0 (2 x Ar*C*H), 130.2 (Ar*C*H), 129.8 (Ar*C*H), 129.6 (2 x Ar*C*H), 128.6 (2 x Ar*C*H), 128.6 (2 x Ar*C*H), 127.2 (Ar*C*H), 126.7 (ArC*H* + ArC*H*), 51.8 (*C*H), 26.6 (NCH_3). **HRMS (ESI$^+$)** *m/z* calcd for $C_{21}H_{18}NOClNa^+$ $[M+Na]^+$ 358.0969; found 358.0975.

2-((4-Bromophenyl)(phenyl)methyl)-*N*-methylbenzamide (4i)

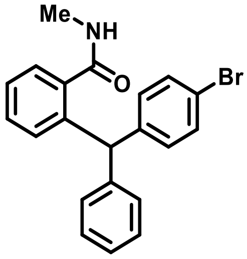

By following GP2, using 2-benzyl-*N*-(4-bromophenyl)-*N*-methylbenzamide (**3i**) (76 mg, 0.2 mmol) as the aryl amide, performing the reaction at 20 °C for 2 hours, and purifying by flash chromatography (SiO_2, 0-100% diethyl ether in *n*-pentane), afforded the title compound (46 mg, 61%) as a white solid.

M.p. 127-130 °C ($CDCl_3$). **IR (film, CH_2Cl_2, cm^{-1})** ν_{max} = 3295, 2934, 1635, 1486. **^{1}H NMR (400 MHz, $CDCl_3$)** δ 7.39 (2 H, d, *J* 8.4, 2 x ArC*H*), 7.34 (1 H, d, *J* 7.3, ArC*H*), 7.32-7.26 (3 H, m, 3 x ArC*H*), 7.26-7.21 (2 H, m, 2 x ArC*H*), 7.08 (2 H, d, *J* 7.3, 2 x ArC*H*), 7.00-6.96 (3 H, m, 2 x ArC*H* + ArC*H*), 6.08 (1 H, s, C*H*), 5.28 (1 H, br. s, N*H*), 2.71 (3 H, d, *J* 4.9, C*H*$_3$). **^{13}C NMR (101 MHz, $CDCl_3$)** δ 170.8 (*C*O), 142.9 (C_{quat}), 142.6 (C_{quat}), 141.3 (C_{quat}), 137.5 (C_{quat}), 131.5 (2 x Ar*C*H), 131.4 (2 x Ar*C*H), 130.3 (Ar*C*H), 129.8 (Ar*C*H), 129.6 (2 x Ar*C*H), 128.6 (2 x Ar*C*H), 127.2 (Ar*C*H), 126.8 (ArC*H* + ArC*H*), 120.5 (Ar*C*Br), 51.9 (*C*H), 26.6 (*C*H$_3$). **HRMS (ESI$^+$)** *m/z* calcd for $C_{21}H_{19}NOBr^+$ $[M+H]^+$ 380.0645; found 380.0627.

***N*-Methyl-2-(phenyl(pyridin-4-yl)methyl)benzamide (4j)**

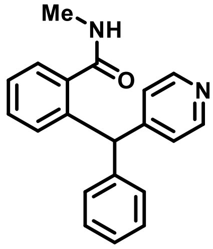

By following GP2, using 2-benzyl-*N*-methyl-*N*-(pyridin-4-yl)benzamide (**3j**) (61 mg, 0.2 mmol) as the aryl amide, heating the reaction mixture at 50 °C for 2.5 hours, and purifying by flash chromatography (SiO_2, 0-60% THF in *n*-pentane), afforded the title compound (60 mg, 98%) as a colourless film.

IR (film, CH_2Cl_2, cm^{-1}) ν_{max} = 3277, 3026, 1638, 1595. **^{1}H NMR (400 MHz, $CDCl_3$)** δ 8.46 (2 H, d, *J* 6.2, 2 x ArC*H*), 7.37-7.31 (2 H, m, ArC*H* + ArC*H*), 7.31-7.26 (3 H, m, 2 x ArC*H* + ArC*H*), 7.25-7.20 (1 H, m, ArC*H*), 7.08 (2 H, d, *J* 7.1, 2 x ArC*H*), 7.02 (2 H, d, *J* 6.2, 2 x ArC*H*), 6.96 (1 H, d, *J* 7.7, ArC*H*), 6.16 (1 H, s, C*H*), 5.46 (1 H, br. s, N*H*), 2.71 (3 H, d, *J* 4.9, C*H*$_3$). **^{13}C NMR (101 MHz, $CDCl_3$)** δ 170.6 (*C*O), 152.6 (C_{quat}), 149.9 (2 x Ar*C*H), 141.7 (C_{quat}), 140.4 (C_{quat}), 137.4 (C_{quat}), 130.3 (Ar*C*H), 130.0 (Ar*C*H), 129.6 (2 x Ar*C*H), 128.7 (2 x Ar*C*H), 127.2 (Ar*C*H), 127.0 (Ar*C*H + Ar*C*H), 124.9 (2 x Ar*C*H), 51.7 (*C*H), 26.6 (*C*H$_3$). **HRMS (ESI$^+$)** *m/z* calcd for $C_{20}H_{19}N_2O^+$ $[M+H]^+$ 303.1492; found 303.1500.

***N*-Methyl-2-(phenyl(pyridin-3-yl)methyl)benzamide (4k)**

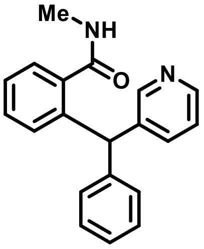

By following GP2, using 2-benzyl-*N*-methyl-*N*-(pyridin-3-yl)benzamide (**3k**) (61 mg, 0.2 mmol) as the aryl amide, heating the reaction mixture at 50 °C for 2.5 hours, and purifying by flash chromatography (SiO_2, 0-60% THF in *n*-pentane), afforded the title compound (54 mg, 89%) as a light-brown solid.

M.p. 148-151 °C ($CDCl_3$). **IR (film, CH_2Cl_2, cm^{-1})** ν_{max} = 3270, 2921, 1637, 1543. **^{1}H NMR (400 MHz, $CDCl_3$)** δ 8.42 (1 H, dd, *J* 4.8, 1.6, ArC*H*), 8.28 (1 H, d, *J* 2.2, ArC*H*), 7.44 (1 H, td, *J* 8.0, 1.9, ArC*H*), 7.36-7.31 (2 H, m, ArC*H* + ArC*H*), 7.31-7.27 (2 H, m, 2 x ArC*H*), 7.27-7.22 (2 H, m, ArC*H* + ArC*H*), 7.20 (1 H, dd, *J* 7.9, 4.8, ArC*H*), 7.13-7.08 (2 H, m, 2 x ArC*H*), 6.97 (1 H, d, *J* 7.9, ArC*H*), 6.21 (1 H, s, C*H*), 5.65 (1 H, br. s, N*H*), 2.70 (3 H, d, *J* 4.9, CH_3). **^{13}C NMR (101 MHz, $CDCl_3$)** δ 170.7 (*C*O), 150.9 (Ar*C*H), 147.7 (Ar*C*H), 142.2 (C_{quat}), 141.0 (C_{quat}), 139.0 (C_{quat}), 137.4 (C_{quat}), 137.3 (Ar*C*H), 130.1 (Ar*C*H), 129.9 (Ar*C*H), 129.6 (2 x Ar*C*H), 128.7 (2 x Ar*C*H), 127.2 (Ar*C*H), 126.9 (Ar*C*H), 126.9 (Ar*C*H), 123.3 (Ar*C*H), 50.1 (*C*H), 26.5 (*C*H_3). **HRMS (ESI$^+$)** *m/z* calcd for $C_{20}H_{19}N_2O^+$ [M+H]$^+$ 303.1492; found 303.1486.

***N*-Methyl-2-(phenyl(pyridin-2-yl)methyl)benzamide (4l)**

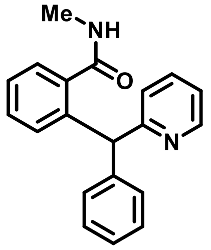

By following GP2, using 2-benzyl-*N*-methyl-*N*-(pyridin-2-yl)benzamide (**3l**) (61 mg, 0.2 mmol) as the aryl amide, heating the reaction mixture at 50 °C for 2 hours, and purifying by flash chromatography (SiO_2, 0-60% THF in *n*-pentane), afforded the title compound (53 mg, 88%) as a white solid.

M.p. 120-123 °C ($CDCl_3$). **IR (film, CH_2Cl_2, cm^{-1})** ν_{max} = 3285, 2937, 1636, 1538. **^{1}H NMR (400 MHz, $CDCl_3$)** δ 8.54 (1 H, ddd, *J* 4.9, 1.7, 0.8, ArC*H*), 7.64 (1 H, td, *J* 7.7, 1.8, ArC*H*), 7.45 (1 H, dd, *J* 7.5, 1.6, ArC*H*) 7.34-7.26 (4 H, m, 4 x ArC*H*), 7.26-7.24 (1 H, m, ArC*H*), 7.24-7.21 (1 H, m, ArC*H*), 7.18 (1 H, dd, *J* 7.7, 1.3, ArC*H*), 7.17-7.12 (3 H, m, 3 x ArC*H*), 6.86 (1 H, br. s, N*H*), 6.16 (1 H, s, C*H*), 2.74 (3 H, d, *J* 4.9, CH_3). **^{13}C NMR (101 MHz, $CDCl_3$)** δ 170.6 (*C*O), 163.1 (C_{quat}), 149.4 (Ar*C*H), 141.9 (C_{quat}), 139.9 (C_{quat}), 137.6 (C_{quat}), 136.9 (Ar*C*H), 130.6 (Ar*C*H), 129.7 (Ar*C*H), 129.3 (2 x Ar*C*H), 128.4 (2 x Ar*C*H), 128.1 (Ar*C*H), 126.8 (Ar*C*H), 126.7 (Ar*C*H), 124.2 (Ar*C*H), 121.6 (Ar*C*H), 55.1 (*C*H), 26.5 (*C*H_3). **HRMS (ESI$^+$)** *m/z* calcd for $C_{20}H_{19}N_2O^+$ [M+H]$^+$ 303.1492; found 303.1502.

2-((3-Chlorophenyl)(4-methoxyphenyl)methyl)-*N*-methylbenzamide (6a)

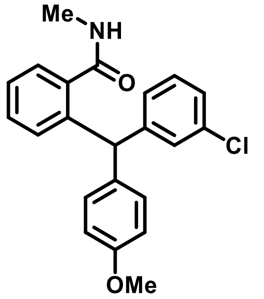

By following GP2, using *N*-(3-chlorophenyl)-2-(4-methoxybenzyl)-*N*-methylbenzamide (**5a**) (73 mg, 0.2 mmol) as the aryl amide, heating the reaction mixture at 50 °C for 3 hours, and purifying by flash chromatography (SiO_2, 0-100% diethyl ether in *n*-pentane), afforded the title compound (53 mg, 73%) as a colourless film.

IR (film, CH_2Cl_2, cm^{-1}) ν_{max} = 3296, 1641, 1510, 1249, 1179. **^{1}H NMR (400 MHz, $CDCl_3$)** δ 7.35-7.29 (2 H, m, ArC*H* + ArC*H*), 7.24 (1 H, td, *J* 7.4, 1.3, ArC*H*), 7.21-7.17 (2 H, m, ArC*H* + ArC*H*), 7.06 (1 H, br. s, ArC*H*), 7.02-7.00 (4 H, m, 2 x ArC*H* + ArC*H* + ArC*H*), 6.83 (2 H, d, *J* 8.8, 2 x ArC*H*), 6.04 (1 H, s, C*H*), 5.36 (1 H, br. s, N*H*), 3.78 (3 H, s, OCH_3), 2.72 (3 H, d, *J* 4.9, NCH_3). **^{13}C NMR (101 MHz, $CDCl_3$)** δ 170.8 (*C*O), 158.3 (Ar*C*O), 146.0 (C_{quat}), 141.4 (C_{quat}), 137.4 (C_{quat}), 134.7 (C_{quat}), 134.3 (Ar*C*Cl), 130.6 (2 x Ar*C*H), 130.2 (Ar*C*H), 129.8 (Ar*C*H), 129.6 (Ar*C*H), 129.5 (Ar*C*H), 127.9 (Ar*C*H), 127.2 (Ar*C*H), 126.7 (Ar*C*H), 126.7 (Ar*C*H), 114.0 (2 x Ar*C*H), 55.3 (O*C*H_3), 51.4 (*C*H), 26.6 (N*C*H_3). **HRMS (ESI$^+$)** *m/z* calcd for $C_{22}H_{21}ClNO_2^+$ $[M+H]^+$ 366.1255; found 366.1265.

Procedure for 3.7 mmol scale:

A flame-dried 100 mL RBF was sequentially charged with *N*-(3-chlorophenyl)-2-(4-methoxybenzyl)-*N*-methylbenzamide (**5a**) (1.34 g, 3.7 mmol), nitrogen and anhydrous THF (30 mL). The amide solution was sparged with nitrogen for 30 minutes, then 1 M KHMDS in THF (7.3 mL, 7.3 mmol) was added slowly at room temperature. The reaction mixture was heated to 50 °C and stirred for 1.25 hours. The reaction was quenched with brine (50 mL) and extracted with ethyl acetate (3 x 40 mL). The organic extracts were combined, dried ($MgSO_4$) and concentrated under reduced pressure giving crude material, which was purified by flash chromatography (SiO_2, 0-60% diethyl ether in 40-60 petroleum ether), affording the title compound (1.07 g, 80%) as a pale-yellow solid.

2-((3-Chlorophenyl)(*p*-tolyl)methyl)-*N*-methylbenzamide (6b)

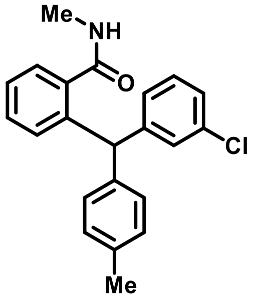

By following GP2, using *N*-(3-chlorophenyl)-*N*-methyl-2-(4-methylbenzyl)benzamide (**5b**) (70 mg, 0.2 mmol) as the aryl amide, heating the reaction mixture at 50 °C for 2 hours, and purifying by flash chromatography (SiO_2, 0-100% diethyl ether in *n*-pentane), afforded the title compound (46 mg, 66%) as a white foam.

IR (film, CH_2Cl_2, cm^{-1}) ν_{max} = 3290, 3021, 1633, 749. **^{1}H NMR (400 MHz, $CDCl_3$)** δ 7.34 (1 H, dd, *J* 7.6, 1.4, ArC*H*), 7.31 (1 H, td, *J* 7.6, 1.4, ArC*H*), 7.23 (1 H, td, *J* 7.6, 1.4, ArC*H*), 7.20-7.16 (2 H, m, ArC*H* + ArC*H*), 7.10 (2 H, d, *J* 8.0, 2 x ArC*H*), 7.08-7.07 (1 H, m, ArC*H*), 7.02-6.96 (4 H, m, 2 x ArC*H* + ArC*H* + ArC*H*), 6.05 (1 H, s, C*H*), 5.33 (1 H, br. s, N*H*), 2.72 (3 H, d, *J* 4.9, NCH_3), 2.32 (3 H, s, ArCCH_3). **^{13}C NMR (101 MHz, $CDCl_3$)** δ 170.7 (*C*O), 145.9 (C_{quat}), 141.2 (C_{quat}), 139.6 (C_{quat}), 137.5 (C_{quat}), 136.4 (C_{quat}), 134.3 (Ar*C*Cl), 130.2 (Ar*C*H), 129.8 (Ar*C*H), 129.6 (Ar*C*H), 129.6 (Ar*C*H), 129.4 (2 x Ar*C*H), 129.3 (2 x Ar*C*H), 128.0 (Ar*C*H), 127.2 (Ar*C*H), 126.7 (Ar*C*H), 126.7 (Ar*C*H), 51.9 (*C*H), 26.6 (N*C*H_3), 21.1 (ArC*C*H_3). **HRMS (ESI$^+$)** *m/z* calcd for $C_{22}H_{20}NOClNa^+$ $[M+Na]^+$ 372.1126; found 372.1125.

2-([1,1'-Biphenyl]-4-yl(3-chlorophenyl)methyl)-*N*-methylbenzamide (6c)

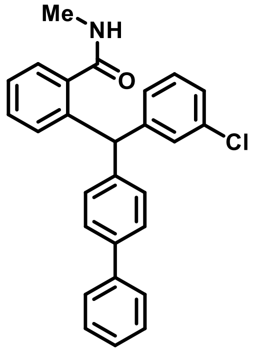

By following GP2, using 2-([1,1'-biphenyl]-4-ylmethyl)-*N*-(3-chlorophenyl)-*N*-methylbenzamide (**5c**) (82 mg, 0.2 mmol) as the aryl amide, heating the reaction mixture at 55 °C for 2.5 hours, and purifying by flash chromatography (SiO_2, 0-100% diethyl ether in *n*-pentane), afforded the title compound (62 mg, 76%) as a white solid.

M.p. 77-80 °C ($CDCl_3$). **IR (film, CH_2Cl_2, cm^{-1})** ν_{max} = 3290, 3056, 1636, 1529, 737. **^{1}H NMR (400 MHz, $CDCl_3$)** δ 7.59-7.56 (2 H, m, 2 x Ar*C*H), 7.53 (2 H, d, *J* 8.3, 2 x Ar*C*H), 7.43 (2 H, t, *J* 7.3, 2 x ArC*H*), 7.37-7.32 (3 H, m, ArC*H* + ArC*H* + ArC*H*), 7.27 (1 H, dd, *J* 7.8, 1.1, ArC*H*), 7.24-7.20 (2 H, m, ArC*H* + ArC*H*), 7.17 (2 H, d, *J* 8.3, 2 x Ar*C*H), 7.13-7.11 (1 H, m, Ar*C*H), 7.07-7.03 (2 H, m, ArC*H* + ArC*H*), 6.16 (1 H, s, C*H*), 5.35 (1 H, br. q, *J* 4.8, N*H*), 2.73 (3 H, d, *J* 4.8, C*H*$_3$). **^{13}C NMR (101 MHz, $CDCl_3$)** δ 170.8 (*C*O), 145.6 (C_{quat}), 141.8 (C_{quat}), 141.1 (C_{quat}), 140.7 (C_{quat}), 139.6 (C_{quat}), 137.4 (C_{quat}), 134.4 (Ar*C*Cl), 130.3 (Ar*C*H), 130.0 (2 x Ar*C*H), 129.9 (Ar*C*H), 129.7 (Ar*C*H), 129.7 (Ar*C*H), 128.9 (2 x Ar*C*H), 128.0 (Ar*C*H), 127.4 (ArCH), 127.3 (2 x Ar*C*H), 127.2 (Ar*C*H), 127.1 (2 x Ar*C*H), 126.9 (Ar*C*H), 126.8 (Ar*C*H), 51.9 (*C*H), 26.6 (*C*H$_3$). **HRMS (ESI$^+$)** *m/z* calcd for $C_{27}H_{22}ClNONa^+$ $[M+Na]^+$ 434.1282; found 434.1296.

2-((3-Chlorophenyl)(4-fluorophenyl)methyl)-*N*-methylbenzamide (6d)

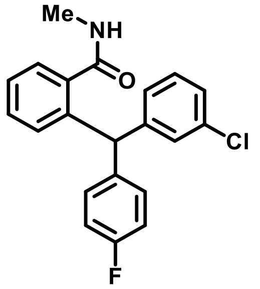

By following GP2, using *N*-(3-chlorophenyl)-2-(4-fluorobenzyl)-*N*-methylbenzamide (**5d**) (71 mg, 0.2 mmol) as the aryl amide, heating the reaction mixture at 55 °C for 2.5 hours, and purifying by flash chromatography (SiO_2, 0-100% diethyl ether in *n*-pentane), afforded the title compound (50 mg, 70%) as a colourless film.

IR (film, CH_2Cl_2, cm^{-1}) ν_{max} = 3288, 1633, 1506, 1223, 734. **^{1}H NMR (400 MHz, $CDCl_3$)** δ 7.36-7.30 (2 H, m, ArC*H* + ArC*H*), 7.27-7.23 (1 H, m, ArC*H*), 7.22-7.18 (2 H, m, ArC*H* + ArC*H*), 7.08-7.03 (3 H, m, 2 x ArC*H* + ArC*H*), 7.01-6.95 (4 H, m, 2 x ArC*H* + ArC*H* + ArC*H*), 6.15 (1 H, s, C*H*), 5.37 (1 H, br. s, N*H*), 2.73 (3 H, d, *J* 4.9, CH_3). **^{13}C NMR (101 MHz, $CDCl_3$)** δ 170.6 (*C*O), 161.6 (Ar*C*F, d, J_{CF} 245.3), 145.5 (C_{quat}), 141.2 (C_{quat}), 138.4 (C_{quat}, d, J_{CF} 3.5), 137.3 (C_{quat}), 134.4 (Ar*C*Cl), 131.1 (2 x Ar*C*H, d, J_{CF} 8.2), 130.1 (Ar*C*H), 130.0 (Ar*C*H), 129.8 (Ar*C*H), 129.6 (Ar*C*H), 127.9 (Ar*C*H), 127.2 (Ar*C*H), 126.9 (Ar*C*H), 126.9 (Ar*C*H), 115.4 (2 x Ar*C*H, d, J_{CF} 21.5), 51.3 (*C*H), 26.6 (*C*H_3). **^{19}F NMR (377 MHz, $CDCl_3$)** δ –116.0 (ArC*F*, tt, *J* 8.6, 5.4). **HRMS (ESI$^+$)** *m/z* calcd for $C_{21}H_{17}ClFNONa^+$ [M+Na]$^+$ 376.0875; found 376.0885.

2-((3-Chlorophenyl)(*o*-tolyl)methyl)-*N*-methylbenzamide (6e)

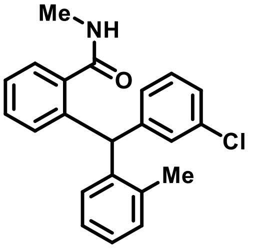

By following GP2, using *N*-(3-chlorophenyl)-*N*-methyl-2-(2-methylbenzyl)benzamide (**5e**) (70 mg, 0.2 mmol) as the aryl amide, performing the reaction mixture at 20 °C for 2 hours, and purifying by flash chromatography (SiO_2, 12-100% diethyl ether in 40-60 petroleum ether), afforded the title compound (45 mg, 64%) as a yellow solid.

M.p. 150-152 °C (CH_2Cl_2). **IR (film, $CDCl_3$, cm^{-1})** ν_{max} = 3301, 3064, 2928, 1635, 1594, 1570, 1532, 1474, 908, 730, 687. **^{1}H NMR (400 MHz, $CDCl_3$)** δ 7.35 (1 H, dd, *J* 7.4, 1.6, ArC*H*), 7.30 (1 H, td, *J* 7.4, 1.6, ArC*H*), 7.24 (1 H, td, *J* 7.4, 1.6, ArC*H*), 7.22-7.18 (2 H, m, ArC*H* + ArC*H*), 7.18-7.12 (2 H, m, ArC*H* + ArC*H*), 7.09 (1 H, td, *J* 7.3, 2.1, ArC*H*), 7.06-7.03 (1 H, m, ArC*H*), 7.00-6.94 (1 H, m, ArC*H*), 6.88 (1 H, dd, *J* 7.4, 1.6, ArC*H*), 6.76 (1 H, dd, *J* 7.5, 2.1, ArC*H*), 6.22 (1 H, s, C*H*), 5.19 (1 H, br. q *J* 4.8, N*H*), 2.64 (3 H, d, *J* 4.8, NCH_3), 2.21 (3 H, s, ArCCH_3). **^{13}C NMR (101 MHz, $CDCl_3$)** δ 170.7 (*C*O), 144.9 (C_{quat}), 141.2 (C_{quat}), 140.9 (C_{quat}), 137.5 (C_{quat}), 137.3 (C_{quat}), 134.5 (Ar*C*Cl), 130.8 (Ar*C*H), 130.1 (Ar*C*H), 129.8 (Ar*C*H), 129.8 (Ar*C*H), 129.7 (Ar*C*H), 129.1 (Ar*C*H), 128.2 (Ar*C*H), 127.3 (Ar*C*H), 126.9 (Ar*C*H), 126.8 (Ar*C*H), 126.8 (Ar*C*H), 126.0 (Ar*C*H), 49.2 (*C*H), 26.5 (N*C*H_3), 19.9 (ArC*C*H_3). **HRMS (ESI$^+$)** *m/z* calcd for $C_{22}H_{20}ClNONa^+$ $[M+Na]^+$ 372.1126; found 372.1127.

2-((3-Chlorophenyl)(naphthalen-1-yl)methyl)-*N*-methylbenzamide (6f)

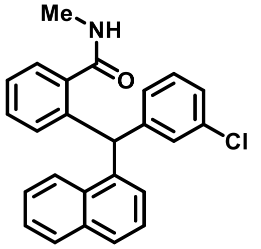

By following GP2, using *N*-(3-chlorophenyl)-*N*-methyl-2-(naphthalen-1-ylmethyl)benzamide (**5f**) (77 mg, 0.2 mmol) as the aryl amide, performing the reaction at 20 °C for 2 hours, and purifying by flash chromatography (SiO_2, 12-100% diethyl ether in 40-60 petroleum ether), afforded the title compound (66 mg, 86%) as a yellow solid.

M.p. 196-198 °C (CH_2Cl_2). **IR (film, $CDCl_3$, cm^{-1})** ν_{max} = 3300, 3061, 2935, 1634, 1594, 1571, 1532, 1474, 907, 790, 778, 730, 696. **^{1}H NMR (400 MHz, $CDCl_3$)** δ 8.13-8.07 (1 H, m, ArC*H*), 7.87-7.82 (1 H, m, ArC*H*), 7.77 (1 H, d, *J* 8.2, ArC*H*), 7.48-7.41 (2 H, m, ArC*H* + ArC*H*), 7.41-7.33 (2 H, m, ArC*H* + ArC*H*), 7.29-7.22 (2 H, m, ArC*H* + ArC*H*), 7.22-7.17 (2 H, m, ArC*H* + ArC*H*), 7.12-7.09 (1 H, m, ArC*H*), 7.02 (1 H, td, *J* 4.6, 1.7, ArC*H*), 6.95 (1 H, dt, *J* 7.2, 1.0, ArC*H*), 6.93-6.89 (1 H, m, ArC*H*), 6.88 (1 H, s, C*H*), 5.24 (1 H, br. q, *J* 4.9, N*H*), 2.56 (3 H, d, *J* 4.9, NCH_3). **^{13}C NMR (101 MHz, $CDCl_3$)** δ 170.7 (*C*O), 145.5 (C_{quat}), 140.9 (C_{quat}), 139.0 (C_{quat}), 137.2 (C_{quat}), 134.5 (Ar*C*Cl), 134.1 (C_{quat}), 131.9 (C_{quat}), 130.2 (Ar*C*H), 129.9 (Ar*C*H + Ar*C*H), 129.8 (Ar*C*H), 128.6 (Ar*C*H), 128.3 (Ar*C*H), 127.9 (Ar*C*H), 127.4 (Ar*C*H), 127.4 (Ar*C*H), 126.9 (Ar*C*H), 126.9 (Ar*C*H), 126.8 (Ar*C*H), 126.0 (Ar*C*H), 125.1 (Ar*C*H), 124.6 (Ar*C*H), 48.7 (*C*H), 26.5 (N*C*H_3). **HRMS (ESI$^+$)** *m/z* calcd for $C_{25}H_{21}ClNO^+$ $[M+H]^+$ 386.1306; found 386.1308.

2-((3-Chlorophenyl)(3,4-dimethylphenyl)methyl)-*N*-methylbenzamide (6g)

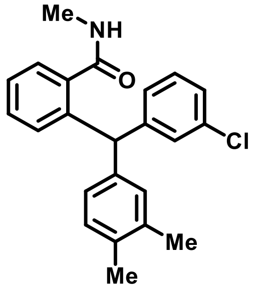

By following GP2, using *N*-(3-chlorophenyl)-2-(3,4-dimethylbenzyl)-*N*-methylbenzamide (**5g**) (73 mg, 0.2 mmol) as the aryl amide, performing the reaction at 20 °C for 2 hours, and purifying by flash chromatography (SiO_2, 12-100% diethyl ether in 40-60 petroleum ether), afforded the title compound (55 mg, 75%) as an off-white solid.

M.p. 57-58 °C (CH_2Cl_2). **IR (film, $CDCl_3$, cm^{-1})** ν_{max} = 3294, 2923, 2855, 1635, 1594, 1569, 1532, 1473, 908, 731, 687. **^{1}H NMR (400 MHz, $CDCl_3$)** δ 7.30-7.21 (2 H, m, ArC*H* + ArC*H*), 7.20-7.14 (1 H, m, ArC*H*), 7.13-7.08 (2 H, m, ArC*H* + ArC*H*), 7.04-6.91 (4 H, m, 4 x ArC*H*), 6.82 (1 H, s, ArC*H*), 6.72 (1 H, dd, *J* 7.7, 2.0, ArC*H*), 5.93 (1 H, s, C*H*), 5.25 (1 H, br. q, *J* 4.9, N*H*), 2.65 (3 H, d, *J* 4.9, NCH_3), 2.16 (3 H, s, ArCCH_3), 2.13 (3 H, s, ArCCH_3). **^{13}C NMR (101 MHz, $CDCl_3$)** δ 170.8 (*C*O), 146.0 (C_{quat}), 141.2 (C_{quat}), 140.0 (C_{quat}), 137.4 (C_{quat}), 136.8 (C_{quat}), 135.0 (C_{quat}), 134.3 (Ar*C*Cl), 130.8 (Ar*C*H), 130.3 (Ar*C*H), 129.8 (Ar*C*H), 129.8 (Ar*C*H), 129.6 (Ar*C*H + Ar*C*H), 128.0 (Ar*C*H), 127.2 (Ar*C*H), 126.9 (Ar*C*H), 126.7 (Ar*C*H), 126.6 (Ar*C*H), 51.9 (*C*H), 26.5 (N*C*H$_3$), 19.9 (ArC*C*H$_3$), 19.4 (ArC*C*H$_3$). **HRMS (ESI$^+$)** *m/z* calcd for $C_{23}H_{22}ClNONa^+$ $[M+Na]^+$ 386.1282; found 286.1301.

2-((3-Chlorophenyl)(3-fluoro-4-methylphenyl)methyl)-*N*-methylbenzamide (6h)

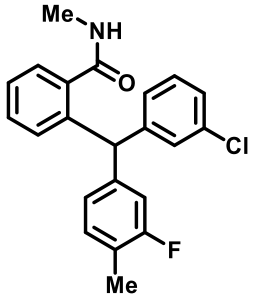

By following GP2, using *N*-(3-chlorophenyl)-2-(3-fluoro-4-methylbenzyl)-*N*-methylbenzamide (**5h**) (74 mg, 0.2 mmol) as the aryl amide, performing the reaction at 20 °C for 1 hour, and purifying by flash chromatography (SiO_2, 12-100% diethyl ether in 40-60 petroleum ether), afforded the title compound (50 mg, 68%) as a yellow solid.

M.p. 103-105 °C (CH_2Cl_2). **IR (film, CH_2Cl_2, cm^{-1})** ν_{max} = 3293, 2960, 2929, 1634, 1594, 1532, 1499, 1473, 1120, 908, 729, 686. **^{1}H NMR (400 MHz, $CDCl_3$)** δ 7.36-7.29 (2 H, m, ArC*H* + ArC*H*), 7.27-7.16 (3 H, m, ArC*H* + ArC*H* + ArC*H*), 7.07-7.04 (1 H, m, ArC*H*), 7.01-6.95 (2 H, m, ArC*H* + ArC*H*), 6.95-6.91 (1 H, m, ArC*H*), 6.89 (1 H, d, *J* 9.1, ArC*H*), 6.83 (1 H, ddd, *J* 8.1, 5.0, 2.4, ArC*H*), 6.08 (1 H, s, C*H*), 5.39 (1 H, br. s, N*H*), 2.73 (3 H, d, *J* 4.8, NCH_3), 2.20 (3 H, d, *J* 2.1, ArCCH_3). **^{13}C NMR (101 MHz, $CDCl_3$)** δ 170.7 (*C*O), 160.2 (d, J_{CF} 244.3, Ar*C*F), 145.6 (C_{quat}), 141.2 (C_{quat}), 138.1 (d, J_{CF} 3.7, C_{quat}), 137.3 (C_{quat}), 134.4 (Ar*C*Cl), 132.6 (d, J_{CF} 5.2, Ar*C*H), 130.2 (Ar*C*H), 129.9 (Ar*C*H), 129.7 (Ar*C*H), 129.5 (Ar*C*H), 128.3 (d, J_{CF} 7.9, Ar*C*H), 127.9 (Ar*C*H), 127.2 (Ar*C*H), 126.8 (Ar*C*H), 126.8 (Ar*C*H), 124.9 (d, J_{CF} 17.4, C_{quat}), 115.0 (d, J_{CF} 22.2, Ar*C*H), 51.3 (*C*H), 26.6 (N*C*H_3), 14.7 (d, J_{CF} 3.4, ArC*C*H_3). **^{19}F NMR (377 MHz, $CDCl_3$)** δ –120.5 (ArC*F*, dddt, *J* 9.3, 7.1, 4.8. 2.1). **HRMS (ESI$^+$)** *m/z* calcd for $C_{22}H_{19}ClFNONa^+$ $[M+Na]^+$ 390.1031; found 390.1039.

2-((3-Chlorophenyl)(dibenzo[*b,d*]thiophen-2-yl)methyl)-*N*-methylbenzamide (6i)

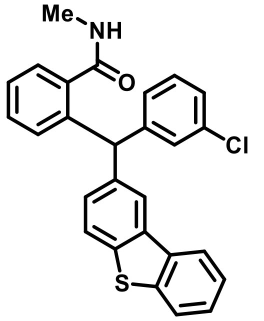

By following GP2, using *N*-(3-chlorophenyl)-2-(dibenzo[*b,d*]thiophen-2-ylmethyl)-*N*-methylbenzamide (**5i**) (88 mg, 0.2 mmol) as the aryl amide, heating the reaction mixture at 30 °C for 2.5 hours, and purifying by flash chromatography (SiO_2, 0-100% diethyl ether in *n*-pentane), afforded the title compound (73 mg, 83%) as a white solid.

M.p. 98-101 °C ($CDCl_3$). **IR (film, CH_2Cl_2, cm^{-1})** ν_{max} = 3298, 3059, 1640, 1535, 764. **^{1}H NMR (400 MHz, $CDCl_3$)** δ 8.03-8.00 (1 H, m, ArC*H*), 7.87 (1 H, br. d, *J* 1.8, ArC*H*), 7.85-7.82 (1 H, m, ArC*H*), 7.77 (1 H, d, *J* 8.3, ArC*H*), 7.45-7.40 (2 H, m, ArC*H* + ArC*H*), 7.38-7.32 (2 H, m, ArC*H* + ArC*H*), 7.28 (1 H, dd, *J* 7.4, 1.4, ArC*H*), 7.24-7.20 (3 H, m, ArC*H* + ArC*H* + ArC*H*), 7.14-7.12 (1 H, m, ArC*H*), 7.08-7.04 (2 H, m, ArC*H* + ArC*H*), 6.35 (1 H, s, C*H*), 5.32 (1 H, br. q, *J* 4.9, N*H*), 2.96 (3 H, d, *J* 4.9, CH_3). **^{13}C NMR (101 MHz, $CDCl_3$)** δ 170.8 (*C*O), 145.6 (C_{quat}), 141.3 (C_{quat}), 139.9 (C_{quat}), 139.2 (C_{quat}), 137.9 (C_{quat}), 137.5 (C_{quat}), 135.9 (C_{quat}), 135.4 (C_{quat}), 134.5 (Ar*C*Cl), 130.3 (Ar*C*H), 130.0 (Ar*C*H), 129.8 (Ar*C*H), 129.7 (Ar*C*H), 128.5 (Ar*C*H), 128.1 (Ar*C*H), 127.2 (Ar*C*H), 127.0 (Ar*C*H), 126.9 (Ar*C*H), 126.9 (Ar*C*H), 124.5 (Ar*C*H), 123.0 (Ar*C*H), 122.9 (Ar*C*H), 122.5 (Ar*C*H), 121.8 (Ar*C*H), 52.0 (*C*H), 26.6 (*C*H_3). **HRMS (ESI$^+$)** *m/z* calcd for $C_{27}H_{20}ClNOSNa^+$ $[M+H]^+$ 464.0846; found 464.0849.

2-Methoxy-*N*-methyl-6-(phenyl(quinolin-8-yl)methyl)benzamide (6j)

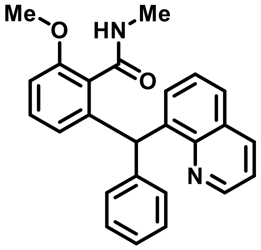

By following GP2, using 2-benzyl-6-methoxy-*N*-methyl-*N*-(quinolin-8-yl)benzamide (**5j**) (76 mg, 0.2 mmol) as the aryl amide, heating the reaction mixture at 100 °C (μwave) for 1 hour, and purifying by flash chromatography (SiO_2, 12-100% ethyl acetate in 40-60 petroleum ether), afforded the title compound (63 mg, 83%) as a yellow solid.

M.p. 203-205 °C (CH_2Cl_2). **IR (film, CH_2Cl_2, cm^{-1})** ν_{max} = 3259, 3061, 2934, 1659, 1597, 1580, 1495, 1467, 1262, 1066, 909, 726, 699. **^{1}H NMR (400 MHz, $CDCl_3$)** δ 8.81 (1 H, dd, *J* 4.2, 1.8, ArC*H*), 8.16 (1 H, dd, *J* 8.2, 1.8, ArC*H*), 8.02 (1 H, br. q, *J* 4.9, N*H*), 7.72 (1 H, dd, *J* 8.1, 1.2, ArC*H*), 7.47 (1 H, dd, *J* 8.1, 7.0, ArC*H*), 7.38 (1 H, dd, *J* 8.2, 4.2, ArC*H*), 7.34 (1 H, d, *J* 7.0, ArC*H*), 7.29-7.09 (6 H, m, 2 x ArC*H* + 2 x ArC*H* + ArC*H* + ArC*H*), 6.92 (1 H, s, C*H*), 6.75 (1 H, dd, *J* 8.4, 0.9, ArC*H*), 6.60 (1 H, dd, *J* 8.0, 0.9, ArC*H*), 3.83 (3 H, s, OCH_3), 2.93 (3 H, d, *J* 4.9, NCH_3). **^{13}C NMR (101 MHz, $CDCl_3$)** δ 168.6 (*C*O), 156.9 (C_{quat}), 149.3 (ArCH), 146.2 (C_{quat}), 143.0 (C_{quat}), 142.4 (C_{quat}), 141.1 (C_{quat}), 137.6 (Ar*C*H), 130.5 (Ar*C*H), 129.8 (2 x Ar*C*H), 129.2 (Ar*C*H), 128.7 (C_{quat}), 128.4 (2 x Ar*C*H), 127.6 (C_{quat}), 127.0 (Ar*C*H), 126.5 (Ar*C*H), 126.4 (Ar*C*H), 121.9 (Ar*C*H), 121.1 (Ar*C*H), 109.1 (Ar*C*H), 55.9 (O*C*H_3), 48.1 (*C*H), 26.5 (N*C*H_3). **HRMS (ESI$^+$)** *m/z* calcd for $C_{25}H_{23}N_2O_2^+$ $[M+H]^+$ 383.1754; found 383.1752.

14-Phenyl-7,8,9,14-tetrahydrodibenzo[*c,f*]azecin-5(6*H*)-one (8a)

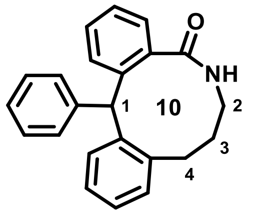

By following GP2, using (2-benzylphenyl)(3,4-dihydroquinolin-1(2*H*)-yl)methanone (**7a**) (262 mg, 0.8 mmol) as the aryl amide, heating the reaction mixture at 100 °C (µwave) for 2 hours, and purifying by flash chromatography (SiO_2, 7-60% ethyl acetate in 40-60 petroleum ether), afforded the title compound (160 mg, 61%) as an off-white solid.

M.p. 245-247 °C (CH_2Cl_2). **IR (film, $CDCl_3$, cm^{-1})** ν_{max} = 3193, 3064, 2934, 1647, 724. **^{1}H NMR (400 MHz, $CDCl_3$, mixture of rotamers A:B in a 0.6:0.4)** δ 7.70 (0.4 H, br. d, *J* 7.9, ArC*H* rot. B), 7.60 (0.4 H, br. d, *J* 7.3, ArC*H* rot. B), 7.51-7.11 (10.2 H, m, 11 × ArC*H* rot. A + 9 × ArCH rot. B), 7.11-6.94 (2 H, m, 2 × ArC*H* rot. A + 2 × ArC*H* rot. B), 6.89 (0.4 H, br. d, *J* 11.7, N*H* rot. B), 6.11 (0.6 H, br. s, C**1***H* rot. A), 5.88 (0.4 H, br. s, C**1***H* rot. B), 5.38 (0.6 H, br. d, *J* 9.3, N*H* rot. A), 4.41-4.21 (0.6 H, m, NC**2**H_aH_b rot. A), 3.37-3.16 (1 H, m, NC**2**H_aH_b rot. A + C**4**H_aH_b rot. B), 3.09 (0.4 H, br. d, *J* 13.6, NC**4**H_aH_b rot. B), 3.01-2.75 (1.6 H, m, NC**2**H_aH_b rot. B + C**4**H_aH_b rot. A + NC**2**H_aH_b rot. A), 2.55 (0.4 H, br. d, *J* 14.1, C**4**H_aH_b rot. B), 2.47-2.26 (0.6 H, m, C**3**H_aH_b rot. A), 2.14-1.86 (1 H, m, C**3**H_aH_b rot. B + C**3**H_aH_b rot. A), 1.63 (0.4 H, br. t, *J* 14.0, C**3**H_aH_b rot. B). **^{13}C NMR (101 MHz, $CDCl_3$, mixture of rotamers A:B in a 0.6:0.4)** δ 174.3 (*C*O rot. B), 170.8 (*C*O rot. A), 145.0 (C_{quat} rot. B), 143.0 (C_{quat} rot. A), 142.8 (C_{quat} rot. A), 142.3 (C_{quat} rot. B), 142.1 (C_{quat} rot. A), 140.2 (C_{quat} rot. B), 139.7 (C_{quat} rot. B), 139.4 (C_{quat} rot. A), 138.4 (C_{quat} rot. A), 136.9 (C_{quat} rot. B), 131.3 (2 × Ar*C*H rot. B), 130.6 (Ar*C*H rot. B), 130.6 (Ar*C*H rot. A), 129.9 (Ar*C*H rot. A + Ar*C*H rot. B), 129.7 (Ar*C*H rot. A), 129.7 (Ar*C*H rot. A), 129.3 (2 × Ar*C*H rot. A), 129.0 (2 × Ar*C*H rot. B), 128.1 (2 × Ar*C*H rot. A), 128.0 (2 × Ar*C*H rot. B), 127.3 (Ar*C*H rot. A + Ar*C*H rot. B), 127.1 (Ar*C*H rot. B), 126.8 (Ar*C*H rot. B), 126.8 (Ar*C*H rot. A), 126.6 (Ar*C*H rot. A), 126.5 (Ar*C*H rot. B), 126.4 (Ar*C*H rot. A), 126.2 (Ar*C*H rot. A), 126.0 (Ar*C*H rot. B), 48.2 (*C***1**H rot. A), 47.3 (*C***1**H rot. B), 41.2 (N*C***2**H_2 rot. A), 40.9 (N*C***2**H_2 rot. B), 32.4 (*C***3**H_2 rot. B), 30.0 (*C***3**H_2 rot. A), 29.0 (C**4**H_2 rot. A), 27.6 (*C***4**H_2 conf. B). **HRMS (ESI$^+$)** *m/z* calcd for $C_{23}H_{22}NO^+$ $[M+H]^+$ 328.1696; found 328.1699.

15-Phenyl-6,7,8,9,10,15-hexahydro-5*H*-dibenzo[*c,f*][1]azacycloundecin-5-one (8b)

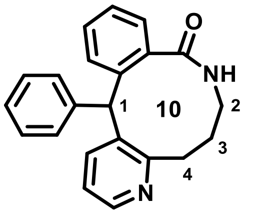

By following GP2, using (2-benzylphenyl)(3,4-dihydro-1,5-naphthyridin-1(2*H*)-yl)methanone (**7b**) (263 mg, 0.8 mmol) as the aryl amide, heating the reaction mixture at 100 °C (μwave) for 2 hours, and purifying by flash chromatography (SiO_2, 0-10% methanol in ethyl acetate) afforded the title compound (185 mg, 70%) as a pale orange solid.

M.p. 222-224 °C (CH_2Cl_2). **IR (film, $CDCl_3$, cm^{-1})** ν_{max} = 3264, 3061, 2934, 1646, 1601, 1538, 1444, 1432, 727, 699. **^{1}H NMR (400 MHz, $CDCl_3$, mixture of rotamers A:B in a 0.65:0.35)** δ 8.50 (0.65 H, dd, *J* 4.6, 1.7, ArC*H* rot. A), 8.48 (0.35 H, dd, *J* 4.6, 1.7, ArC*H* rot. B), 7.88 (0.35 H, dd, *J* 8.0, 1.7, ArC*H* rot. B), 7.71 (0.65 H, dd, *J* 8.0, 1.7, ArC*H* rot. A), 7.54-7.49 (0.35 H, m, ArC*H* rot. B), 7.41-7.18 (6.65 H, m, 7 x ArC*H* rot. A + 6 x ArC*H* rot. B), 7.18-7.14 (0.35 H, m, ArC*H* rot. B), 7.11 (0.65 H, dd, *J* 8.0, 4.6, ArC*H* rot. A), 7.04-6.99 (1.3 H, m, 2 x ArC*H* rot. A), 6.99-6.94 (0.7 H, m, 2 x ArC*H* rot. B), 6.51 (0.35 H, d, *J* 11.7, N*H* rot. B), 6.08 (0.65 H, s, C**1***H* rot. A), 5.86 (0.35 H, s, C**1***H* rot. B), 5.19 (0.65 H, d, *J* 10.0 N*H* rot. A), 4.41-4.28 (0.65 H, m, C**2**$H_a$$H_b$ rot. A), 3.47 (0.35 H, td, *J* 13.0, 3.1, C**4**$H_a$$H_b$ rot. B), 3.20 (0.65 H, ddt, *J* 13.7, 5.7, 1.7, C**2**$H_a$$H_b$ rot. A), 3.16-3.08 (1 H, m, C**4**$H_a$$H_b$ rot. A + C**2**$H_a$$H_b$ rot. B), 2.95 (0.65 H, ddd, *J* 14.1, 7.9, 1.9, C**4**$H_a$$H_b$ rot. A), 2.88-2.73 (0.7 H, m, C**2**$H_a$$H_b$ rot. B + C**4**$H_a$$H_b$ rot. B), 2.36-2.17 (1.3 H, m, C**3**H_2 rot. A), 2.12-1.93 (0.7 H, m, C**3**H_2 rot. B). **^{13}C NMR (101 MHz, $CDCl_3$, mixture of rotamers A:B in a 0.65:0.35)** δ 173.9 (*C*O rot. B), 170.5 (*C*O rot. A), 161.6 (C_{quat} rot. A), 159.1 (C_{quat} rot. B), 148.5 (Ar*C*H rot. B), 148.3 (Ar*C*H rot. A), 143.8 (C_{quat} rot. B), 142.0 (C_{quat} rot. A), 141.4 (C_{quat} rot. A), 139.1 (C_{quat} rot. B), 138.9 (C_{quat} rot. A), 138.5 (Ar*C*H rot. A), 138.2 (C_{quat} rot. B), 137.8 (Ar*C*H rot. B), 136.9 (C_{quat} rot. B), 135.5 (C_{quat} rot. A), 129.9 (Ar*C*H rot. B), 129.8 (Ar*C*H rot. A), 129.3 (2 x Ar*C*H rot. B), 129.2 (2 x Ar*C*H rot. A + Ar*C*H rot. B), 129.0 (Ar*C*H rot. A), 128.4 (2 x Ar*C*H rot. A), 128.3 (2 x Ar*C*H rot. B), 127.7 (Ar*C*H rot. B), 127.2 (Ar*C*H rot. A), 126.9 (Ar*C*H rot. B), 126.8 (Ar*C*H rot. A), 126.5 (Ar*C*H rot. B), 126.2 (Ar*C*H rot. A), 122.0 (Ar*C*H rot. B), 121.3 (Ar*C*H rot. A), 48.0 (*C***1**H rot. A), 47.4 (*C***1**H rot. B), 41.5 (*C***2**H_2 rot. B), 40.6 (*C***2**H_2 rot. A), 31.3 (*C***4**H_2 rot. A), 30.7 (*C***3**H_2 rot. B), 30.2 (*C***4**H_2 rot. B), 28.4 (*C***3**H_2 rot. A). **HRMS (ESI$^+$)** *m/z* calcd for $C_{22}H_{20}N_2ONa^+$ $[M+Na]^+$ 351.1468; found 351.1476.

15-Phenyl-6,7,8,9,10,15-hexahydro-5*H*-dibenzo[*c,f*][1]azacycloundecin-5-one (8c)

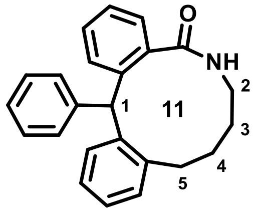

By following GP2, using (2-benzylphenyl)(2,3,4,5-tetrahydro-1*H*-benzo[*b*]azepin-1-yl)methanone (**7c**) (273 mg, 0.8 mmol) as the aryl amide, heating the reaction mixture at 100 °C (μwave) for 2 hours, and purifying by flash chromatography (SiO_2, 7-60% ethyl acetate in 40-60 petroleum ether), afforded the title compound (165 mg, 60%) as an off-white solid.

M.p. 222-224 °C (CH_2Cl_2). **IR (film, $CDCl_3$, cm^{-1})** ν_{max} = 3284, 2923, 2855, 1633, 1599, 1524, 1446, 908, 757, 728, 699, 612. **^{1}H NMR (400 MHz, $CDCl_3$)** δ 7.46 (1 H, d, *J* 7.6, ArC*H*), 7.36-7.16 (9 H, m, 2 x ArC*H* + 7 x ArC*H*), 7.05 (1 H, dd, *J* 7.7, 1.4, ArC*H*), 7.01-6.96 (2 H, m, 2 x ArC*H*), 6.10 (1 H, s, C**1***H*), 5.16 (1 H, d, *J* 8.8, N*H*), 3.91 (1 H, dddd, *J* 13.5, 10.9, 8.8, 2.4 C**2**H_aH_b), 3.02 (1 H, ddd, *J* 15.0, 9.1, 5.0, C**5**H_aH_b), 2.79 (1 H, ddt, *J* 13.5, 6.4, 2.4, C**2**H_aH_b), 2.61 (1 H, dt, *J* 15.0, 6.0, C**5**H_aH_b), 2.18-1.99 (2 H, m, C**4**H_2), 1.82 (1 H, ddt, *J* 17.9, 10.1, 3.7, 2.4, C**3**H_aH_b), 1.49 (1 H, dddd, *J* 17.9, 10.9, 6.4, 3.1, C**3**H_aH_b). **^{13}C NMR (101 MHz, $CDCl_3$)** δ 170.0 (*C*O), 142.5 (C_{quat}), 141.5 (C_{quat}), 140.8 (C_{quat}), 139.5 (C_{quat}), 137.9 (C_{quat}), 130.7 (Ar*C*H), 130.6 (Ar*C*H), 130.2 (Ar*C*H), 129.7 (Ar*C*H), 129.5 (2 x Ar*C*H), 128.3 (2 x Ar*C*H), 128.2 (Ar*C*H), 127.1 (Ar*C*H), 126.9 (Ar*C*H), 126.6 (Ar*C*H), 126.5 (Ar*C*H), 48.9 (*C***1**H), 40.2 (*C***2**H_2), 29.1 (*C***5**H_2), 27.5 (*C***4**H_2), 25.6 (*C***3**H_2). **HRMS (ESI$^+$)** *m/z* calcd for $C_{24}H_{23}NONa^+$ $[M+Na]^+$ 364.1672; found 364.1660.

11-Phenyl-5,11,16,17-tetrahydro-6*H*-tribenzo[*b*,*f*,*i*][1]azacycloundecin-6-one (8d)

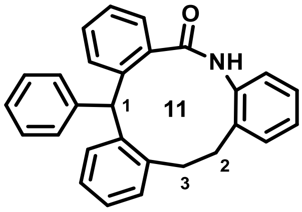

By following GP2, using (2-benzylphenyl)(10,11-dihydro-5*H*-dibenzo[*b*,*f*]azepin-5-yl)methanone (**7d**) (312 mg, 0.8 mmol) as the aryl amide, heating the reaction mixture at 100 °C (μwave) for 2 hours, and purifying by flash chromatography (SiO_2, 7-60% ethyl acetate in 40-60 petroleum ether), afforded the title compound (157 mg, 50%) as a yellow solid.

M.p. decomposed at 102 °C (CH_2Cl_2). **IR (film, $CDCl_3$, cm^{-1})** ν_{max} = 3289, 2859, 1649, 1600, 1520, 1494, 1450, 909, 753, 731, 700. **^{1}H NMR (400 MHz, $CDCl_3$)** δ 7.53 (1 H, d, *J* 7.0, ArC*H*), 7.39-7.00 (13 H, m, 13 x ArC*H*), 6.94 (1 H, d, *J* 7.7, ArC*H*), 6.89 (1 H, t, *J* 7.7, ArC*H*), 6.62 (1 H, d, *J* 7.7, ArC*H*), 6.31 (1 H, s, C**1***H*), 5.99 (1 H, br. s, N*H*), 3.89 (1 H, td, *J* 13.8, 6.5, C**2**H_aH_b), 3.22 (1 H, dd, *J* 13.8, 6.5, C**3**H_aH_b), 2.91 (1 H, dd, *J* 13.8, 6.5, C**2**H_aH_b), 2.71 (1 H, td, *J* 13.8 6.5, C**3**H_aH_b). **^{13}C NMR (101 MHz, $CDCl_3$)** δ 168.7 (*C*O), 143.5 (C_{quat}), 141.7 (C_{quat}), 141.1 (C_{quat}), 138.1 (C_{quat}), 137.9 (C_{quat}), 137.3 (C_{quat}), 135.5 (C_{quat}), 132.7 (Ar*C*H), 131.1 (Ar*C*H), 130.2 (Ar*C*H), 129.9 (2 x Ar*C*H), 129.8 (Ar*C*H), 128.9 (Ar*C*H), 128.6 (2 x Ar*C*H), 128.2 (Ar*C*H), 127.9 (Ar*C*H), 127.5 (Ar*C*H), 127.1 (Ar*C*H + Ar*C*H), 127.0 (Ar*C*H), 126.9 (Ar*C*H), 126.3 (Ar*C*H), 49.6 (*C***1**H), 35.6 (*C***3**H_2), 32.1 (*C***2**H_2). **HRMS (ESI$^+$)** *m/z* calcd for $C_{28}H_{23}NONa^+$ $[M+Na]^+$ 412.1672; found 412.1666.

2-((3-Chlorophenyl)(4-methoxyphenyl)methyl)-*N*-methyl-*N*-nitrosobenzamide (9)

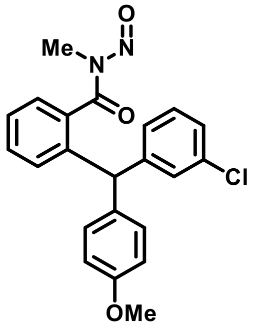

A flame-dried 2 mL microwave vial was charged with 2-((3-chlorophenyl)(4-methoxyphenyl)methyl)-*N*-methylbenzamide (**6a**) (37 mg, 0.1 mmol), nitrogen and DCE (0.5 mL). The amide solution was sparged with nitrogen for 10 minutes, then *tert*-butyl nitrite was added (36 μL, 31 mg, 0.3 mmol) was added. The reaction mixture was heated to 50 °C and stirred for 3.5 hours, then the reaction mixture was concentrated under reduced pressure giving crude material, which was purified by flash chromatography (SiO_2, 0-25% diethyl ether in 40-60 petroleum ether), affording the title compound (30 mg, 76%) as a colourless film.

IR (film, CH_2Cl_2, cm^{-1}) ν_{max} = 2955, 1709, 1509, 1251, 964. **^{1}H NMR (400 MHz, $CDCl_3$)** δ 7.39 (1 H, ddd, *J* 7.9, 6.8, 2.2, ArC*H*), 7.34-7.28 (2 H, m, 2 x ArC*H*), 7.18-7.14 (2 H, m, 2 x ArC*H*), 7.04 (1 H, br. s, ArC*H*), 6.99 (1 H, d, *J* 7.9, ArC*H*), 6.96 (3 H, m, 2 x ArC*H* + ArC*H*), 6.77 (2 H, d, *J* 8.8, 2 x ArC*H*), 5.79 (1 H, s, C*H*), 3.77 (3 H, s, OCH_3), 2.95 (3 H, s, NCH_3). **^{13}C NMR (101 MHz, $CDCl_3$)** δ 175.5 (*C*O), 158.5 (Ar*C*O), 144.7 (C_{quat}), 142.0 (C_{quat}), 134.6 (C_{quat}), 134.4 (Ar*C*Cl), 133.6 (C_{quat}), 130.8 (2 x Ar*C*H), 130.5 (Ar*C*H), 129.9 (Ar*C*H), 129.8 (Ar*C*H), 129.7 (Ar*C*H), 128.9 (Ar*C*H), 128.1 (Ar*C*H), 127.0 (Ar*C*H), 126.2 (Ar*C*H), 114.0 (2 x Ar*C*H), 55.4 (OCH_3), 52.3 (*C*H), 25.6 (NCH_3). **HRMS (ESI$^+$)** *m/z* calcd for $C_{22}H_{19}ClN_2O_3Na^+$ $[M+Na]^+$ 417.0976; found 417.0965.

2-((3-Chlorophenyl)(4-methoxyphenyl)methyl)benzoic acid (10)

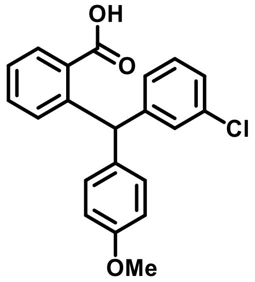

A flame-dried 5 mL microwave vial was charged with 2-((3-chlorophenyl)(4-methoxyphenyl)methyl)-*N*-methylbenzamide (**6a**) (73 mg, 0.2 mmol), nitrogen and DCE (1.0 mL). The amide solution was sparged with nitrogen for 10 minutes, then *tert*-butyl nitrite (71 μL, 62 mg, 0.6 mmol) was added. The reaction mixture was heated to 50 °C and stirred for 14 hours, contents of the reaction vessel was concentrated under reduced, flushed with nitrogen and charged with DMF (1.0 mL). The solution of crude material was cooled to 0 °C and a solution of sodium hydroxide (32 mg, 0.4 mmol) in water (0.5 mL) was added. The reaction mixture was stirred at 0 °C for 1 hour, then quenched with 1 M $HCl_{(aq)}$ (3 mL) and extracted with ethyl acetate (4 x 2 mL). The combined organic extract were concentrated under reduced pressure giving crude material, which was purified by flash chromatography (SiO_2, 0-40% ethyl acetate in 40-60 petroleum ether) yielding the title compound (57 mg, 81%) as a colourless oil.

IR (film, $CDCl_3$, cm^{-1}) ν_{max} = 2959, 1689, 1509, 1247, 731. **^1H NMR (400 MHz, $CDCl_3$)** δ 8.06 (1 H, dd, *J* 7.9, 1.4, ArC*H*), 7.47 (1 H, td, *J* 7.6, 1.6, ArC*H*), 7.33 (1 H, td, *J* 7.6, 1.4, ArC*H*), 7.22-7.15 (2 H, m, ArC*H* + ArC*H*), 7.08-7.03 (2 H, m, ArC*H* + ArC*H*), 6.97 (2 H, d, *J* 8.8, 2 x ArC*H*), 6.96-6.93 (1 H, m, ArC*H*), 6.82 (2 H, d, *J* 8.8, 2 x ArC*H*), 6.65 (1 H, s, C*H*), 3.78 (3 H, s, OCH_3). O*H* not observed. **^{13}C NMR (101 MHz, $CDCl_3$)** δ 172.3 (*C*O), 158.3 (Ar*C*O), 146.4 (C_{quat}), 145.8 (C_{quat}), 135.1 (C_{quat}), 134.3 (Ar*C*Cl), 132.9 (Ar*C*H), 131.9 (Ar*C*H), 131.3 (Ar*C*H), 130.7 (2 x Ar*C*H), 129.7 (Ar*C*H), 129.6 (Ar*C*H), 128.7 (C_{quat}), 128.0 (Ar*C*H), 126.7 (Ar*C*H), 126.6 (Ar*C*H), 113.9 (2 x Ar*C*H), 55.4 (OCH_3), 51.2 (*C*H). **HRMS (ESI$^+$)** *m/z* calcd for $C_{21}H_{17}ClO_3Na^+$ $[M+Na]^+$ 375.0758; found 375.0771.

1-Chloro-3-((4-methoxyphenyl)(phenyl)methyl)benzene (11)

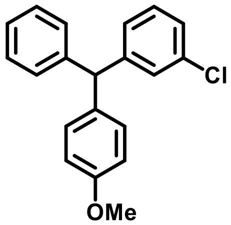

A 2 mL microwave vial was sequentially charged with 2-((3-chlorophenyl)(4-methoxyphenyl)methyl)benzoic acid (**10**) (57 mg, 0.16 mmol), *N*-methyl-2-pyrrolidone (0.5 mL), quinoline (0.2 mL), 1,10-phenanthroline monohydrate (32 mg, 0.16 mmol) and copper(II) acetate (12 mg, 0.06 mmol). The reaction mixture was sealed and stirred at 160 °C (μwave) for 1 hour. The reaction mixture was quenched with 1 M HCl (15 mL) and extracted with ethyl acetate (3 x 8 mL). The combined organic extracts were dried ($MgSO_4$) and concentrated under reduced pressure giving crude material, which was purified by flash chromatography (SiO_2, 0-20% diethyl ether in 40-60 petroleum ether), affording the title compound (25 mg, 51%) as a pale-white oil.

IR (film, CH_2Cl_2, cm^{-1}) ν_{max} = 2927, 1509, 1240, 749. **^{1}H NMR (400 MHz, $CDCl_3$)** δ 7.33-7.28 (2 H, m, 2 x ArC*H*), 7.25-7.22 (1 H, m, ArC*H*), 7.22-7.19 (2 H, m, ArC*H* + ArC*H*), 7.10 (3 H, d, *J* 6.9, 2 x ArC*H* + ArC*H*), 7.02 (2 H, d, *J* 8.9, 2 x ArC*H*), 7.01-6.98 (1 H, m, ArC*H*), 6.84 (2 H, d, *J* 8.9, 2 x ArC*H*), 5.47 (1 H, s, C*H*), 3.80 (3 H, s, OCH_3). **^{13}C NMR (101 MHz, $CDCl_3$)** δ 158.3 (Ar*C*O), 146.5 (C_{quat}), 143.6 (C_{quat}), 135.4 (C_{quat}), 134.4 (Ar*C*Cl), 130.4 (2 x Ar*C*H), 129.6 (Ar*C*H), 129.6 (Ar*C*H), 129.4 (2 x Ar*C*H), 128.6 (2 x Ar*C*H), 127.7 (Ar*C*H), 126.6 (Ar*C*H), 126.6 (Ar*C*H), 114.0 (2 x Ar*C*H), 55.8 (*C*H), 55.4 (O*C*H_3). **HRMS (EI$^+$)** *m/z* calcd for $C_{20}H_{17}ClO^+$ $[M]^+$ 308.0962; found 308.0960.

8-((3-Chlorophenyl)(4-methoxyphenyl)methyl)-2-methyl-3,4-diphenylisoquinolin-1(2*H*)-one (12)

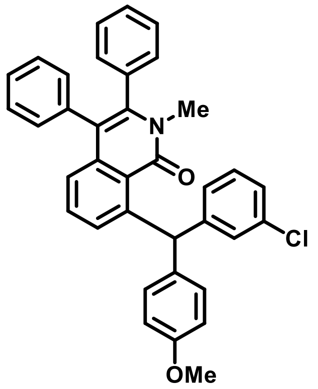

A 2 mL microwave vial was sequentially charged with 2-((3-chlorophenyl)(4-methoxyphenyl)methyl)-*N*-methylbenzamide (**6a**) (37 mg, 0.1 mmol), palladium(II) acetate (2 mg, 0.01 mmol), copper(II) acetate (7 mg, 0.04 mmol), 1,2-diphenylethyne (54 mg, 0.3 mmol), potassium carbonate (14 mg, 0.1 mmol), sodium iodide (30 mg, 0.2 mmol) and DMF (0.5 mL). While open to air the reaction mixture was heated to 120 °C and stirred for 22 hours. The reaction mixture was cooled to room temperature, quenched with water (5 mL) and extracted with ethyl acetate (3 x 5 mL). The combined organic extracts were concentrated under reduced pressure to give crude material, which was purified by flash chromatography (SiO_2, 0-50% diethyl ether in 40-60 petroleum ether), affording the title compound (19 mg, 35%) as a colourless film.

IR (film, $CDCl_3$, cm^{-1}) ν_{max} = 2957, 1643, 1592, 1509, 1248, 700. **^{1}H NMR (400 MHz, $CDCl_3$)** δ 7.79 (1 H, s, C*H*), 7.37 (1 H, t, *J* 7.9, ArC*H*), 7.24-7.14 (8 H, m, 8 x ArC*H*), 7.12-7.02 (10 H, m, 10 x ArC*H*), 6.85 (2 H, d, *J* 8.8, 2 x ArC*H*), 3.80 (3 H, s, OCH_3), 3.24 (3 H, s, NCH_3). **^{13}C NMR (126 MHz, $CDCl_3$)** δ 162.8 (*C*O), 158.0 (Ar*C*O), 147.9 (C_{quat}), 146.3 (C_{quat}), 141.6 (C_{quat}), 139.5 (C_{quat}), 137.3 (C_{quat}), 136.6 (C_{quat}), 135.4 (C_{quat}), 134.1 (Ar*C*Cl), 131.8 (2 x Ar*C*H), 131.1 (2 x Ar*C*H), 131.0 (Ar*C*H), 130.2 (Ar*C*H), 129.9 (2 x Ar*C*H), 129.9 (Ar*C*H), 129.4 (Ar*C*H), 128.5 (Ar*C*H), 128.3 (2 x Ar*C*H), 128.2 (Ar*C*H), 128.1 (2 x Ar*C*H), 126.9 (Ar*C*H), 126.1 (Ar*C*H), 124.8 (Ar*C*H), 122.7 (C_{quat}), 118.8 (C_{quat}), 113.8 (2 x Ar*C*H), 55.3 (O*C*H_3), 51.5 (*C*H), 35.0 (N*C*H_3). **HRMS (ESI$^+$)** *m/z* calcd for $C_{36}H_{29}NO_2Cl^+$ $[M+H]^+$ 542.1881; found 542.1894.

6. Mechanistic Studies

6.1 Deuterium Labelling Studies

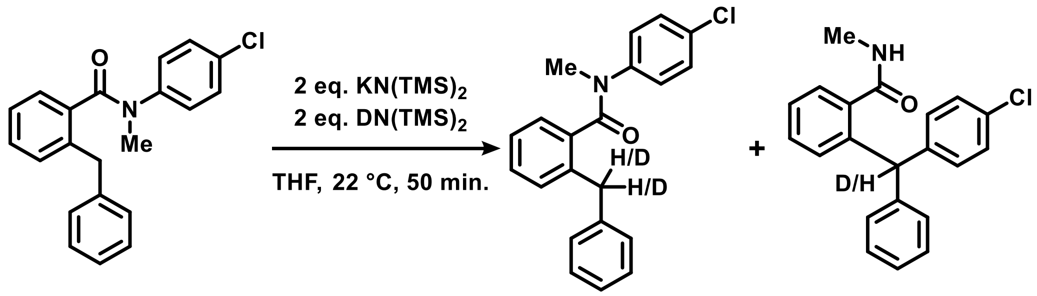

A 5 mL flame dried microwave vial was charged sequentially with 2-benzyl-*N*-(4-chlorophenyl)-*N*-methylbenzamide (**3h**) (34 mg, 0.1 mmol), nitrogen and anhydrous THF (1.1 mL). Under an inert atmosphere, a solution of KHMDS (0.5 M) and $DN(TMS)_2$ (0.5 M) in THF (1.0 mL) was prepared by adding deterium oxide (9 µL, 0.5 mmmol) to a 1 M solution of KHMDS in THF (1.0 mL). 0.4 mL of the KHMDS/$DN(TMS)_2$ solution was added to the 2-benzyl-*N*-(4-chlorophenyl)-*N*-methylbenzamide (**3h**) solution through a 0.45 µm nylon syringe filter pre-wetted with anhydrous THF. The reaction mixture was stirred for 50 minutes at 22 °C. The reaction was quenched with water (2 mL) and extracted with ethyl acetate (3 x 2 mL). The combined organics were concentrated under reduced pressure and purified by flash column chromatography (SiO_2, 0-100% diethyl ether in *n*-pentane) returning 2-benzyl-*N*-(4-chlorophenyl)-*N*-methylbenzamide (**3h**) (16 mg, 47%, 29% deterium incorperation) as a colourless film, and yielding 2-((4-chlorophenyl)(phenyl)methyl)-*N*-methylbenzamide (**4h**) (11 mg, 32%, 45% deuterium incorporation) as a colourless film.

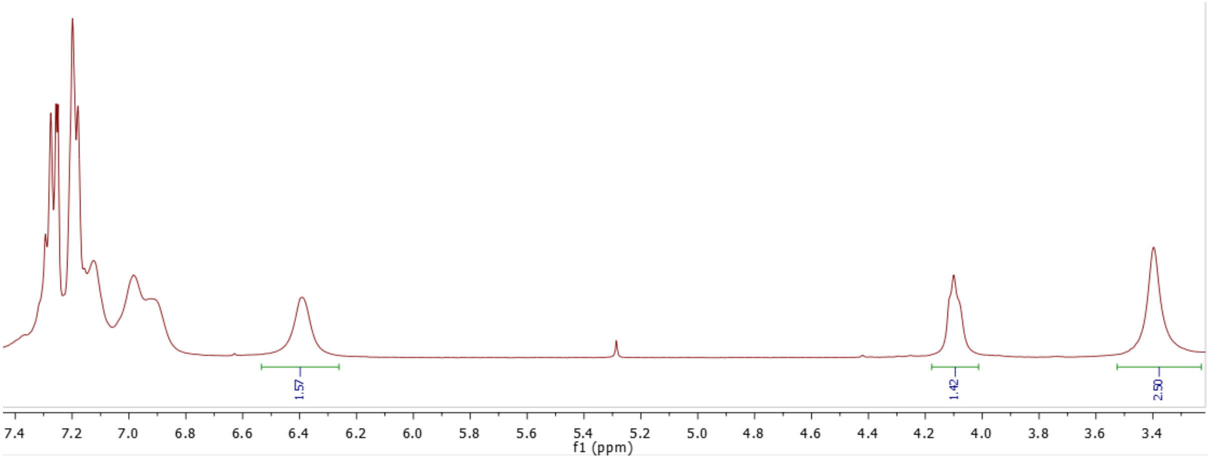

Figure S1. ^{1}H NMR spectra of 2-benzyl-*N*-(4-chlorophenyl)-*N*-methylbenzamide (**3h**) obtained from deuterium labelling studies.

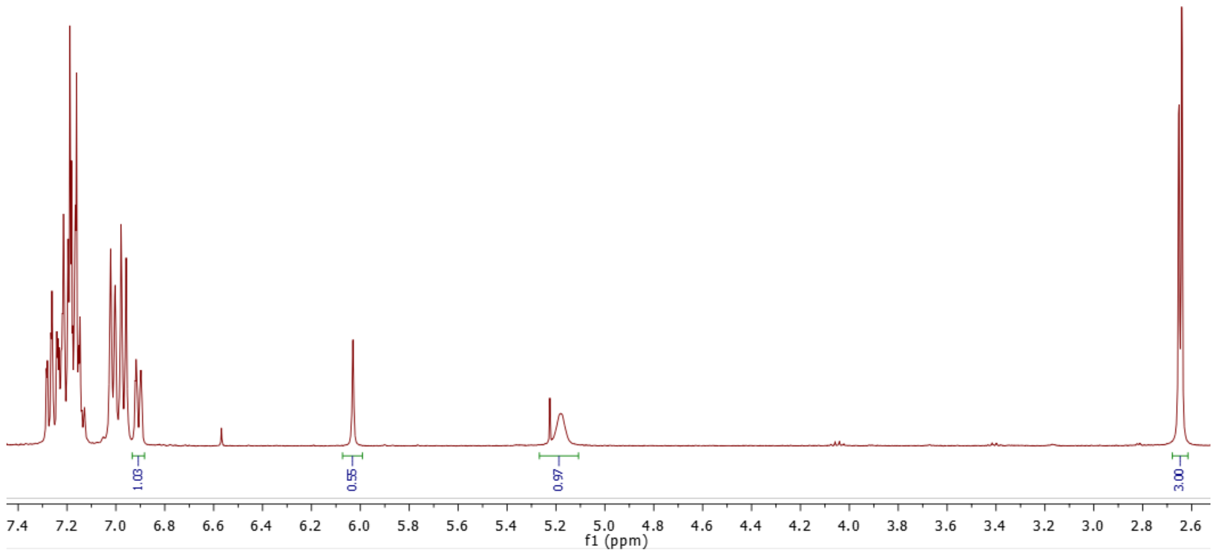

Figure S2. ^{1}H NMR spectra of 2-((4-chlorophenyl)(phenyl)methyl)-*N*-methylbenzamide (**4h**) obtained from deuterium labelling studies.

6.2 Hammett Plot

***In situ* IR spectroscopy (ReactIR)**

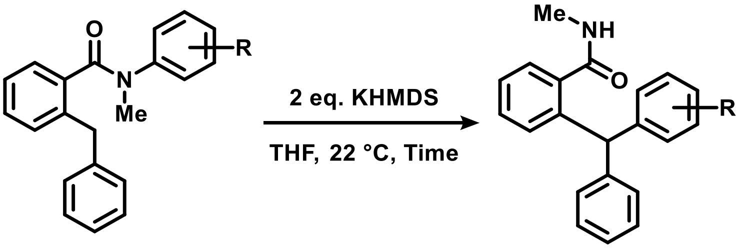

A background spectrum was obtained in anhydrous THF at 22 °C prior to each *in situ* IR analysis run.

Under an inert atmsophere, a solution of *N*-arylbenzamide derivative (0.25 mmol, 1.0 eq.) in anhydrous THF (1 mL, 0.25 M) was added to a three-neck RBF equipped with a stirrer bar, ReactIR probe and nitrogen inlet; the remaining neck was used for the addition of reagents. *In situ* IR analysis was intitated and the spectra of a *N*-arylbenzamide solution was allowed to stabilise for at least 20 minutes prior to addition of 1 M KHMDS in THF (0.5 mmol , 0.5 mL). The reaction mixture was stirred at 22 °C until where possible product anion formation peaks reached a plateau. The reaction mixture was then syringed out of the three-neck RBF and quenched in water (5 mL).

***In situ* IR Spectra**

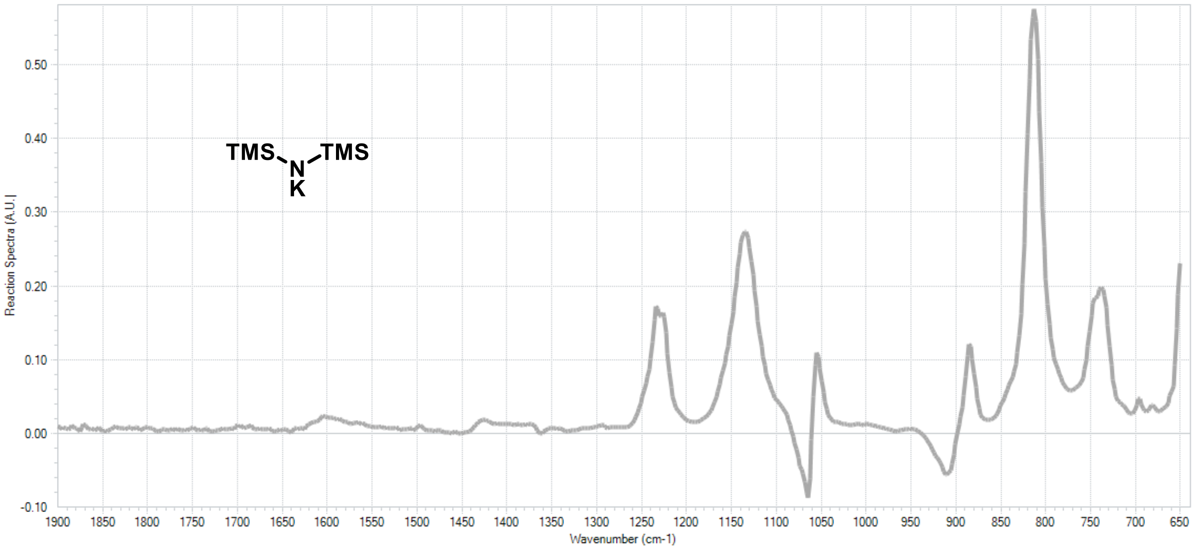

Figure S3. ReactIR spectrum of a 0.33 M solution of KHMDS in THF.

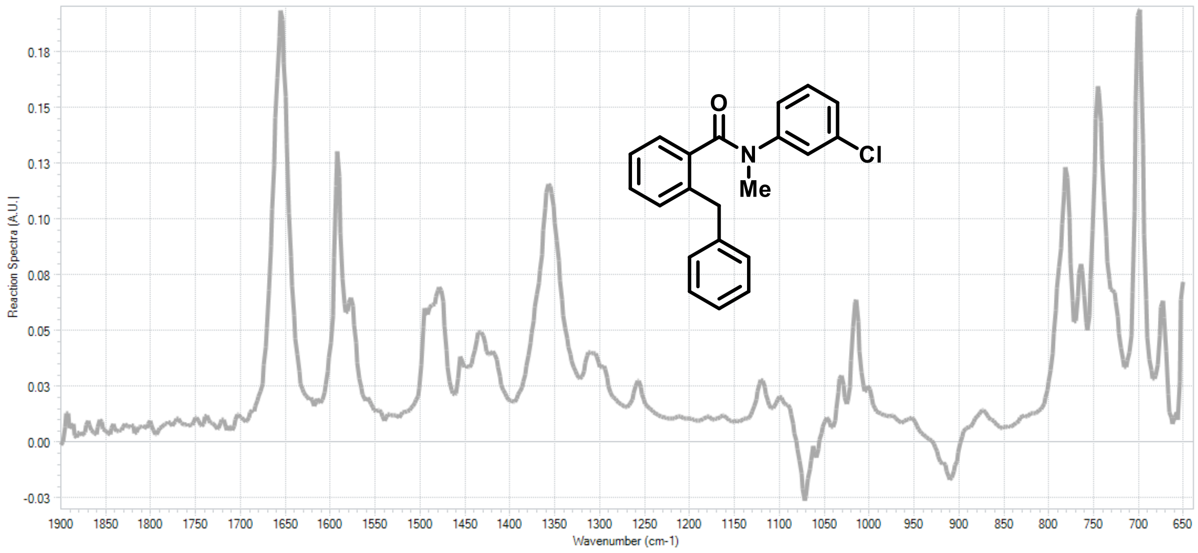

Figure S4. ReactIR spectrum of a 0.17 M solution of 2-benzyl-*N*-(3-chlorophenyl)-*N*-methylbenzamide (**3g**) in THF.

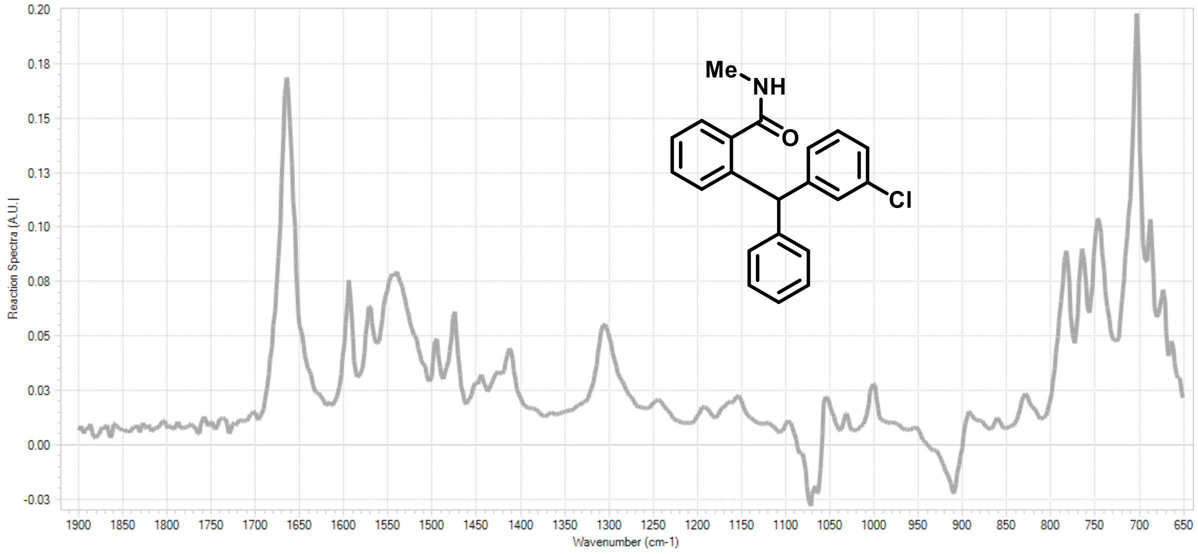

Figure S5. ReactIR spectrum of a 0.17 M solution of 2-((3-chlorophenyl)(phenyl)methyl)-*N*-methylbenzamide (**4g**) in THF.

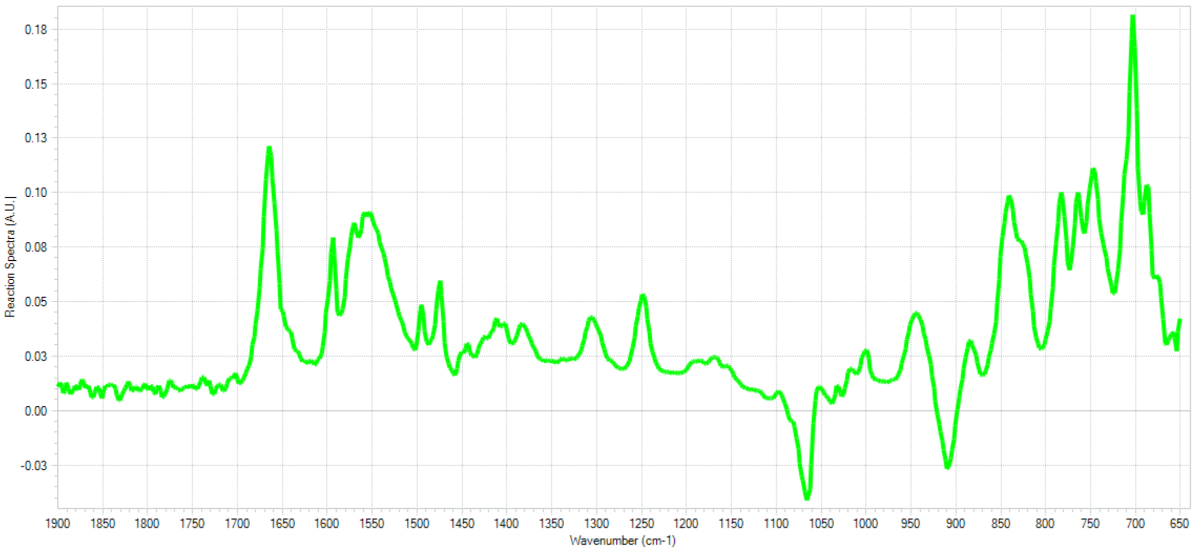

Figure S6. ReactIR spectrum of a 0.17 M solution of 2-((3-chlorophenyl)(phenyl)methyl)-*N*-methylbenzamide (**4g**) (0.25 mmol) in THF plus 0.1 mL of 1 M KHMDS. Note – analysed solution is colourless.

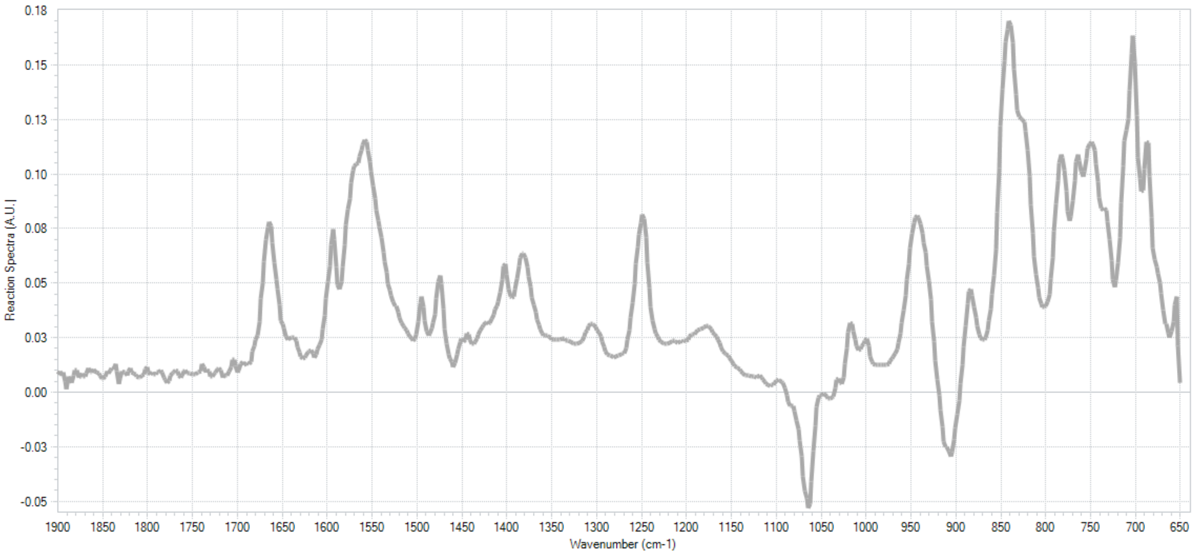

Figure S7. ReactIR spectrum of a 0.17 M solution of 2-((3-chlorophenyl)(phenyl)methyl)-*N*-methylbenzamide (**4g**) (0.25 mmol) in THF plus 0.2 mL of 1 M KHMDS. Note – analysed solution is colourless.

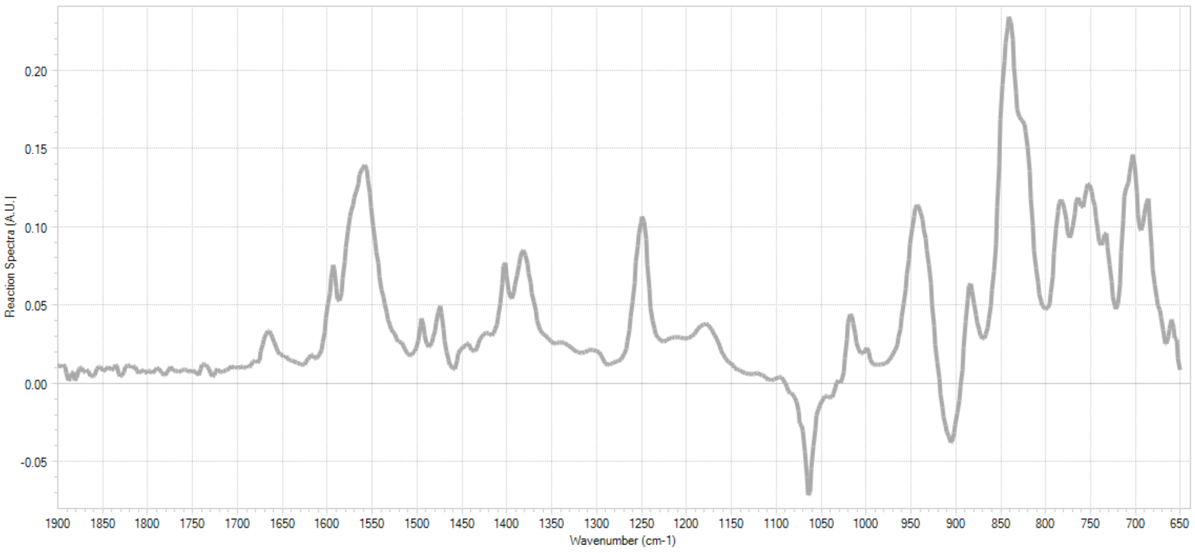

Figure S8. ReactIR spectrum of a 0.17 M solution of 2-((3-chlorophenyl)(phenyl)methyl)-*N*-methylbenzamide (**4g**) (0.25 mmol) in THF plus 0.3 mL of 1 M KHMDS. Note – analysed solution is colourless.

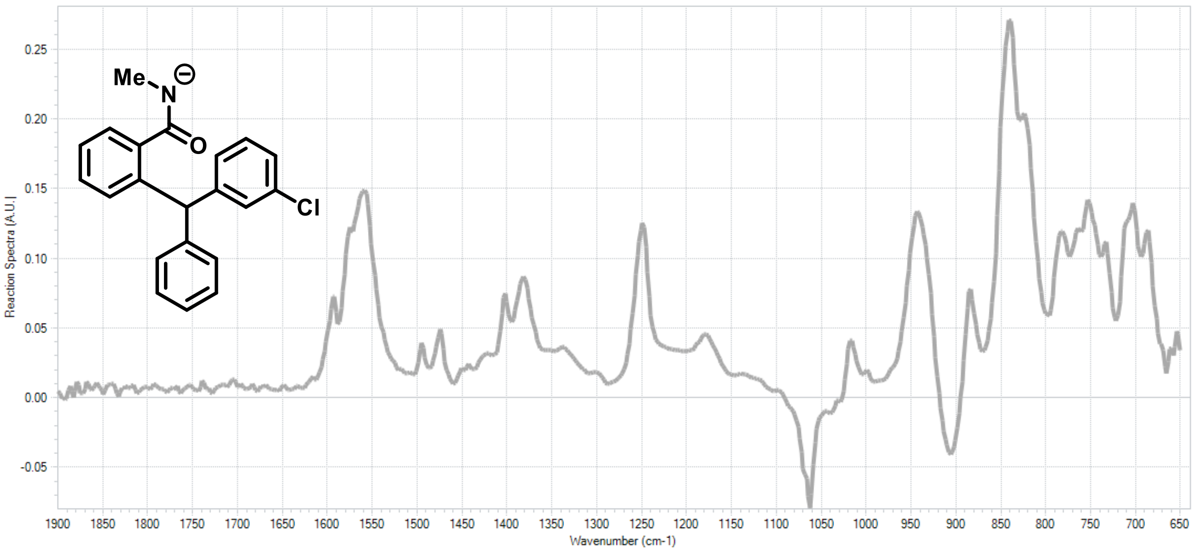

Figure S9. ReactIR spectrum of a 0.17 M solution of 2-((3-chlorophenyl)(phenyl)methyl)-*N*-methylbenzamide (**4g**) (0.25 mmol) in THF plus 0.4 mL of 1 M KHMDS. Note – analysed solution is deep red.

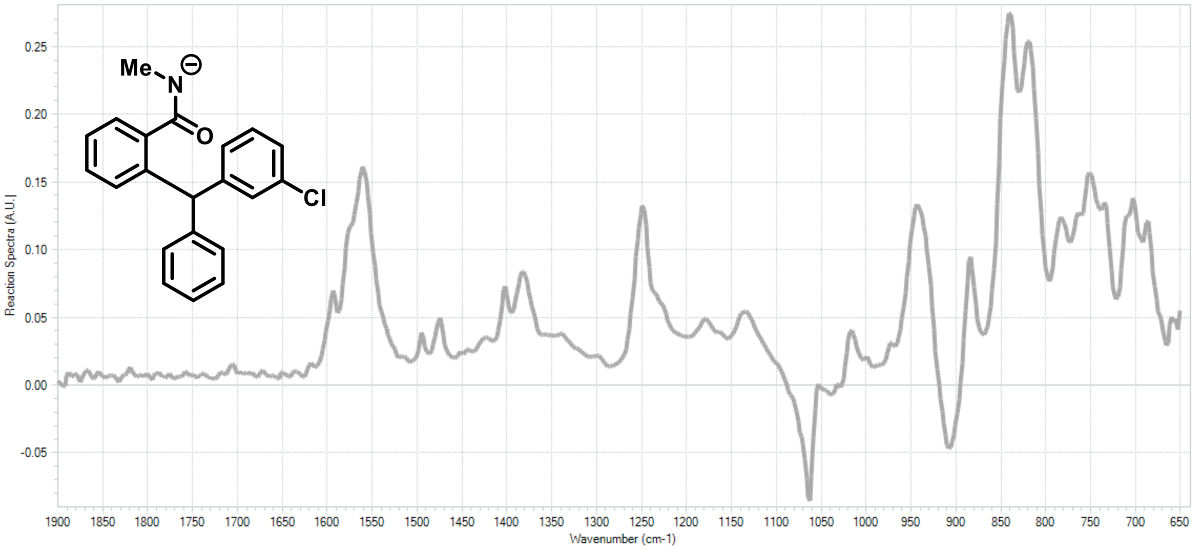

Figure S10. IR spectrum of a 0.17 M solution of 2-((3-chlorophenyl)(phenyl)methyl)-*N*-methylbenzamide (**4g**) (0.25 mmol) in THF plus 0.5 mL of 1 M KHMDS. Note – analysed solution is deep red.

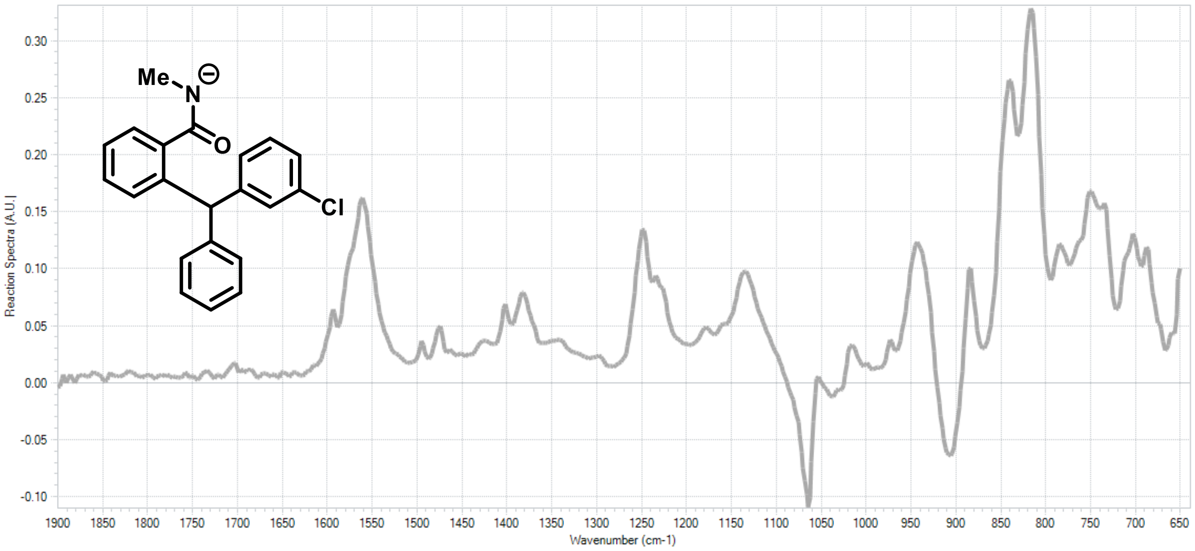

Figure S11. IR spectrum of a 0.17 M solution of 2-((3-chlorophenyl)(phenyl)methyl)-*N*-methylbenzamide (**4g**) (0.25 mmol) in THF plus 0.6 mL of 1 M KHMDS. Note – analysed solution is deep red.

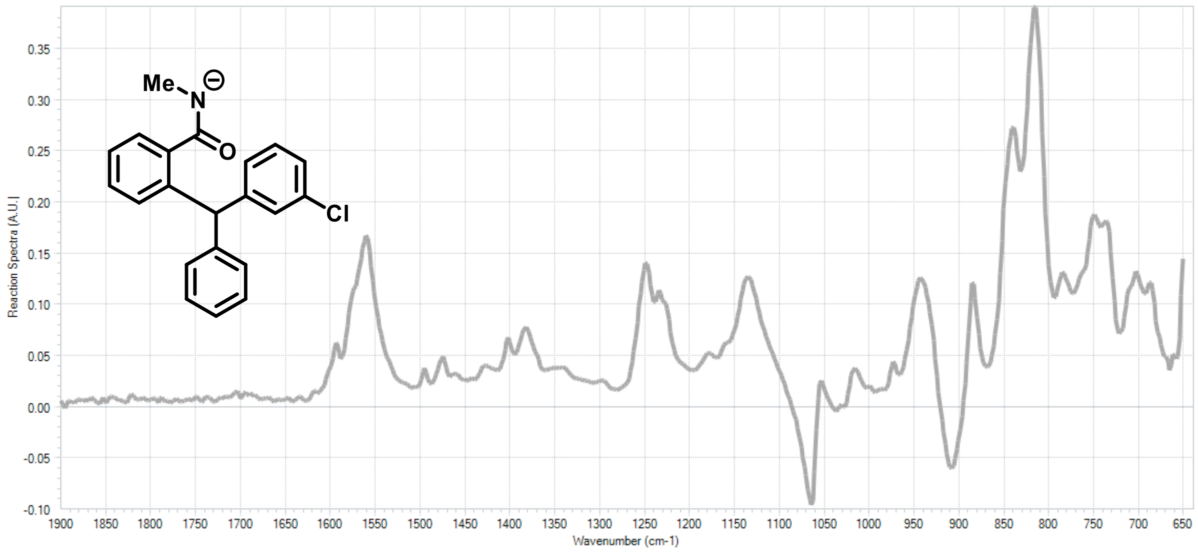

Figure S12. IR spectrum of a 0.17 M solution of 2-((3-chlorophenyl)(phenyl)methyl)-*N*-methylbenzamide (**4g**) (0.25 mmol) in THF plus 0.7 mL of 1 M KHMDS. Note – analysed solution is deep red.

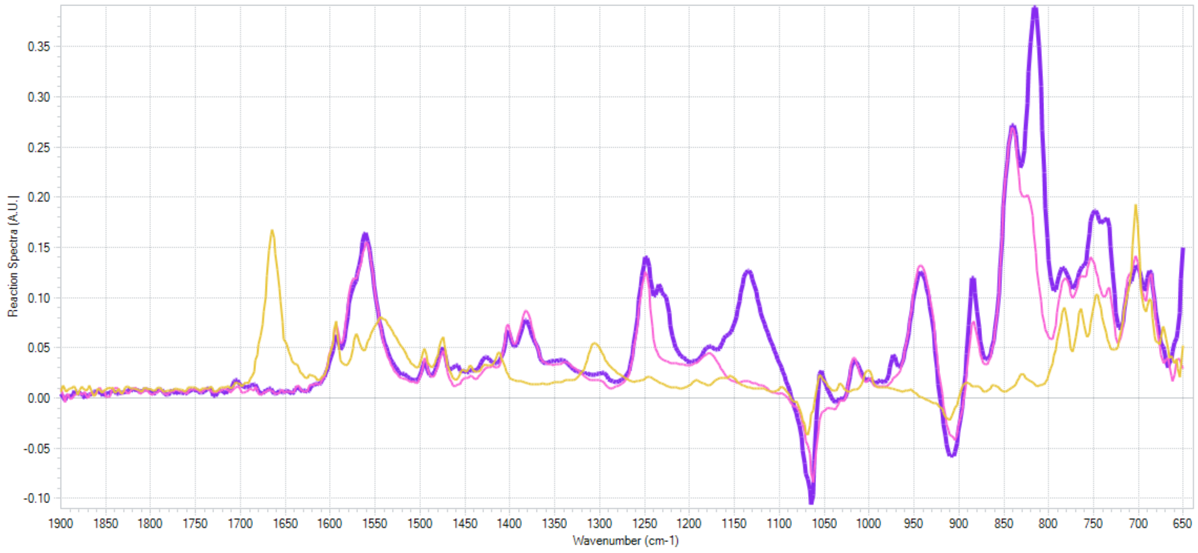

Figure S13. Overlayed spectra of Figure S5 (Light Brown), Figure S9 (Pink) and Figure S12 (purple).

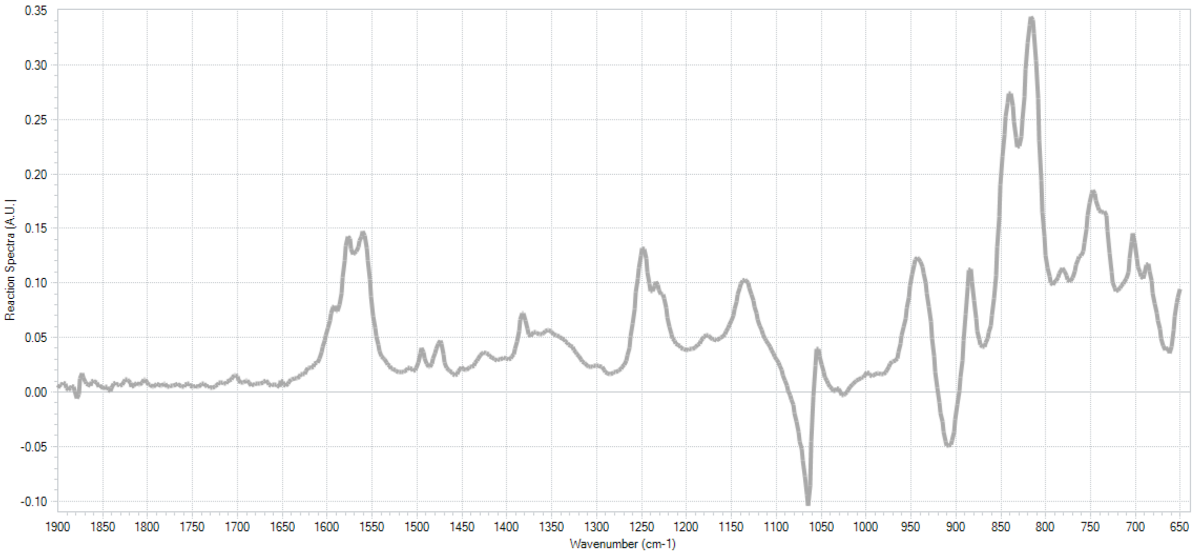

Figure S14. ReactIR spectrum of a complete reaction mixture prepared by exposing KHMDS to 2-benzyl-*N*-(3-chlorophenyl)-*N*-methylbenzamide (**3g**) in THF.

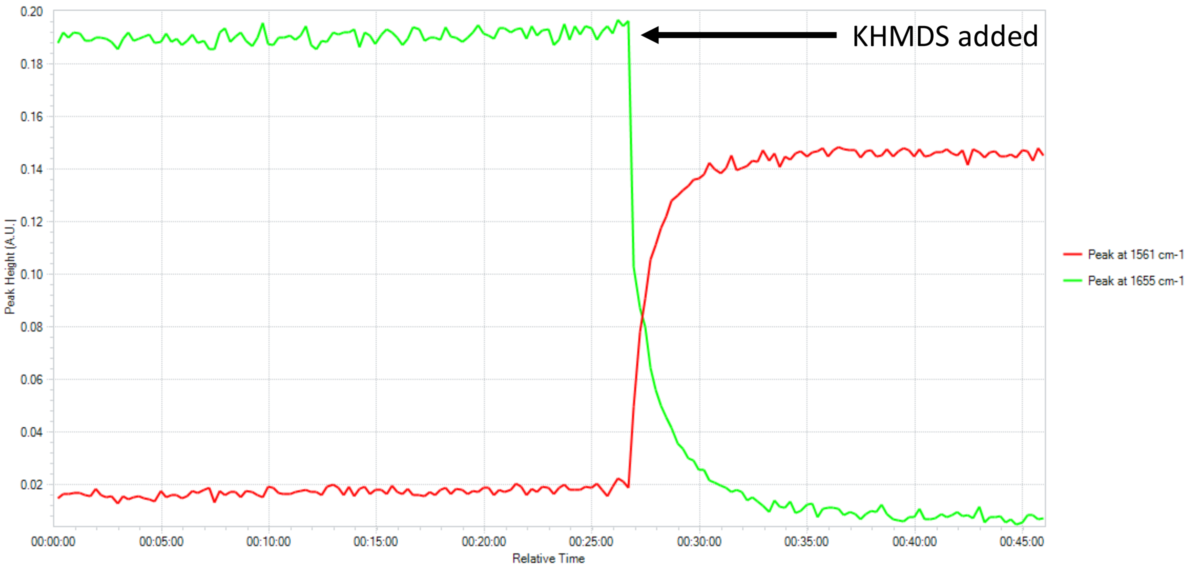

Figure S15. Reaction profile through monitoring the decay of signal at 1655 cm^{-1} (green) for 2-benzyl-*N*-(3-chlorophenyl)-*N*-methylbenzamide (**3g**) and formation of the signal at 1561 cm^{-1} (red) for 2-((3-chlorophenyl)(phenyl)methyl)-*N*-methylbenzamide (**4g**).

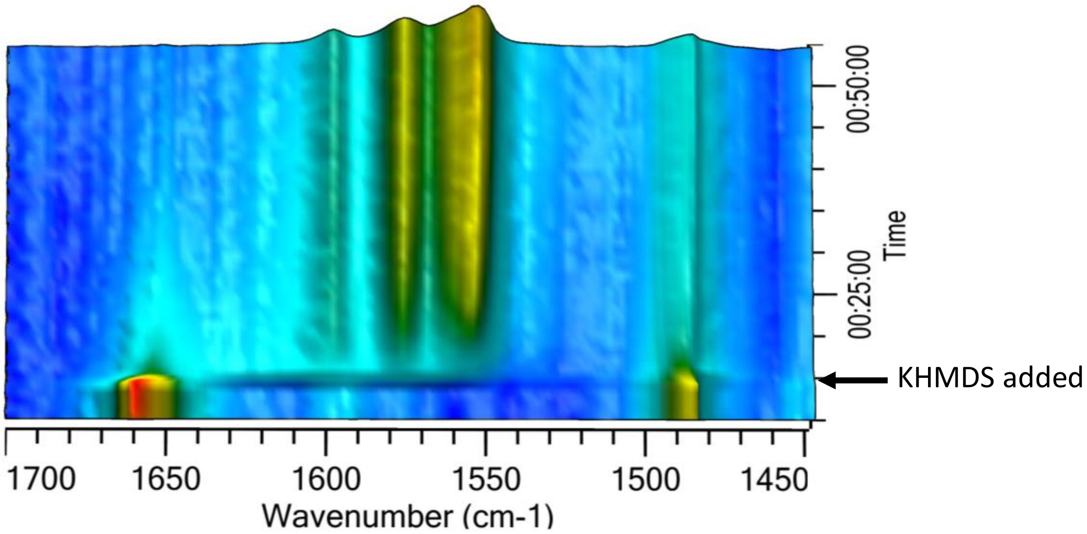

Figure S16. Aerial view of the surface reaction profile for the aryl migration of 2-benzyl-*N*-(4-chlorophenyl)-*N*-methylbenzamide (**3h**). Note – Direct conversion of starting material to product is observed, with no discernable indication of generation of an intermediate.

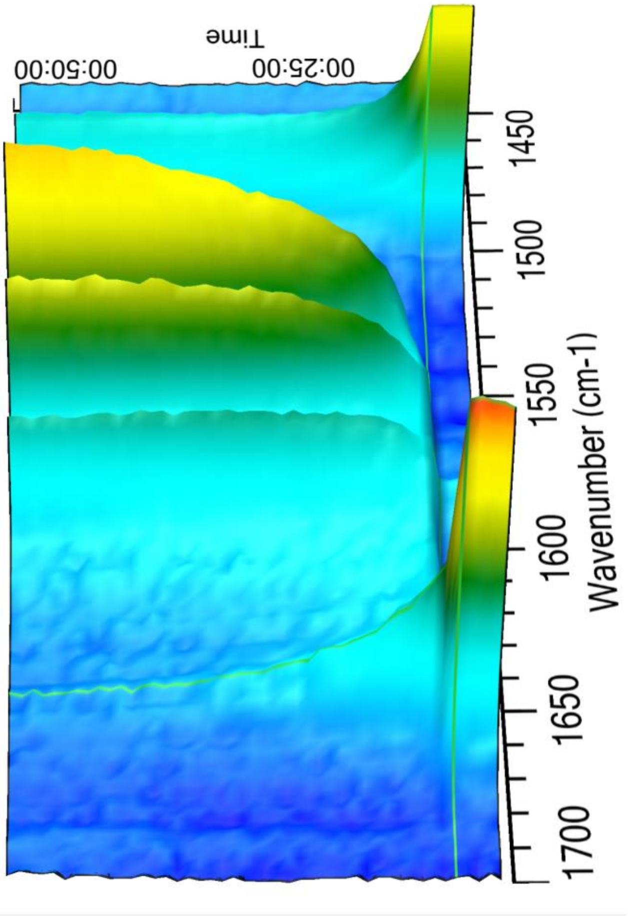

Figure S17. Side view of the surface reaction profile for the aryl migration of 2-benzyl-*N*-(4-chlorophenyl)-*N*-methylbenzamide (**3h**). Note – Direct conversion of starting material to product is observed, with no discernable generation of an intermediate.

Data analysis

For each substrate (with the exception of **3b**) a plot of ln($[P]_\infty-[P]_t$) vs. time for growth of the product anion peak at ca. 1560 cm^{-1} was constructed. For **3b** a plot of ln($[S]_t-[S]_{final}$) vs. time for the decay of the *N*-arylbenzamide signal at ca. 1660 cm^{-1} was used, as this gave more reliable data owing to the exceptionally slow nature of the reaction. The straight-line section of these curves indicated a period of first-order reactivity with the gradient of the line of best fit providing the rate constant k_{obs}. Straight-line plots were also attempted from the intergrated 2nd order rate equation by plotting $1/([P]_\infty-[P]_t)$ vs. time, but this gave poor agreement with the fitted regression line. A Hammett plot of log(k_R/k_H) vs. σ^- was constructed, the gradient of which (ρ = +4.0) is consistent with charge build up on the aryl substituent undergoing rearrangement.

2-Benzyl-*N*-methyl-*N*-(pyridin-3-yl)benzamide (3k) - Run 1

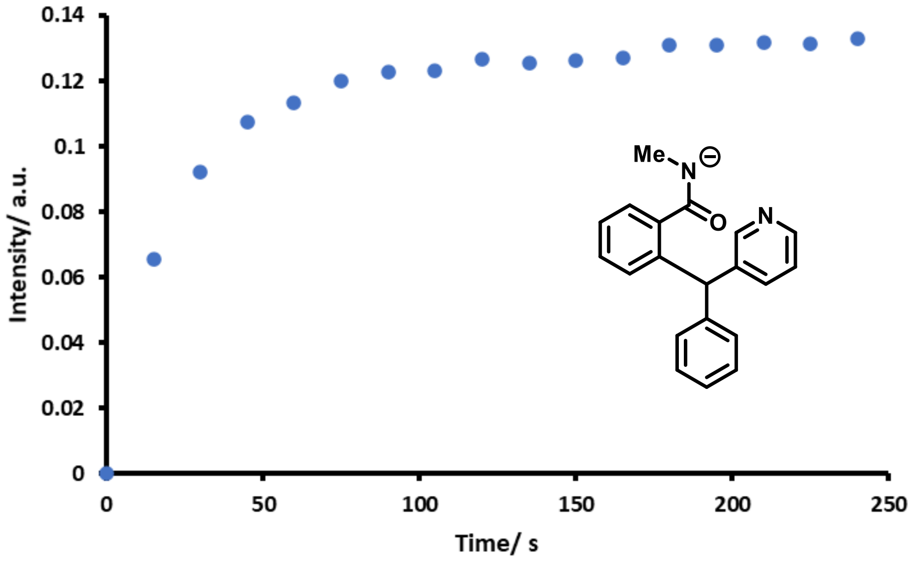

Figure S18. Reaction profile for aryl migration of 2-benzyl-*N*-methyl-*N*-(pyridin-3-yl)benzamide (**3k**) by monitoring formation of signal at 1561 cm^{-1}.

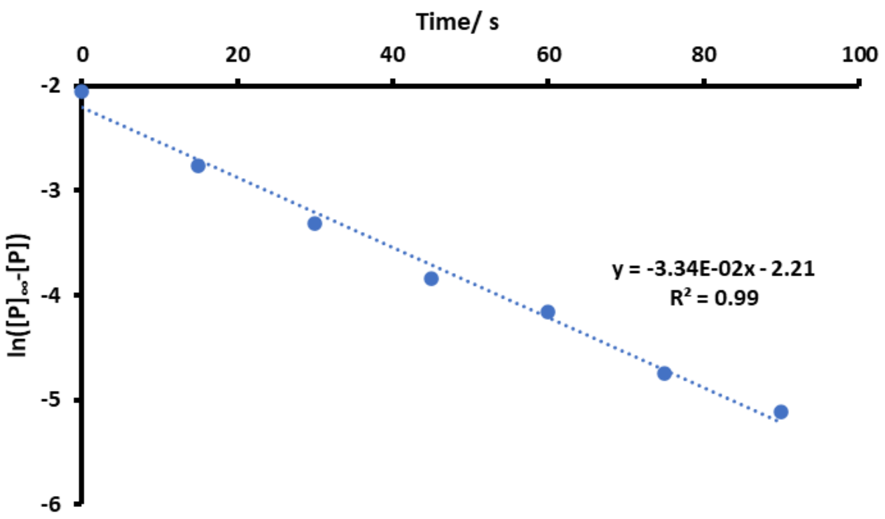

Figure S19. Straight-line plot by using the integrated first order rate law when using data from Figure S18.

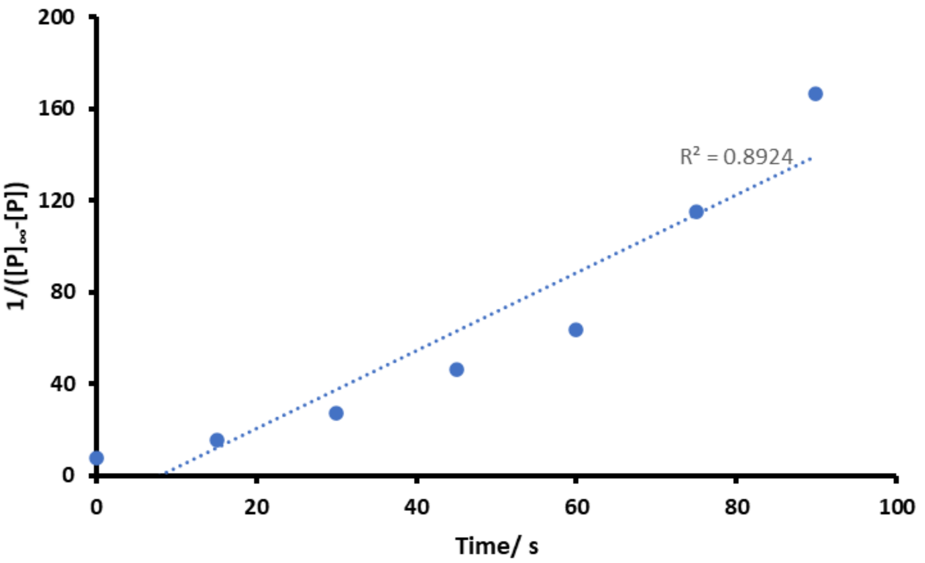

Figure S20. Attempted straight-line plot by using the integrated second order rate law when using data from Figure S18.

Run 2

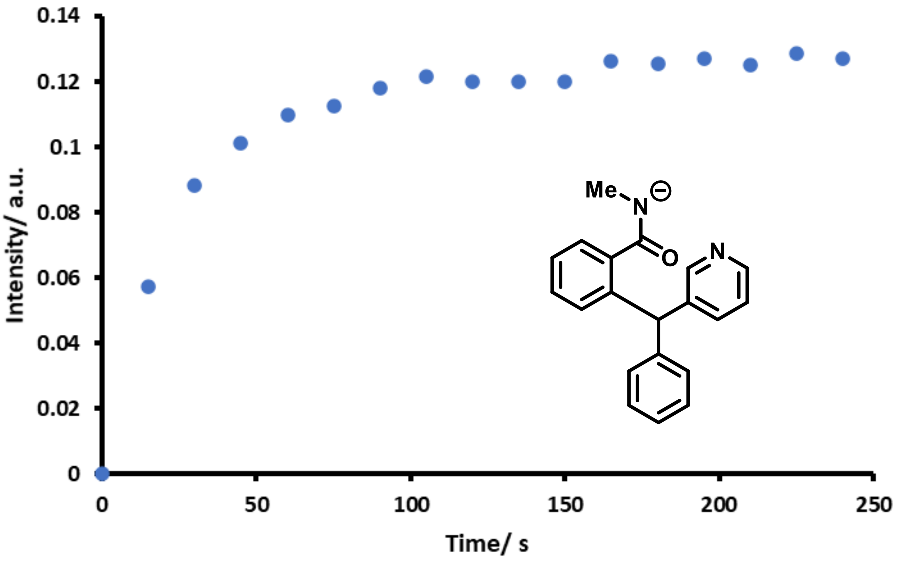

Figure S21. Reaction profile for aryl migration of 2-benzyl-*N*-methyl-*N*-(pyridin-3-yl)benzamide (**3k**) by monitoring formation of signal at 1559 cm^{-1}.

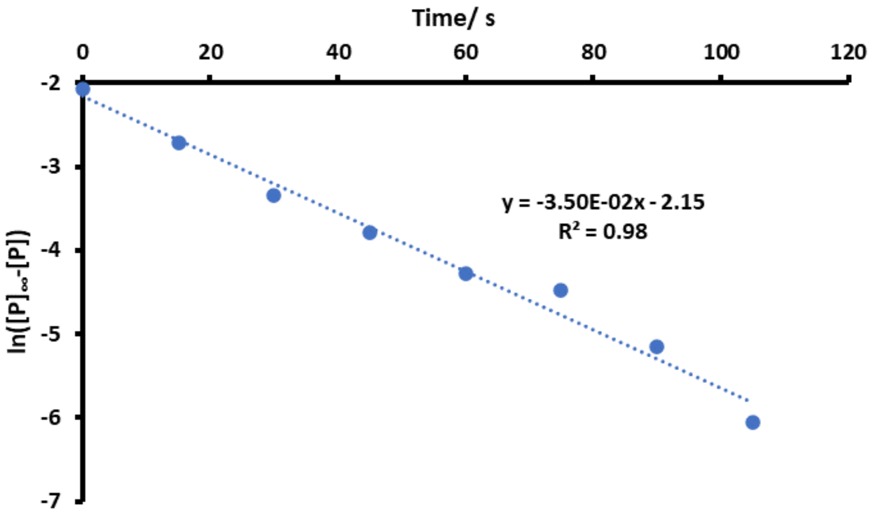

Figure S22. Straight-line plot by using the integrated first order rate law when using data from Figure S21.

Run 3

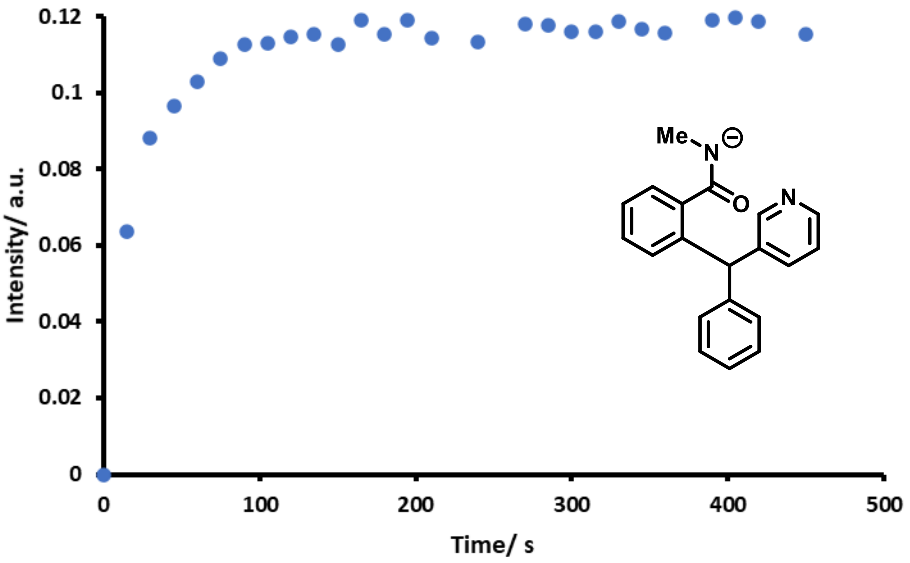

Figure S23. Reaction profile for aryl migration of 2-benzyl-*N*-methyl-*N*-(pyridin-3-yl)benzamide (**3k**) by monitoring formation of signal at 1561 cm^{-1}.

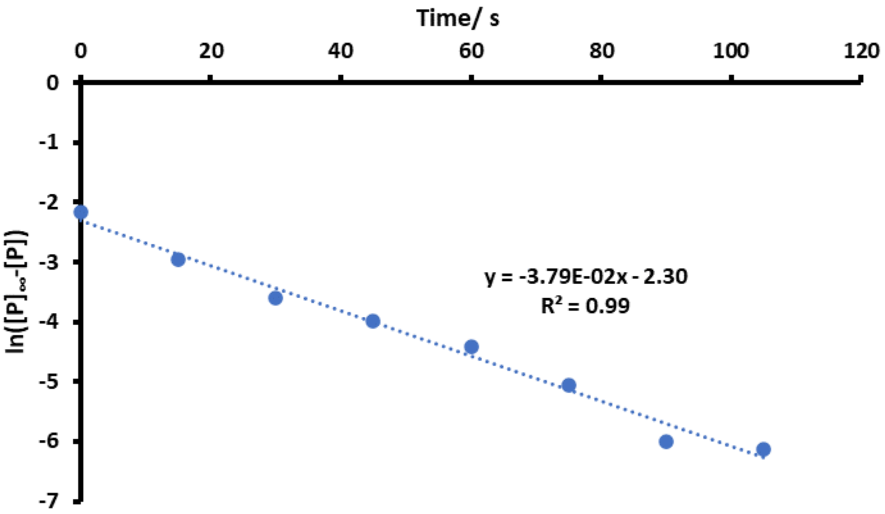

Figure S24. Straight-line plot by using the integrated first order rate law when using data from Figure S23.

Average k_{obs} over three run = 3.54 x 10^{-2} s^{-1}.

2-Benzyl-*N*-methyl-*N*-(pyridin-2-yl)benzamide (3l) - Run 1

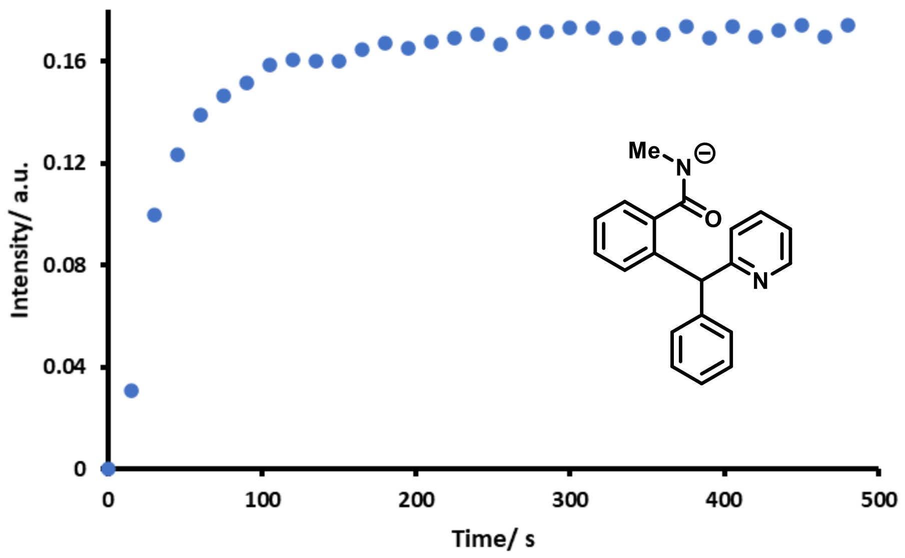

Figure S25. Reaction profile for aryl migration of 2-benzyl-*N*-methyl-*N*-(pyridin-2-yl)benzamide (**3l**) by monitoring formation of signal at 1571 cm^{-1}.

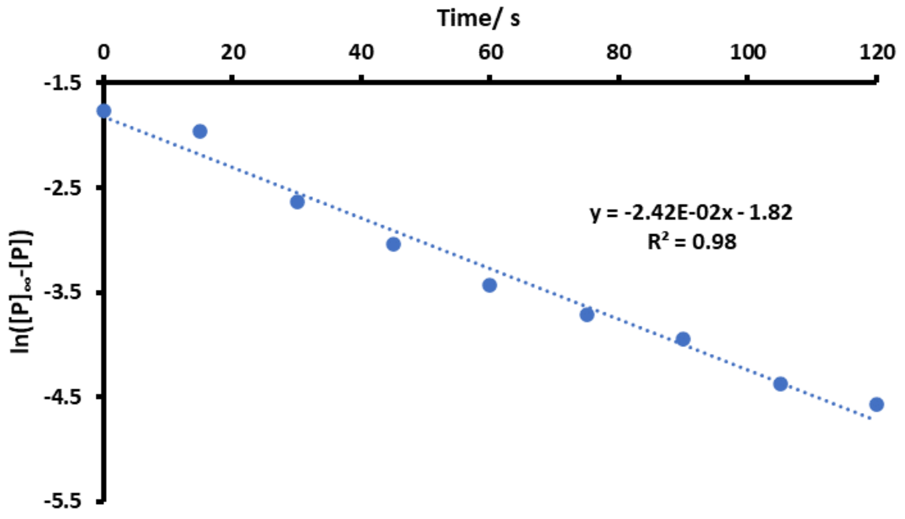

Figure S26. Straight-line plot by using the integrated first order rate law when using data from Figure S25.

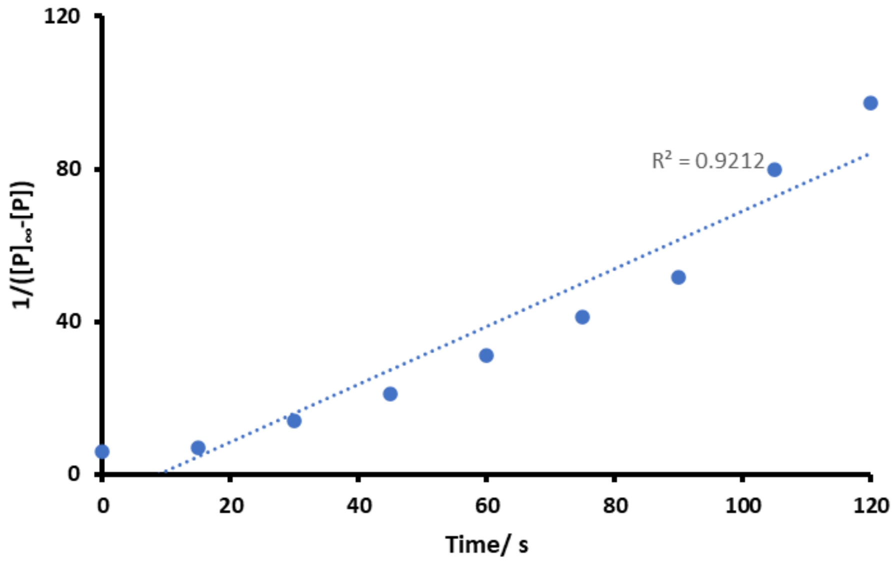

Figure S27. Attempted straight-line plot by using the integrated second order rate law when using data from Figure S25.

<u>Run 2</u>

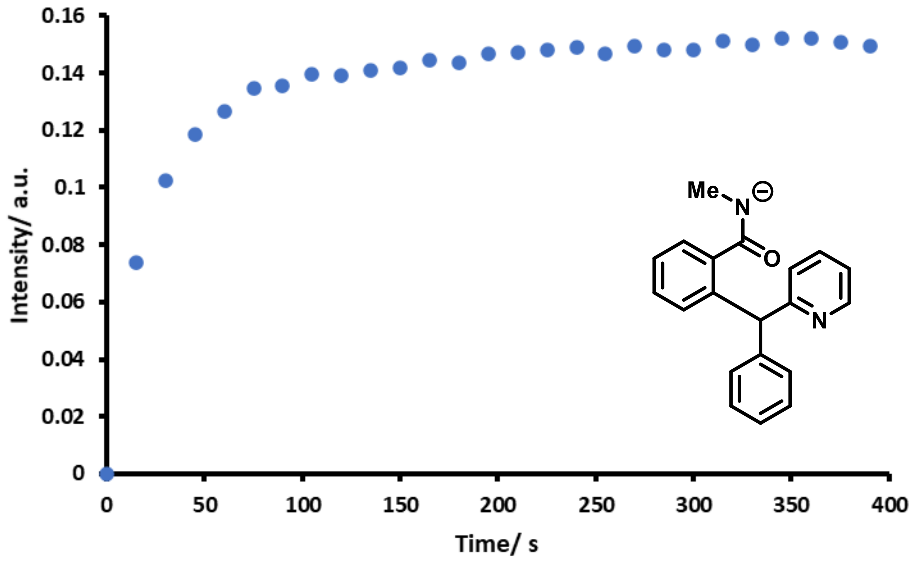

Figure S28. Reaction profile for aryl migration of 2-benzyl-*N*-methyl-*N*-(pyridin-2-yl)benzamide (**3l**) by monitoring formation of signal at 1572 cm^{-1}.

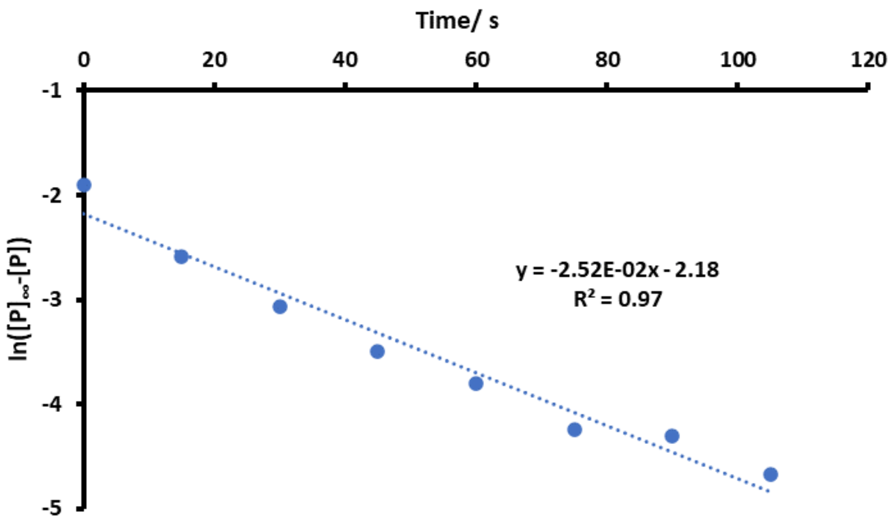

Figure S29. Straight-line plot by using the integrated first order rate law when using data from Figure S28.

<u>**Run 3**</u>

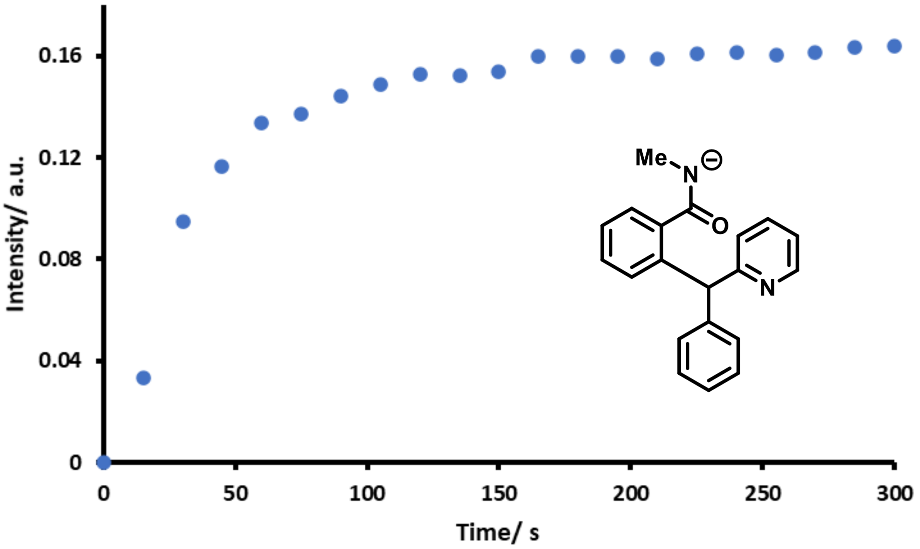

Figure S30. Reaction profile for aryl migration of 2-benzyl-*N*-methyl-*N*-(pyridin-2-yl)benzamide (**3l**) by monitoring formation of signal at 1572 cm^{-1}.

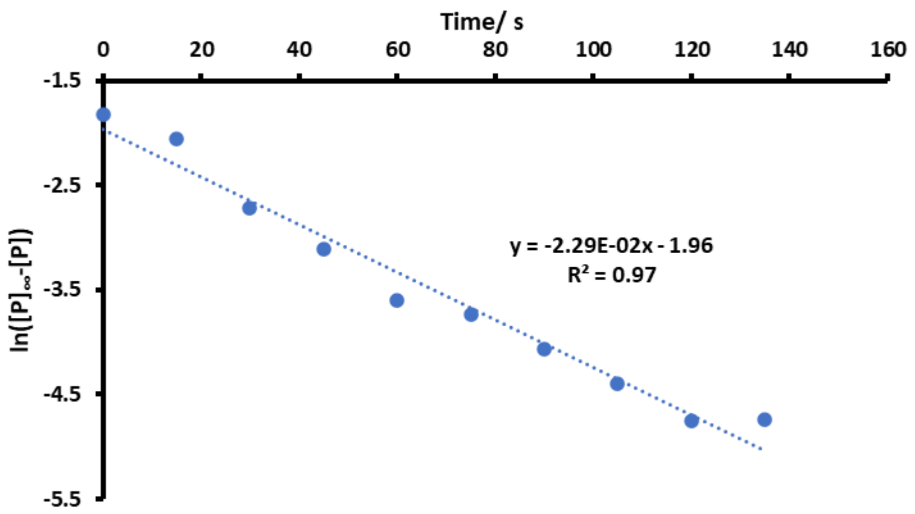

Figure S31. Straight-line plot by using the integrated first order rate law when using data from Figure S30.

Average k_{obs} over three run = 2.41 x 10^{-2} s^{-1}.

2-Benzyl-*N*-(3-chlorophenyl)-*N*-methylbenzamide (3g) - Run 1

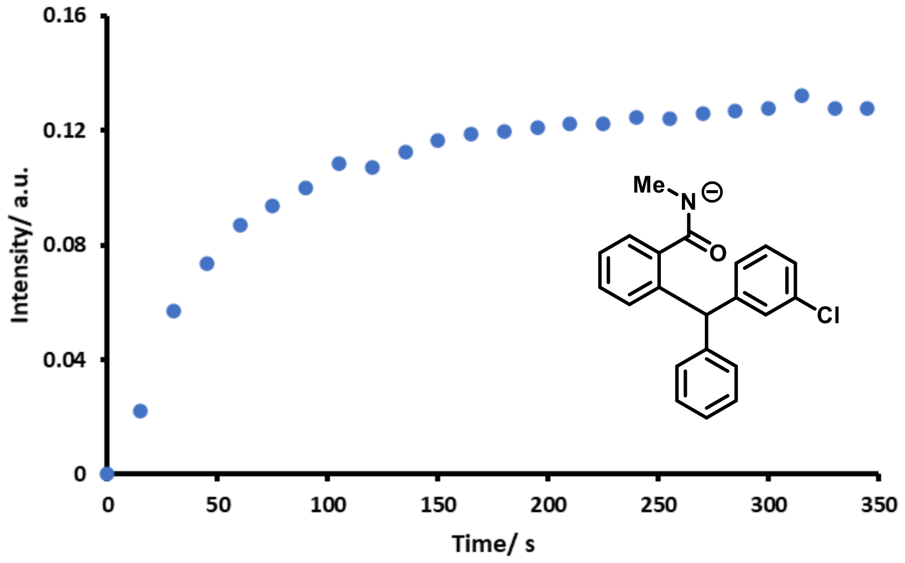

Figure S32. Reaction profile for aryl migration of 2-benzyl-*N*-(3-chlorophenyl)-*N*-methylbenzamide (**3g**) by monitoring formation of signal at 1559 cm^{-1}.

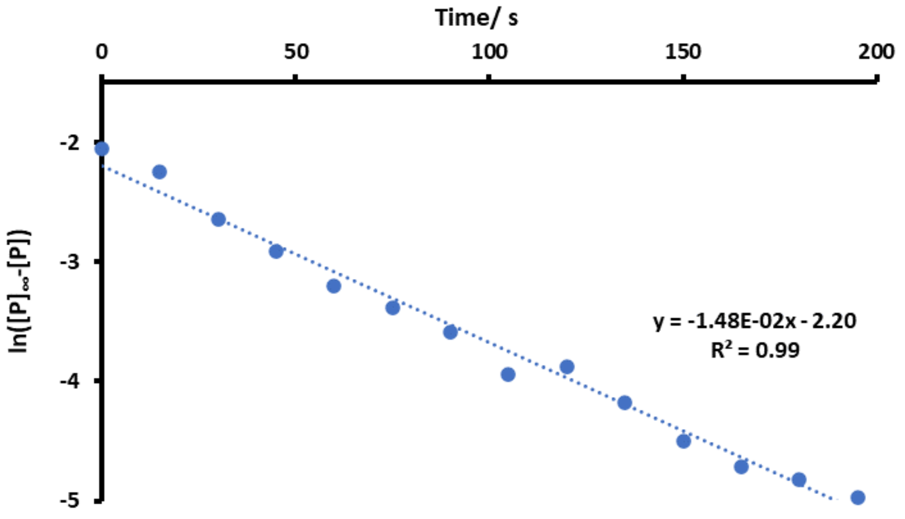

Figure S33. Straight-line plot by using the integrated first order rate law when using data from Figure S32.

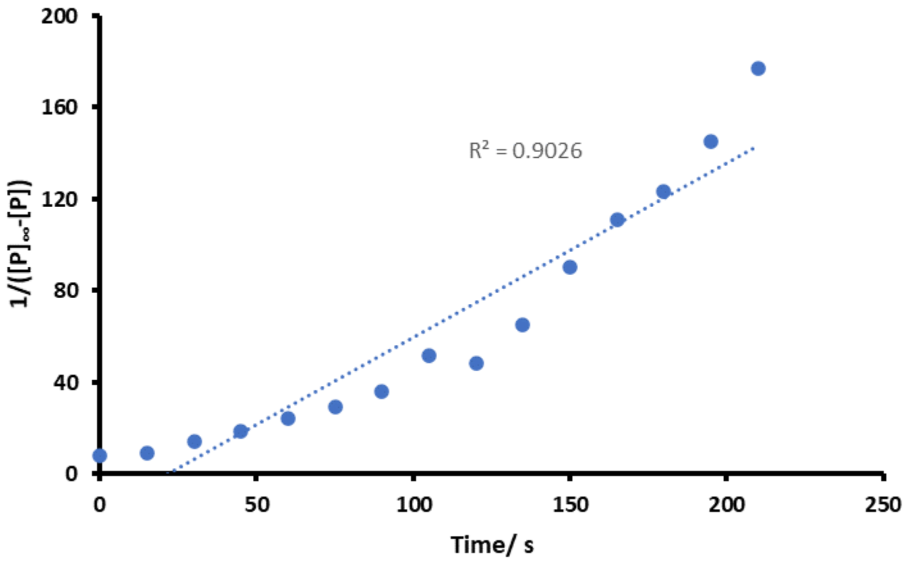

Figure S34. Attempted straight-line plot by using the integrated second order rate law when using data from Figure S32.

__Run 2__

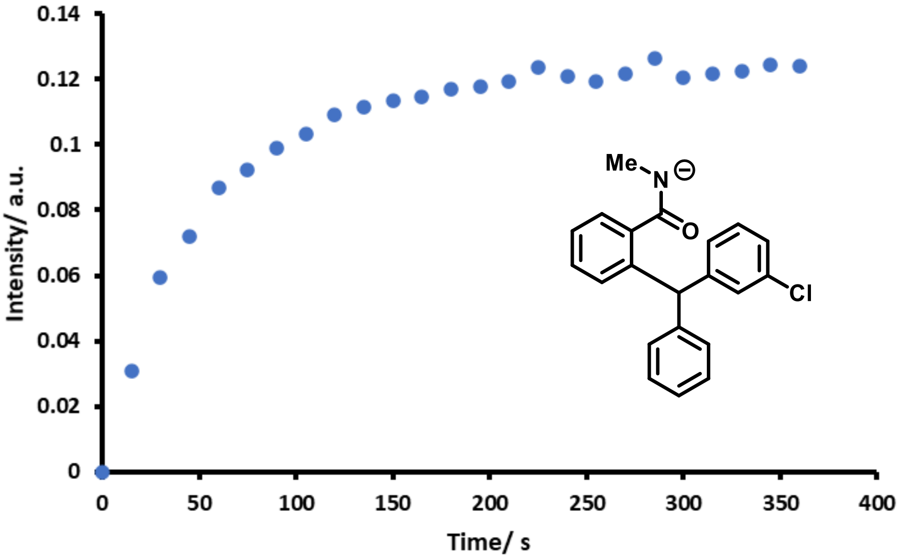

Figure S35. Reaction profile for aryl migration of 2-benzyl-*N*-(3-chlorophenyl)-*N*-methylbenzamide (**3g**) by monitoring formation of signal at 1560 cm^{-1}.

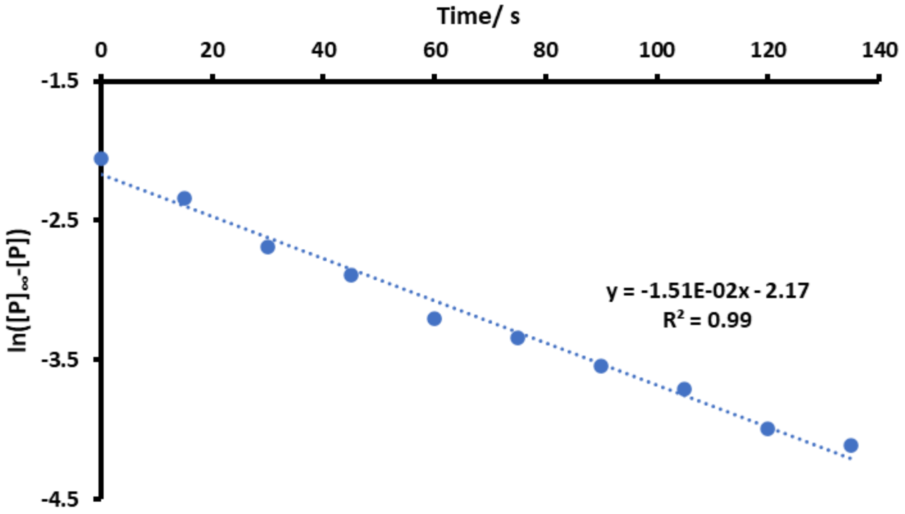

Figure S36. Straight-line plot by using the integrated first order rate law when using data from Figure S35.

<u>Run 3</u>

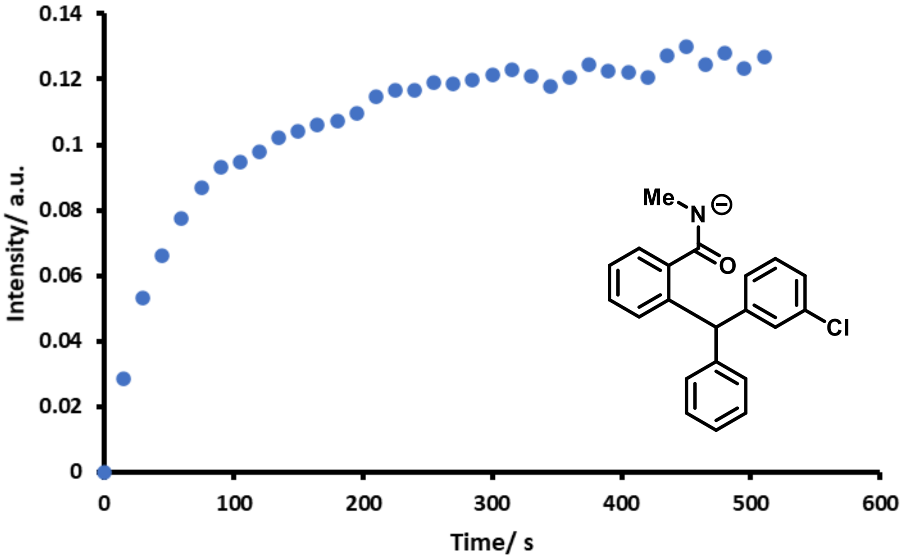

Figure S37. Reaction profile for aryl migration of 2-benzyl-*N*-(3-chlorophenyl)-*N*-methylbenzamide (**3g**) by monitoring formation of signal at 1559 cm^{-1}.

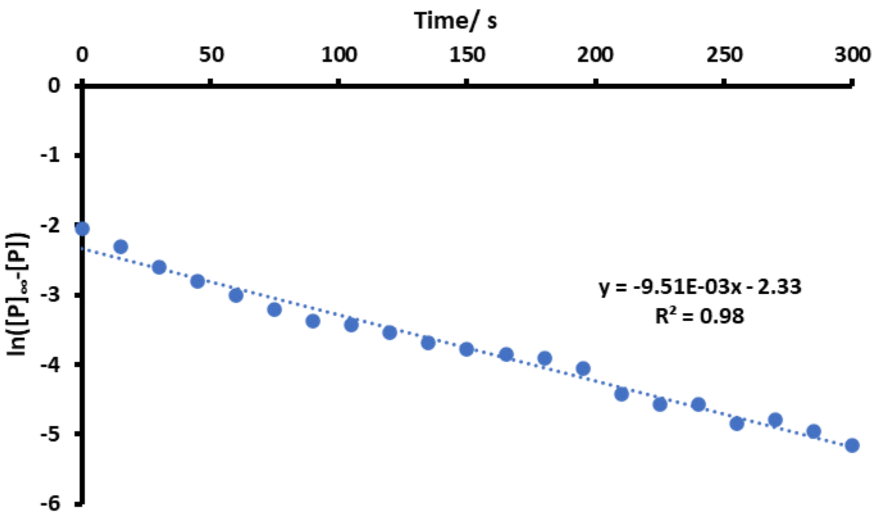

Figure S38. Straight-line plot by using the integrated first order rate law when using data from Figure S37.

Average k_{obs} over three run = 1.31 x 10^{-2} s^{-1}.

2-Benzyl-*N*-(4-bromophenyl)-*N*-methylbenzamide (3i) - Run 1

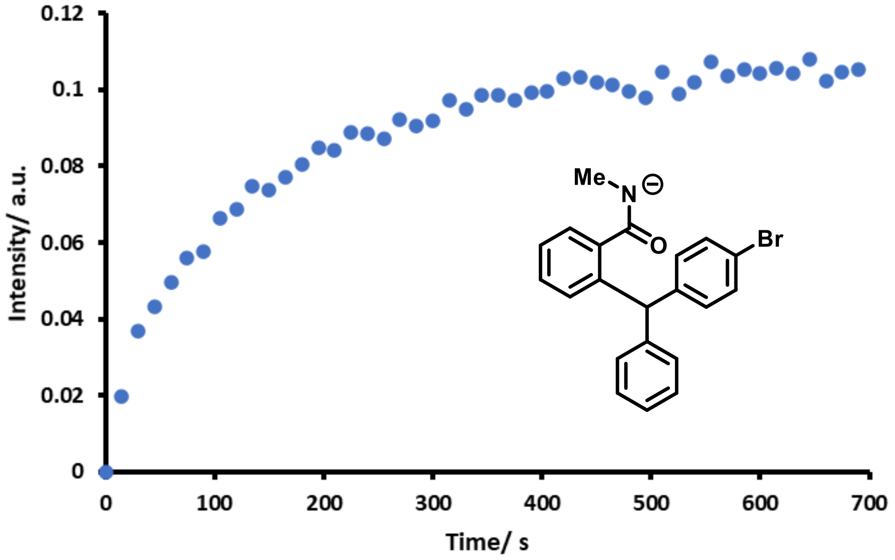

Figure S39. Reaction profile for aryl migration of 2-benzyl-*N*-(4-bromophenyl)-*N*-methylbenzamide (**3i**) by monitoring formation of signal at 1559 cm^{-1}.

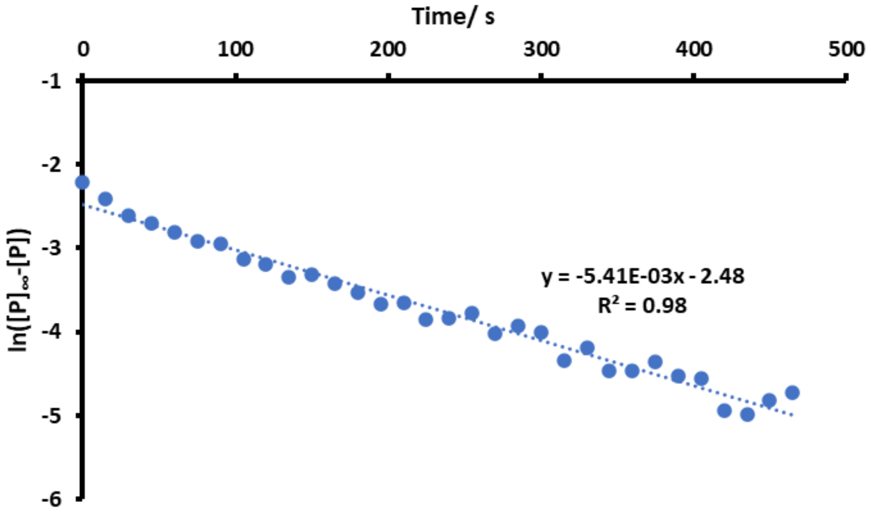

Figure S40. Straight-line plot by using the integrated first order rate law when using data from Figure S39.

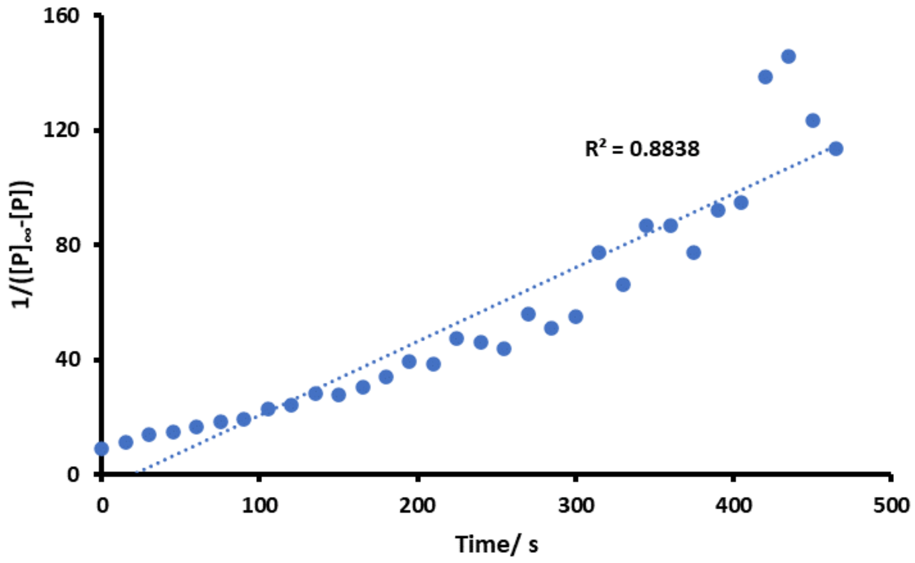

Figure S41. Attempted straight-line plot by using the integrated second order rate law when using data from Figure S39.

<u>**Run 2**</u>

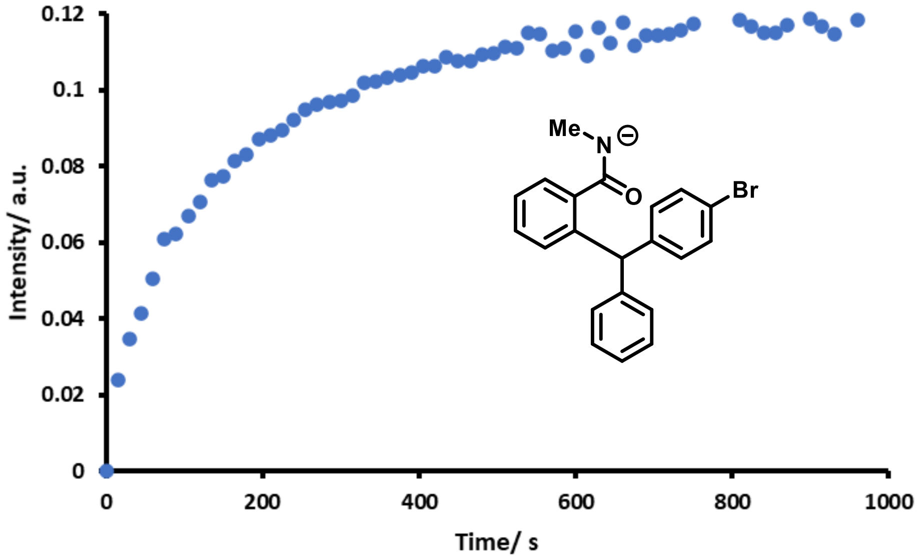

Figure S42. Reaction profile for aryl migration of 2-benzyl-*N*-(4-bromophenyl)-*N*-methylbenzamide (**3i**) by monitoring formation of signal at 1559 cm^{-1}.

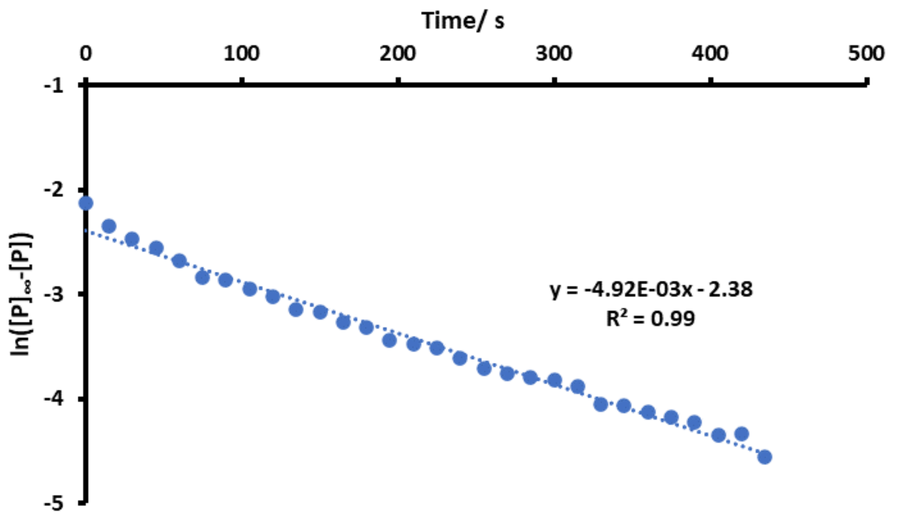

Figure S43. Straight-line plot by using the integrated first order rate law when using data from Figure S42.

Run 3

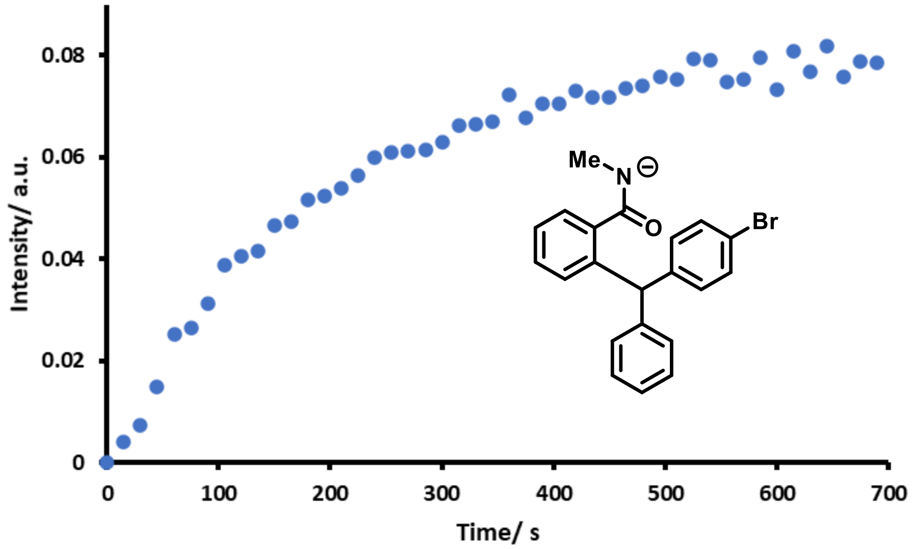

Figure S44. Reaction profile for aryl migration of 2-benzyl-*N*-(4-bromophenyl)-*N*-methylbenzamide (**3i**) by monitoring formation of signal at 1560 cm^{-1}.

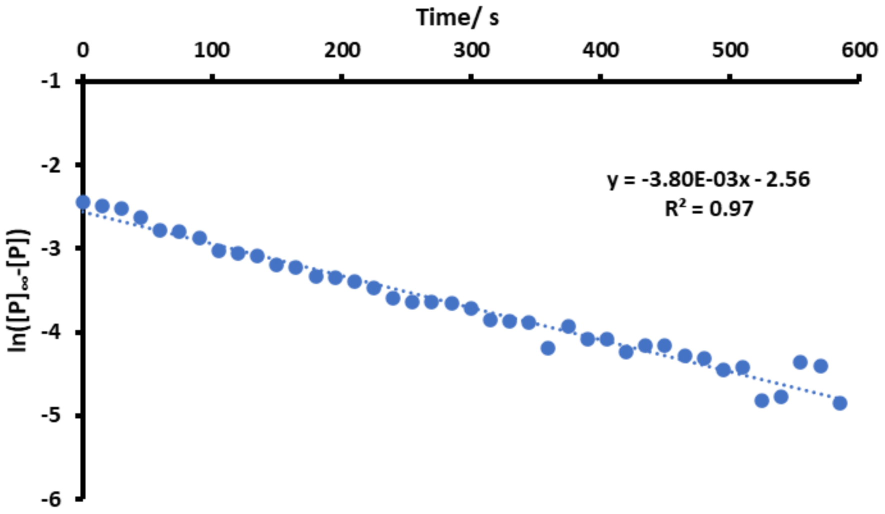

Figure S45. Straight-line plot by using the integrated first order rate law when using data from Figure S44.

Average k_{obs} over three run = 5.41 x 10^{-3} s^{-1}.

2-Benzyl-*N*-(4-chlorophenyl)-*N*-methylbenzamide (3h) - <u>Run 1</u>

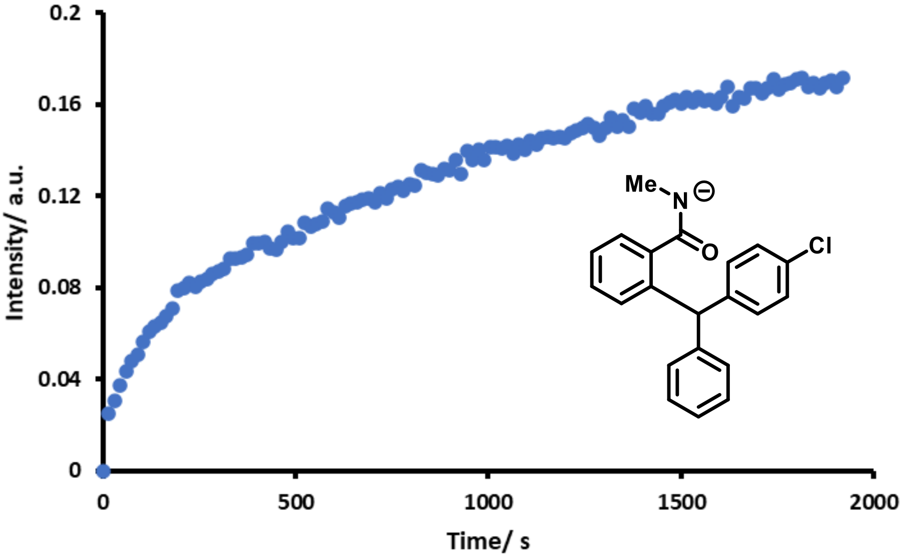

Figure S46. Reaction profile for aryl migration of 2-benzyl-*N*-(4-chlorophenyl)-*N*-methylbenzamide (**3h**) by monitoring formation of signal at 1560 cm^{-1}.

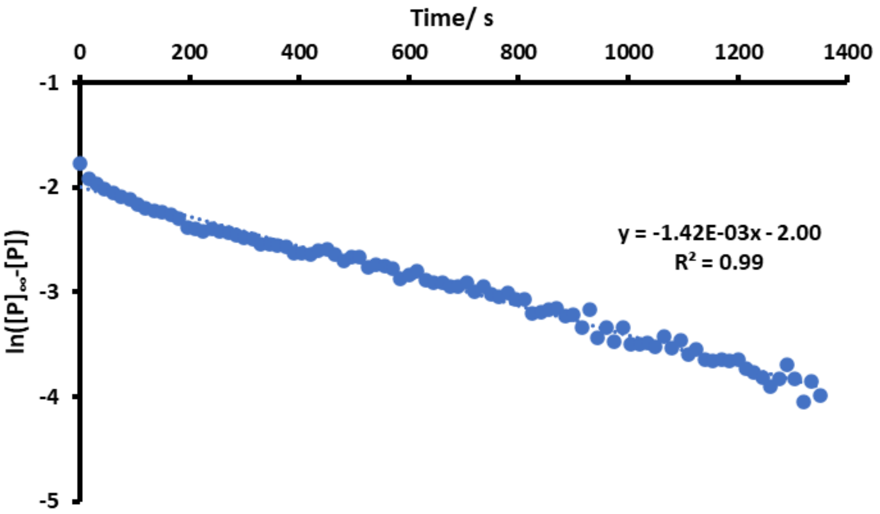

Figure S47. Straight-line plot by using the integrated first order rate law when using data from Figure S46.

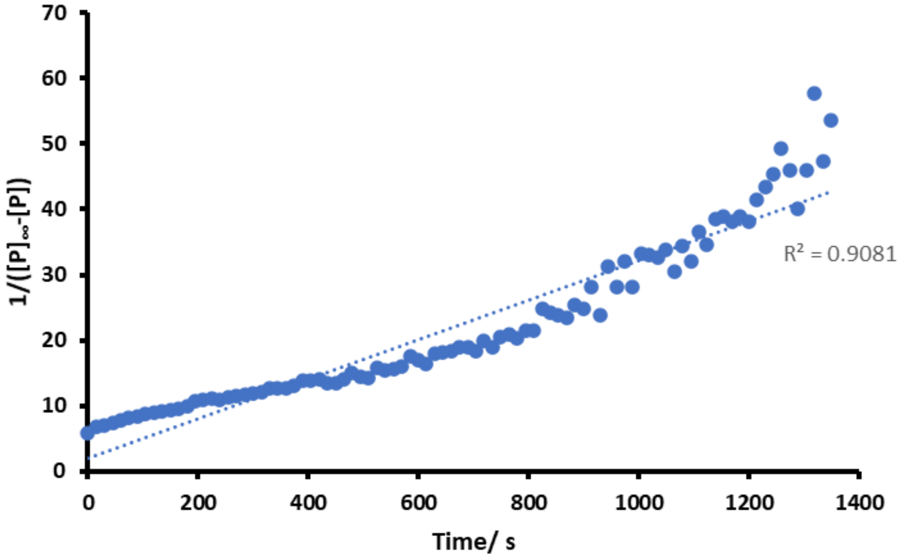

Figure S48. Attempted straight-line plot by using the integrated second order rate law when using data from Figure S46.

Run 2

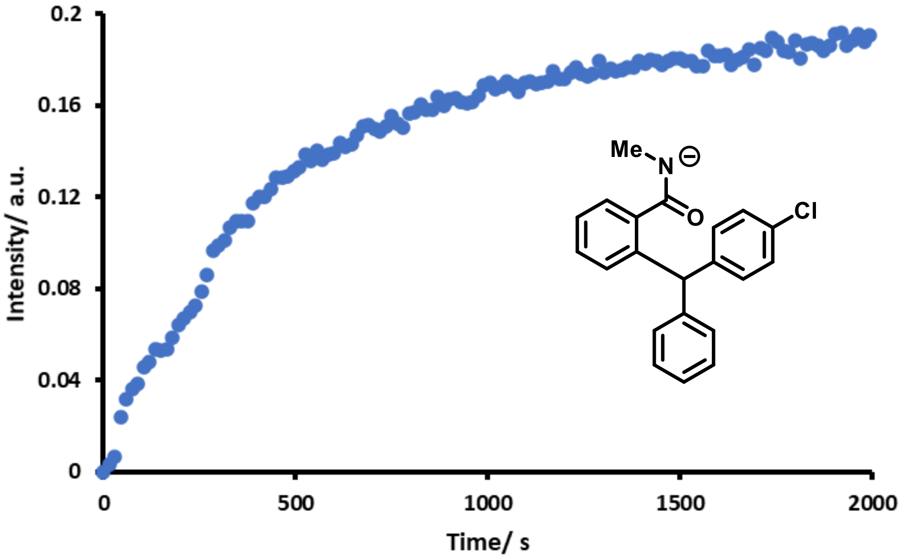

Figure S49. Reaction profile for aryl migration of 2-benzyl-*N*-(4-chlorophenyl)-*N*-methylbenzamide (**3h**) by monitoring formation of signal at 1558 cm^{-1}.

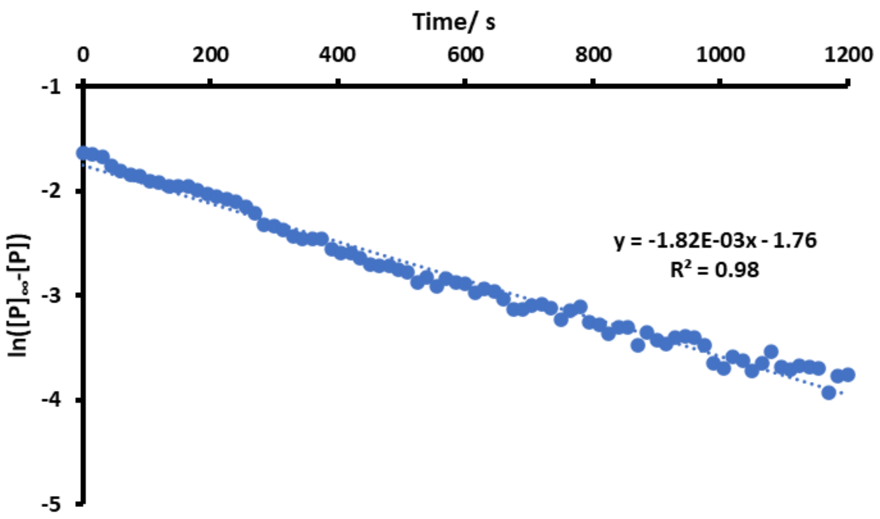

Figure S50. Straight-line plot by using the integrated first order rate law when using data from Figure S49.

<u>**Run 3**</u>

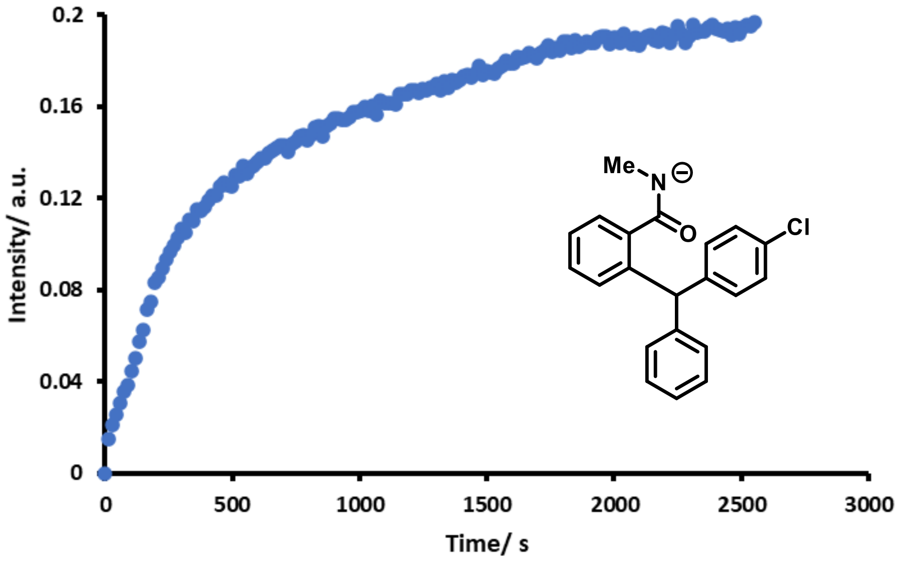

Figure S51. Reaction profile for aryl migration of 2-benzyl-*N*-(4-chlorophenyl)-*N*-methylbenzamide (**3h**) by monitoring formation of signal at 1557 cm^{-1}.

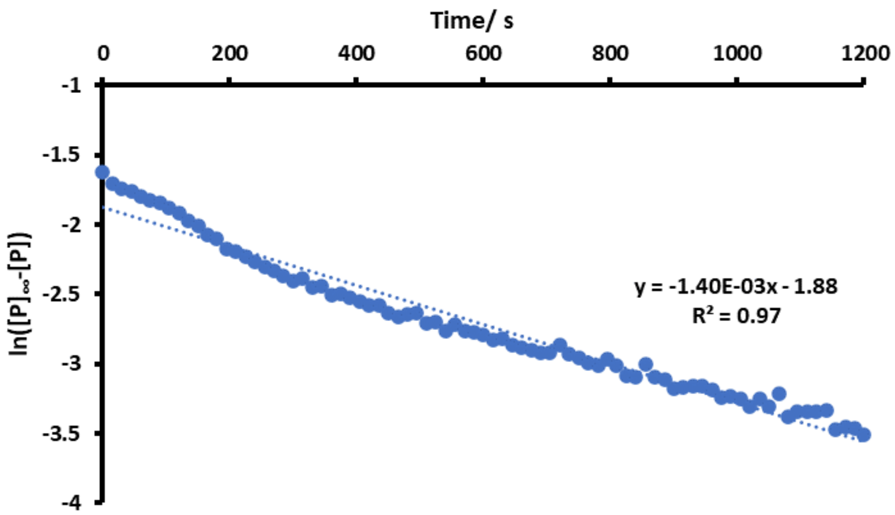

Figure S52. Straight-line plot by using the integrated first order rate law when using data from Figure S51.

Average k_{obs} over three run = 1.55 x 10^{-3} s^{-1}.

2-Benzyl-*N*-methyl-*N*-phenylbenzamide (1) - <u>Run 1</u>

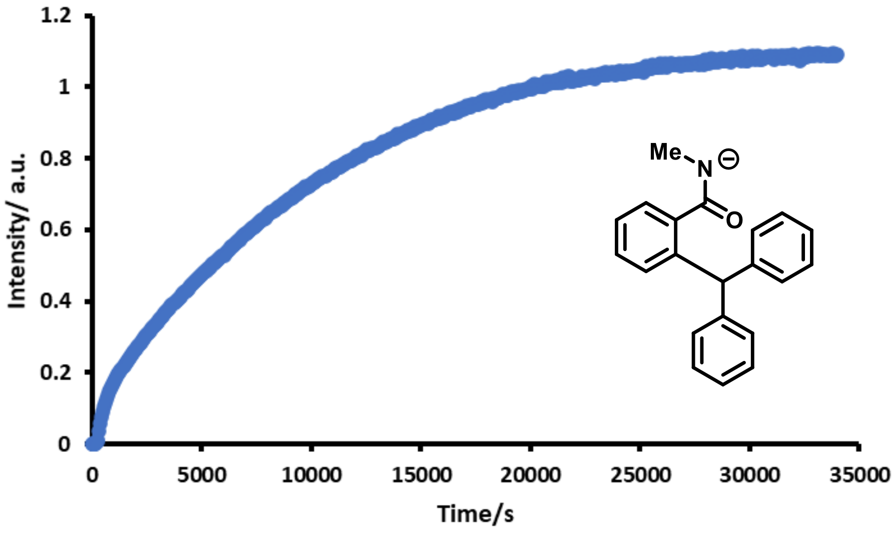

Figure S53. Reaction profile for aryl migration of 2-benzyl-*N*-methyl-*N*-phenylbenzamide (**1**) by monitoring formation of signal at 1552 cm^{-1}.

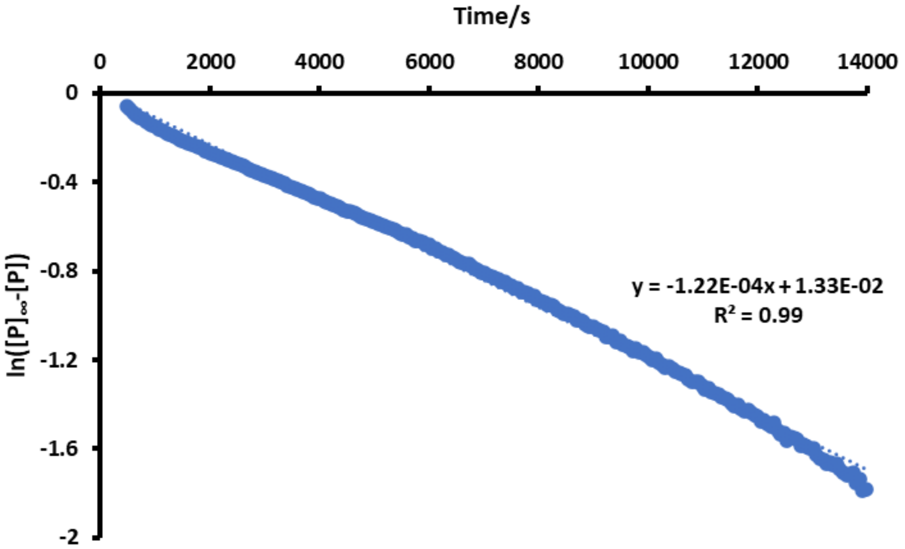

Figure S54. Straight-line plot by using the integrated first order rate law when using data from Figure S53.

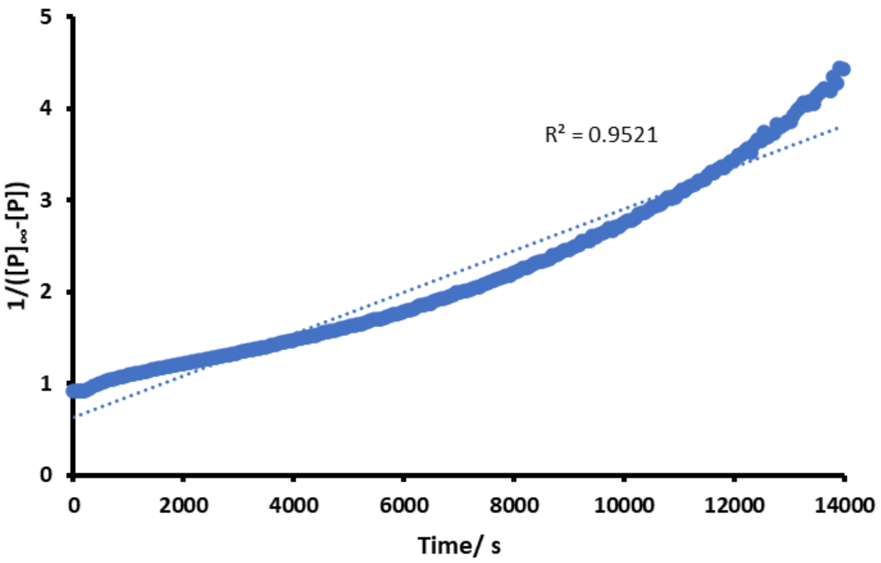

Figure S55. Attempted straight-line plot by using the integrated second order rate law when using data from Figure S53.

__Run 2__

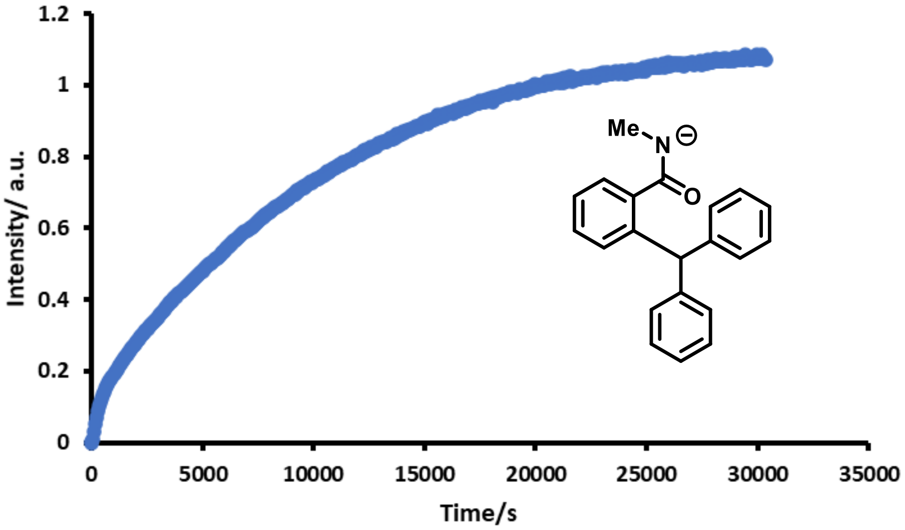

Figure S56. Reaction profile for aryl migration of 2-benzyl-*N*-methyl-*N*-phenylbenzamide (**1**) by monitoring formation of signal at 1552 cm^{-1}.

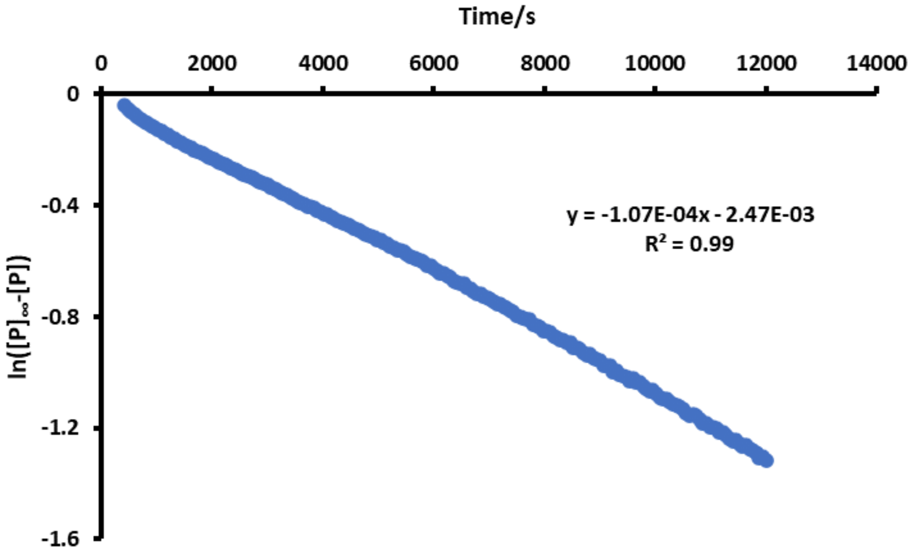

Figure S57. Straight-line plot by using the integrated first order rate law when using data from Figure S56.

__Run 3__

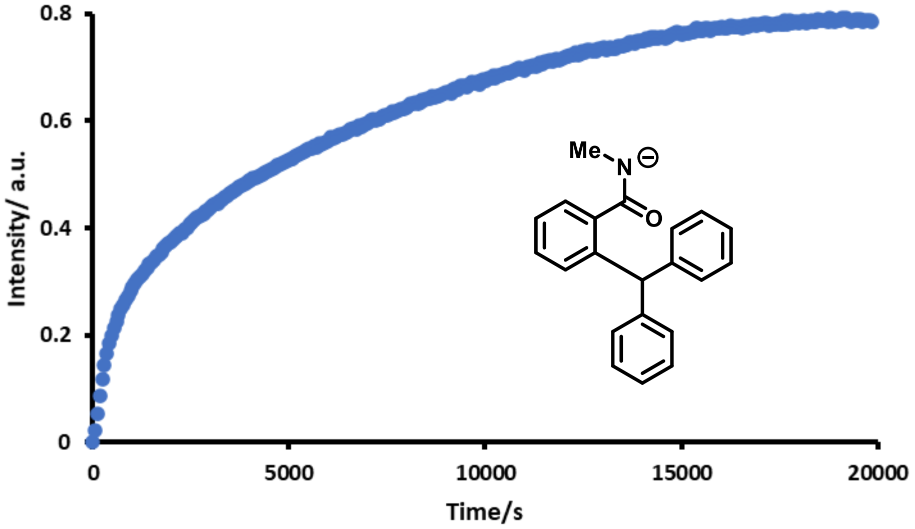

Figure S57. Reaction profile for aryl migration of 2-benzyl-*N*-methyl-*N*-phenylbenzamide (**1**) by monitoring formation of signal at 1553 cm^{-1}.

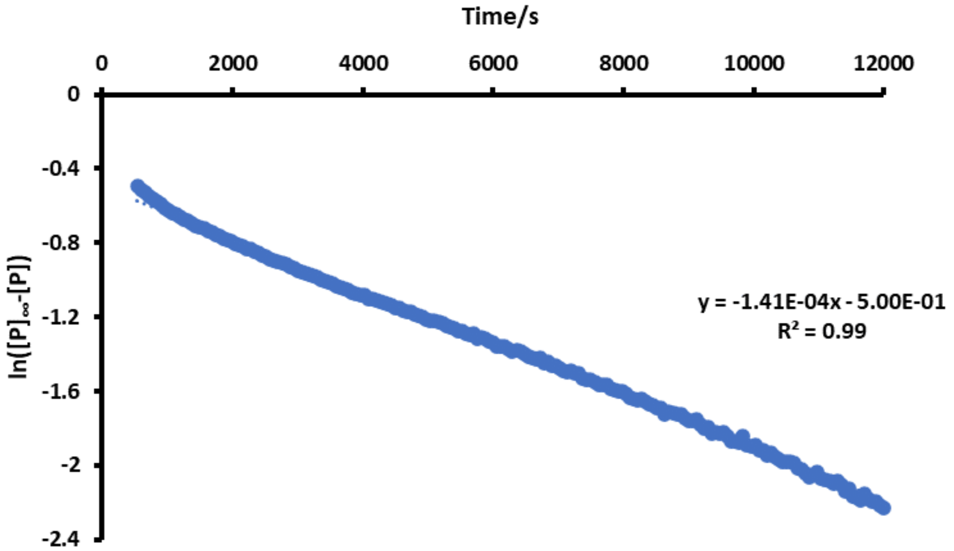

Figure S58. Straight-line plot by using the integrated first order rate law when using data from Figure S57.

Average k_{obs} over three run = 1.23 x 10^{-4} s^{-1}.

2-Benzyl-*N*-methyl-*N*-(*p*-tolyl)benzamide (3b) - Run 1

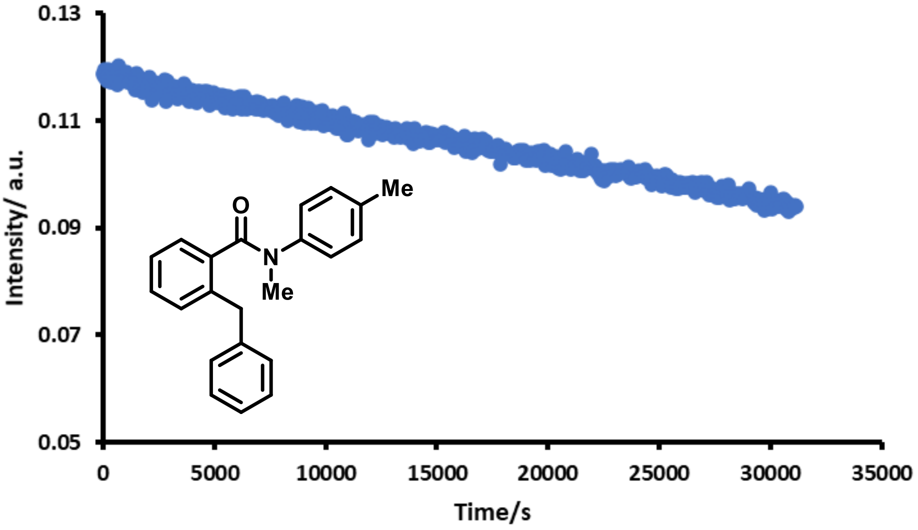

Figure S59. Reaction profile for aryl migration of 2-benzyl-*N*-methyl-*N*-(*p*-tolyl)benzamide (**3b**) by monitoring decay of signal at 1649 cm^{-1}.

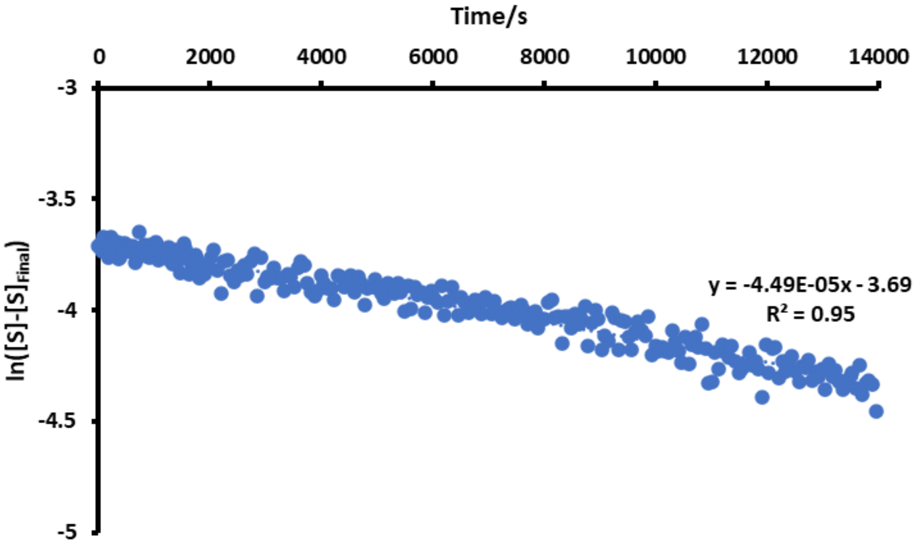

Figure S60. Straight-line plot by using the integrated first order rate law when using data from Figure S59.

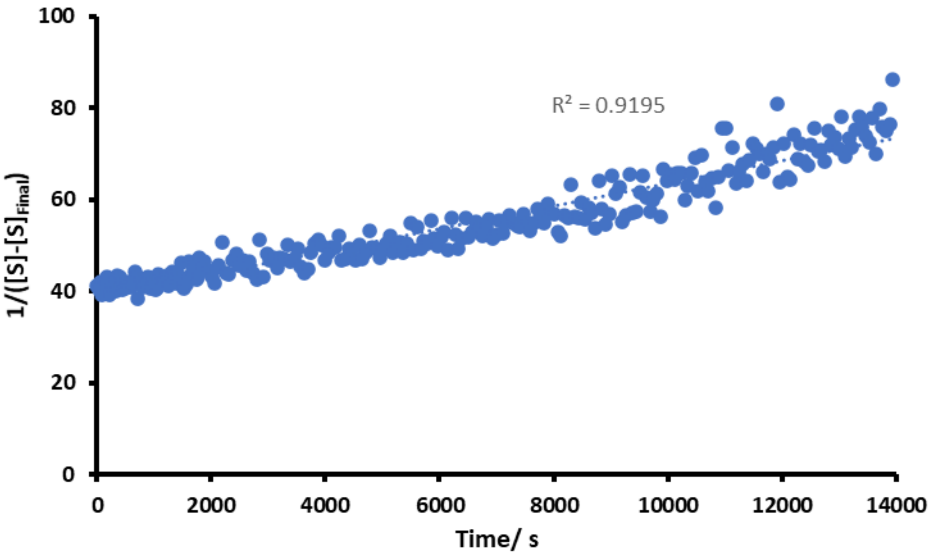

Figure S61. Attempted straight-line plot by using the integrated second order rate law when using data from Figure S59.

Run 2

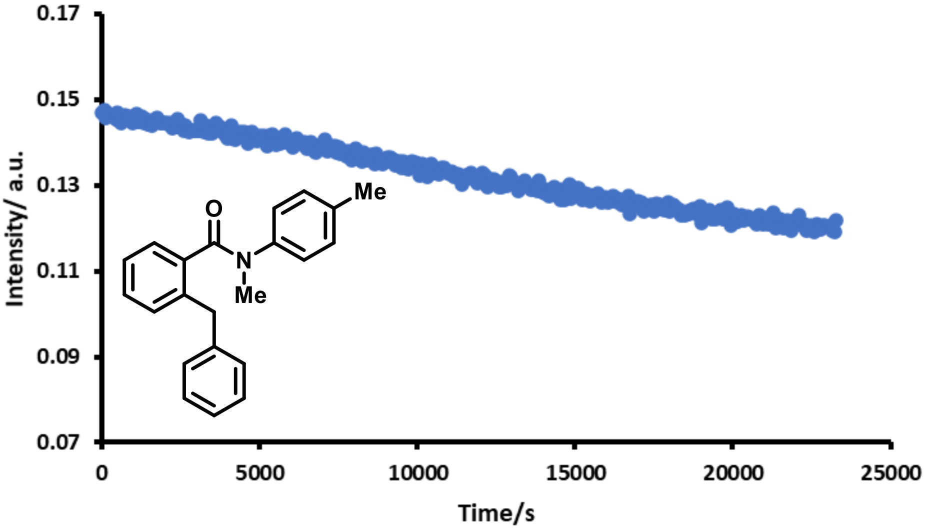

Figure S62. Reaction profile for aryl migration of 2-benzyl-*N*-methyl-*N*-(*p*-tolyl)benzamide (**3b**) by monitoring decay of signal at 1648 cm^{-1}.

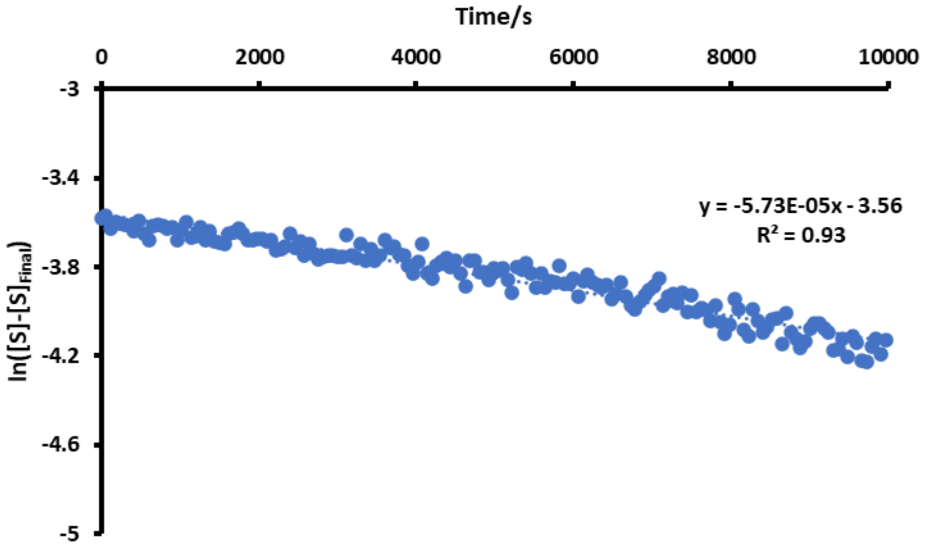

Figure S63. Straight-line plot by using the integrated first order rate law when using data from Figure S62.

<u>Run 3</u>

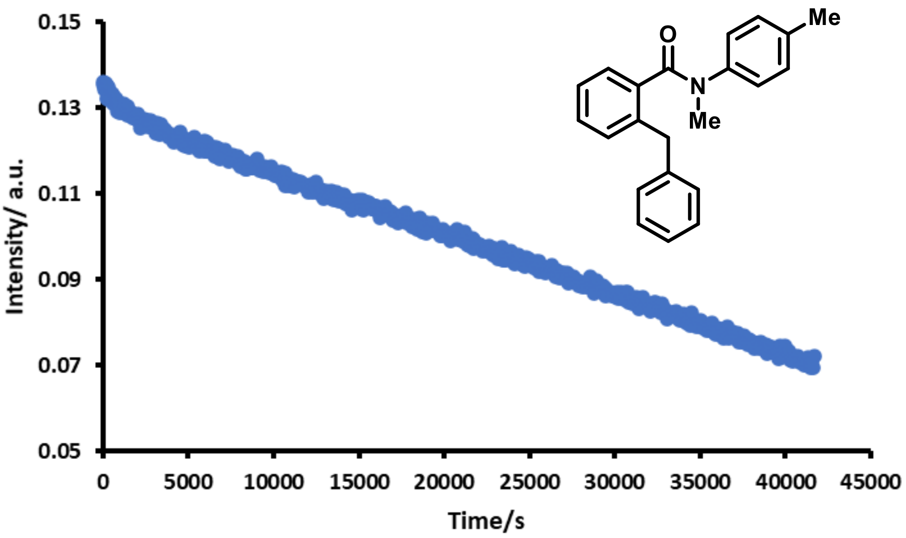

Figure S64. Reaction profile for aryl migration of 2-benzyl-*N*-methyl-*N*-(*p*-tolyl)benzamide (**3b**) by monitoring decay of signal at 1648 cm^{-1}.

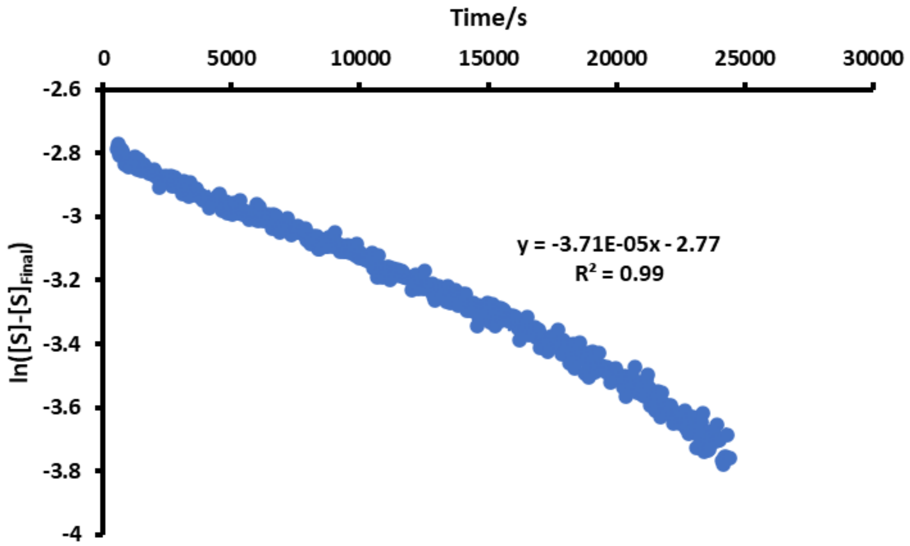

Figure S65. Straight-line plot by using the integrated first order rate law when using data from Figure S64.

Average k_{obs} over three run = 4.64 x 10^{-5} s^{-1}.

Complete data for Hammett Plot:

R	σ^{-} [13]	Avg. k_{obs}/ s^{-1}	k_R/k_H	$\log(k_R/k_H)$	1 SD from $\log(k_R/k_H)$
p-Me	-0.17	4.64×10^{-5}	3.76×10^{-1}	-0.42	0.10
H	0	1.23×10^{-4}	1.00×10^{0}	0	0.06
p-Cl	0.19	1.55×10^{-3}	1.25×10^{1}	1.10	0.07
p-Br	0.25	4.71×10^{-3}	3.82×10^{1}	1.58	0.08
m-Cl	0.37	1.31×10^{-2}	1.07×10^{2}	2.03	0.10
2-Py	0.55	2.41×10^{-2}	1.95×10^{2}	2.29	0.02
3-Py	0.58	3.54×10^{-2}	2.87×10^{2}	2.46	0.03

Table S2. Collated data for Hammett plot. SD – Standard Deviation.

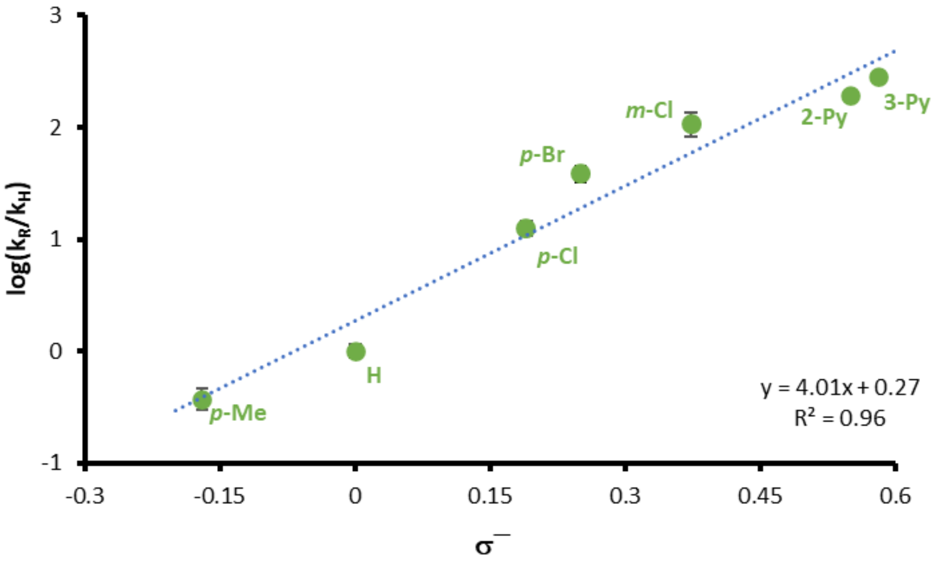

Figure S66. Hammett plot constructed from data in Table S2. Error bar in a direction away from data point represents one standard deviation, in most cases data point is larger than error bars.

7. References

[1] D. F. Wyss, A. Arasappan, M. M. Senior, Y.-S. Wang, B. M. Beyer, F. G. Njoroge, M. A. McCoy, *J. Med. Chem*., 2004, **47**, 2486-2498.

[2] A. K. Turek, D. J. Hardee, A. M. Ullman, D. G. Nocera, E. N. Jacobsen, *Angew. Chem. Int. Ed.*, 2016, **55**, 539-544.

[3] Y. Wang, S. Yang, G. Bian, L. Song, *Synthesis*, 2017, **49**, 1884-1888.

[4] T. Serevičius, R. Komskis, P. Adomėnas, O. Adomėnienė, V. Jankauskas, A. Gruodis, K. Kazlauskasa, S. Juršėnasa, *Phys. Chem. Chem. Phys.*, 2014, **16**, 7089-7101.

[5] S. Alazet, F. Le Vaillant, S. Nicolai, T. Courant, J. Waser, *Chem. Eur. J.*, 2017, **23**, 9501-9504.

[6] A. K. Bhattacharya, B. Miller, *J. Am. Chem. Soc.*, 1983, **105**, 3242-3247.

[7] L. Zhang, G. Y. Ang, S. Chiba, *Org. Lett.*, 2011, **13**, 1622-1625.

[8] S. P. Runyon, P. D. Mosier, B. L. Roth, R. A. Glennon, R. B. Westkaemper, *J. Med. Chem.*, 2008, **51**, 6808-6828.

[9] W. Zhang, J. Zhang, X. Chen, Z. Mao, X. Xie, L. Wang, Y. Liao, G. Yu, Y. Liua, D. Zhu, *J. Mater. Chem. C*, 2013, **1**, 6403-6410.

[10] M. L. Tedjamulia, Y. Tominaga, R. N. Castle, M. L. Lee, *J. Heterocycl. Chem.*, 1983, **20**, 861-866.

[11] E. T. Nadres, G. I. F. Santos, D. Shabashov, O. Daugulis, *J. Org. Chem.*, 2013, **78**, 9689-9714.

[12] Q. Xuan, Q. Song, *Org. Lett.*, 2016, **18**, 4250-4253.

[13] C. Hansch, A. Leo, R. W. Taft, *Chem. Rev.*, 1991, **91**, 165-195.

8. NMR Spectra

^{1}H NMR of **1**

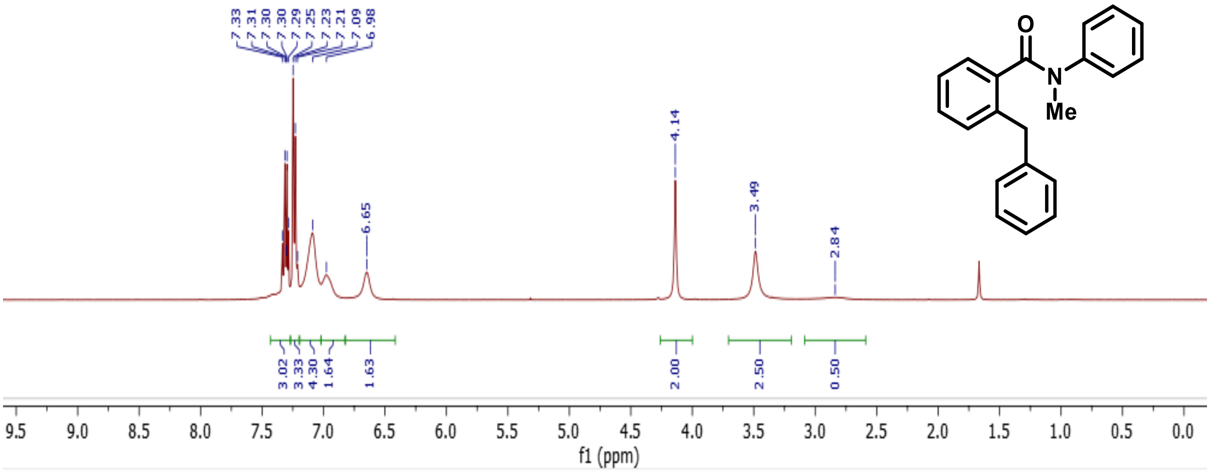

^{13}C NMR of **1**

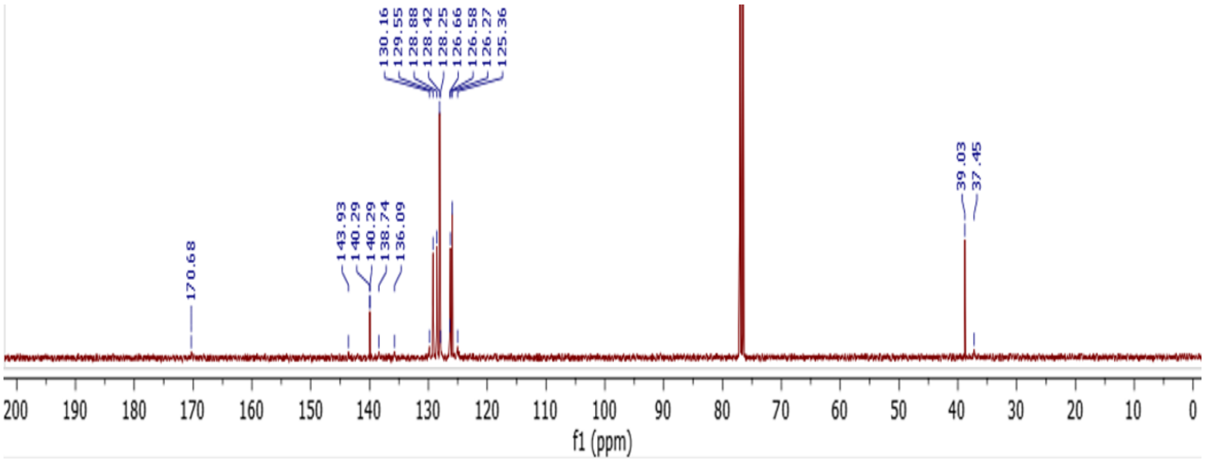

HSQC of **1**

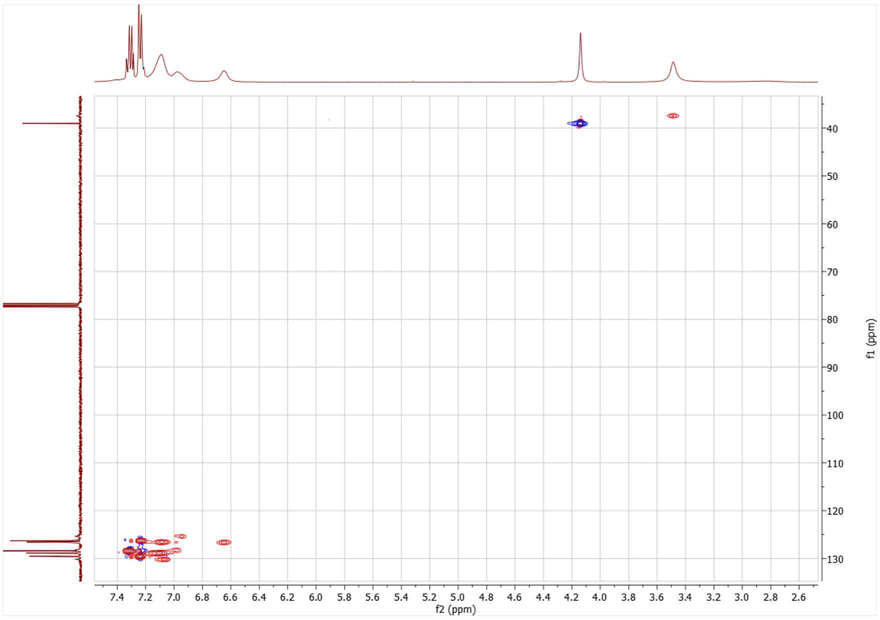

^{1}H NMR of **2**

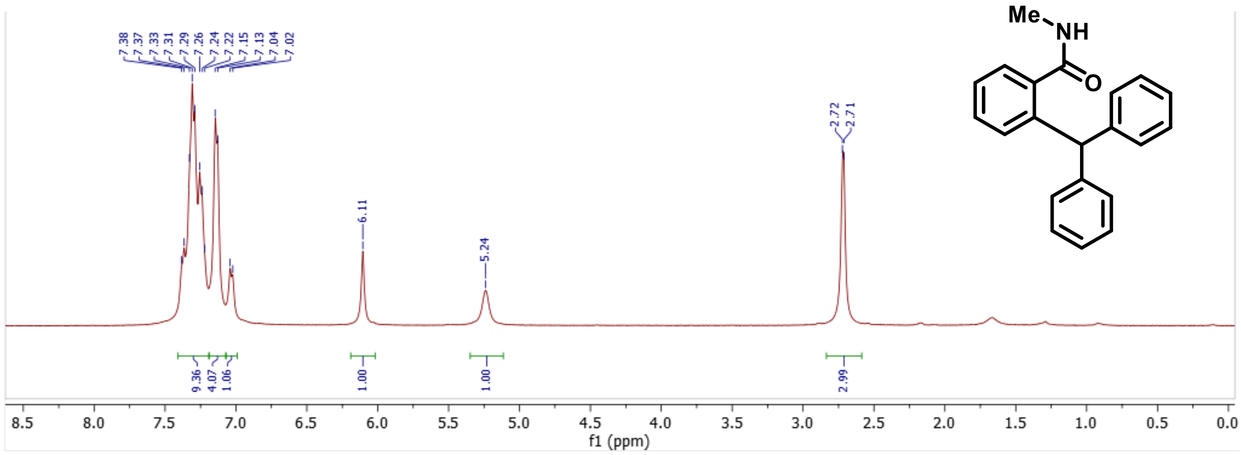

^{13}C NMR of **2**

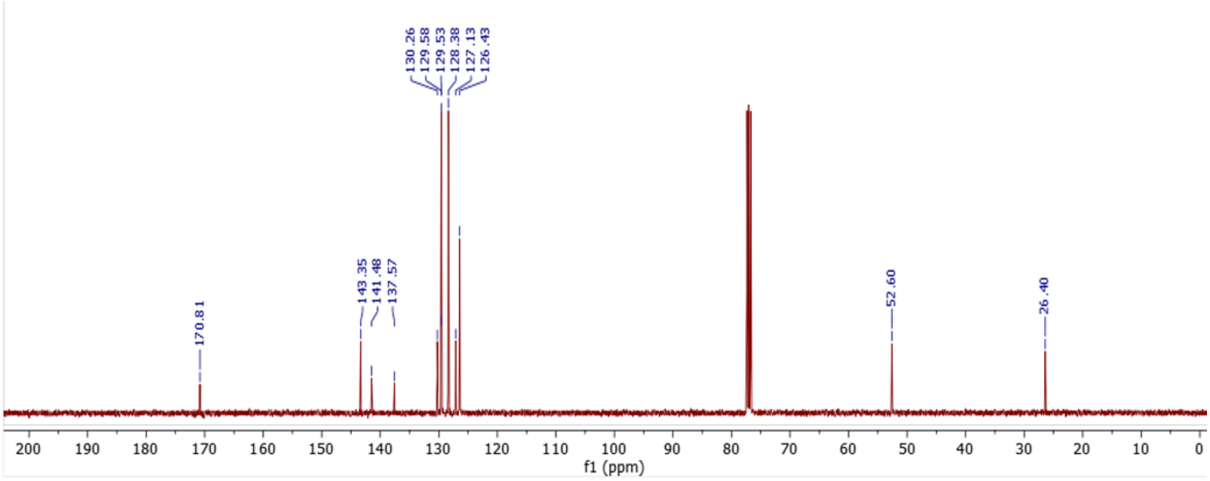

HSQC of **2**

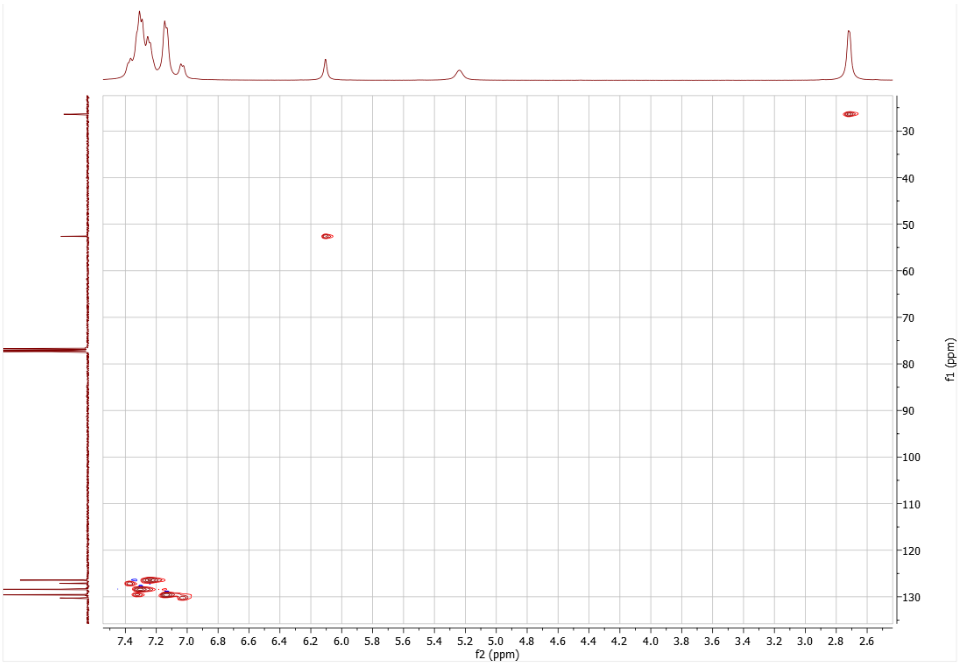

^{1}H NMR of **3a**

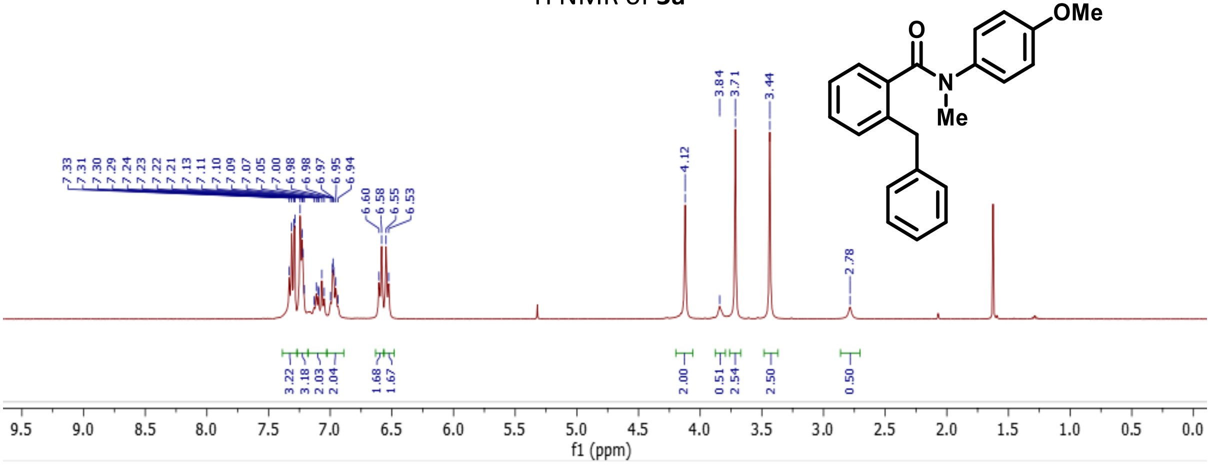

^{13}C NMR of **3a**

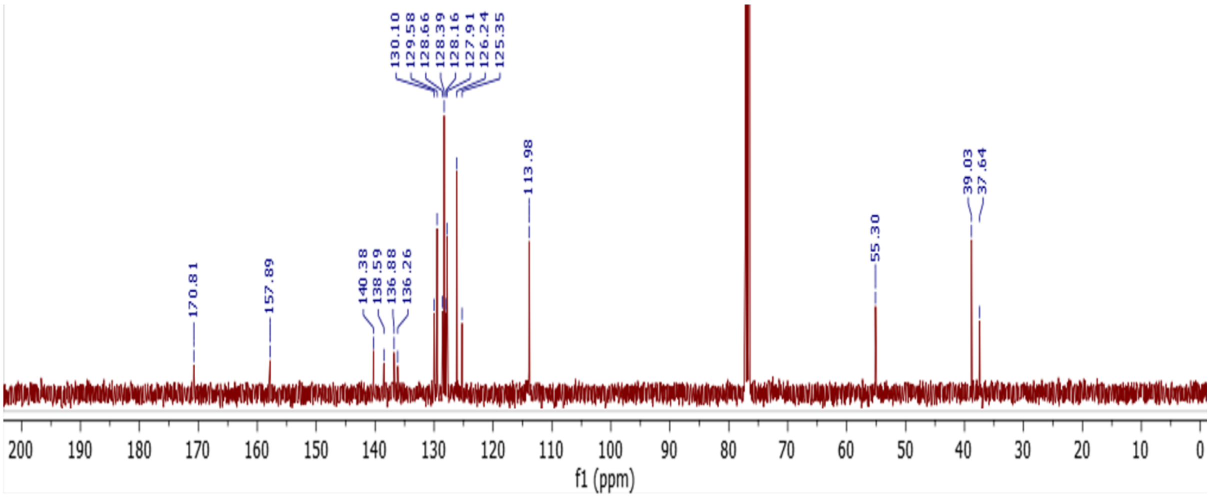

HSQC of **3a**

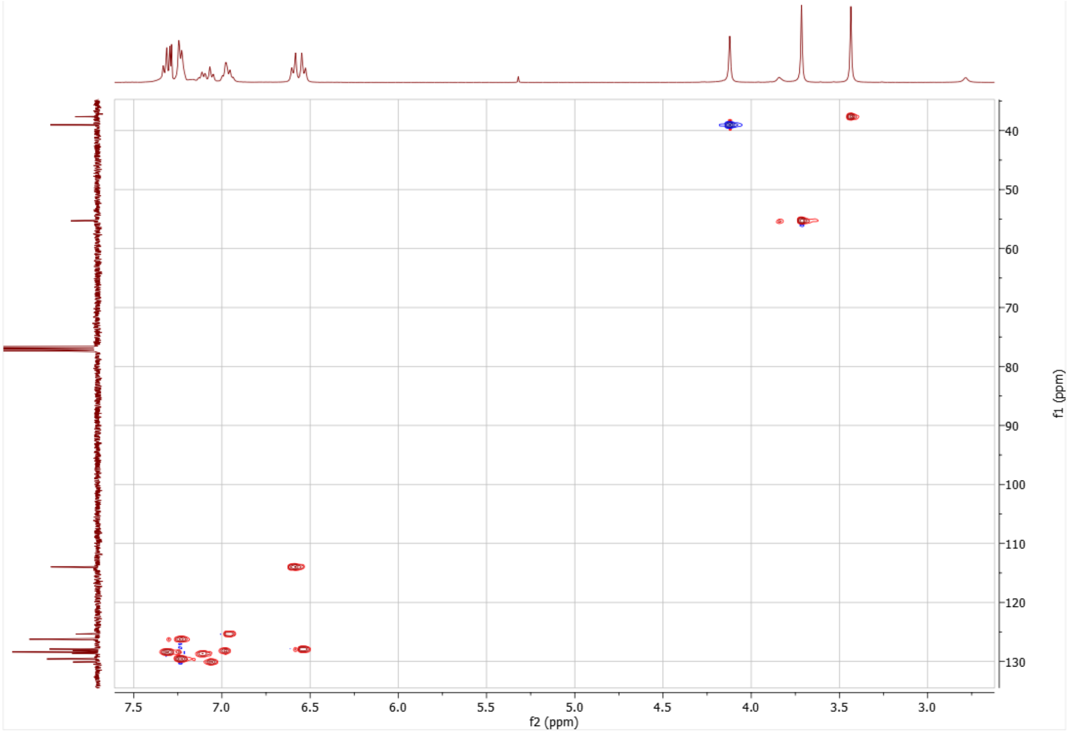

^{1}H NMR of **3b**

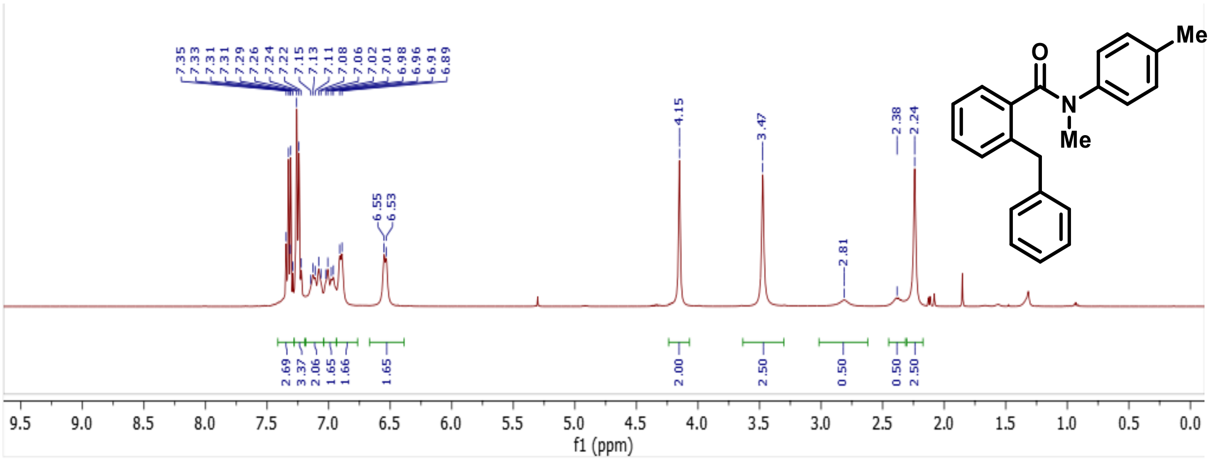

^{13}C NMR of **3b**

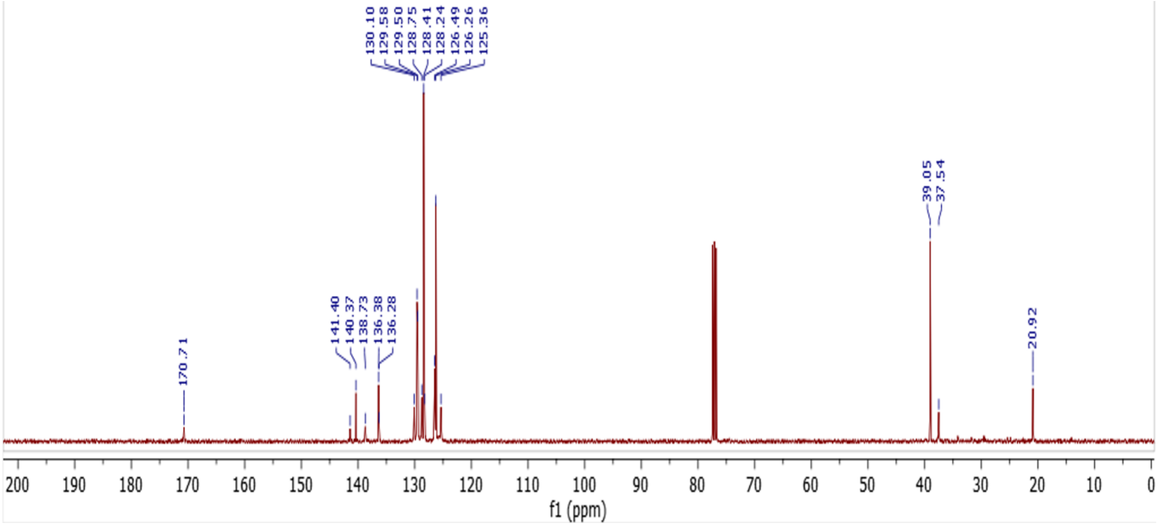

HSQC of **3b**

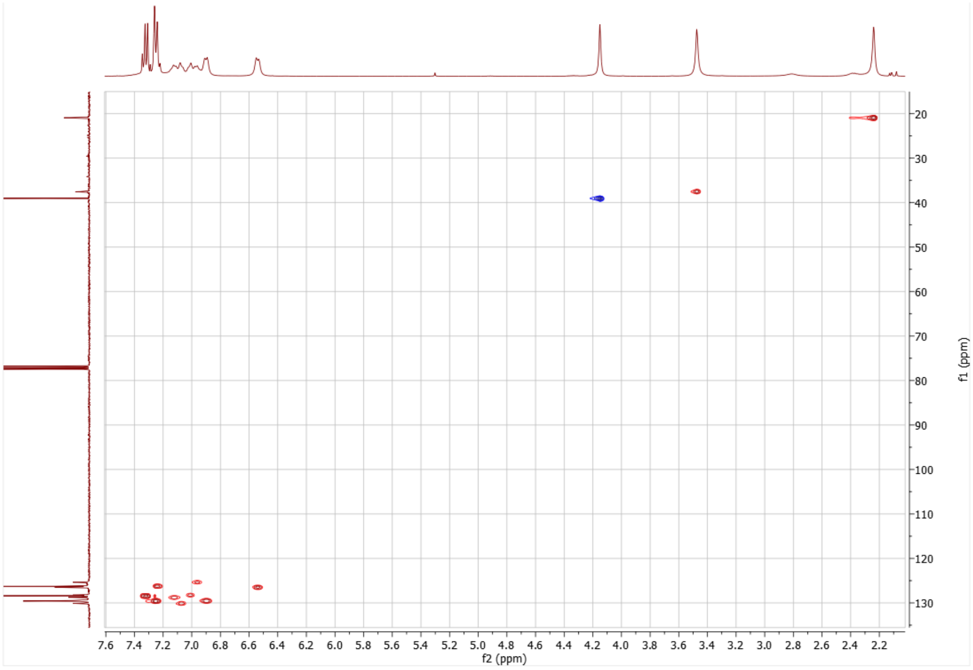

^{1}H NMR of **3c**

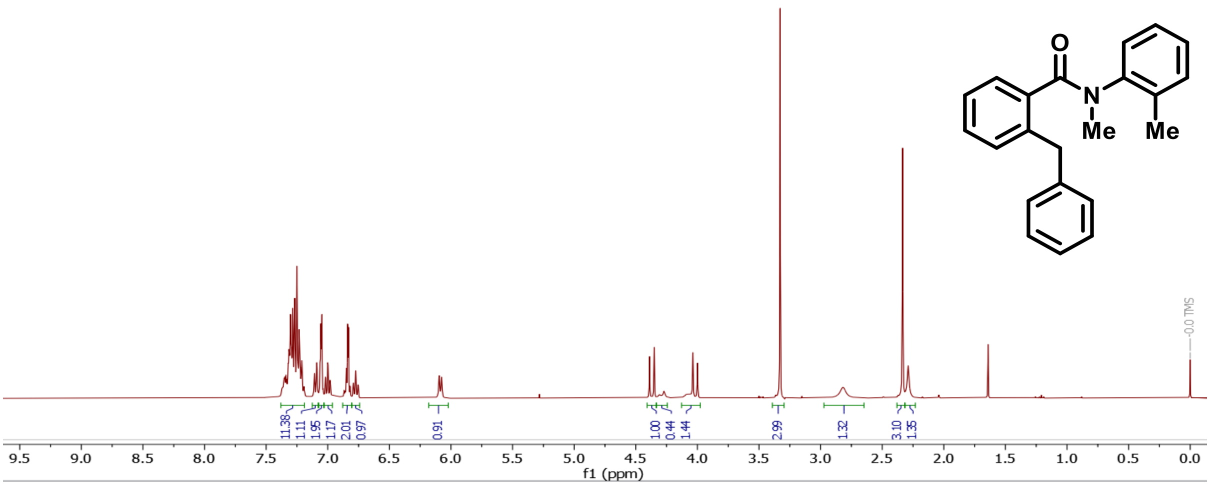

^{13}C NMR of **3c**

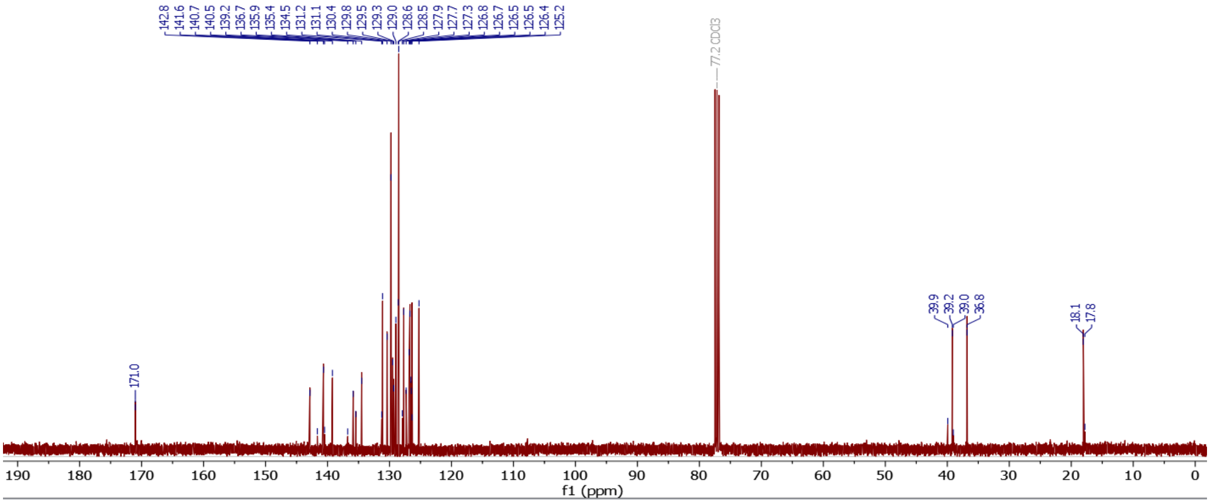

HSQC of **3c**

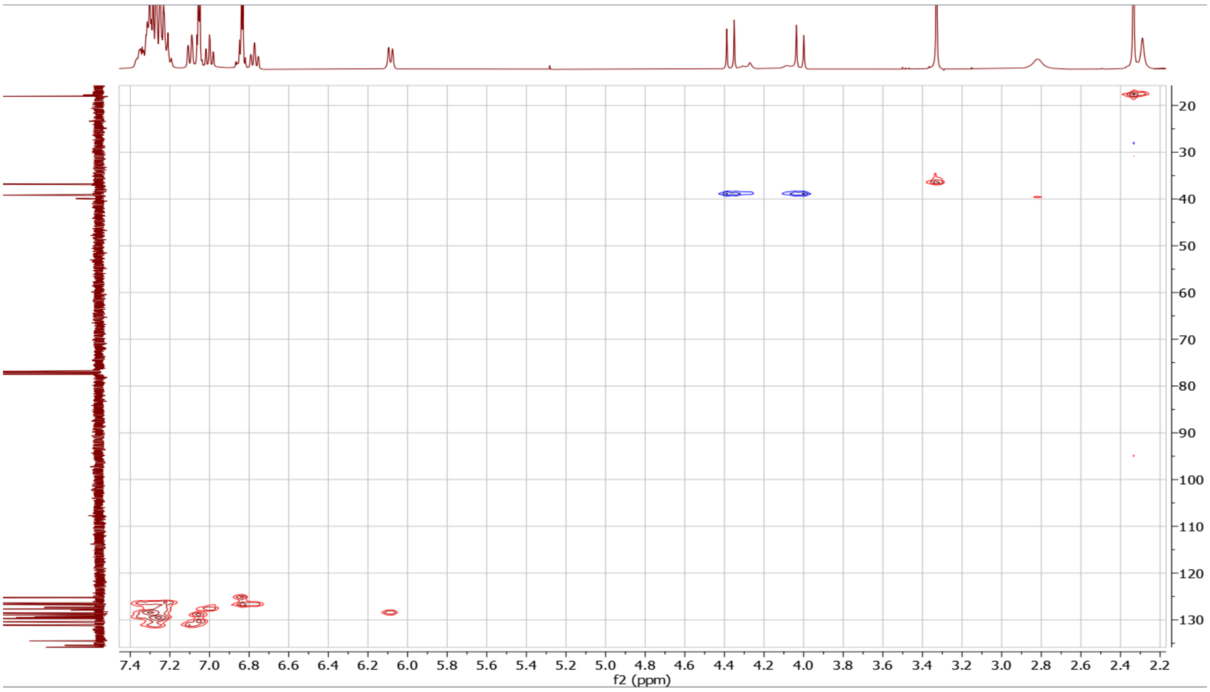

^{1}H NMR of **3d**

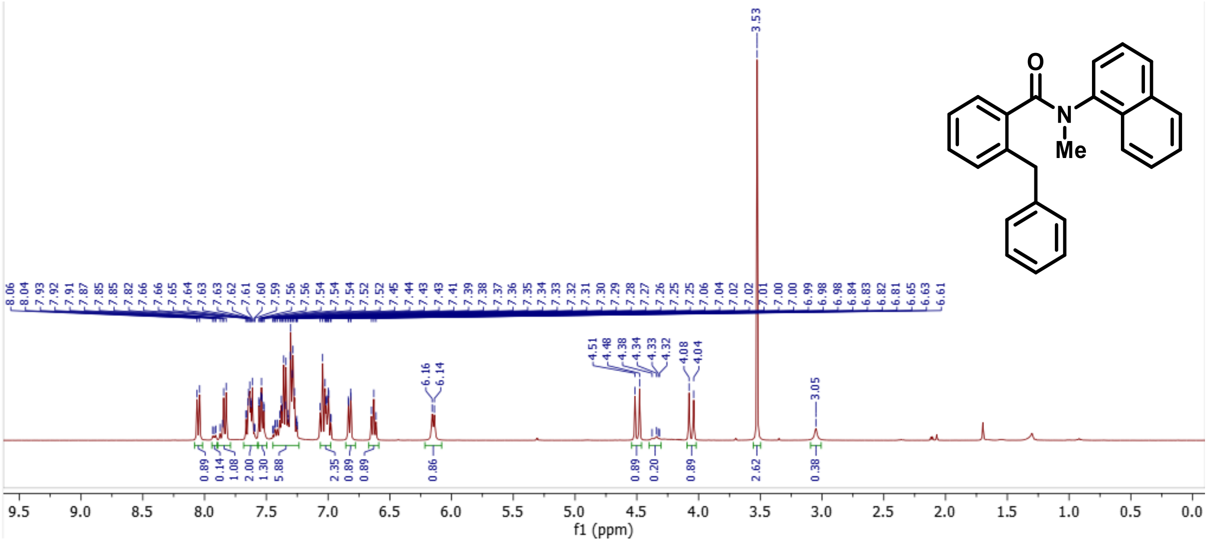

^{13}C NMR of **3d**

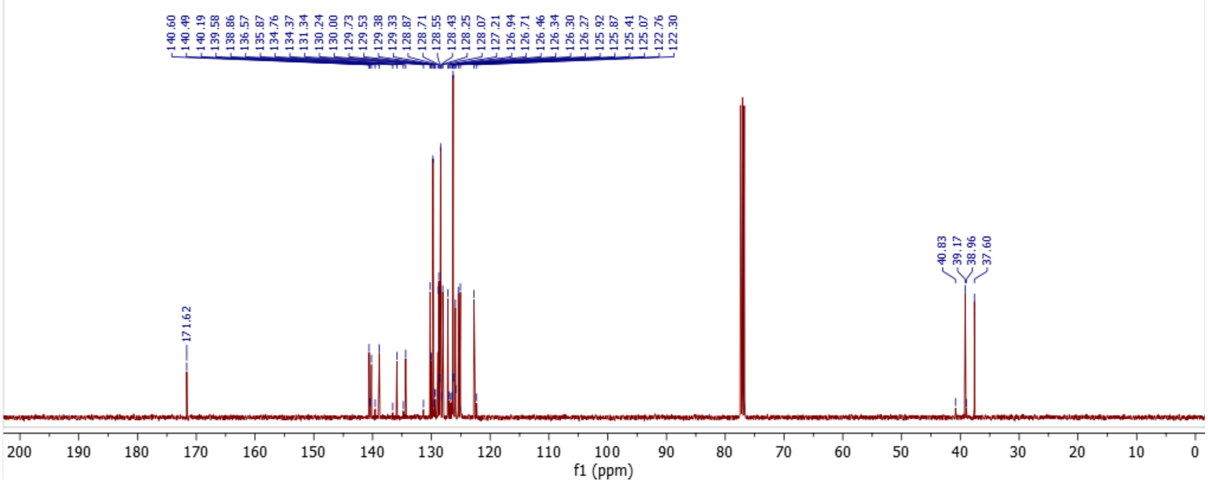

HSQC of **3d**

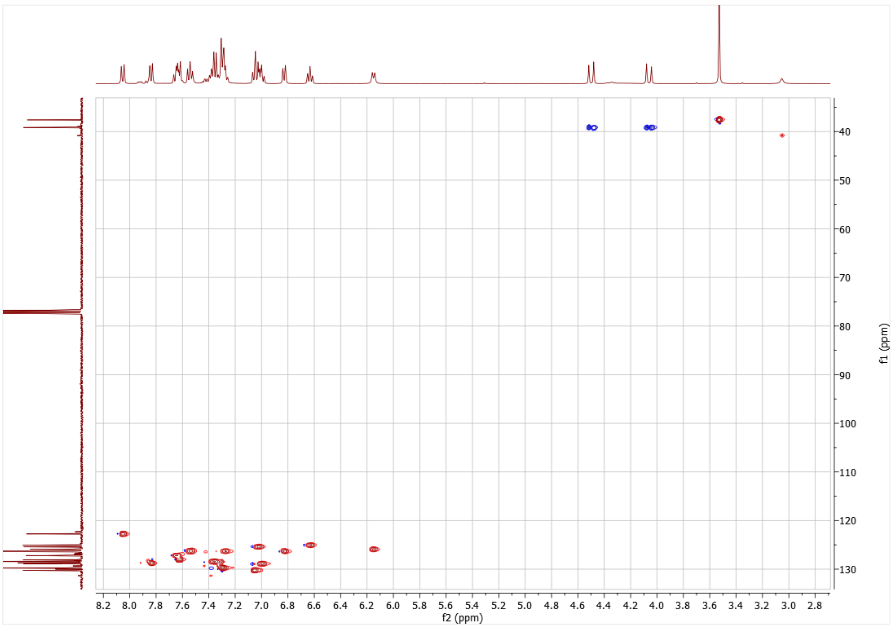

^{1}H NMR of **3e**

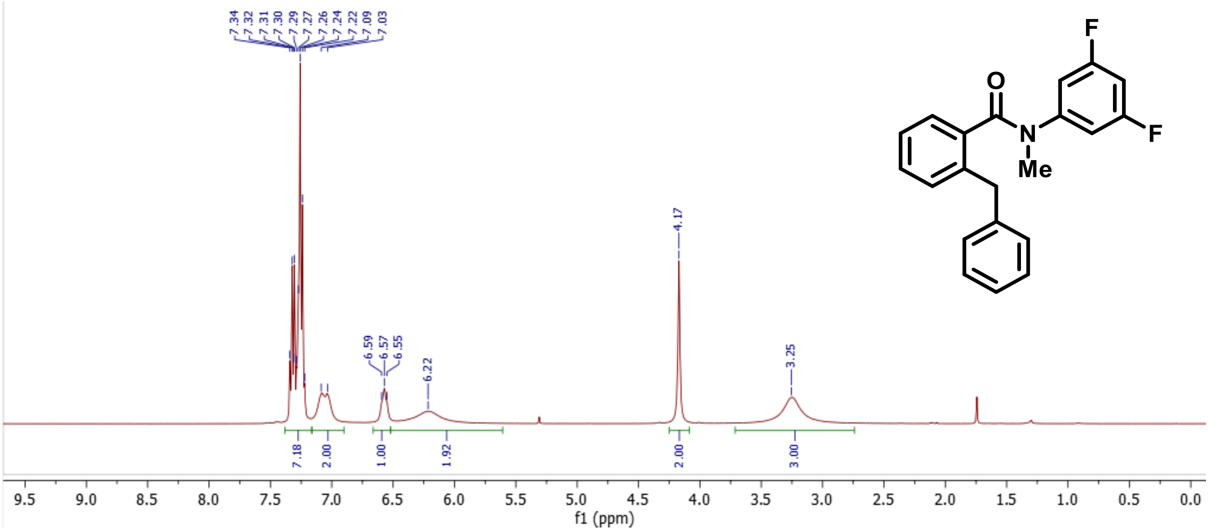

^{13}C NMR of **3e**

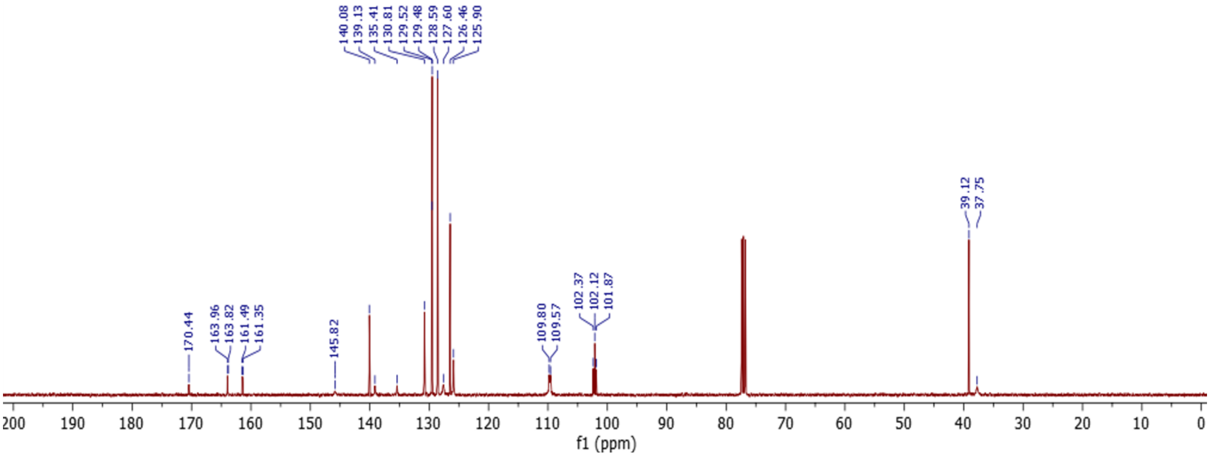

HSQC of **3e**

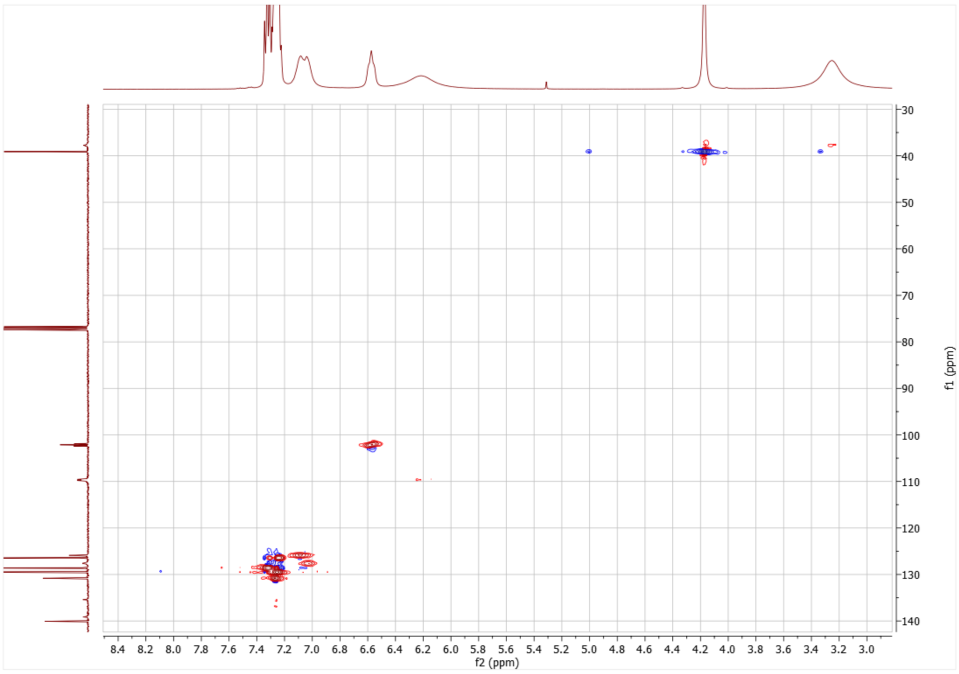

^{1}H NMR of **3f**

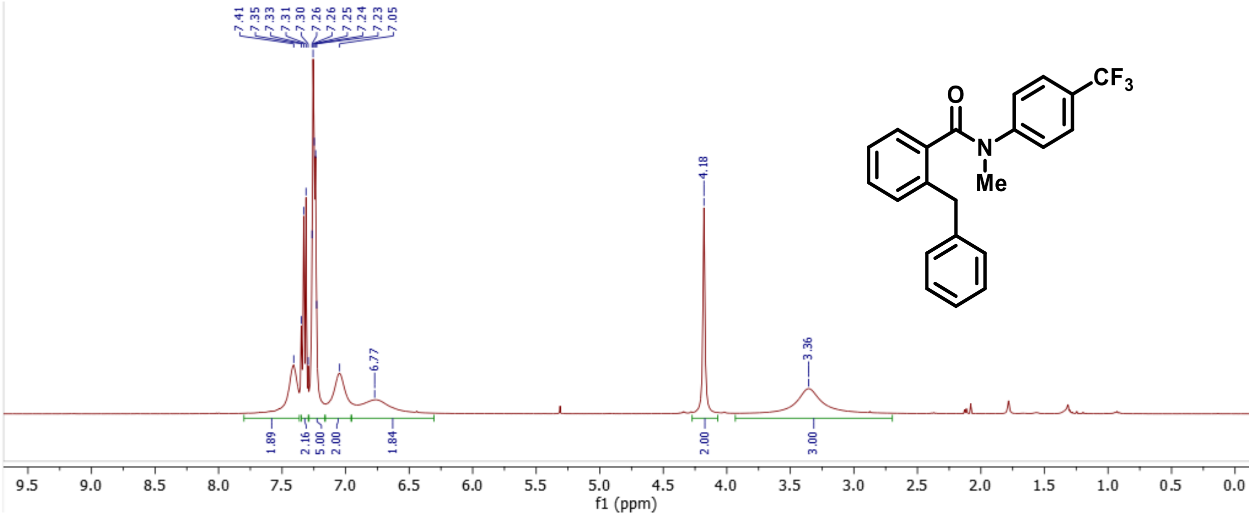

^{13}C NMR of **3f**

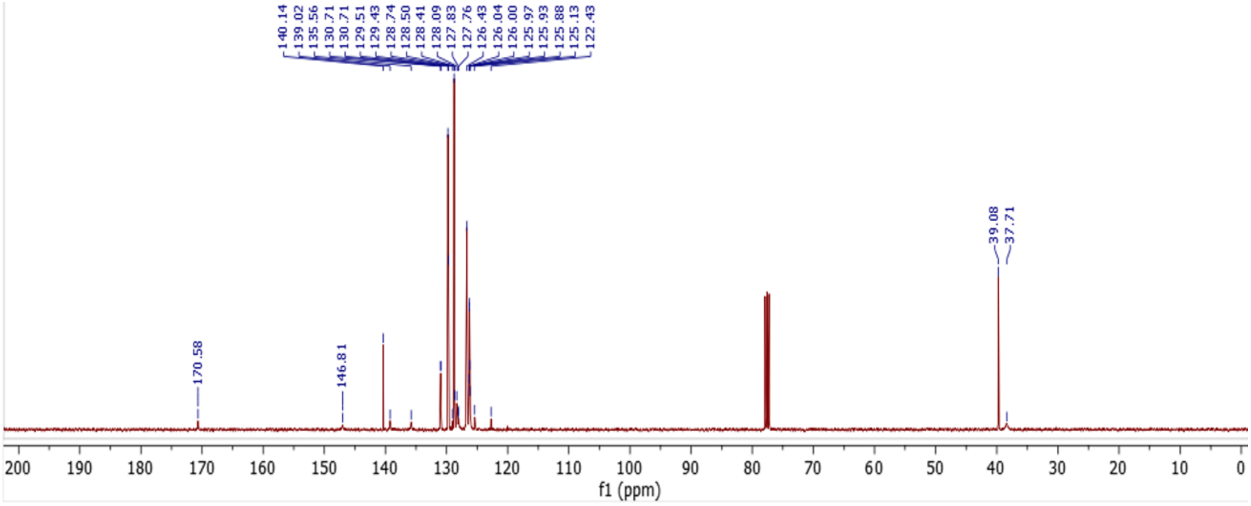

HSQC of **3f**

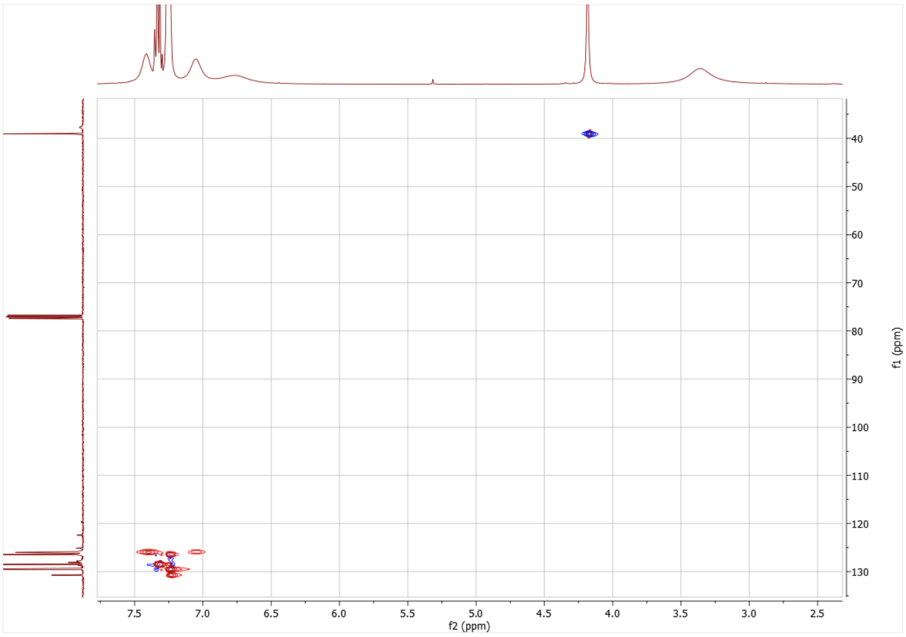

^{1}H NMR of **3g**

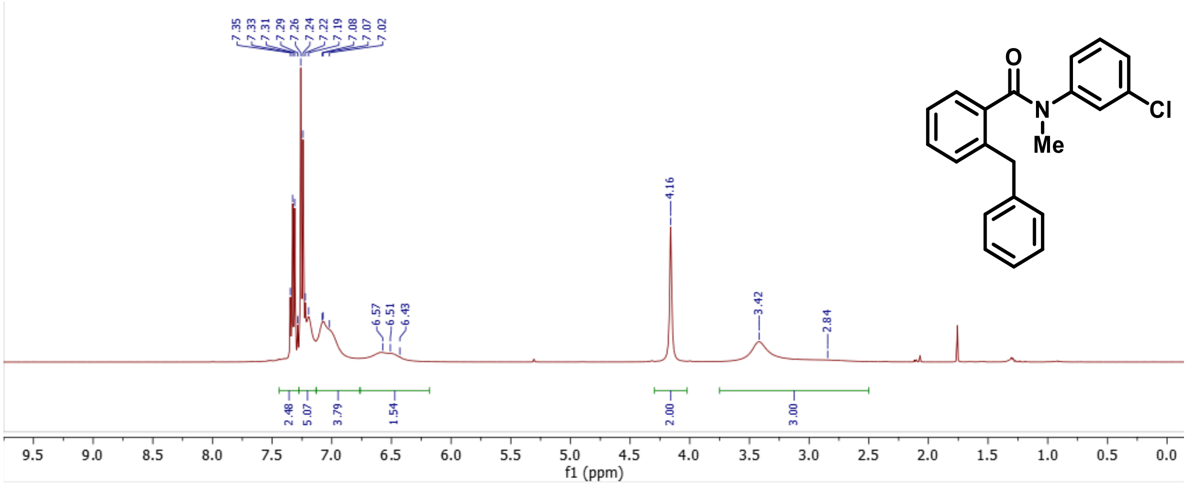

^{13}C NMR of **3g**

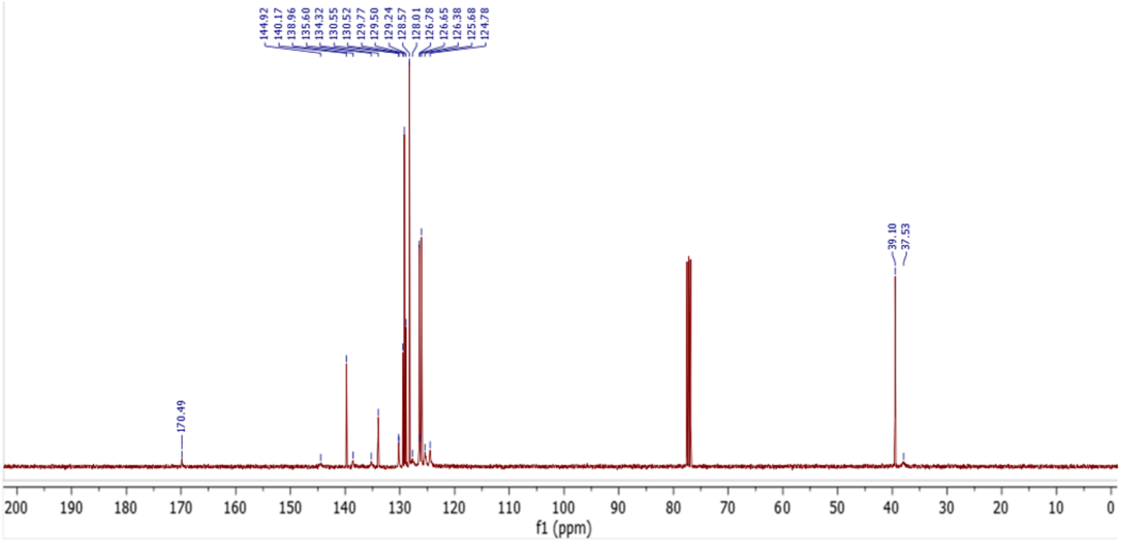

HSQC of **3g**

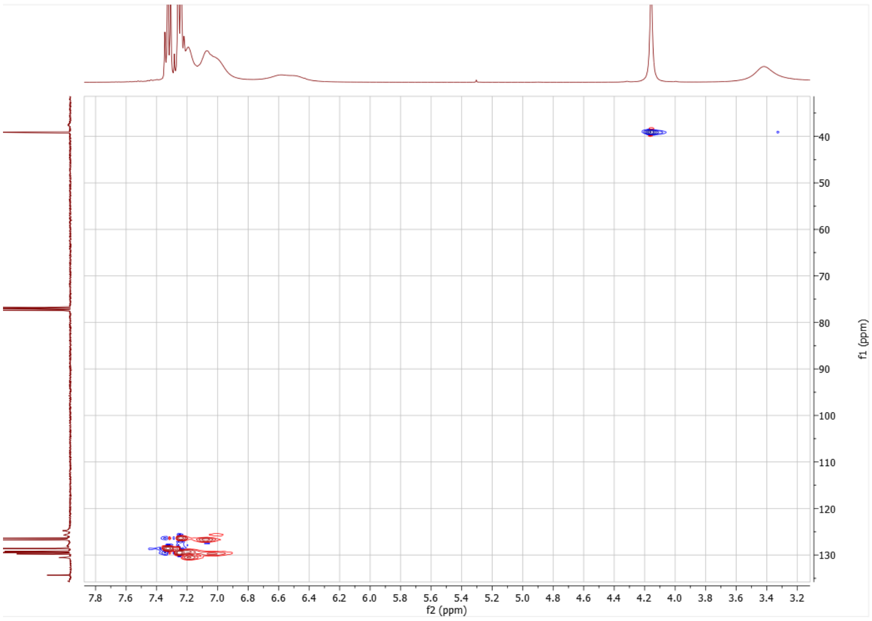

^{1}H NMR of **3h**

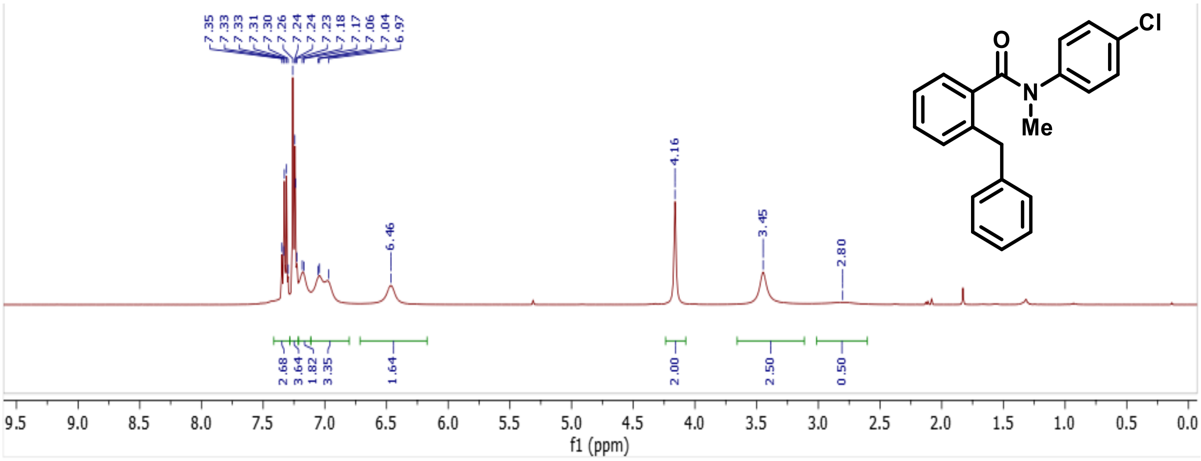

^{13}C NMR of **3h**

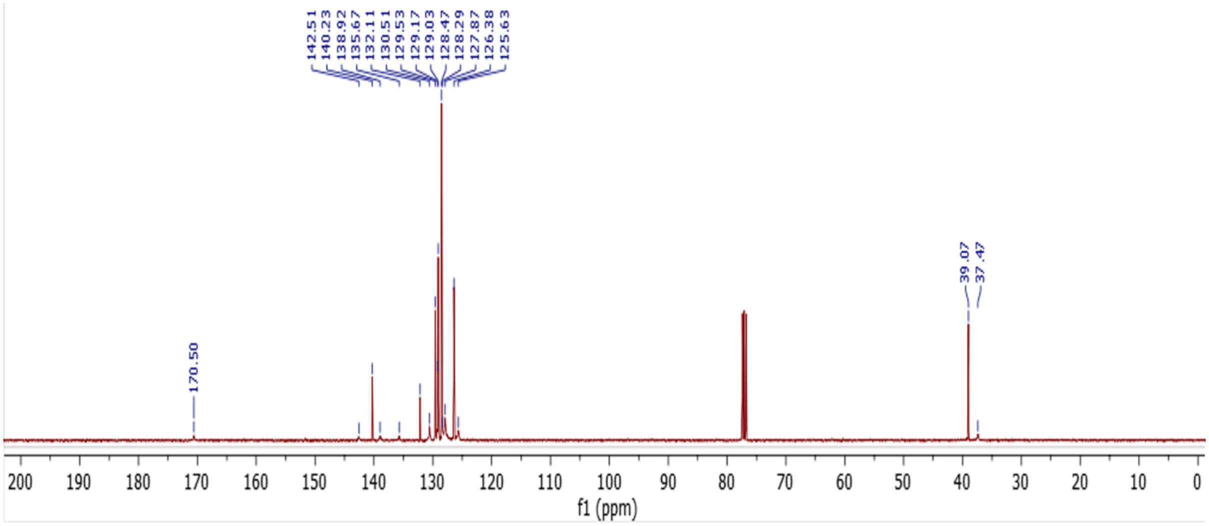

HSQC of **3h**

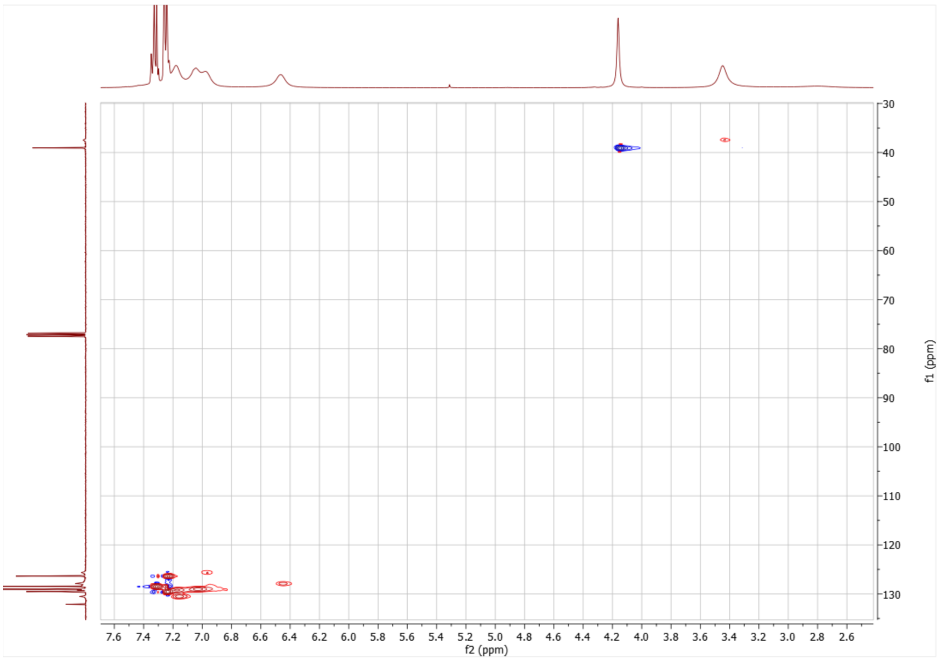

^{1}H NMR of **3i**

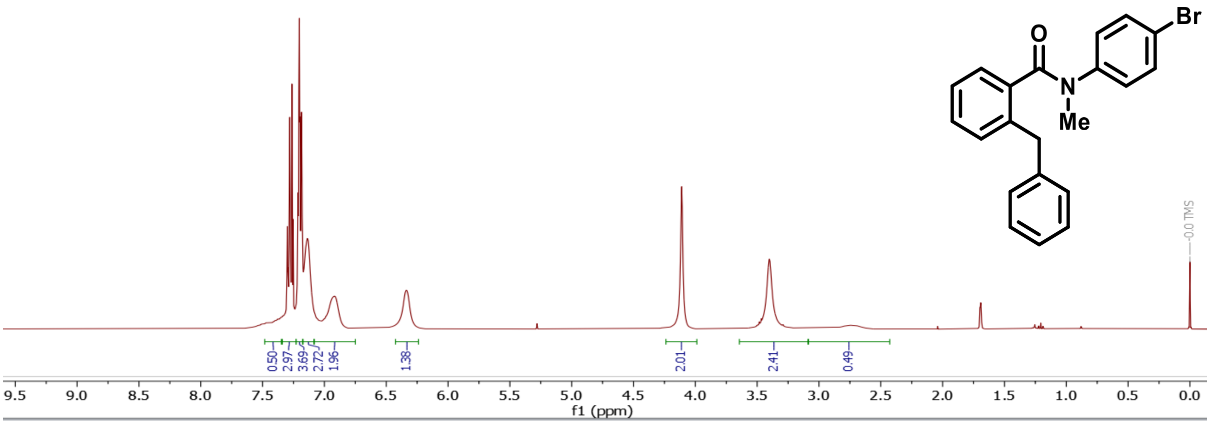

^{13}C NMR of **3i**

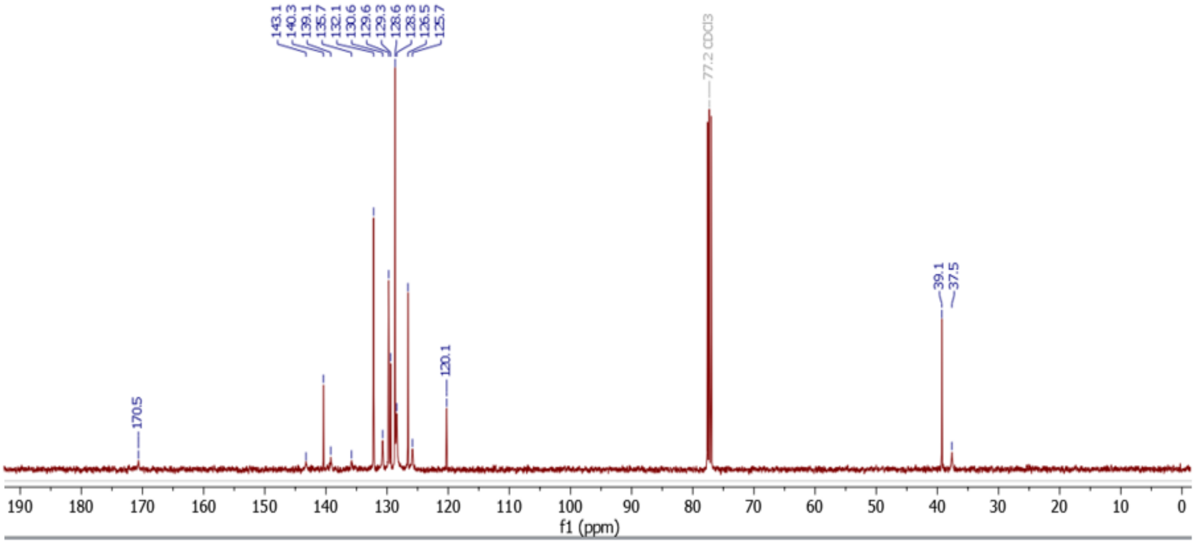

HSQC of **3i**

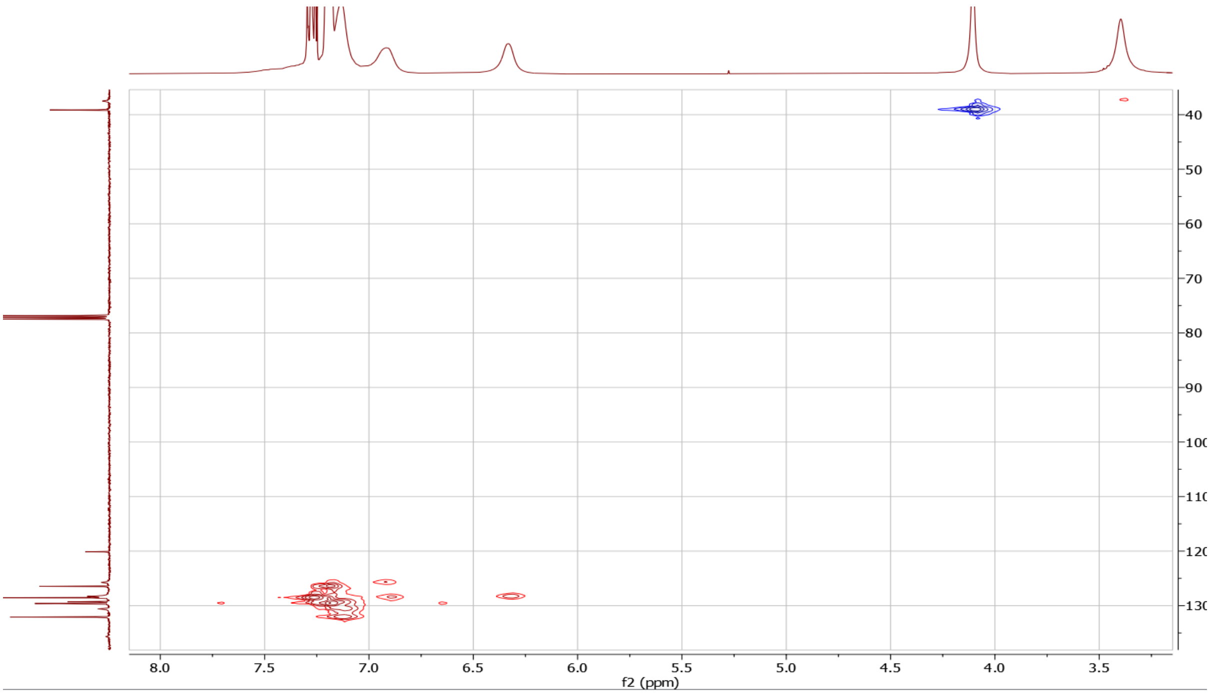

^{1}H NMR of **3j**

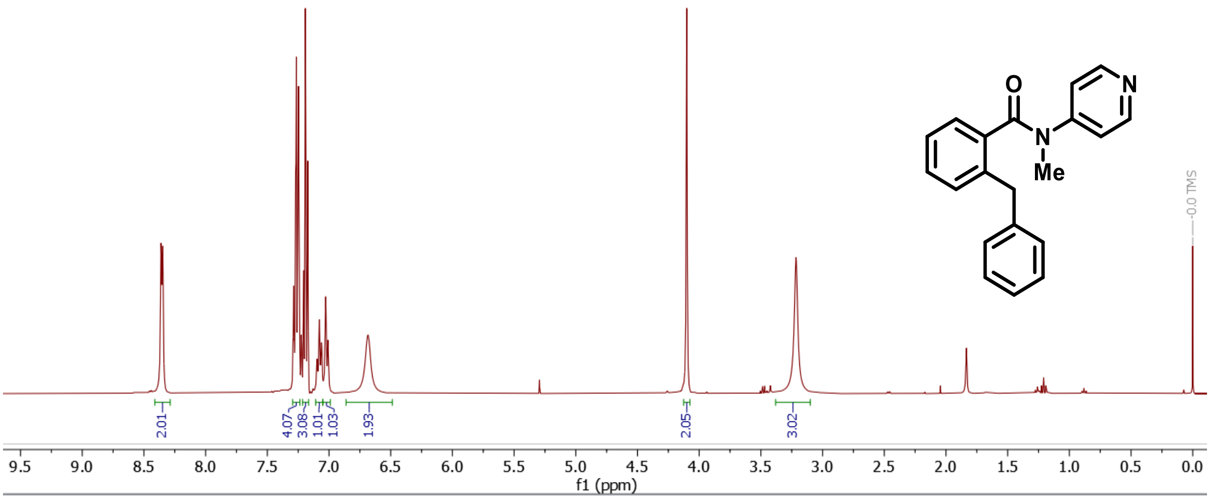

^{13}C NMR of **3j**

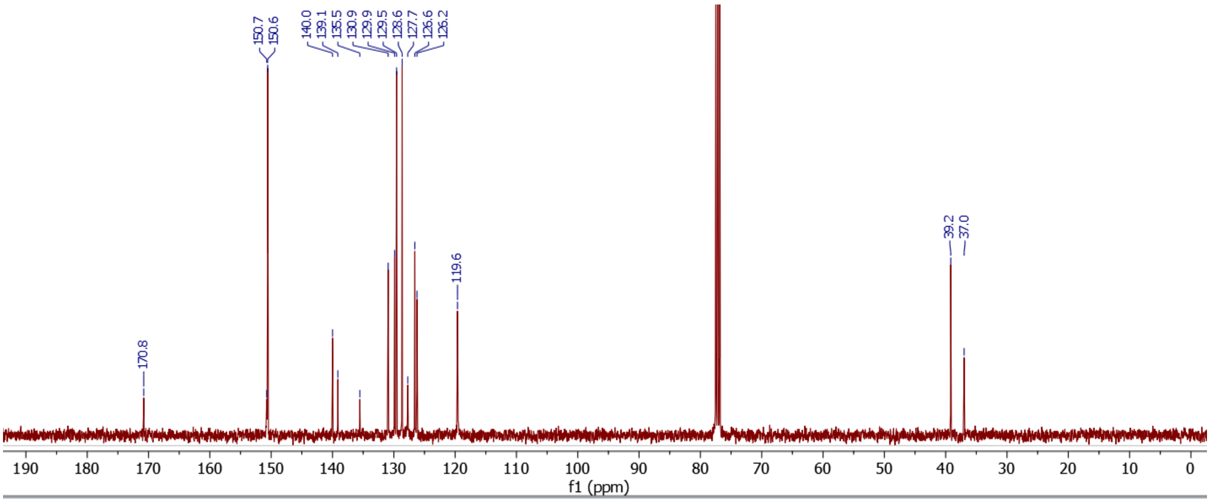

HSQC of **3j**

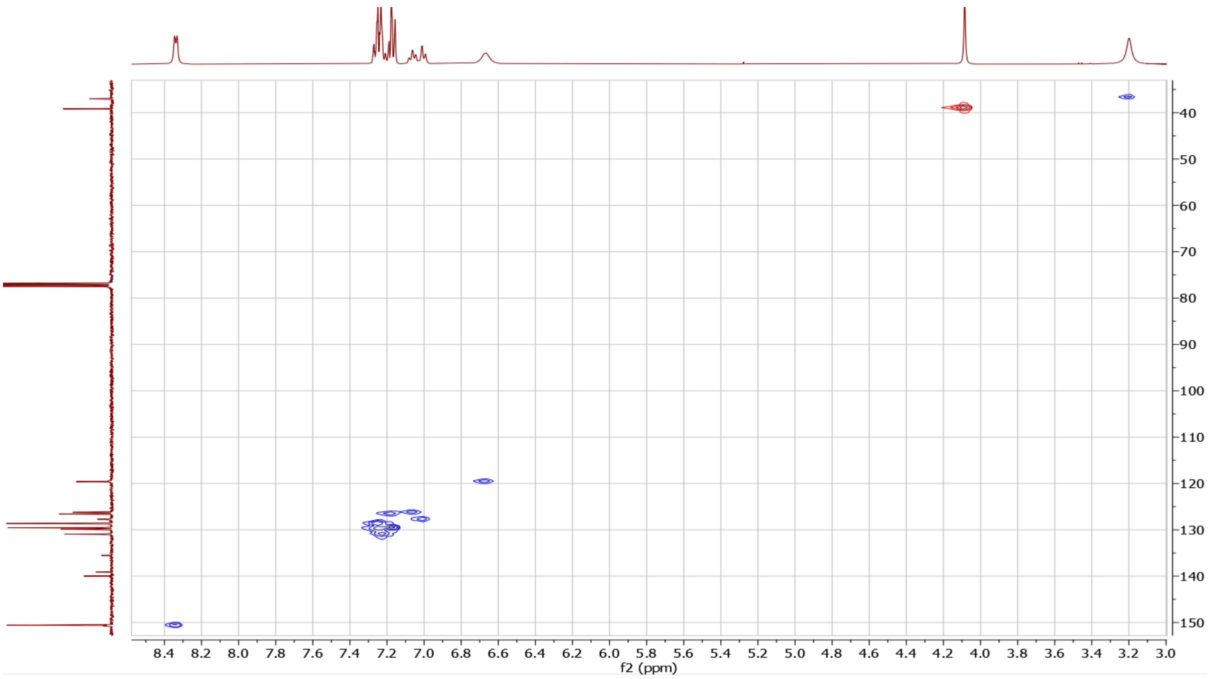

^{1}H NMR of **3k**

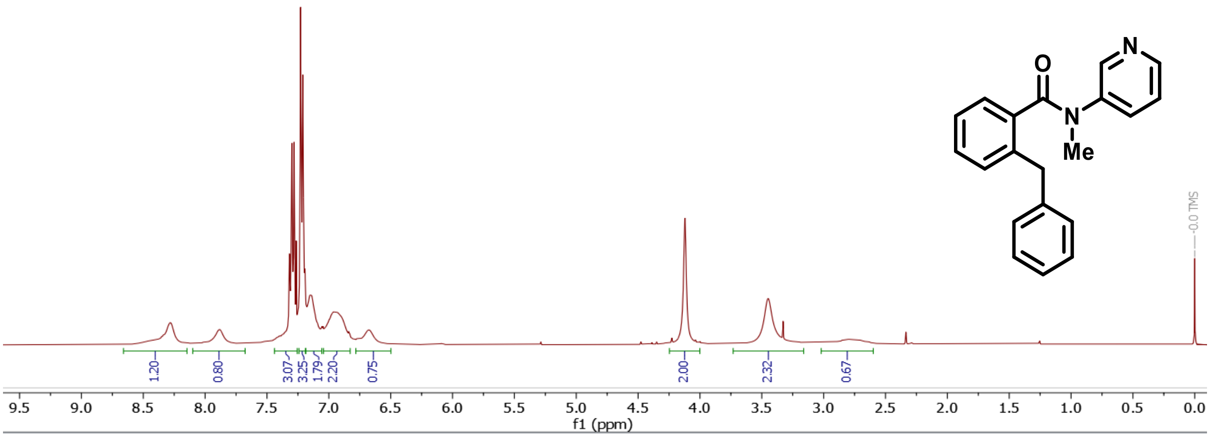

^{13}C NMR of **3k**

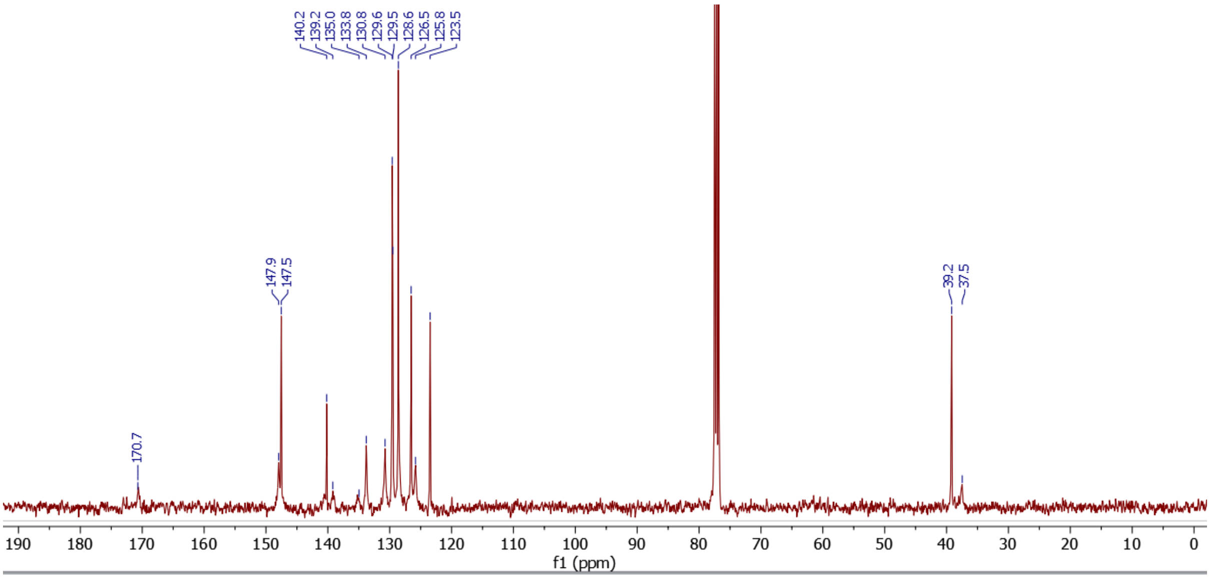

HSQC of **3k**

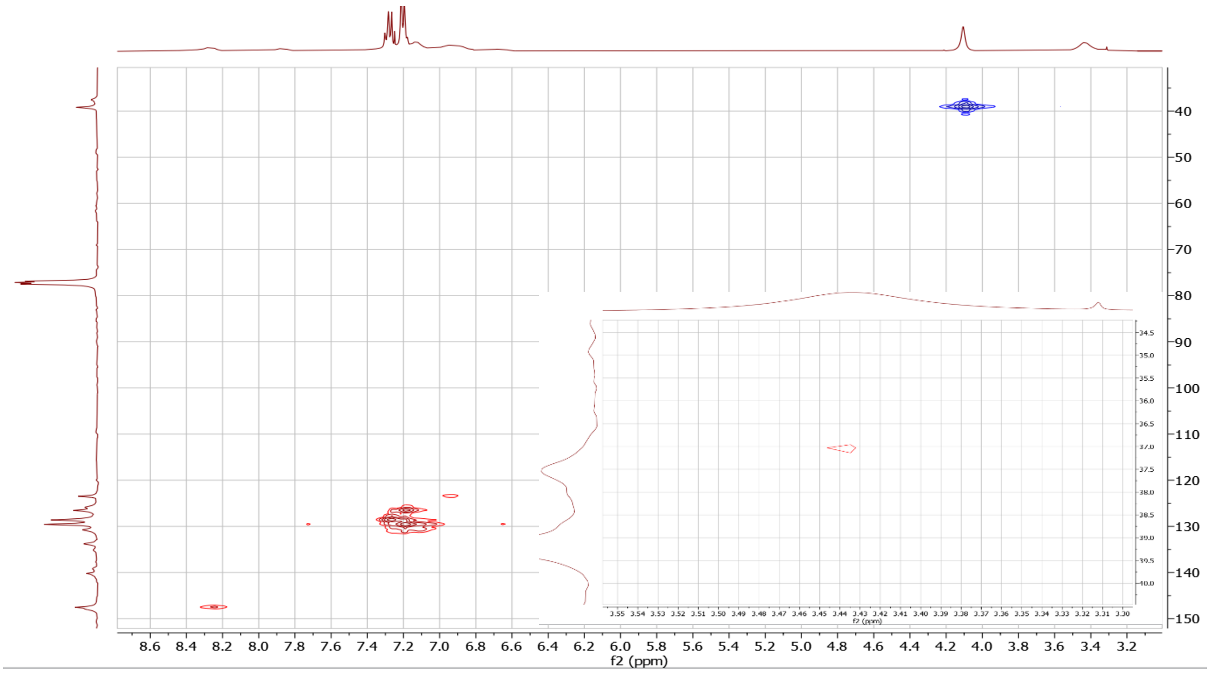

^{1}H NMR of **3l**

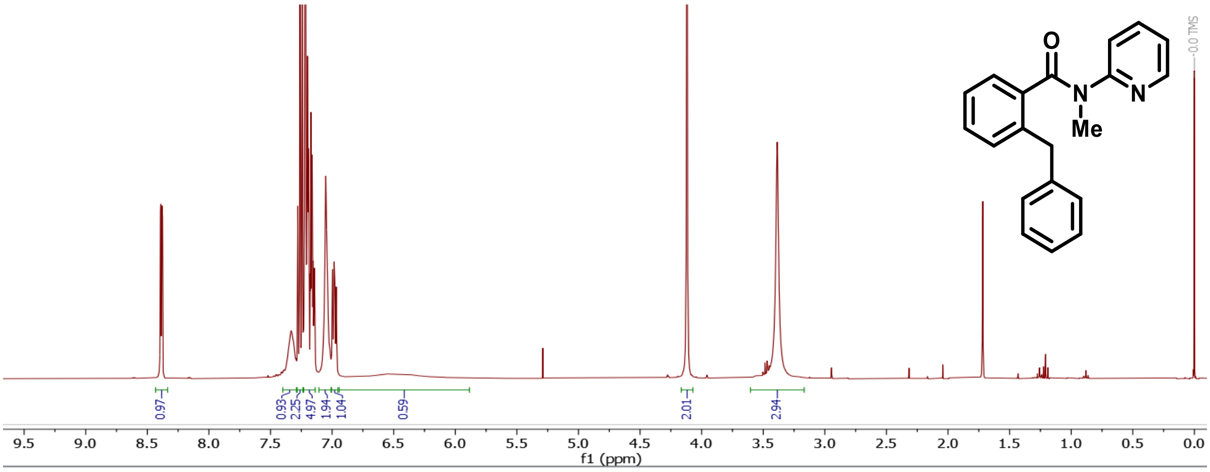

^{13}C NMR of **3l**

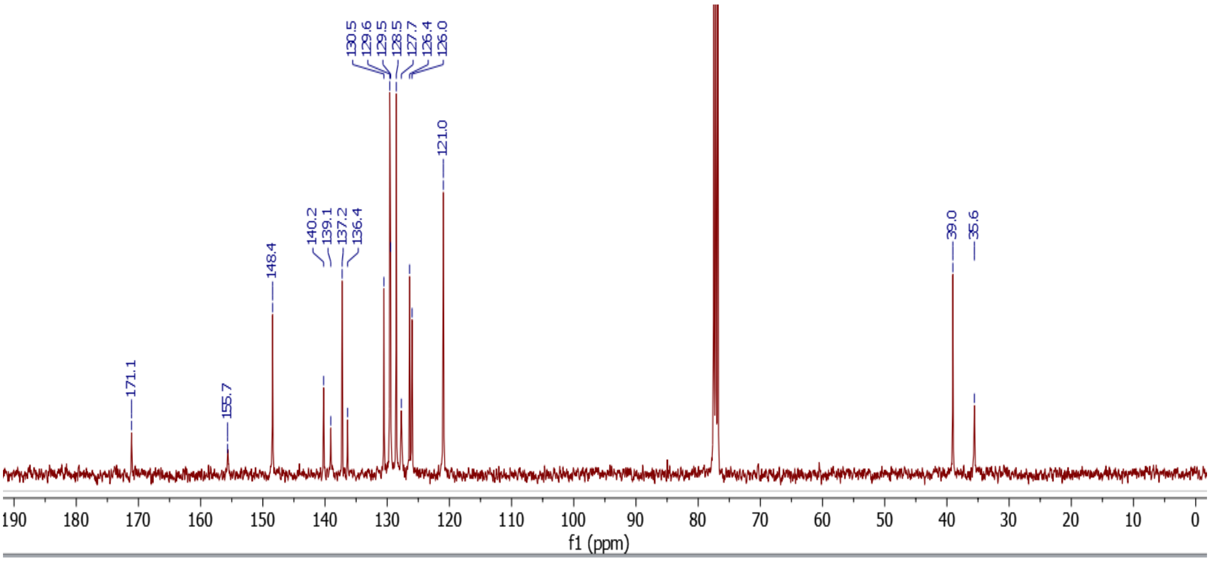

HSQC of **3l**

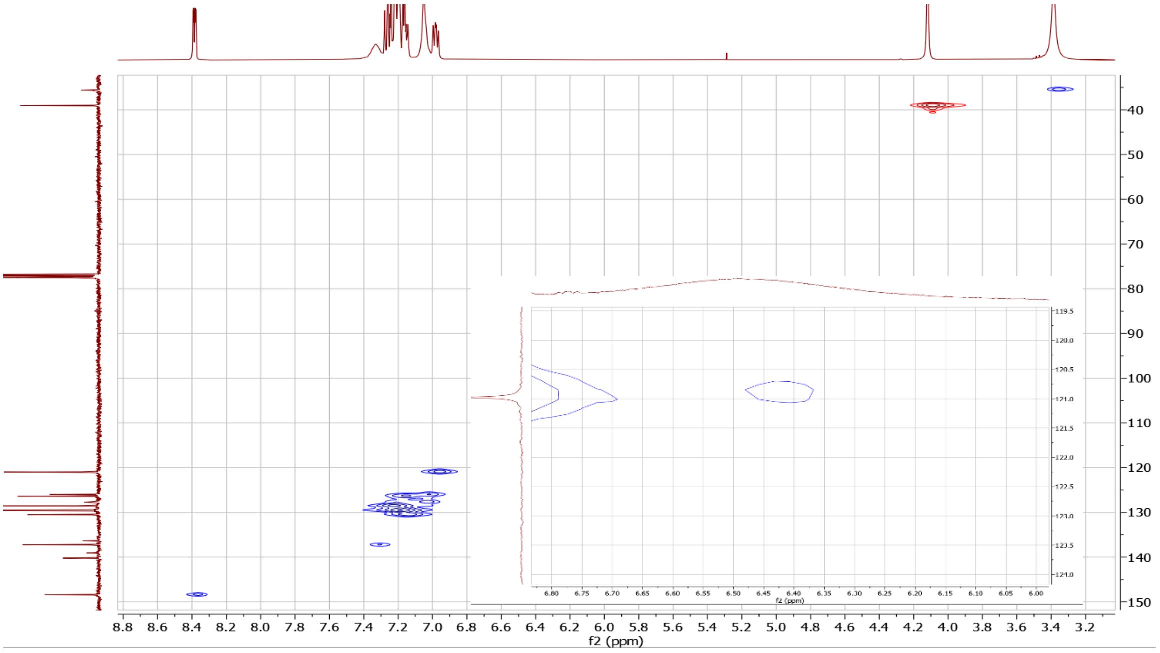

^{1}H NMR of **4a**

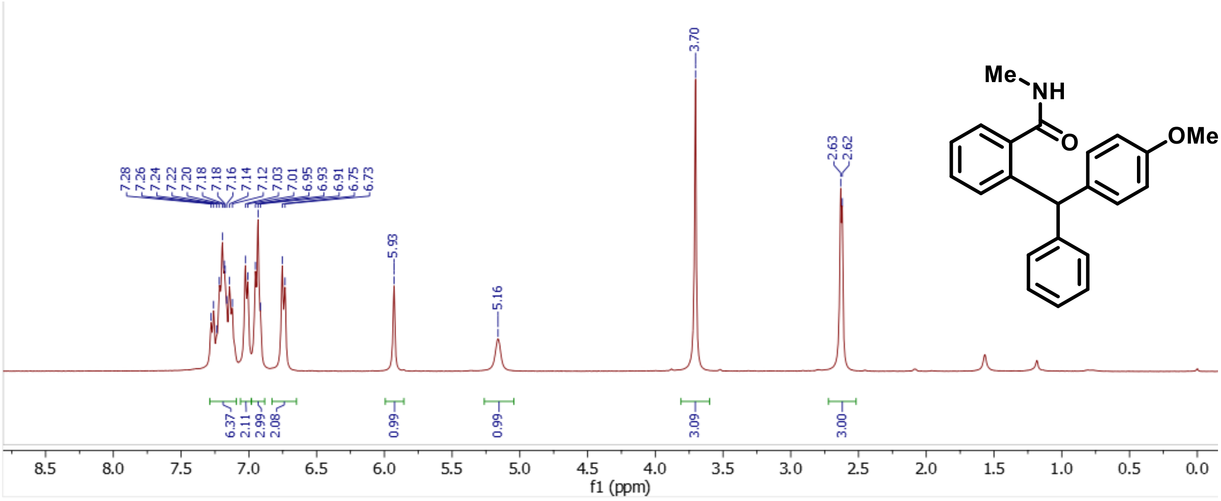

^{13}C NMR of **4a**

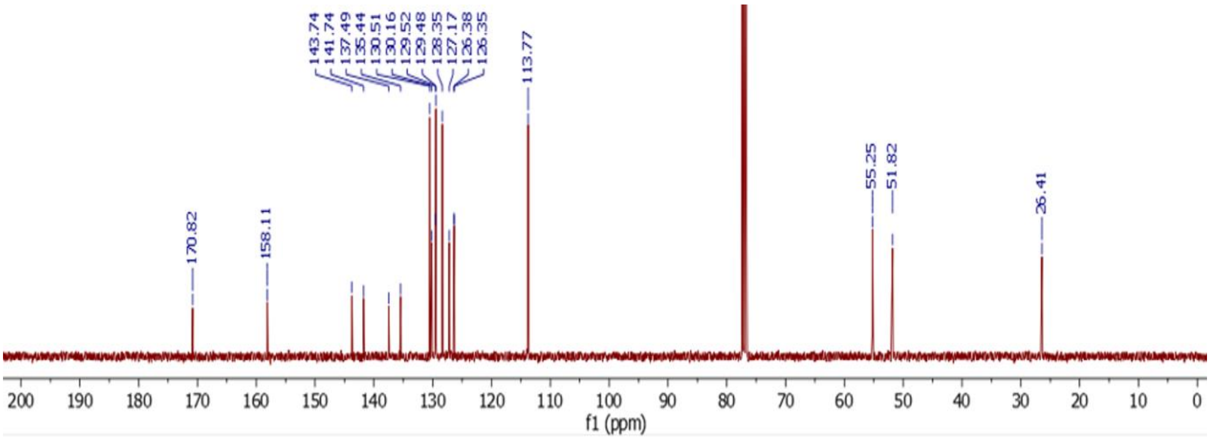

HSQC of **4a**

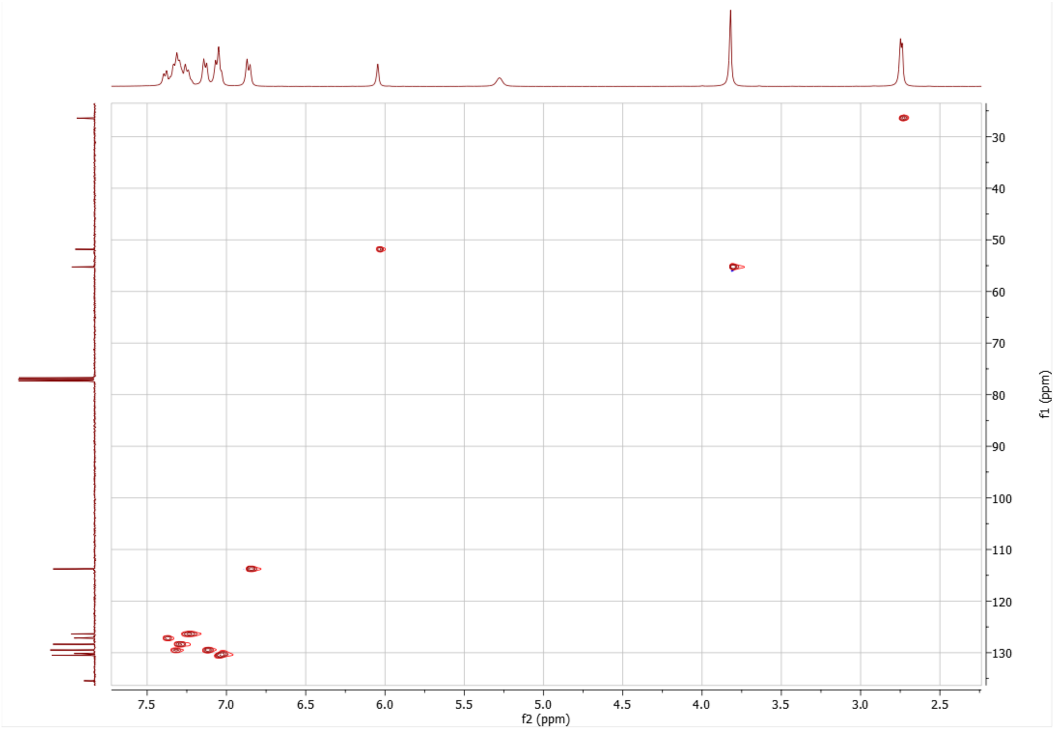

^{1}H NMR of **4b**

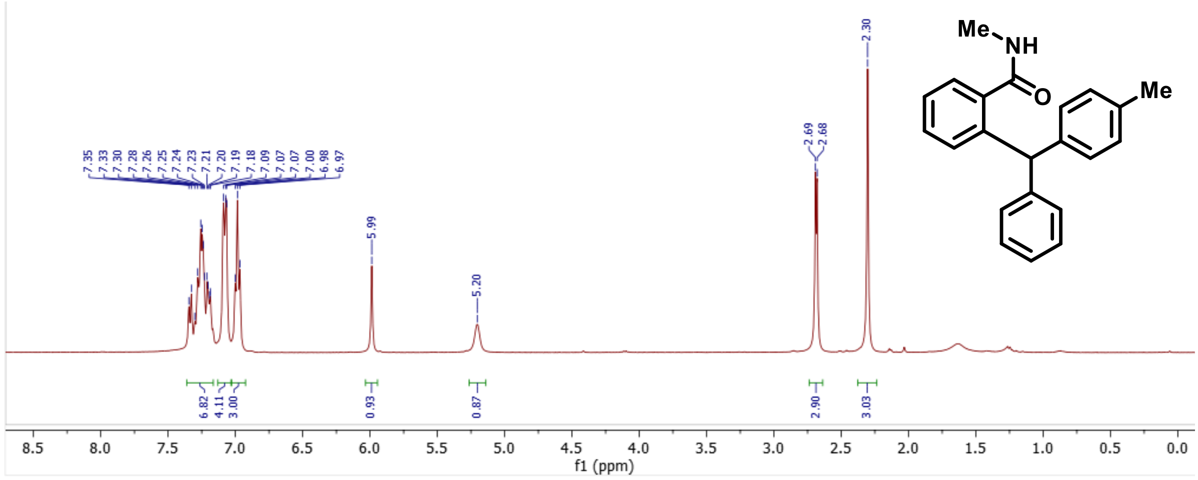

^{13}C NMR of **4b**

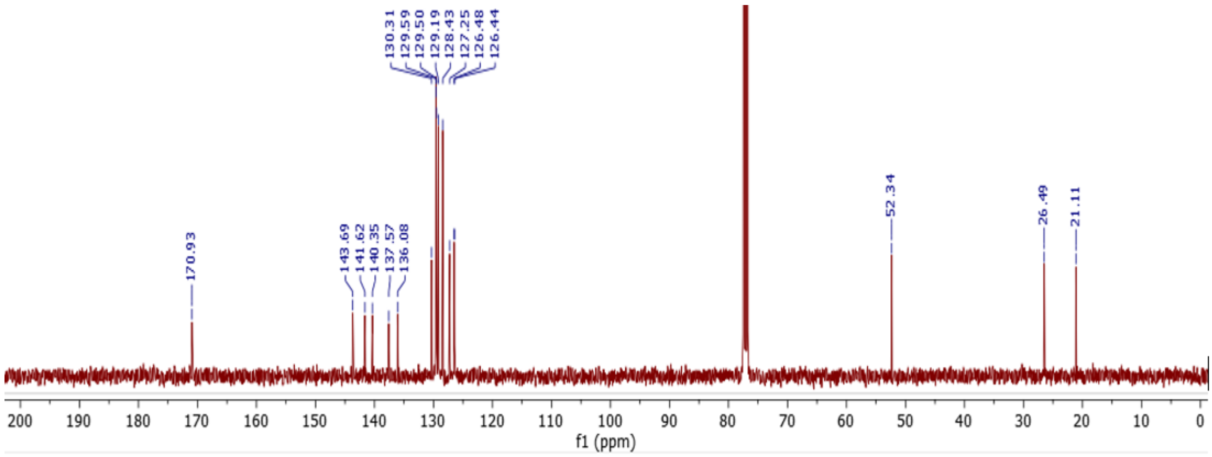

HSQC of **4b**

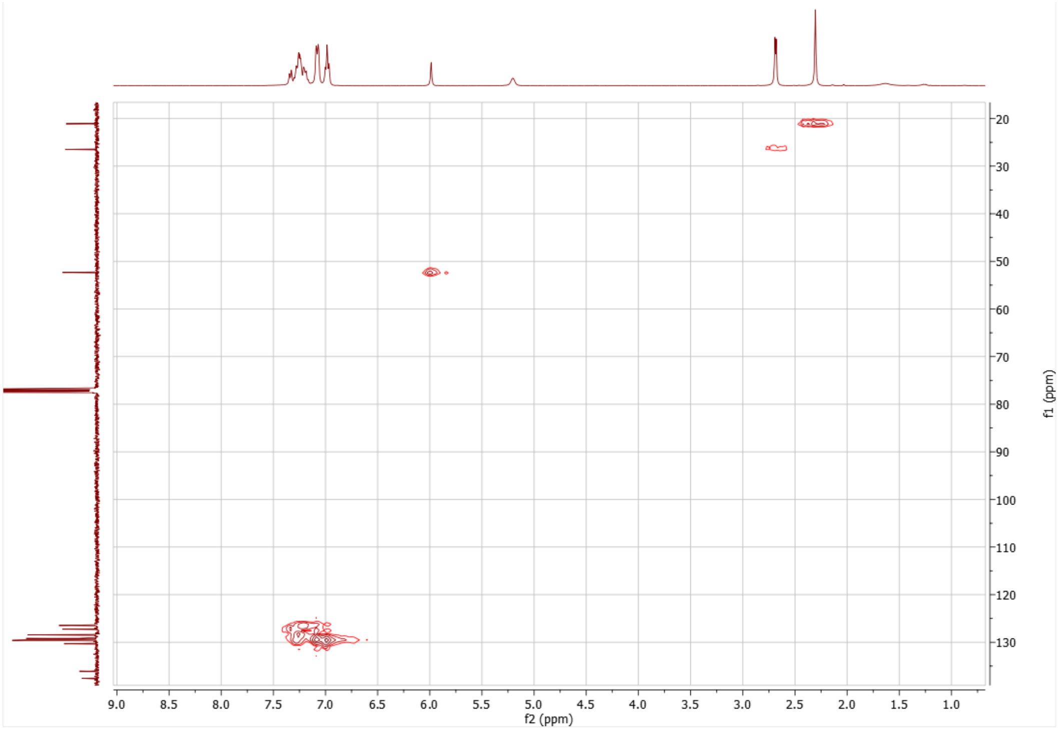

^{1}H NMR of **4c**

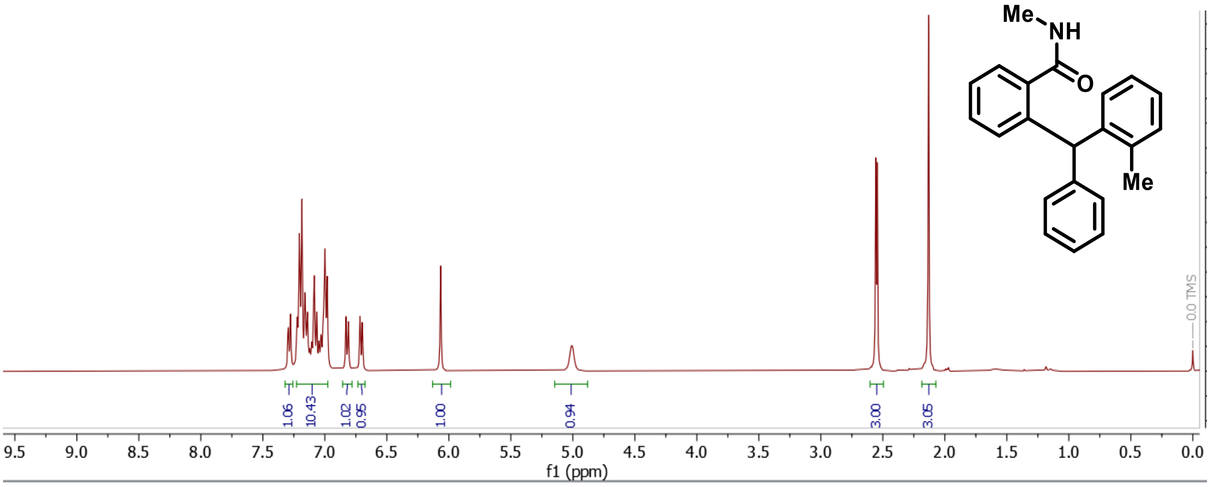

^{13}C NMR of **4c**

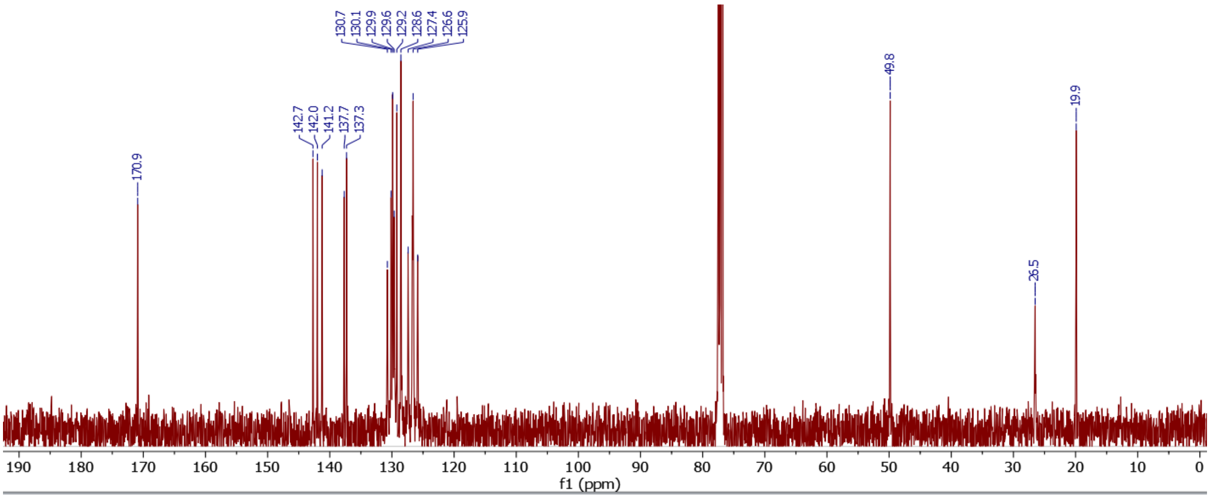

HSQC of **4c**

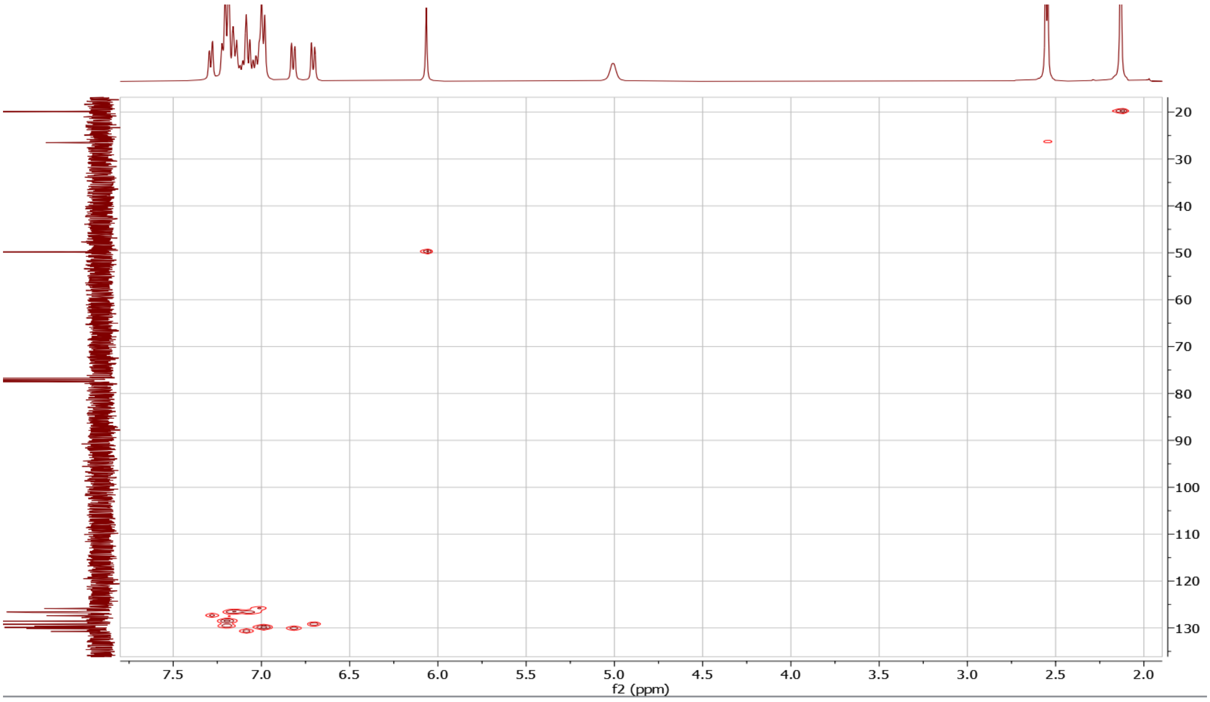

^{1}H NMR of **4d**

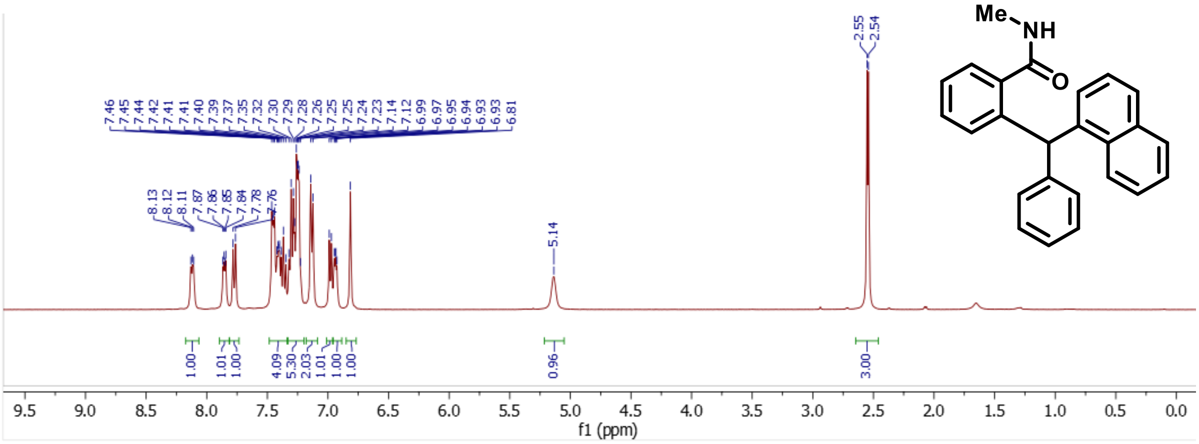

^{13}C NMR of **4d**

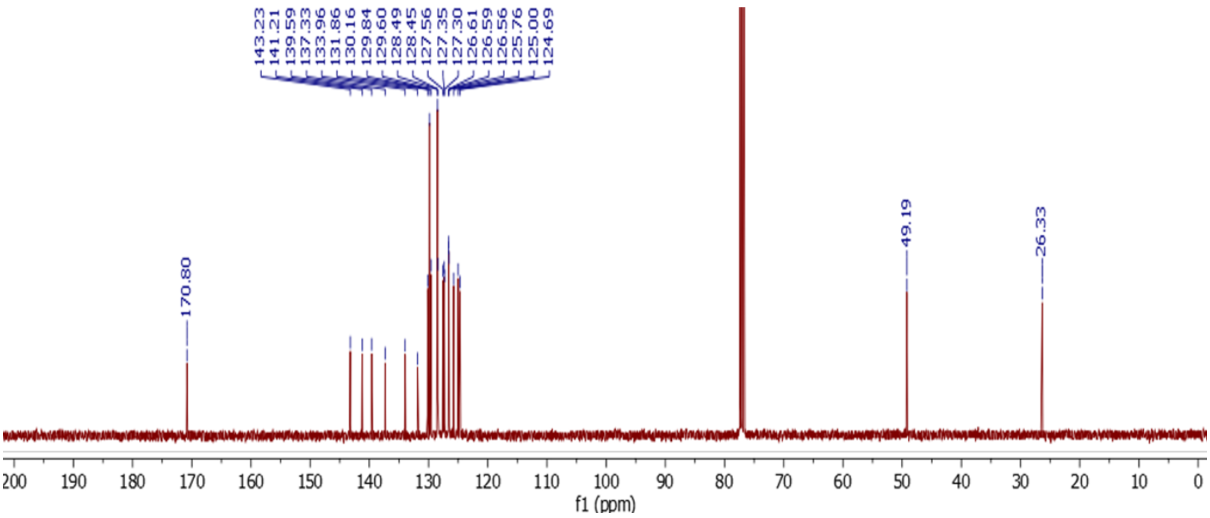

HSQC of **4d**

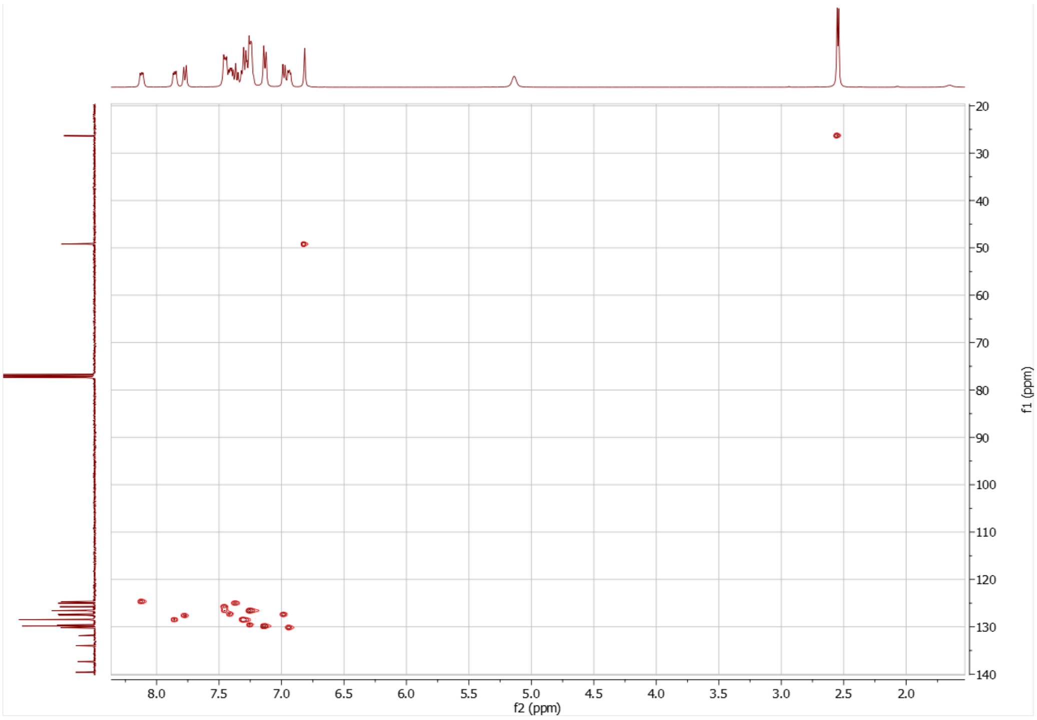

^{1}H NMR of **4e**

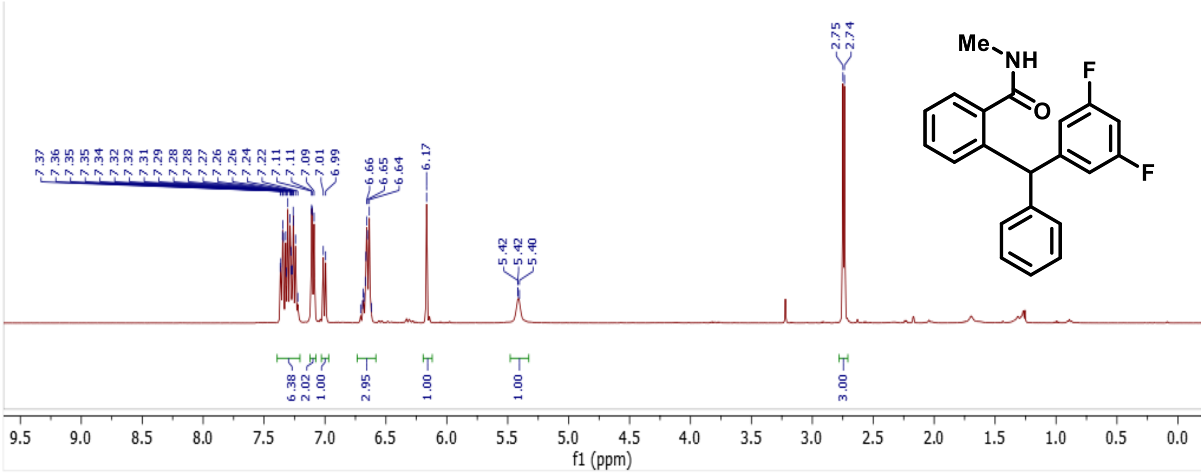

^{13}C NMR of **4e**

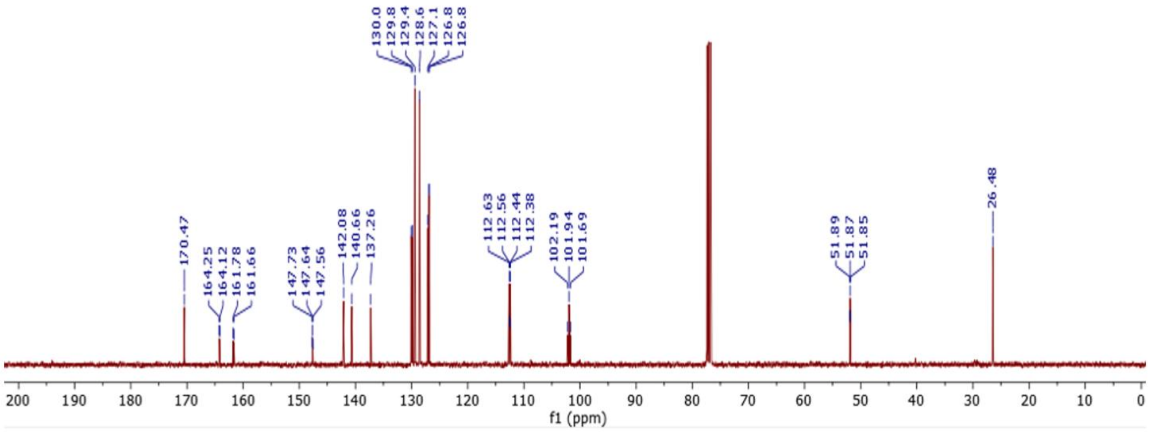

HSQC of **4e**

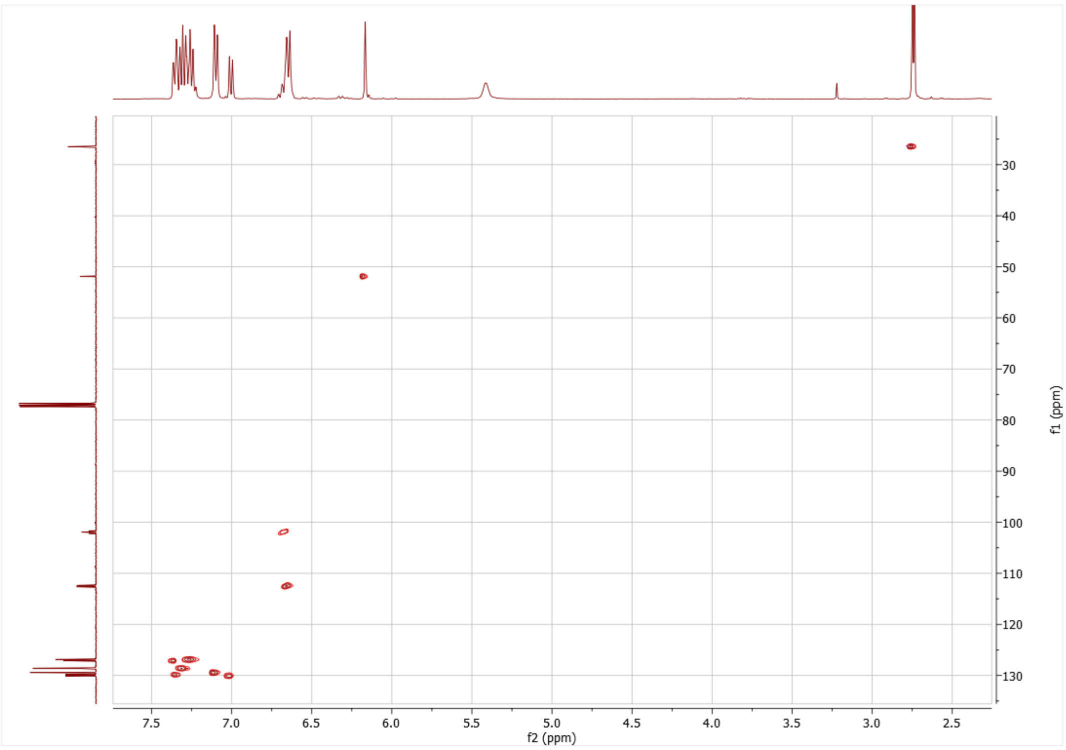

^{1}H NMR of **4f**

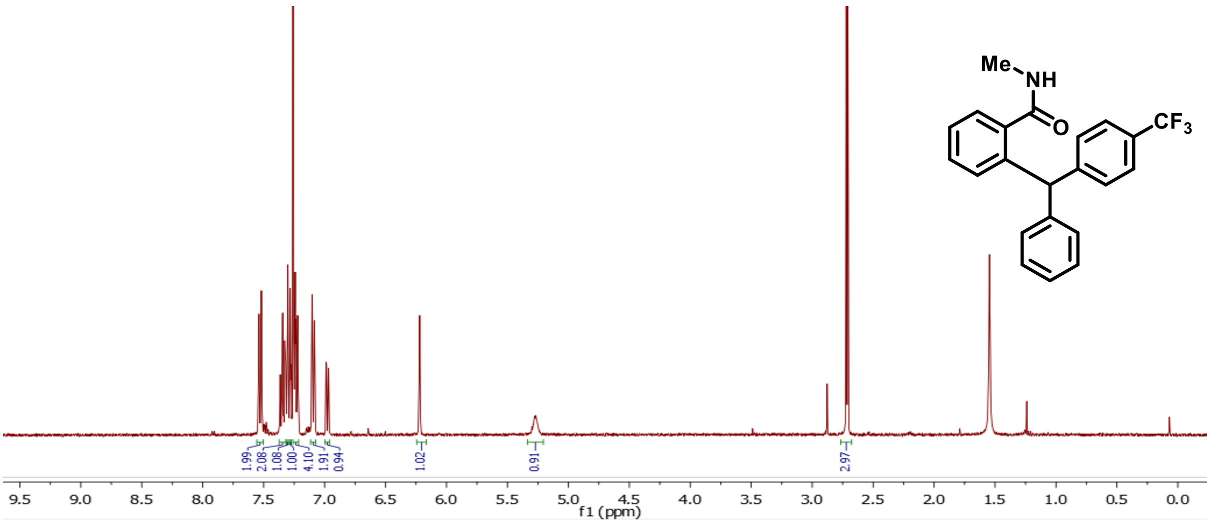

^{13}C NMR of **4f**

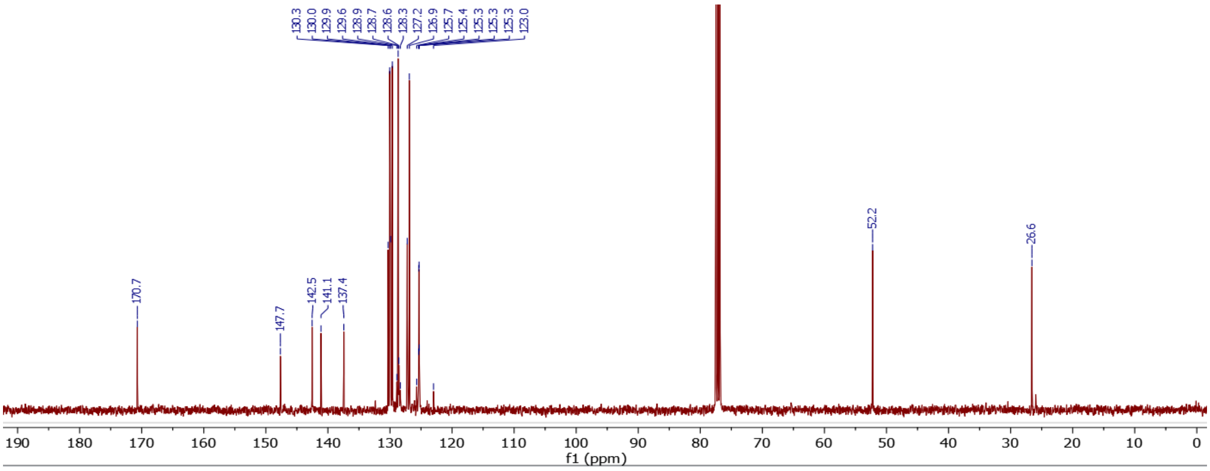

HSQC of **4f**

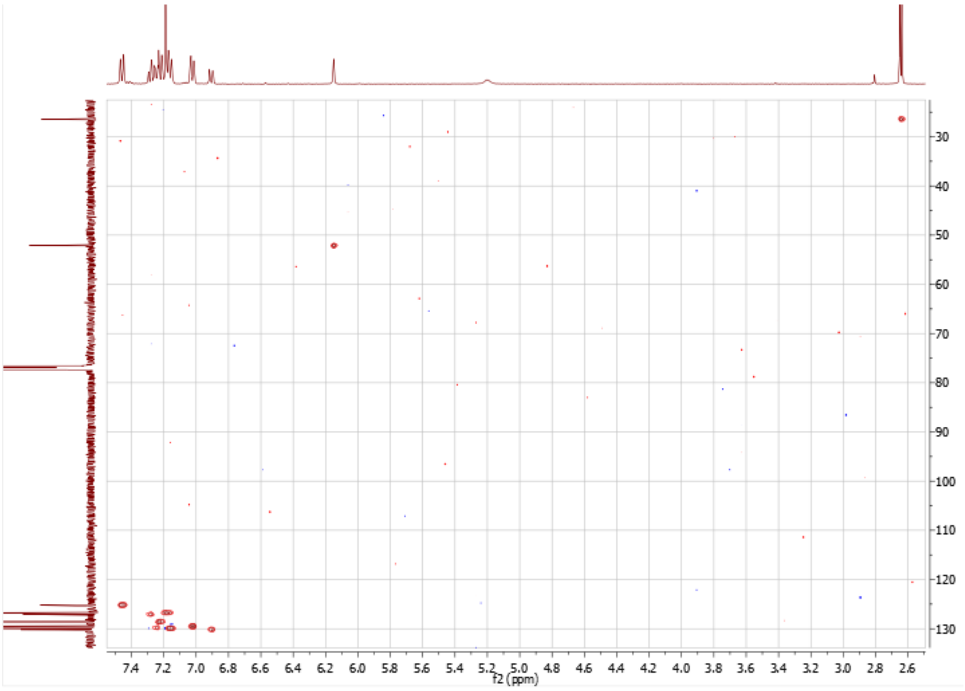

^1H NMR of **4g**

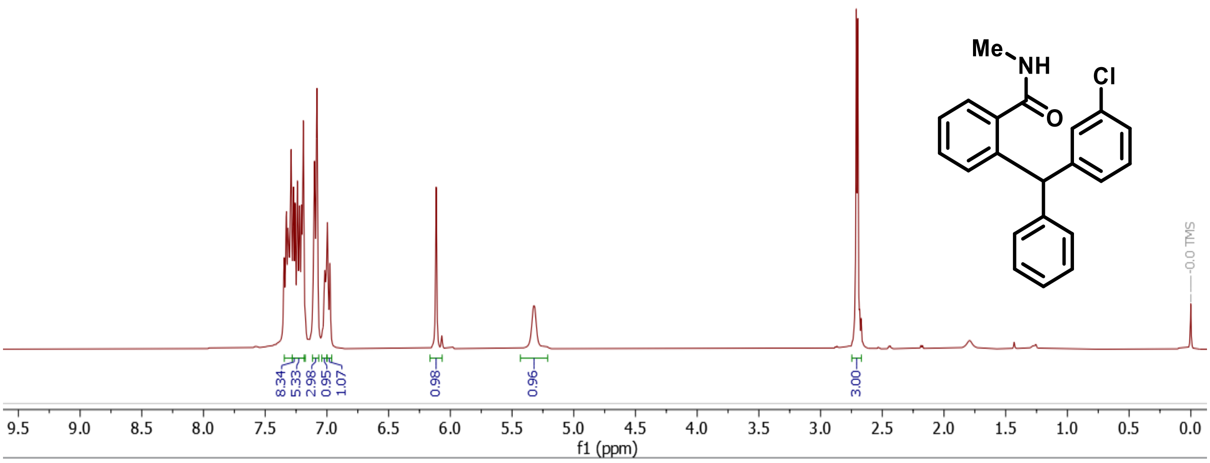

^{13}C NMR of **4g**

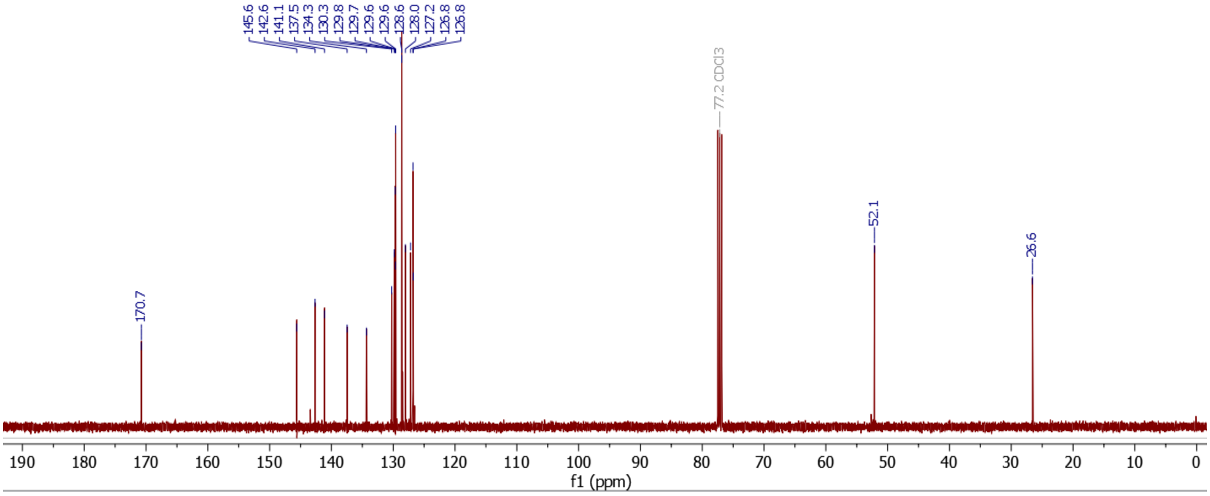

HSQC of **4g**

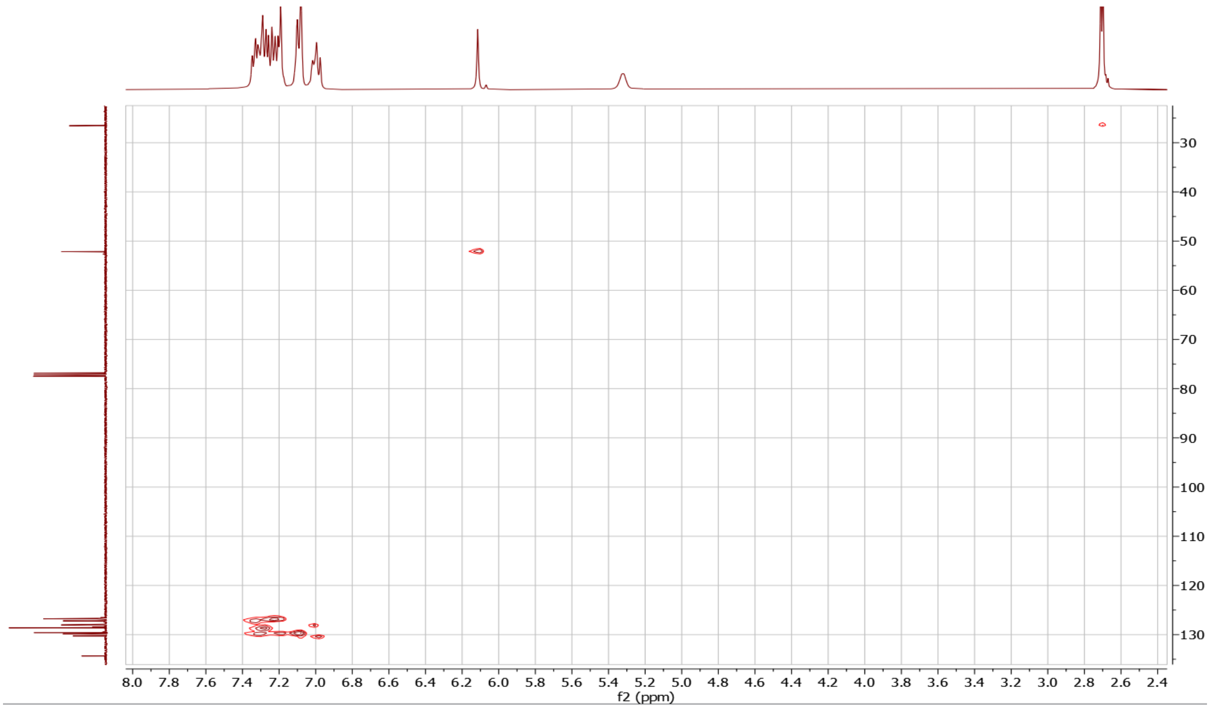

^{1}H NMR of **4h**

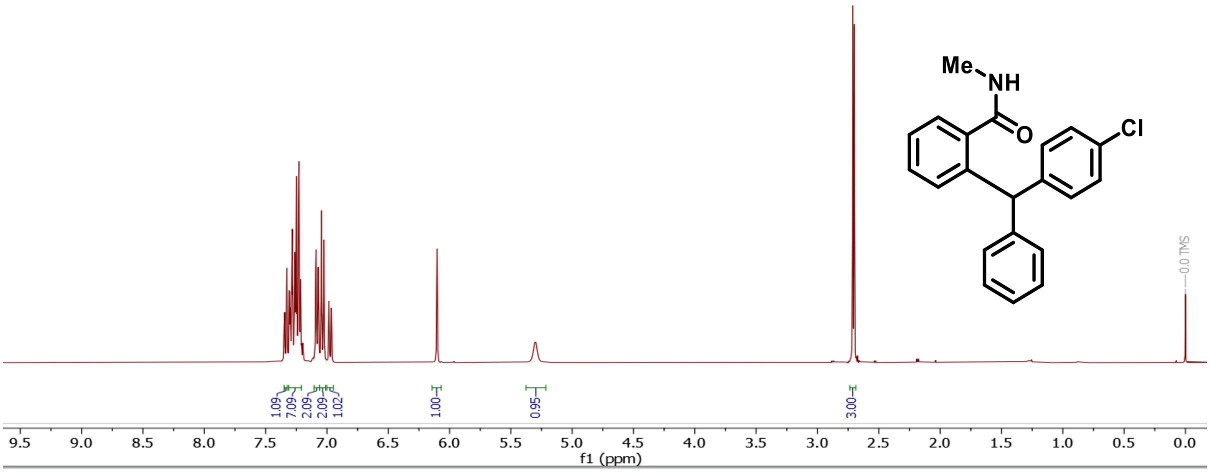

^{13}C NMR of **4h**

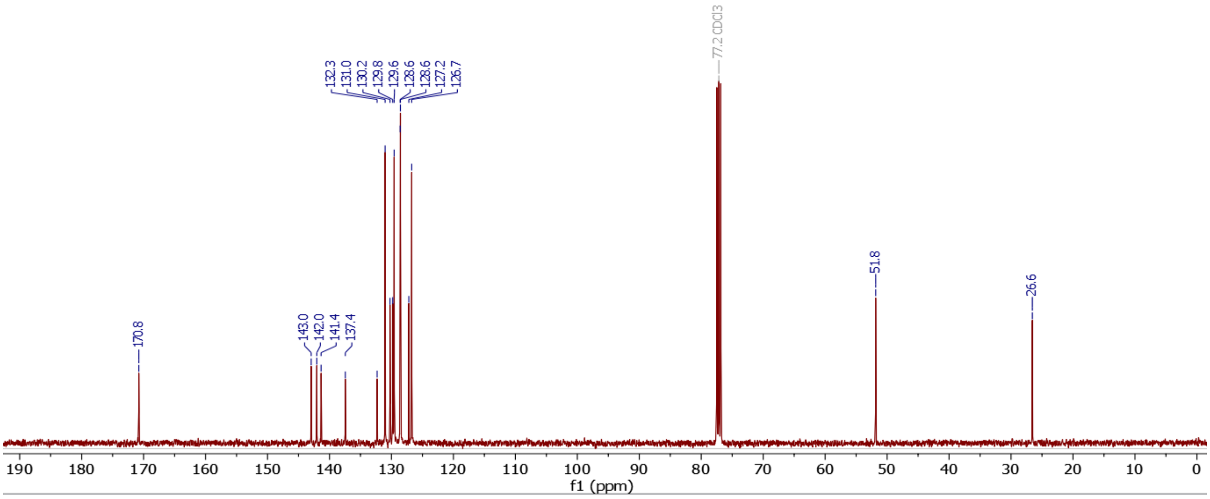

HSQC of **4h**

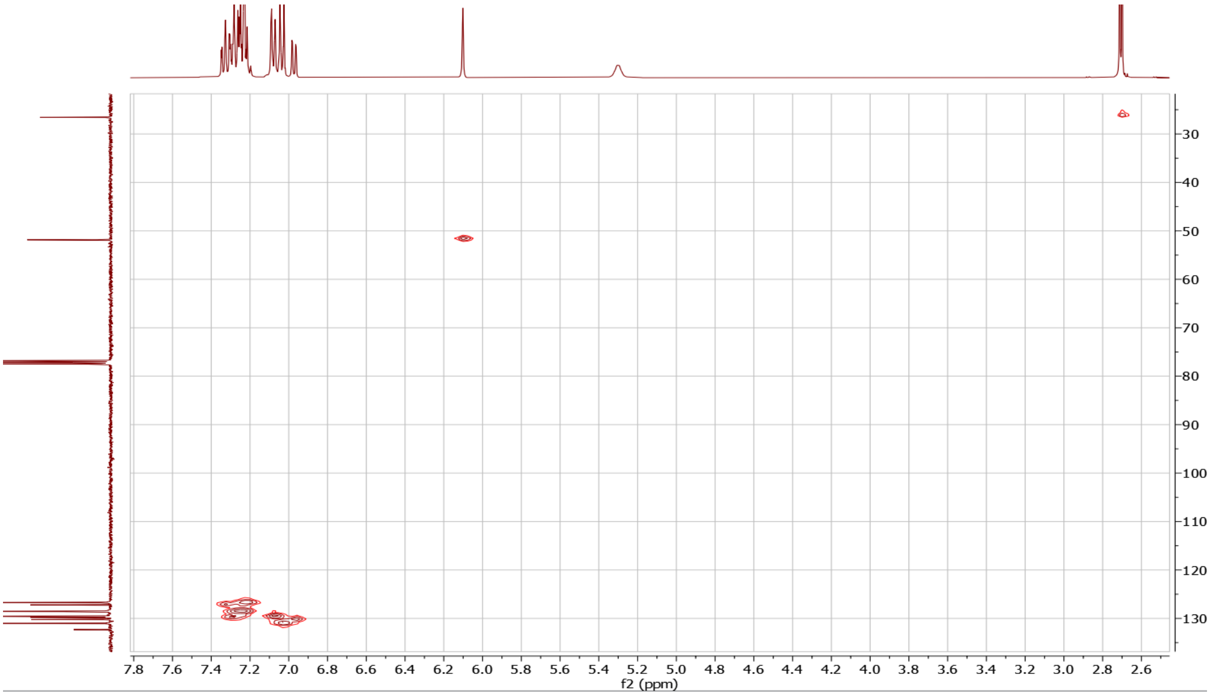

^{1}H NMR of **4i**

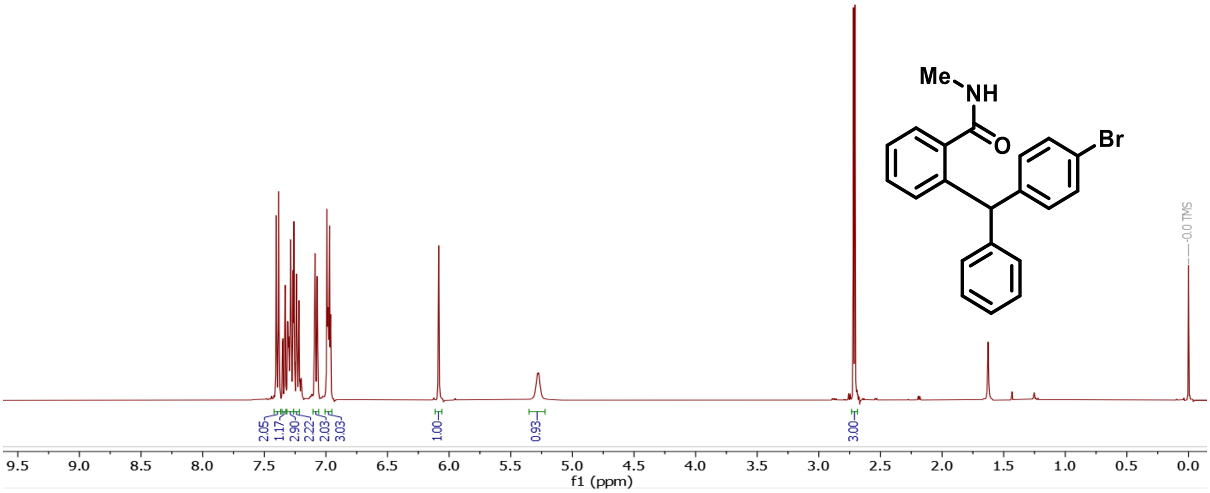

^{13}C NMR of **4i**

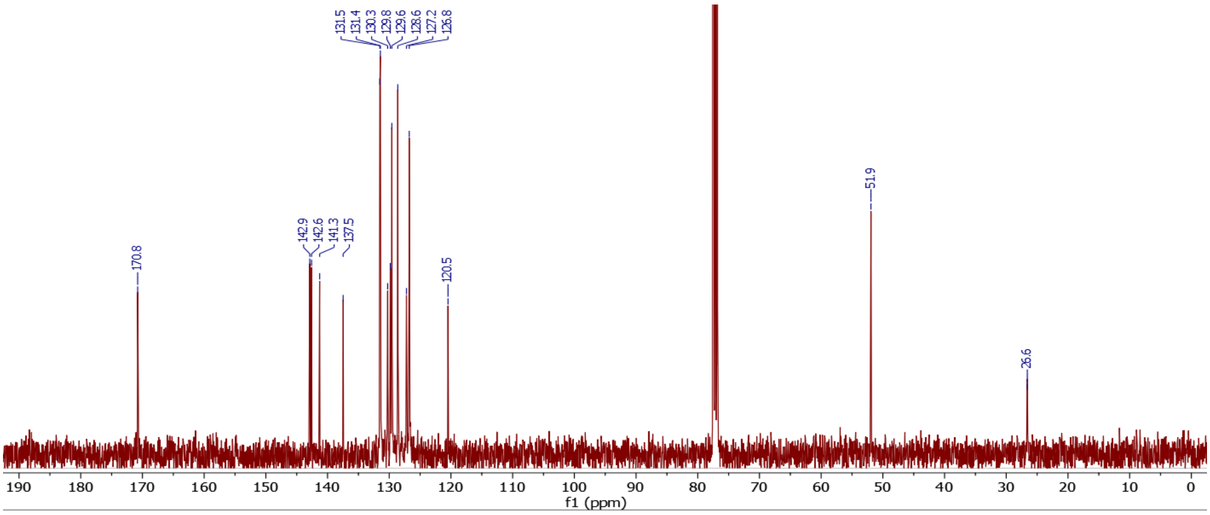

HSQC of **4i**

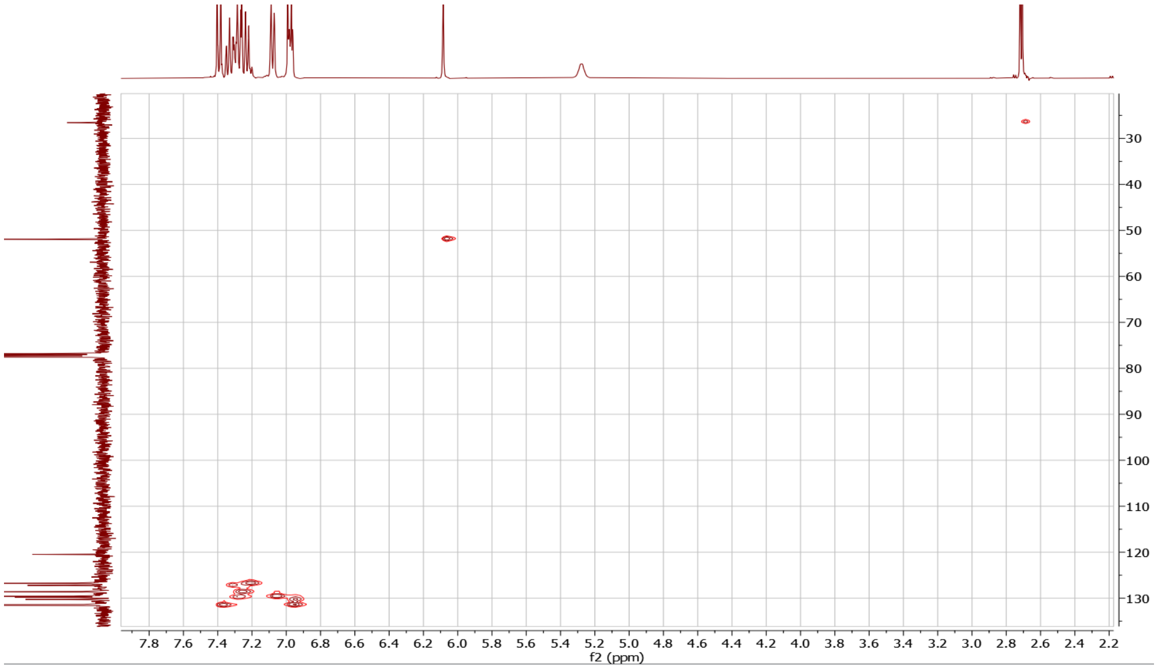

^{1}H NMR of **4j**

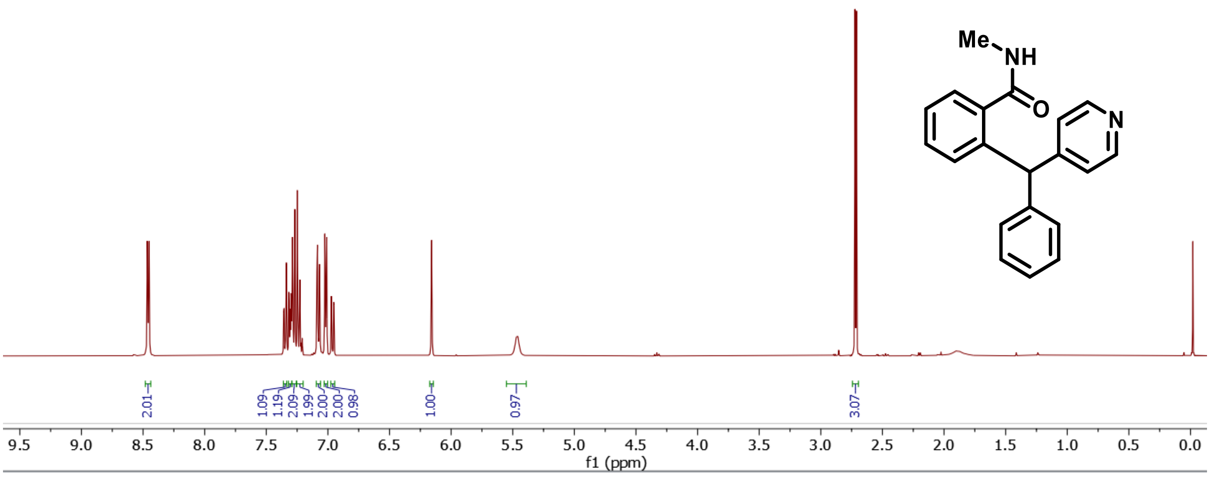

^{13}C NMR of **4j**

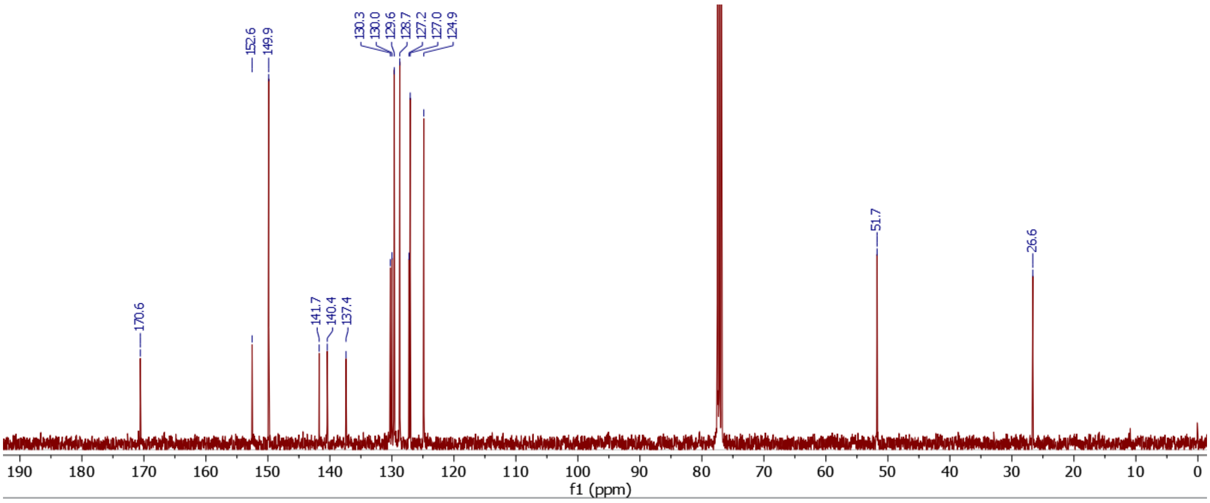

HSQC of **4j**

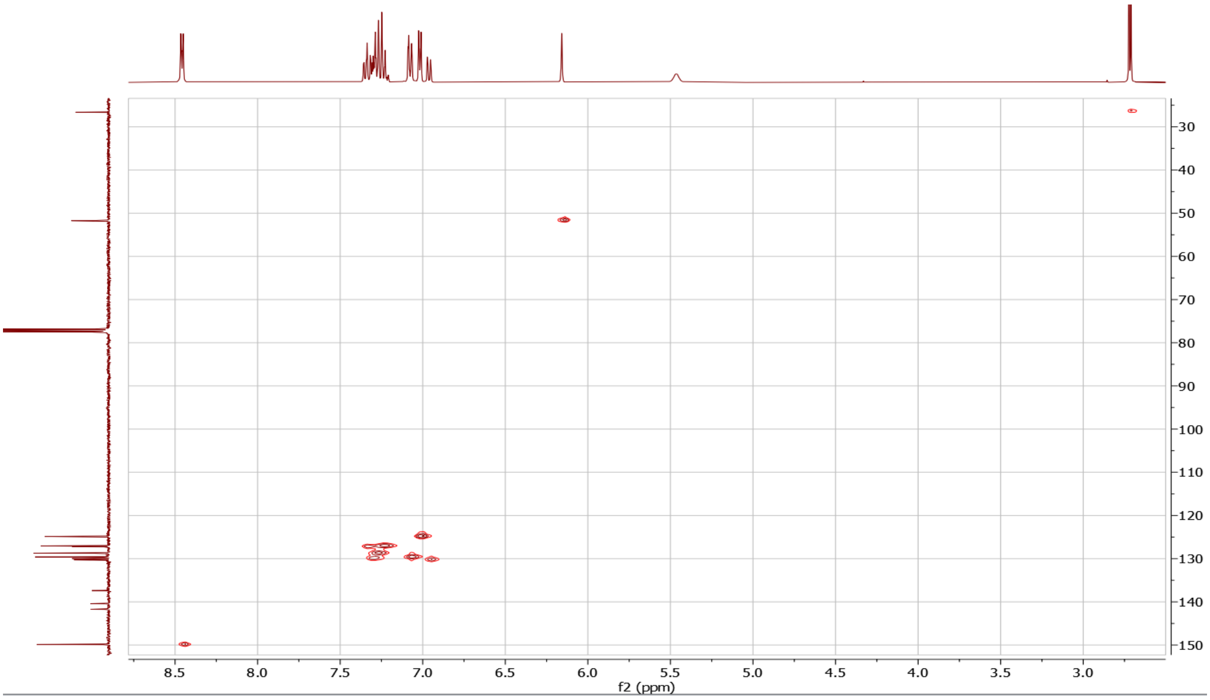

^{1}H NMR of **4k**

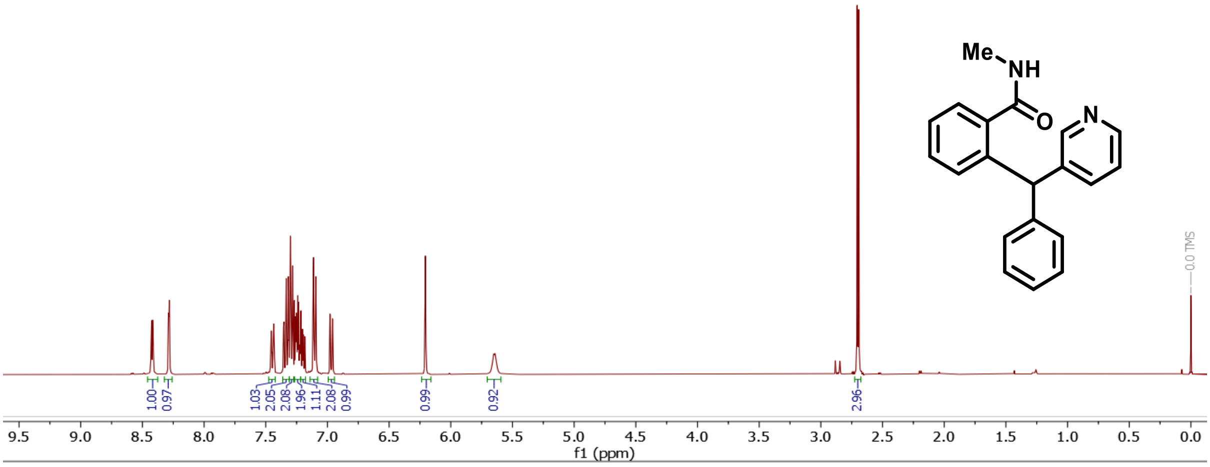

^{13}C NMR of **4k**

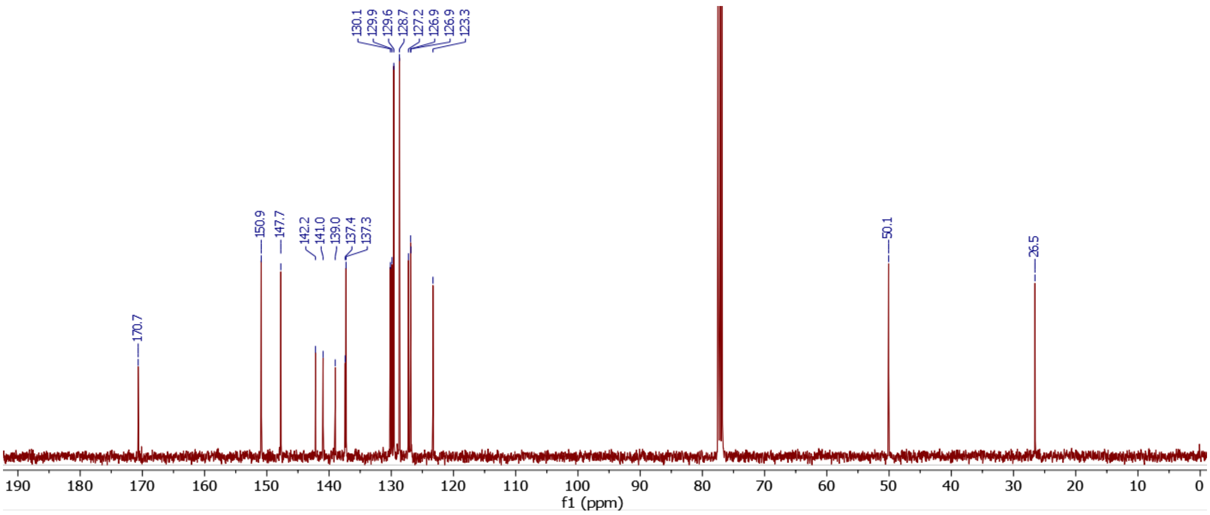

HSQC of **4k**

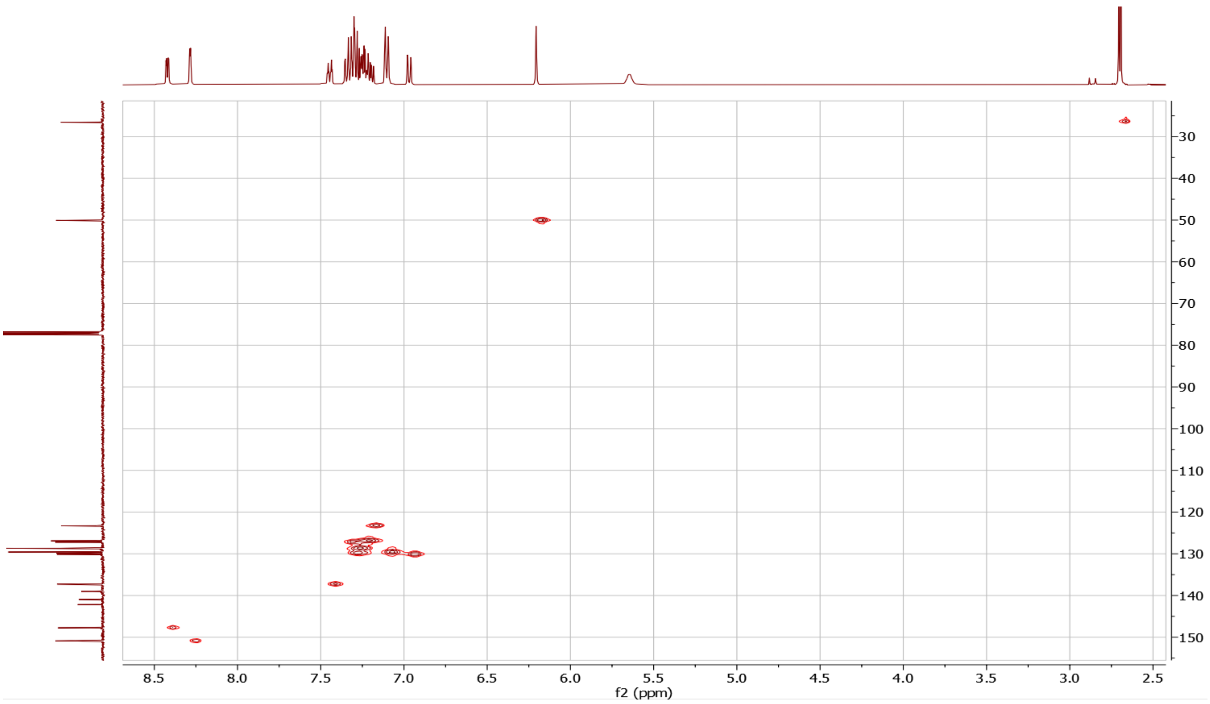

^{1}H NMR of **4l**

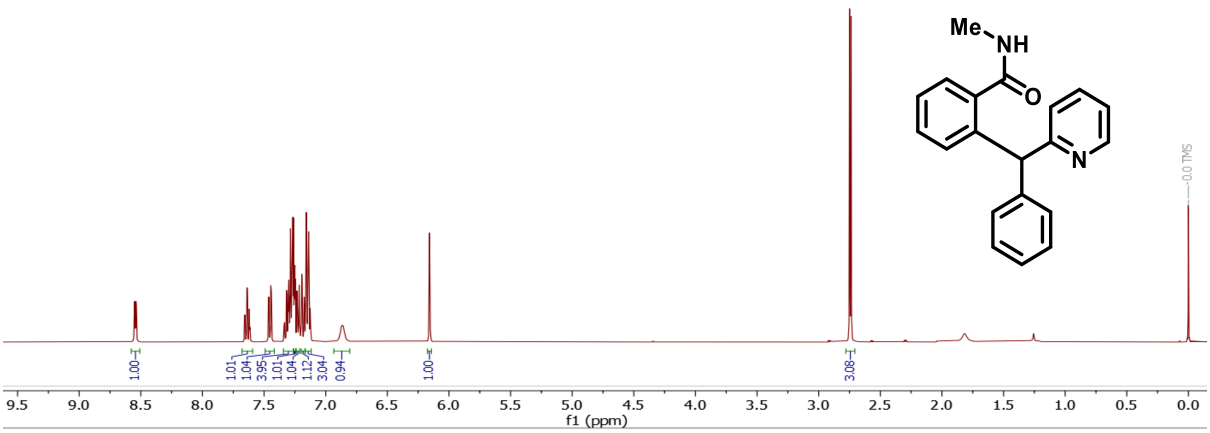

^{13}C NMR of **4l**

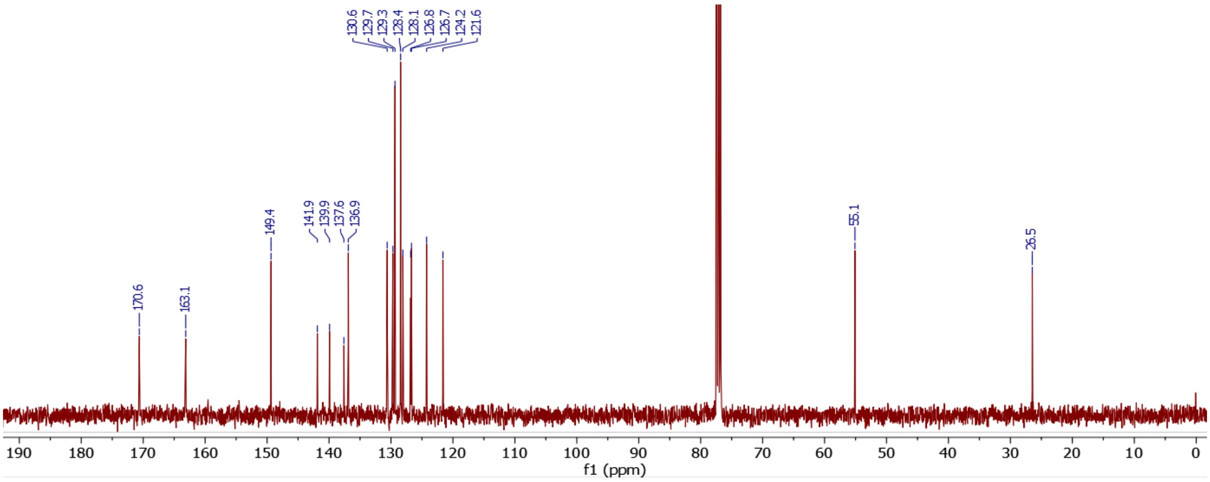

HSQC of **4l**

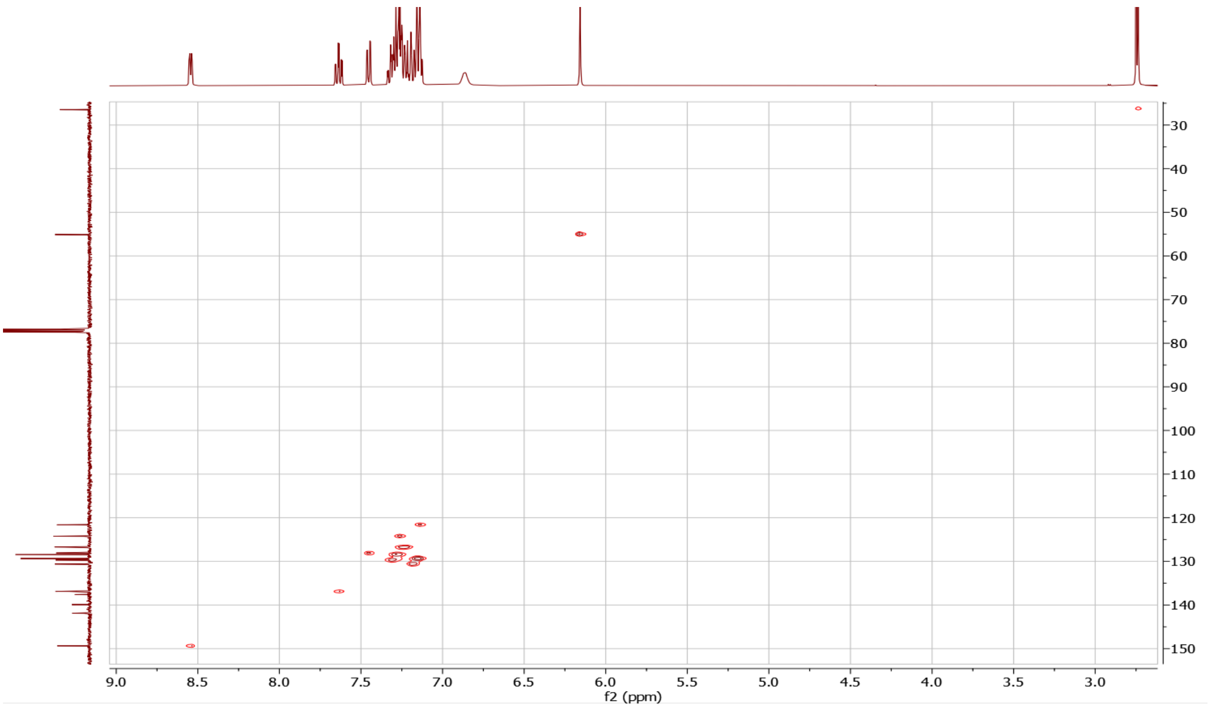

^{1}H NMR of **5a**

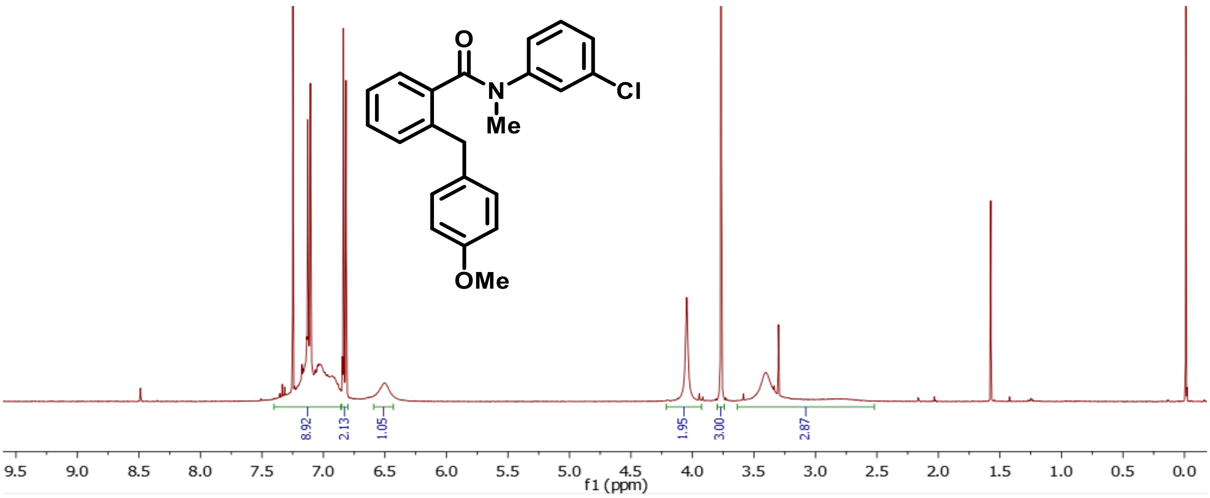

^{13}C NMR of **5a**

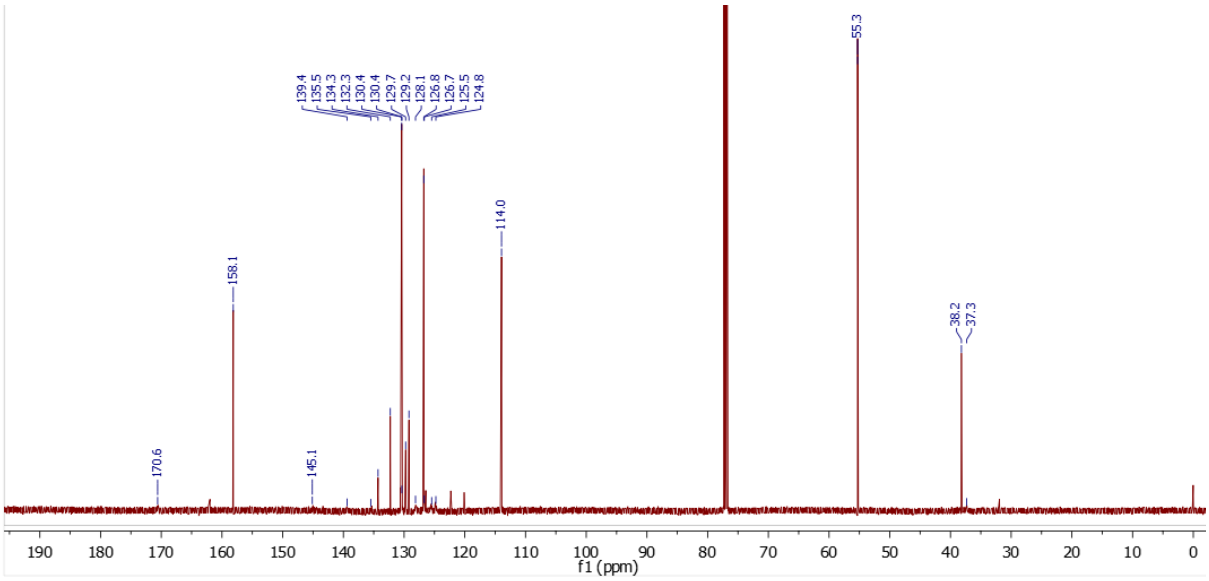

HSQC of **5a**

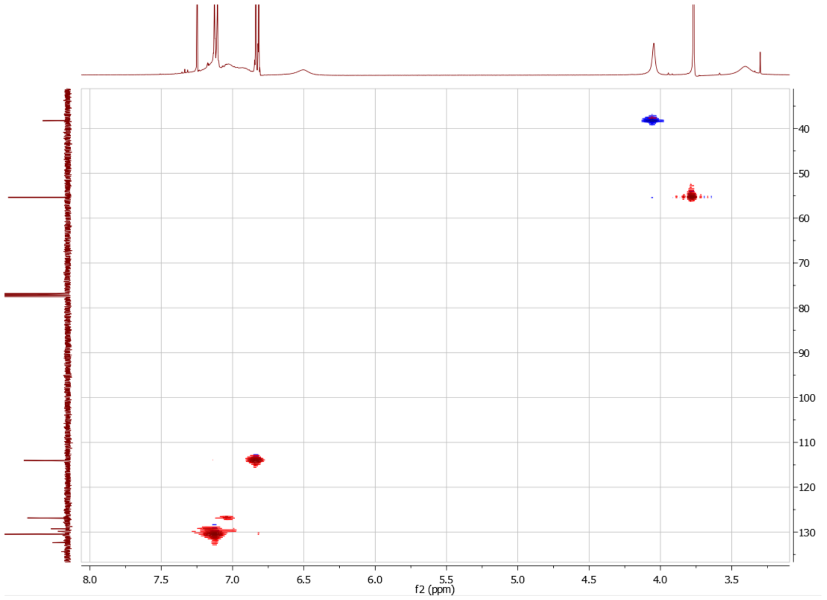

^{1}H NMR of **5b**

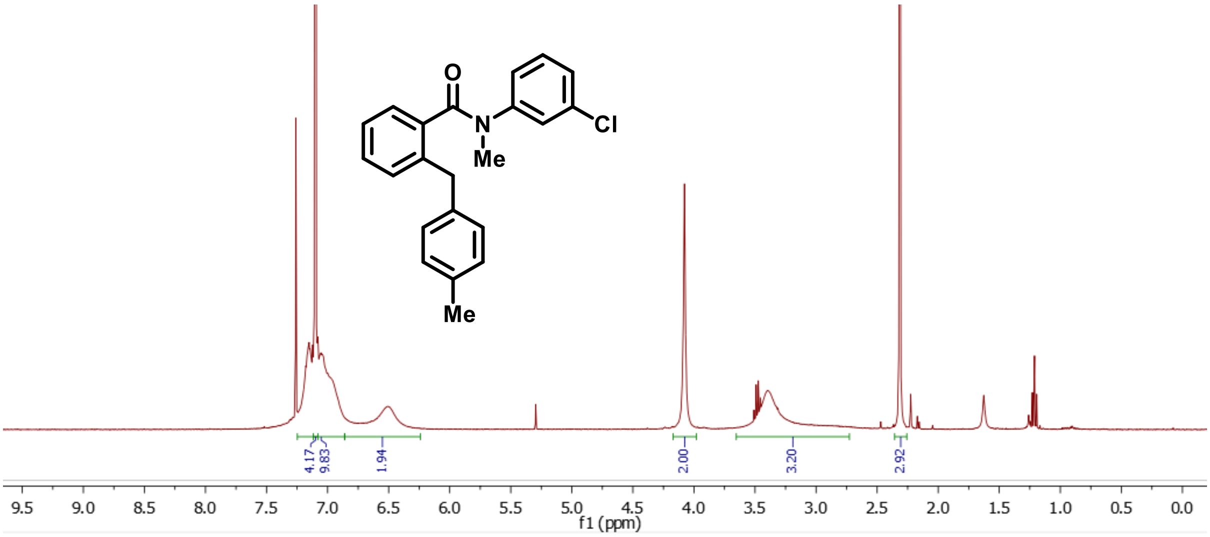

^{13}C NMR of **5b**

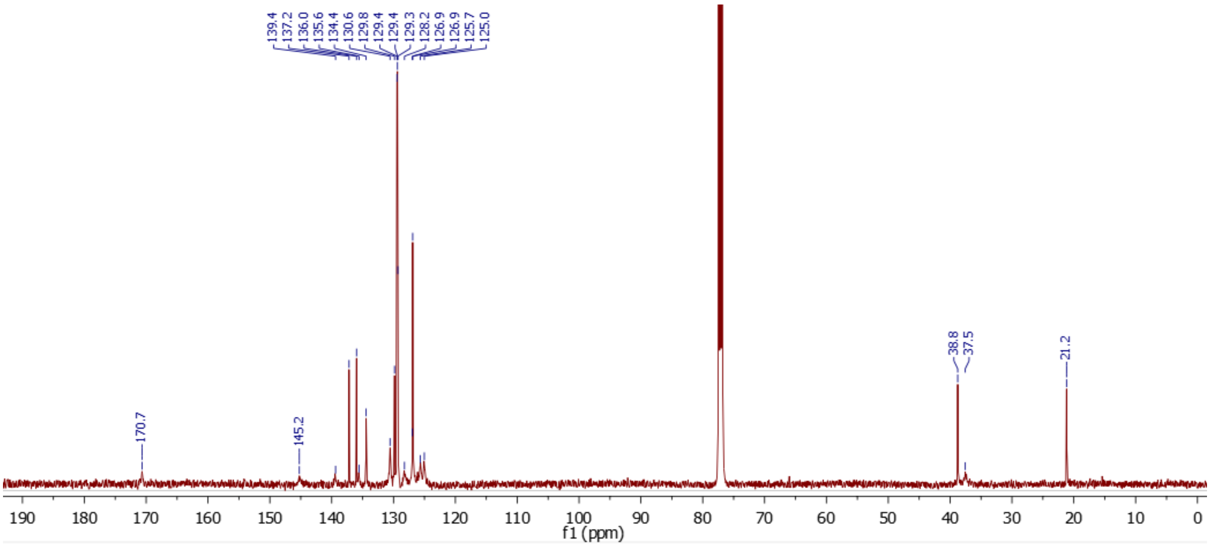

HSQC of **5b**

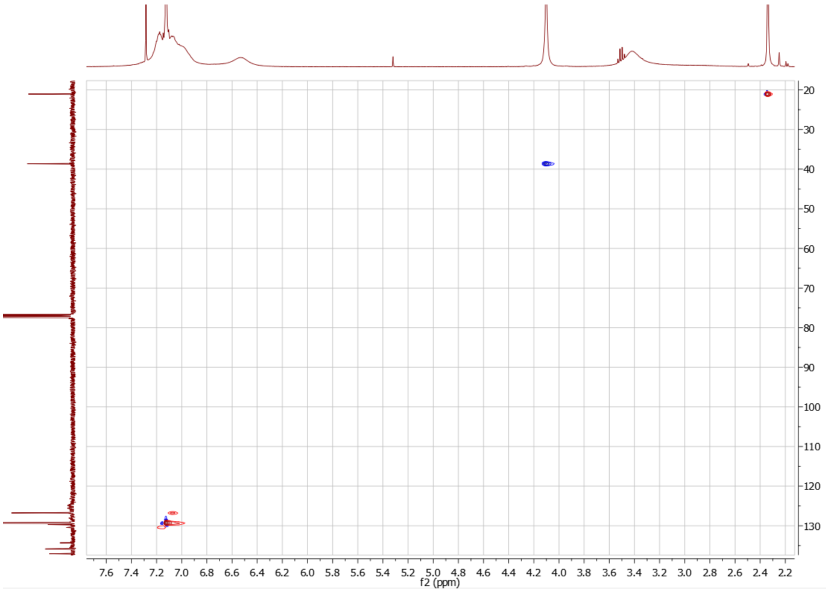

^{1}H NMR of **5c**

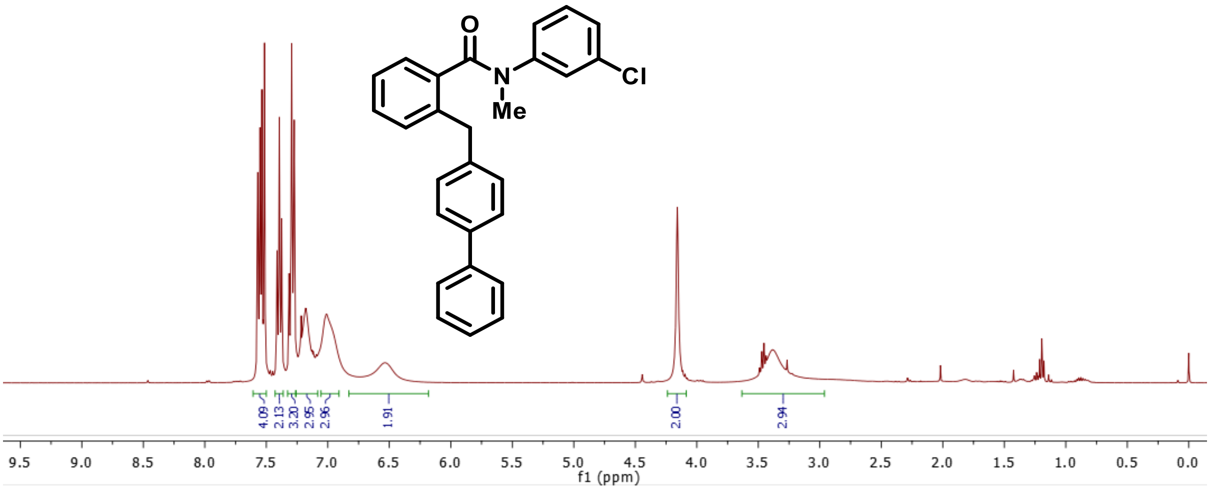

^{13}C NMR of **5c**

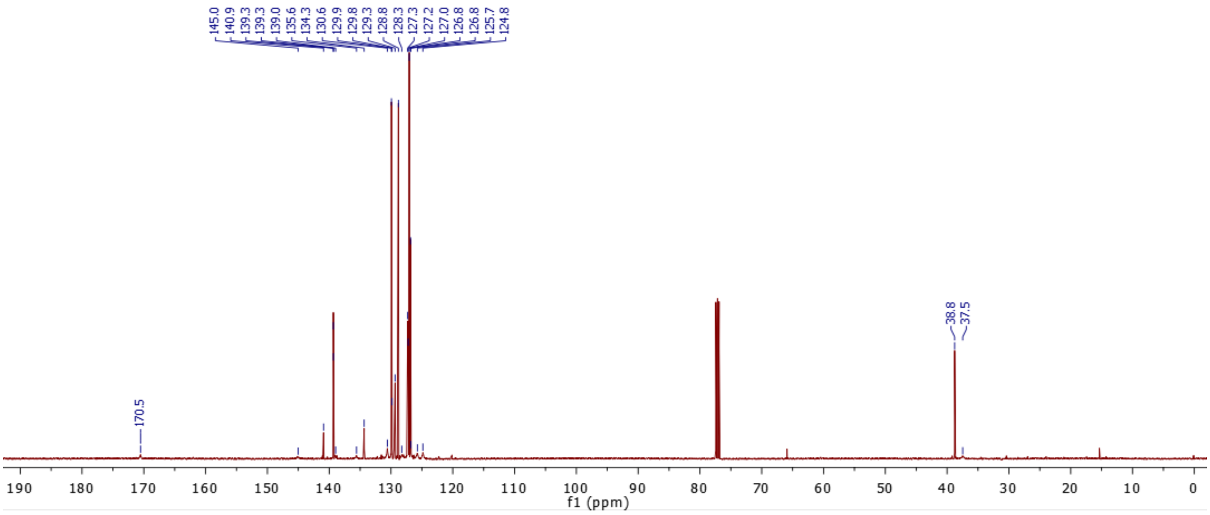

HSQC of **5c**

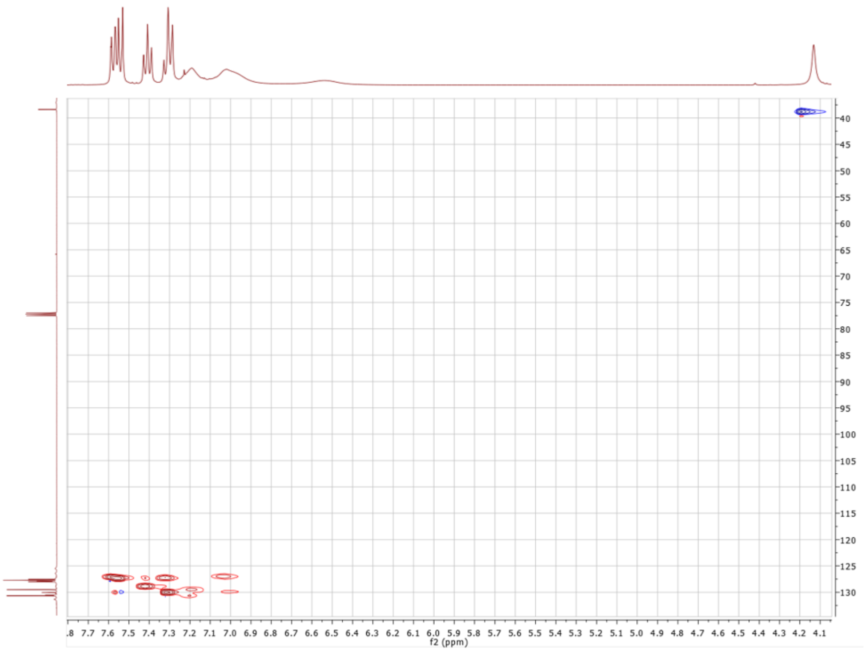

^{1}H NMR of **5d**

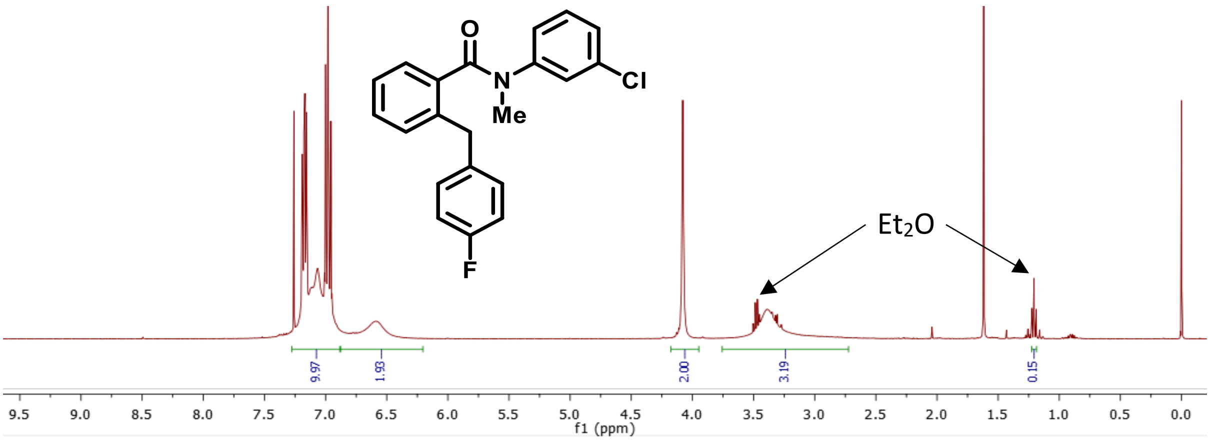

^{13}C NMR of **5d**

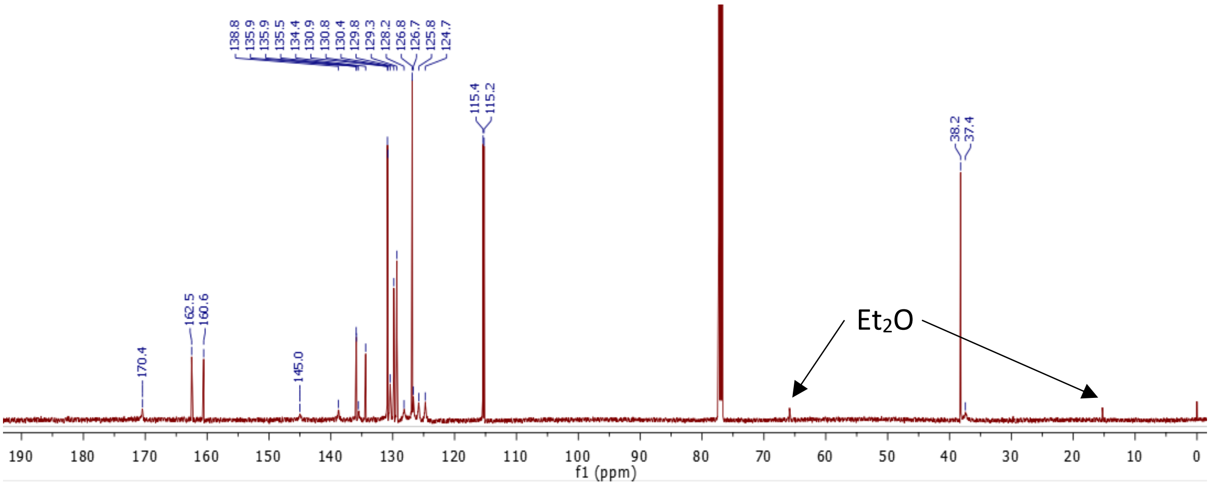

HSQC of **5d**

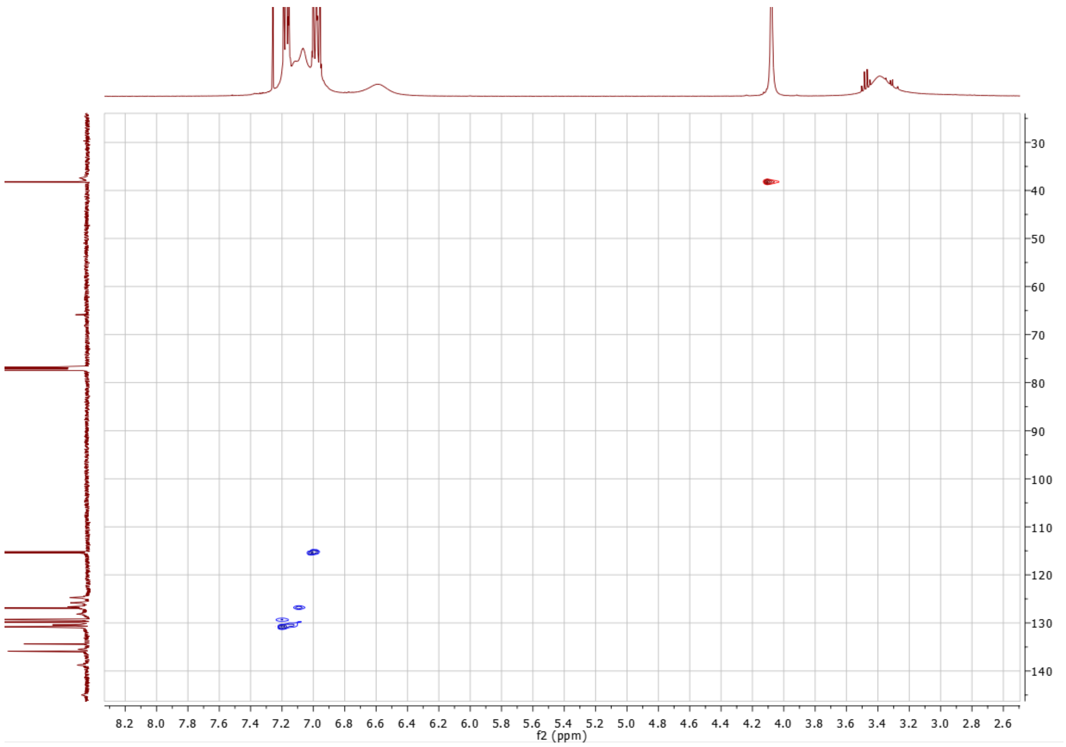

^{1}H NMR of **5e**

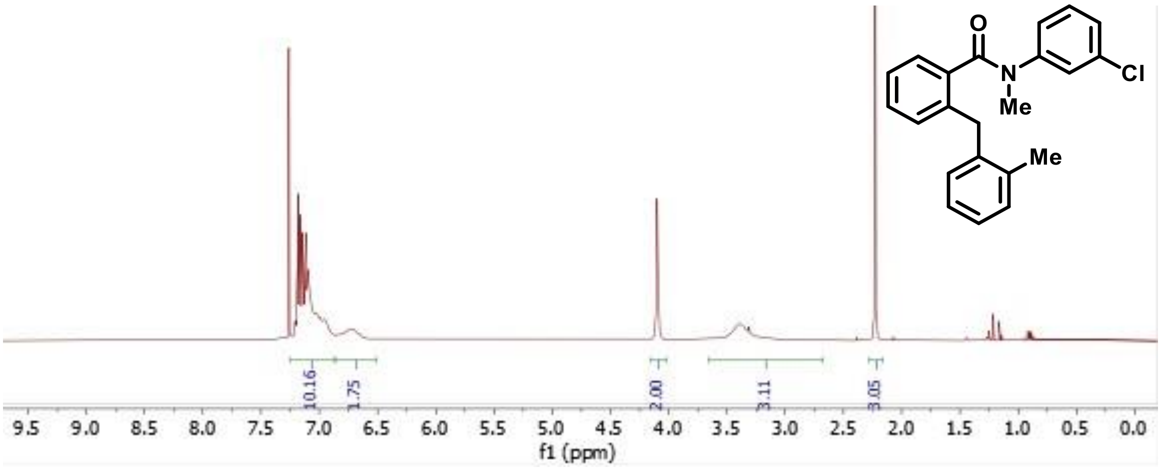

^{13}C NMR of **5e**

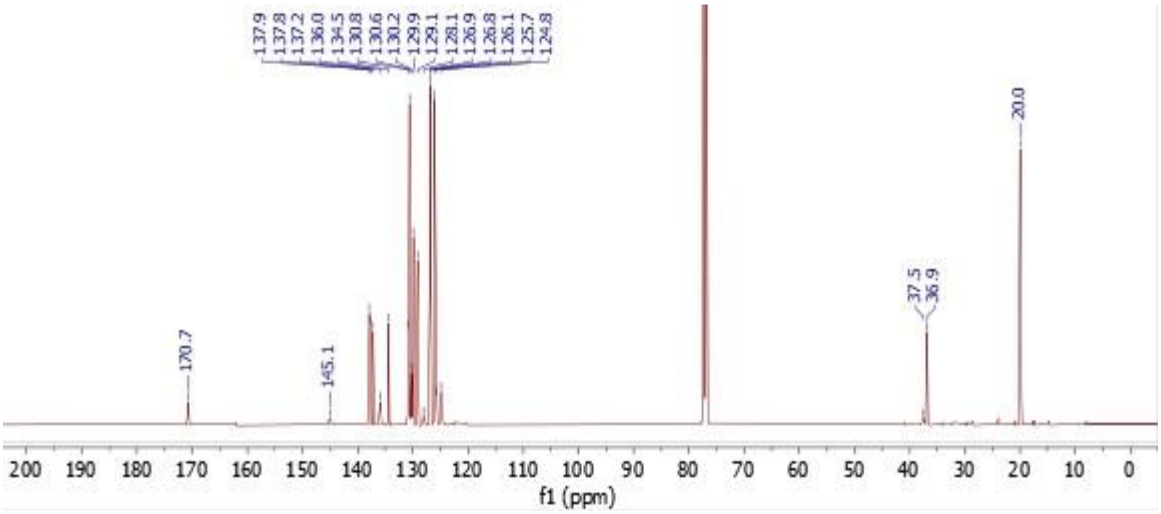

HSQC of **5e**

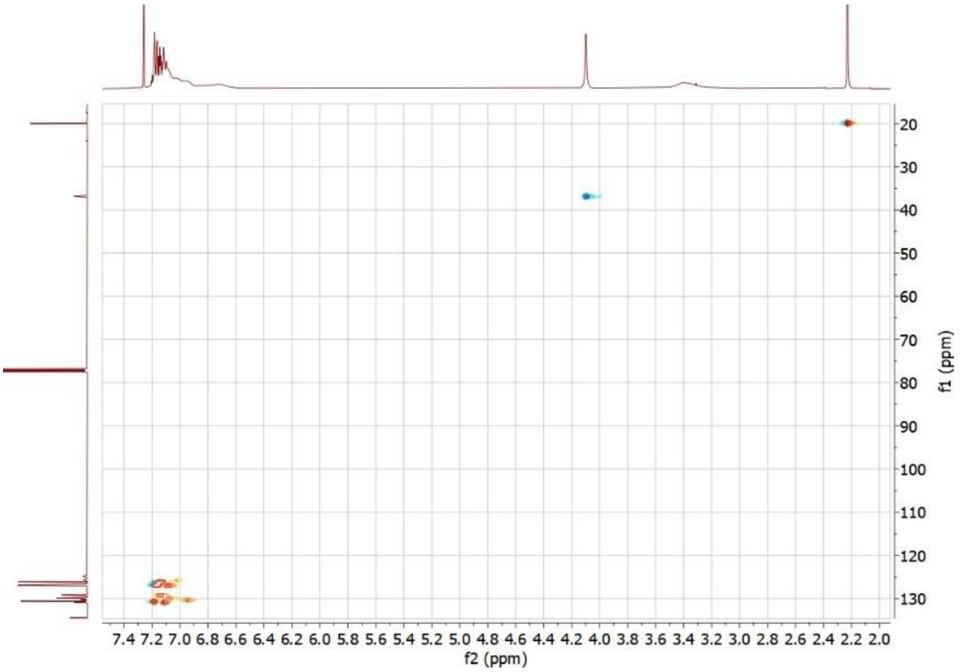

^{1}H NMR of **5f**

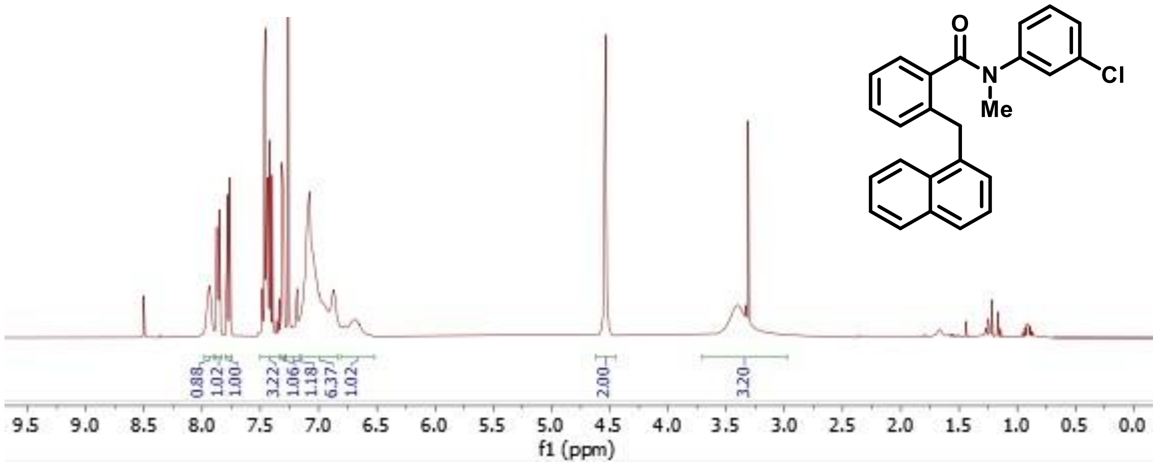

^{13}C NMR of **5f**

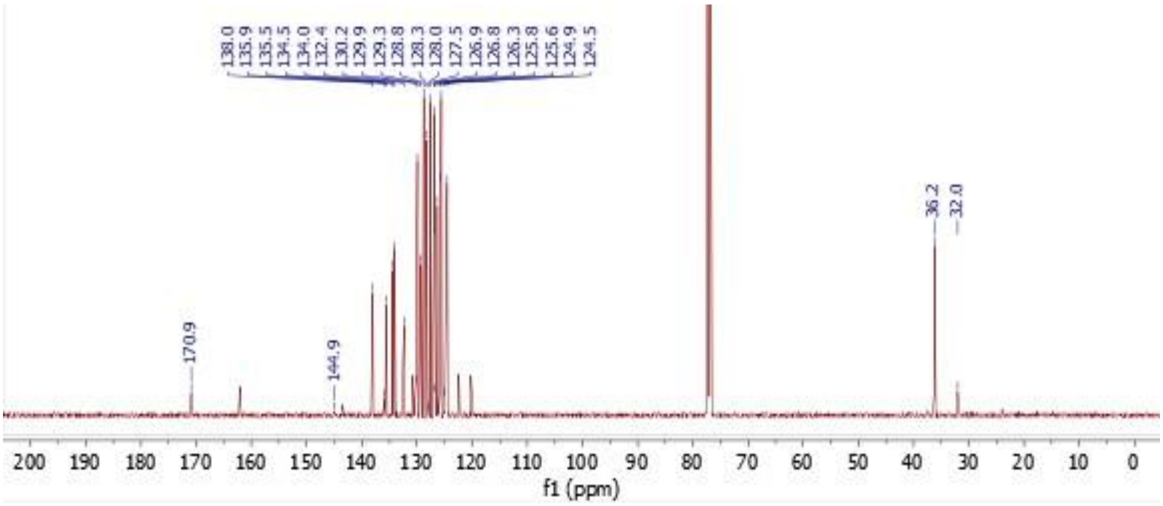

HSQC of **5f**

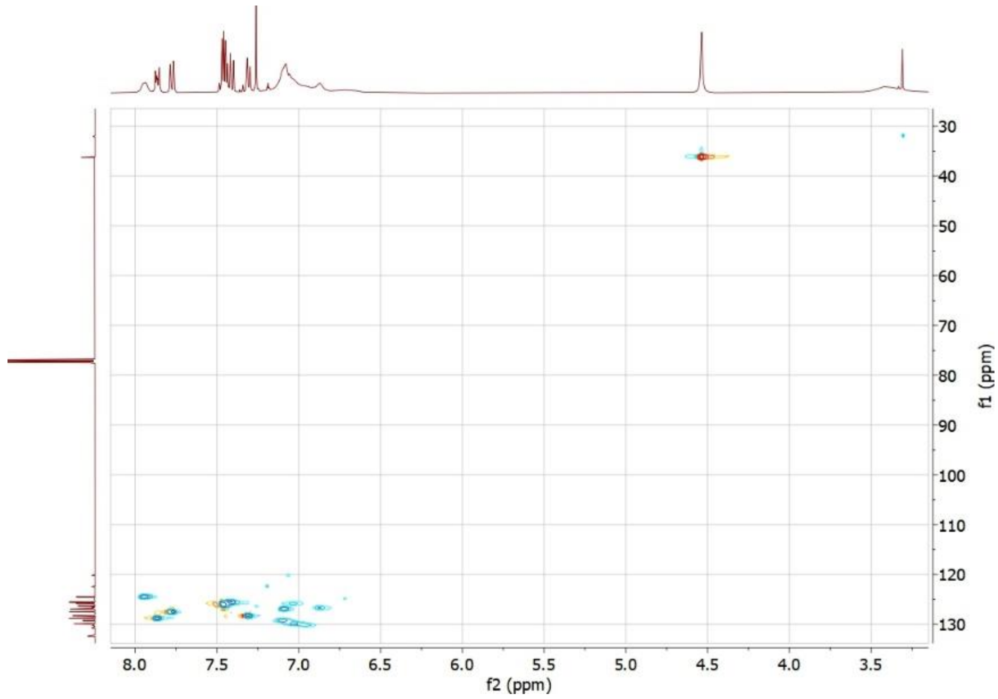

^{1}H NMR of **5g**

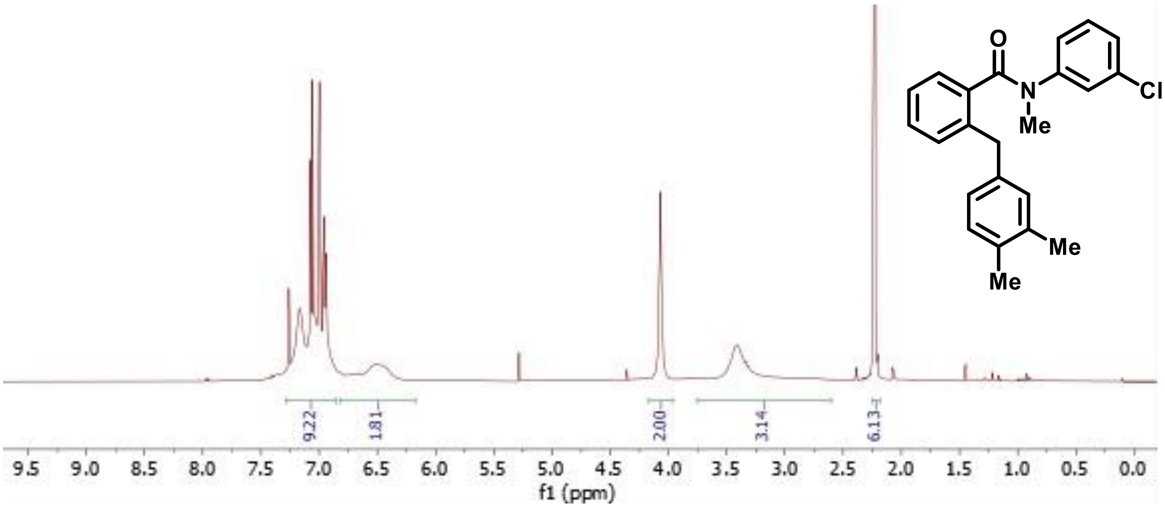

^{13}C NMR of **5g**

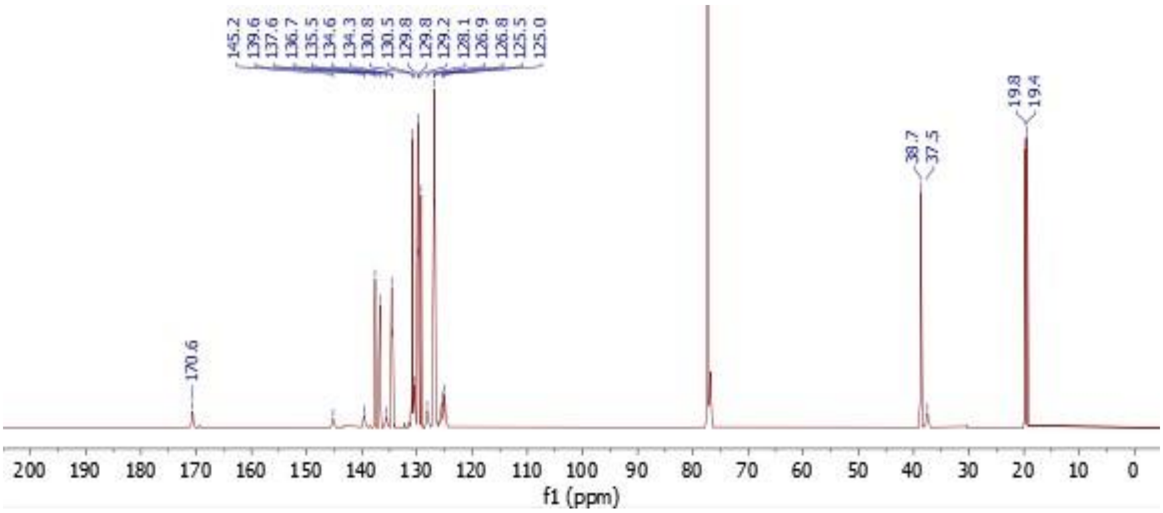

HSQC of **5g**

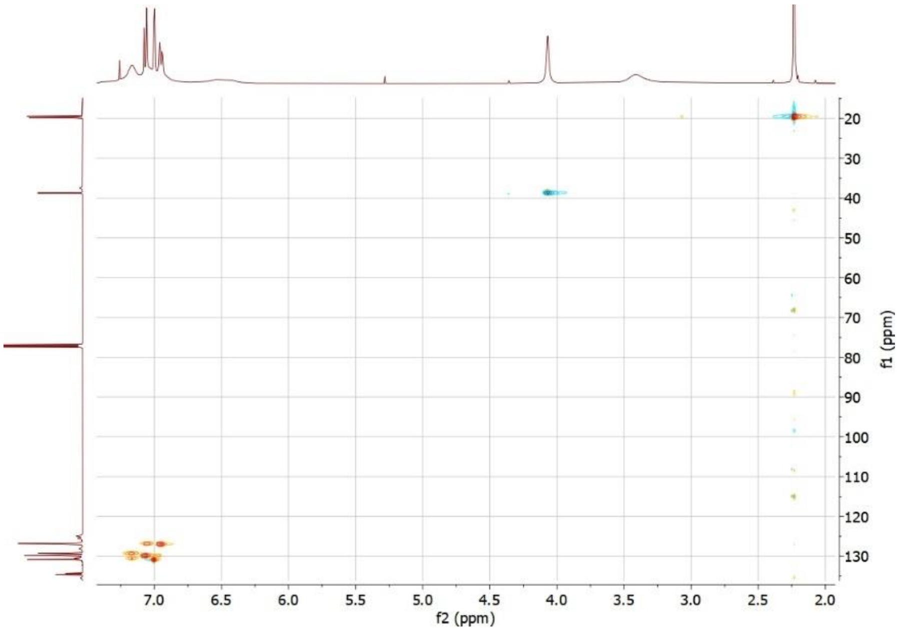

^{1}H NMR of **5h**

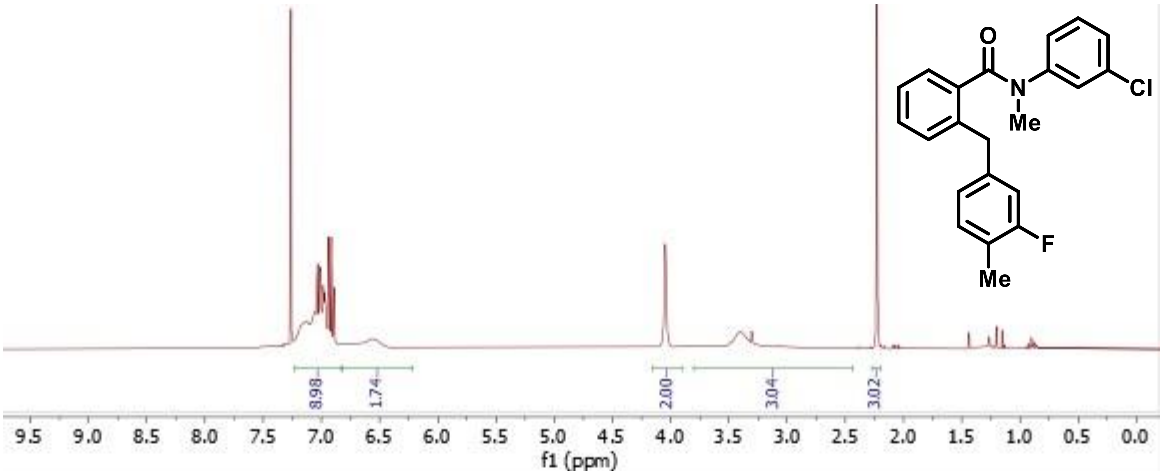

^{13}C NMR of **5h**

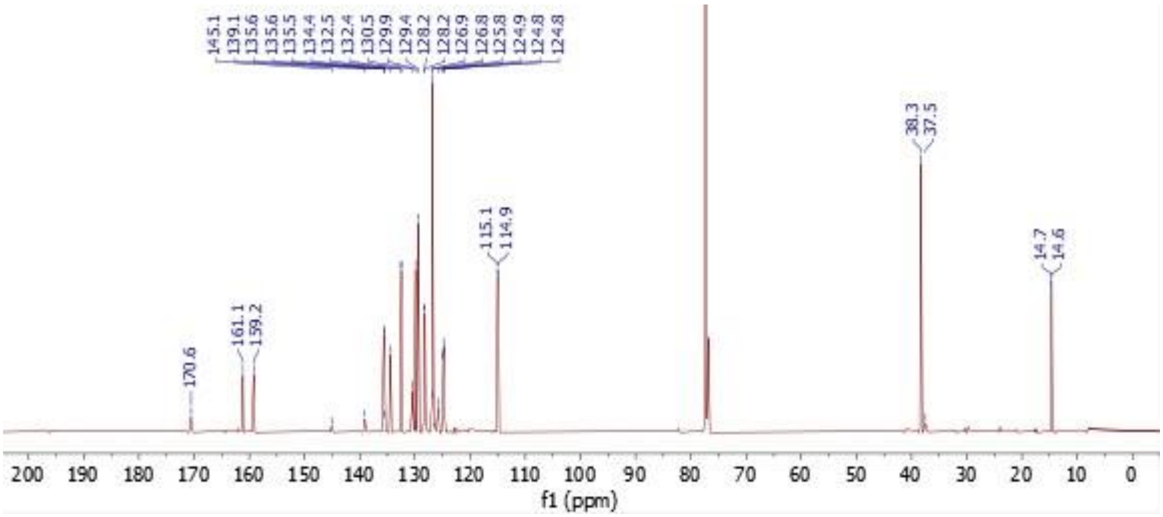

HSQC of **5h**

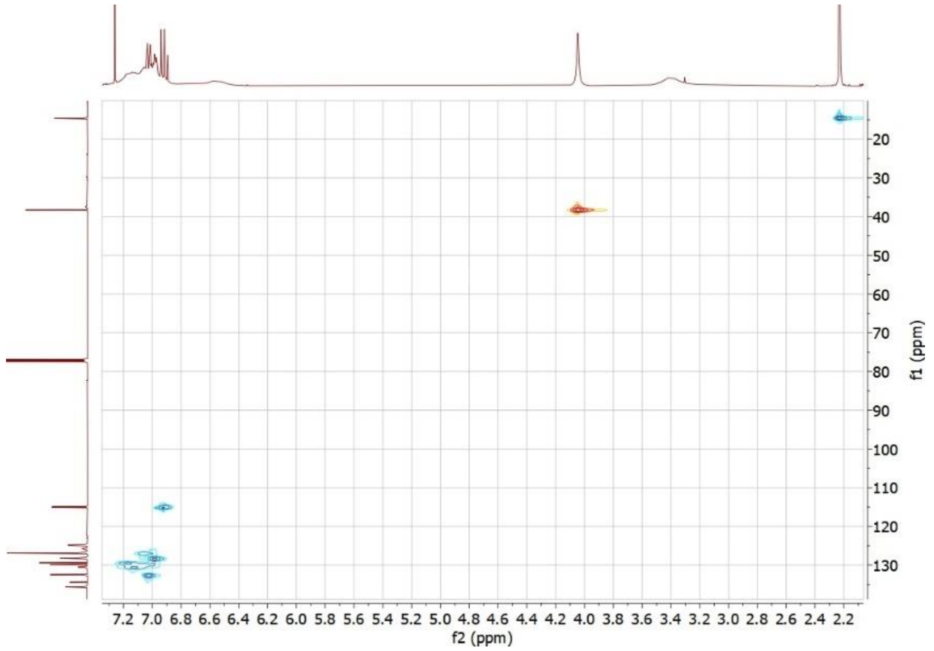

^{1}H NMR of **5i**

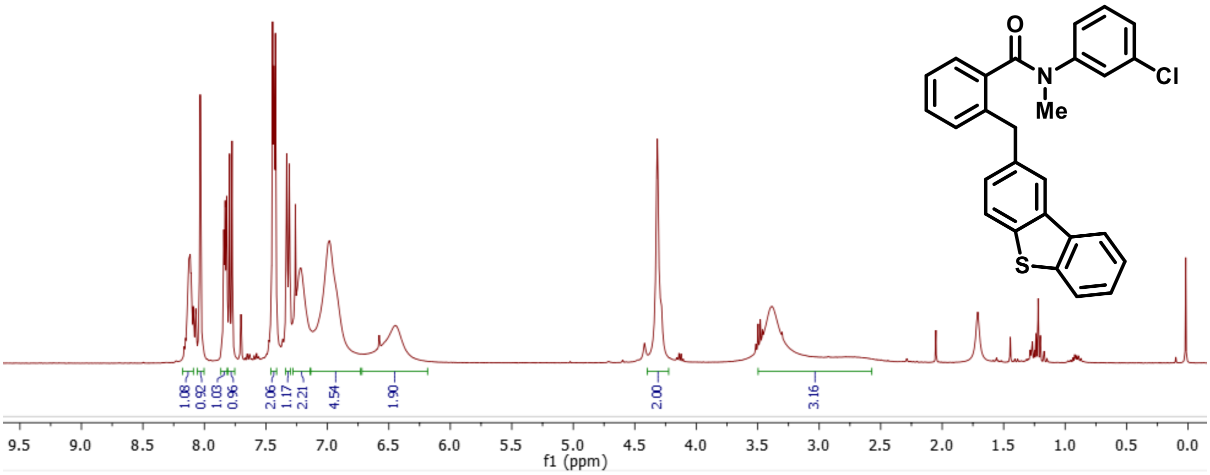

^{13}C NMR of **5i**

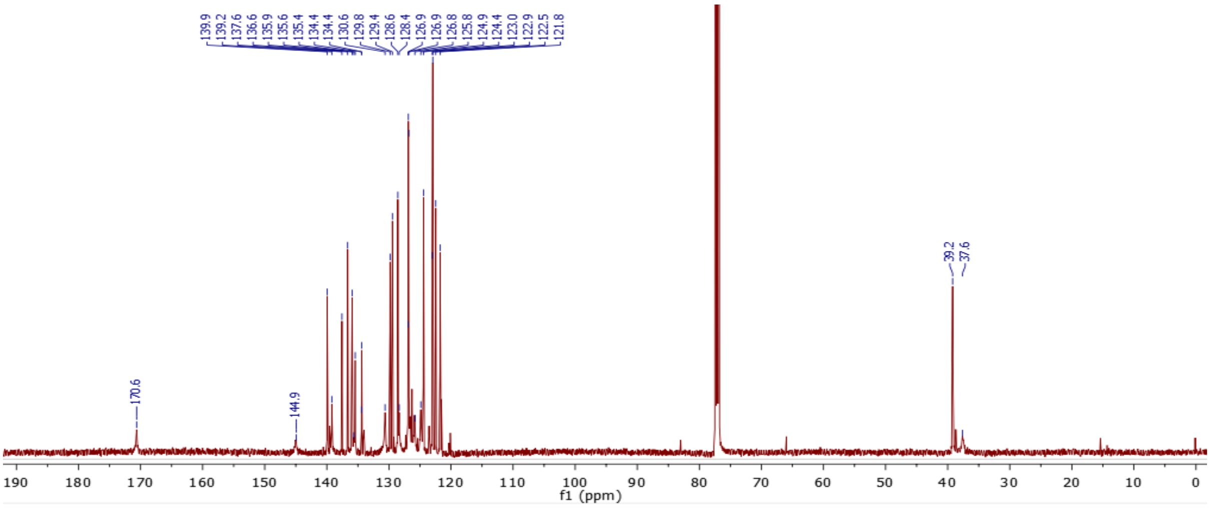

HSQC of **5i**

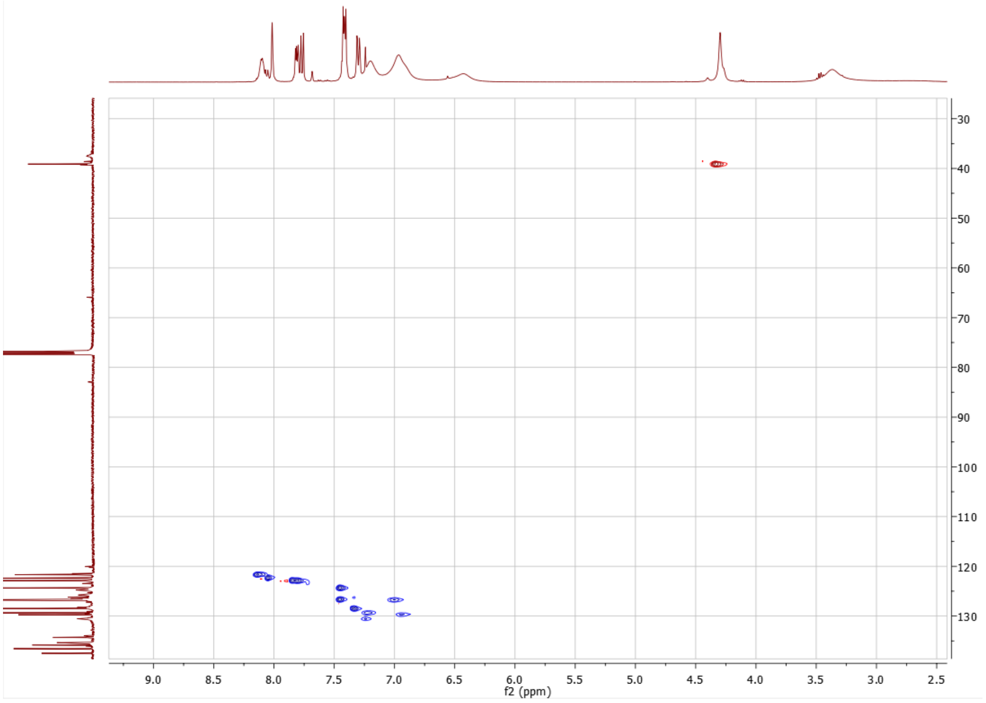

^{1}H NMR of **5j**

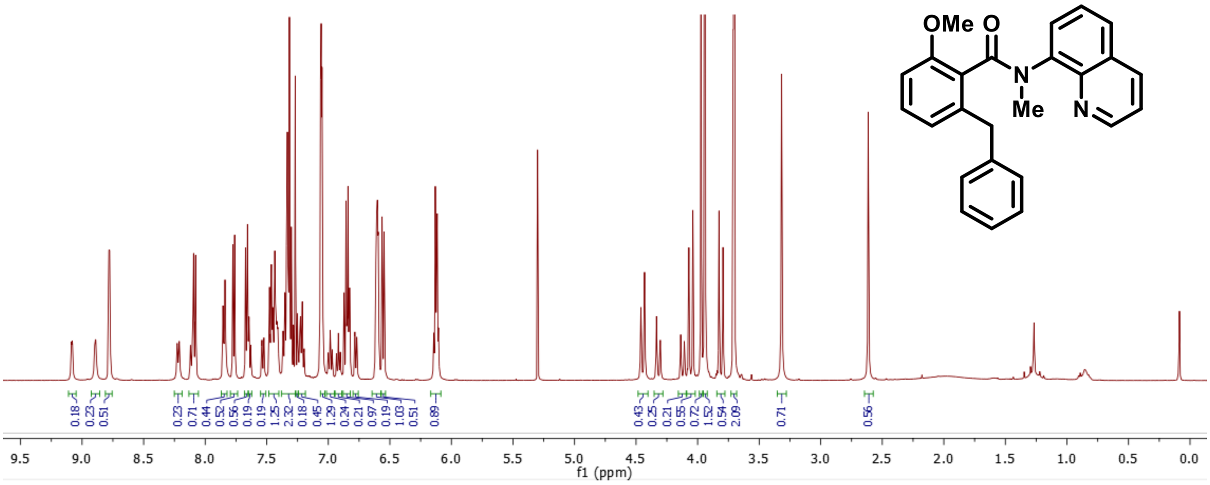

^{13}C NMR of **5j**

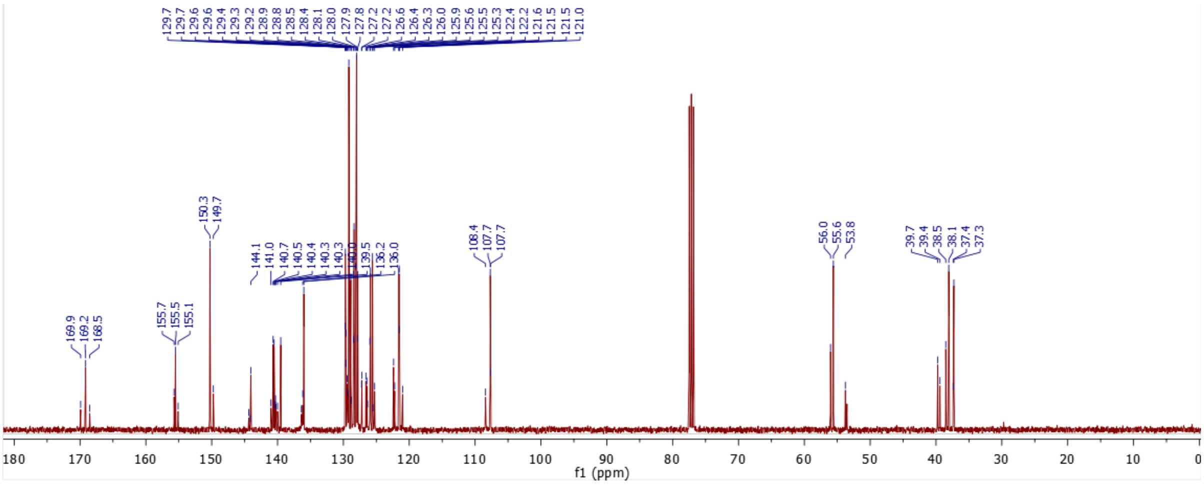

HSQC of **5j**

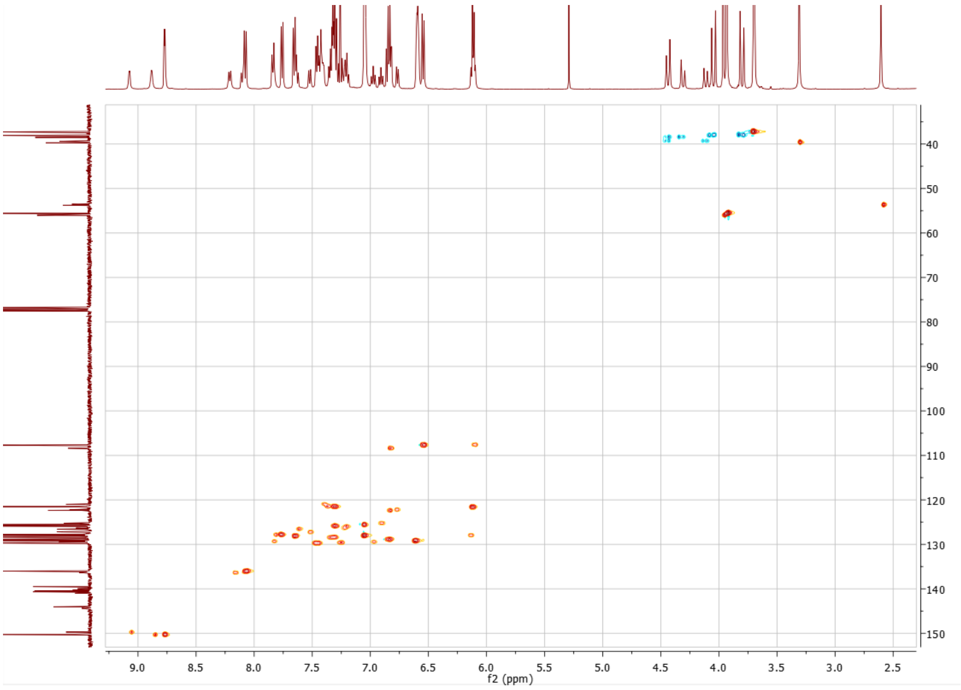

^{1}H NMR of **6a**

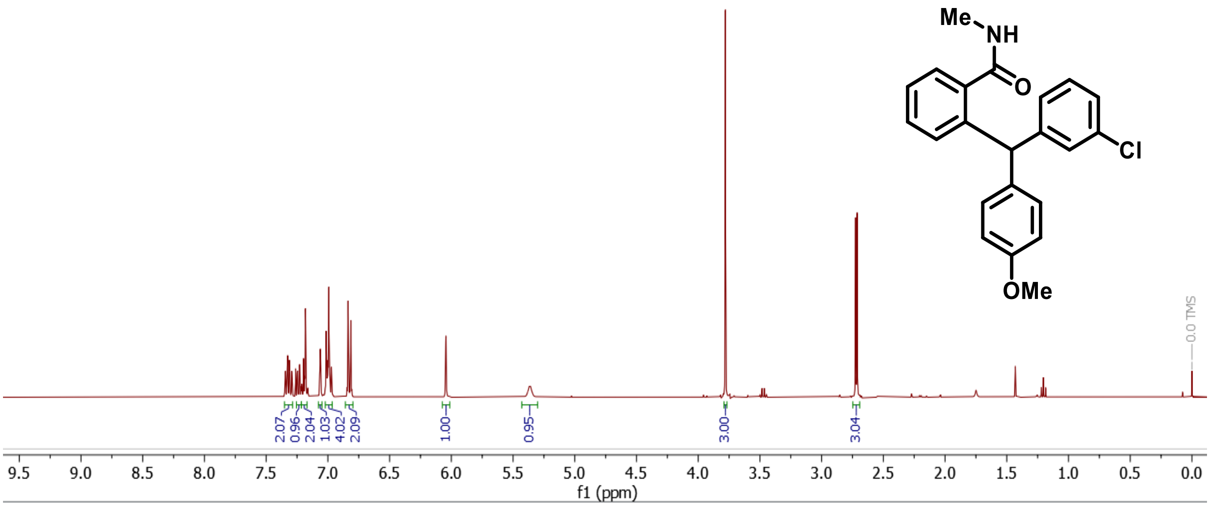

^{13}C NMR of **6a**

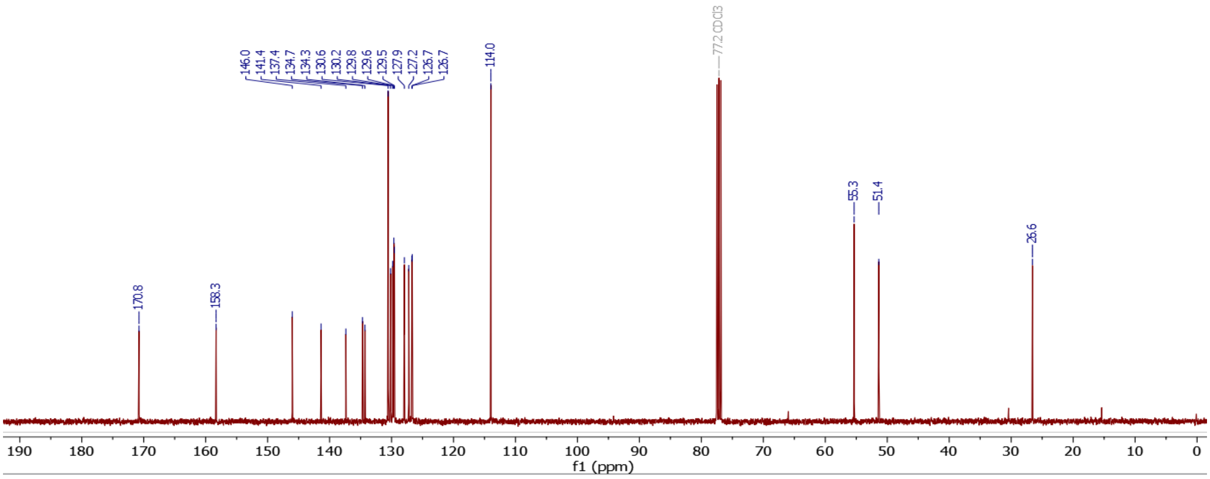

HSQC of **6a**

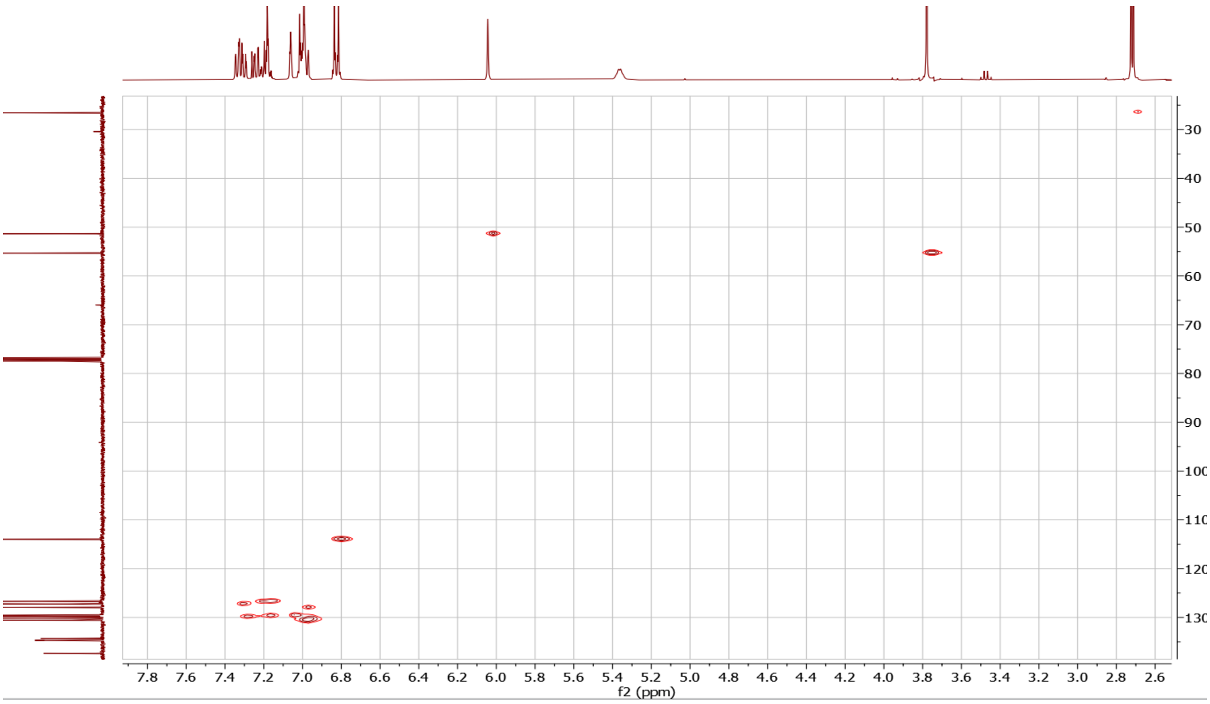

^{1}H NMR of **6b**

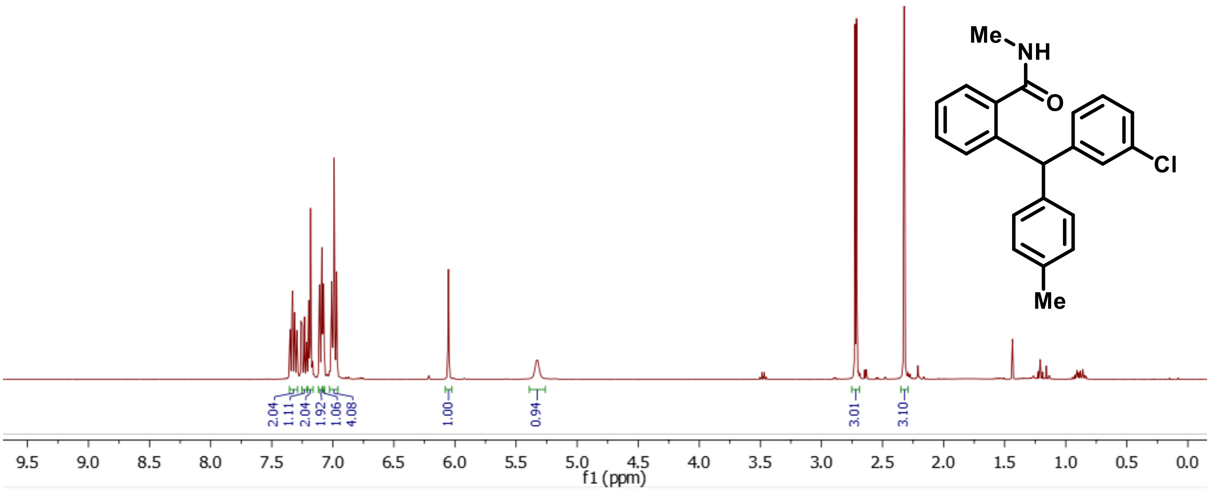

^{13}C NMR of **6b**

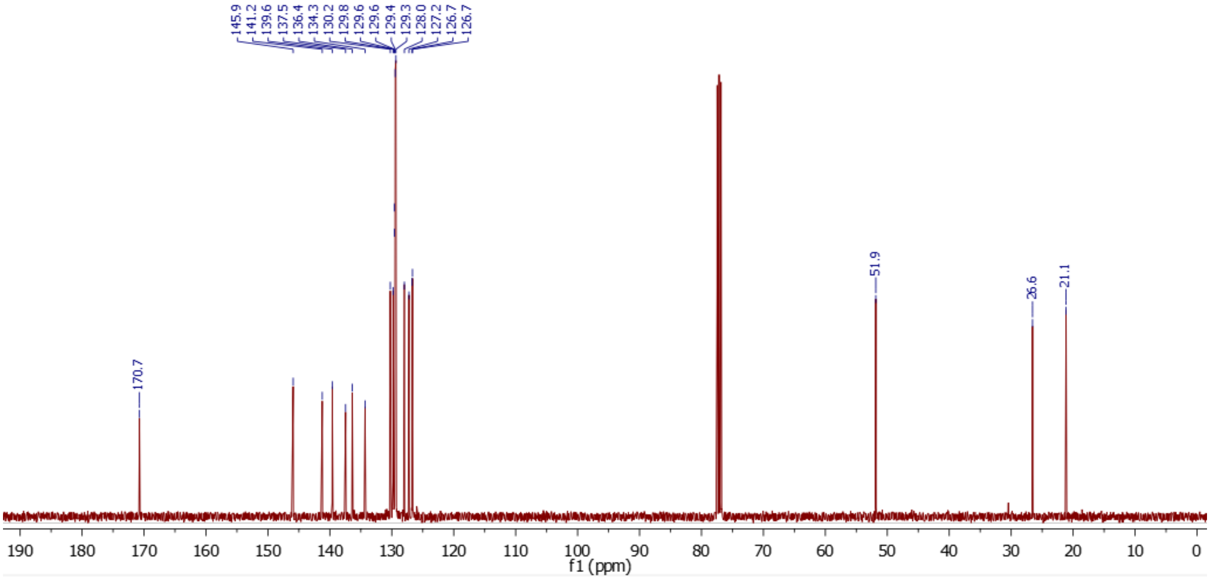

HSQC of **6b**

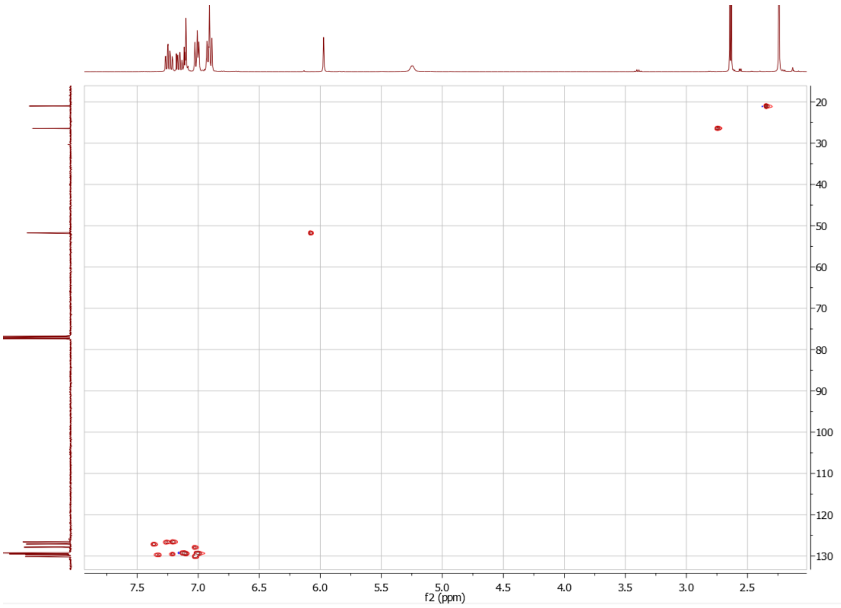

^{1}H NMR of **6c**

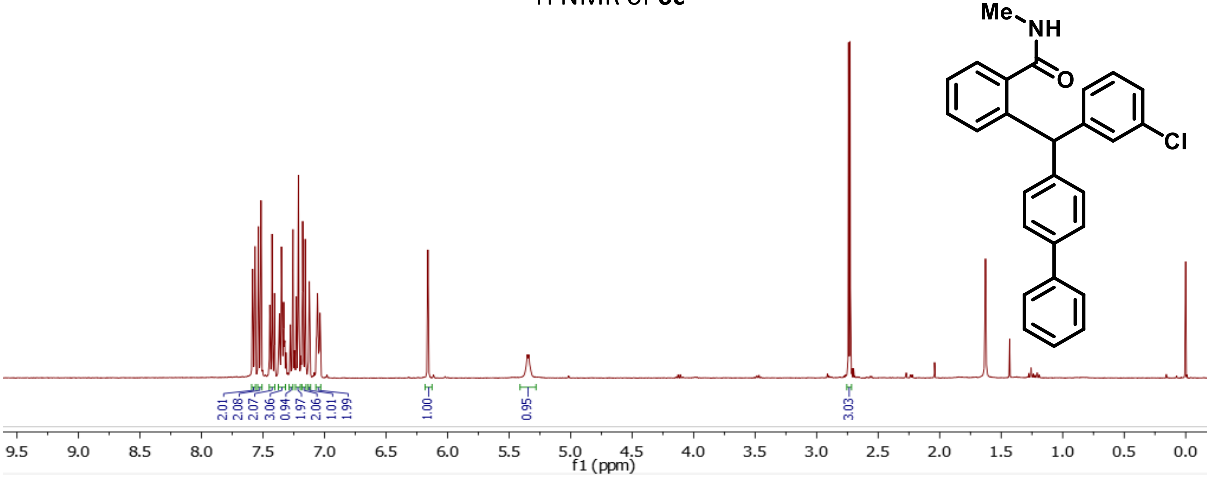

^{13}C NMR of **6c**

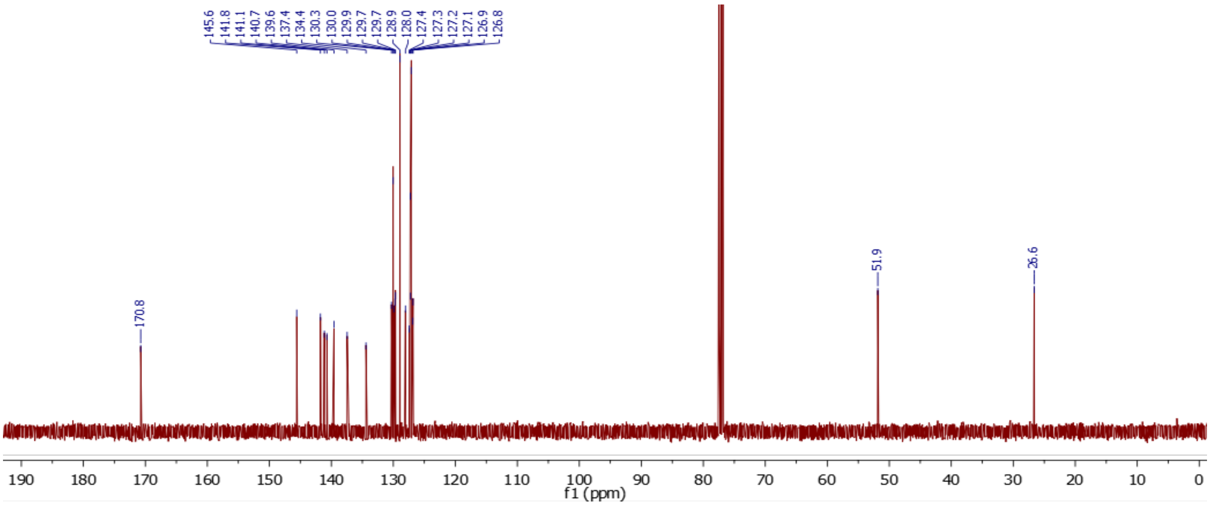

HSQC of **6c**

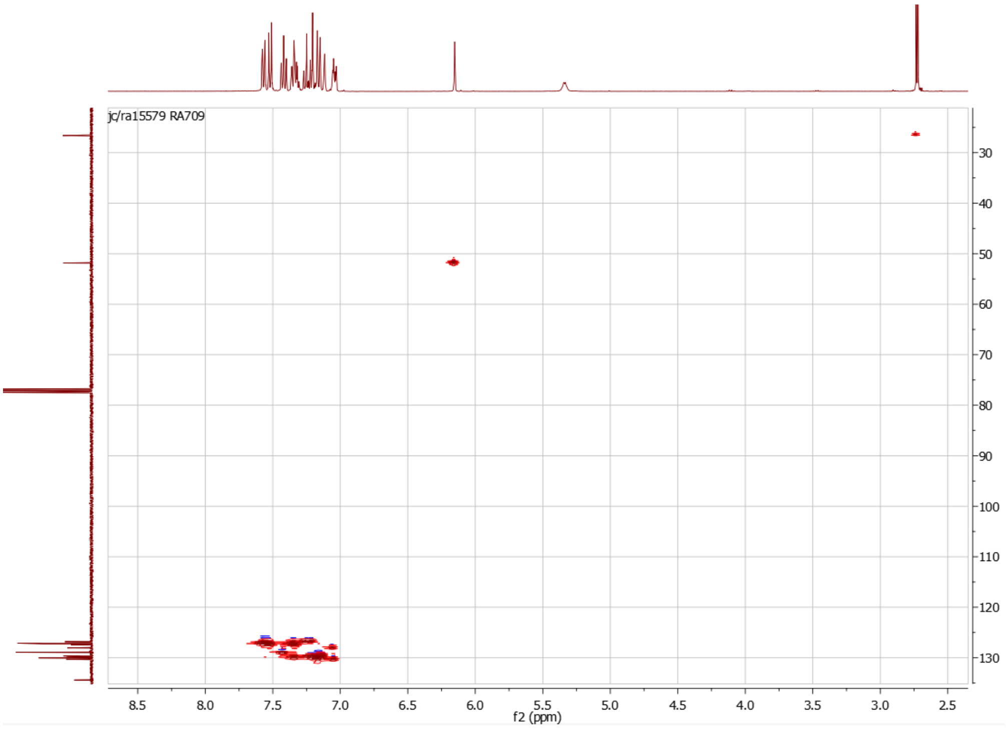

^{1}H NMR of **6d**

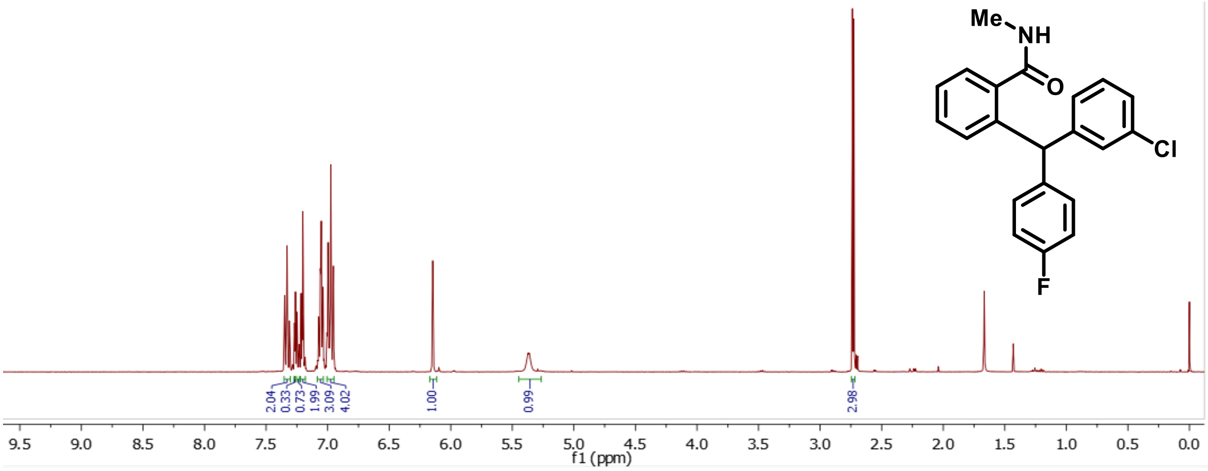

^{13}C NMR of **6d**

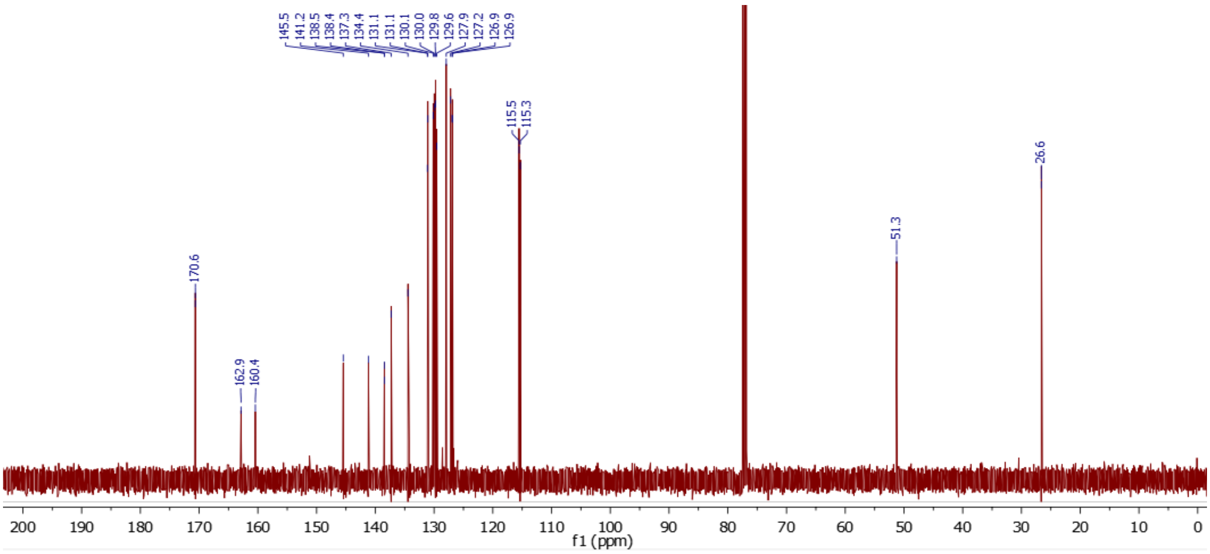

HSQC of **6d**

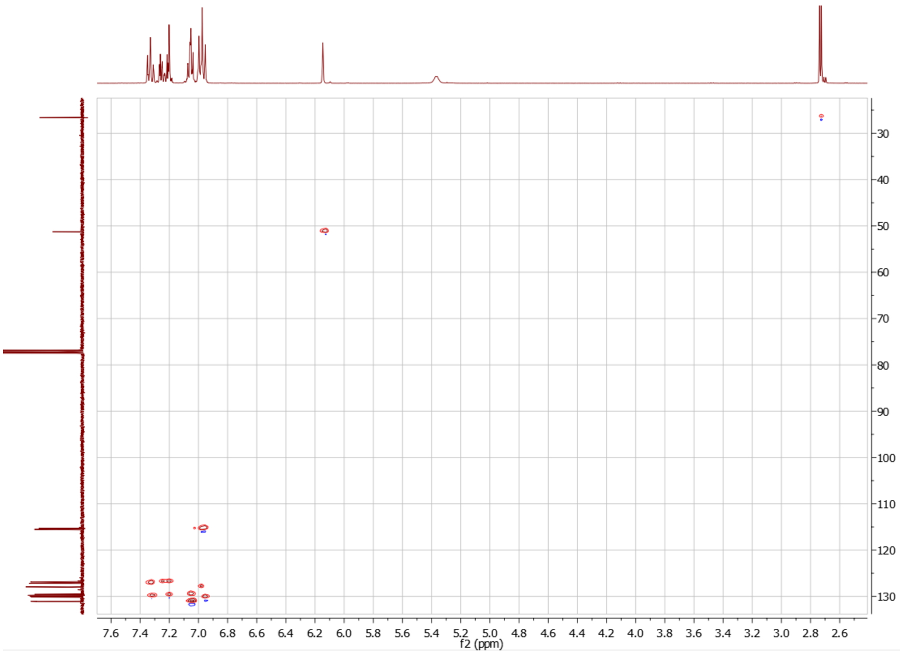

^{1}H NMR of **6e**

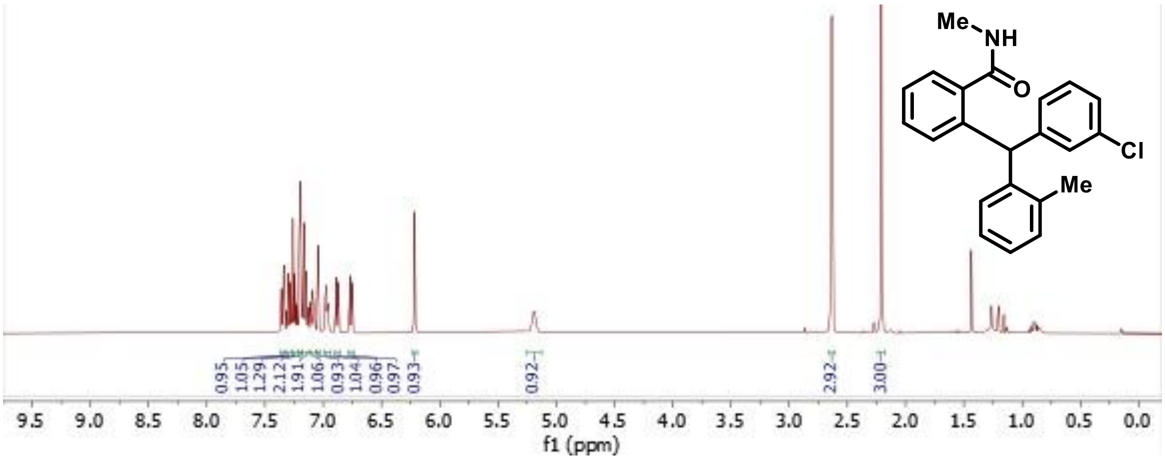

^{13}C NMR of **6e**

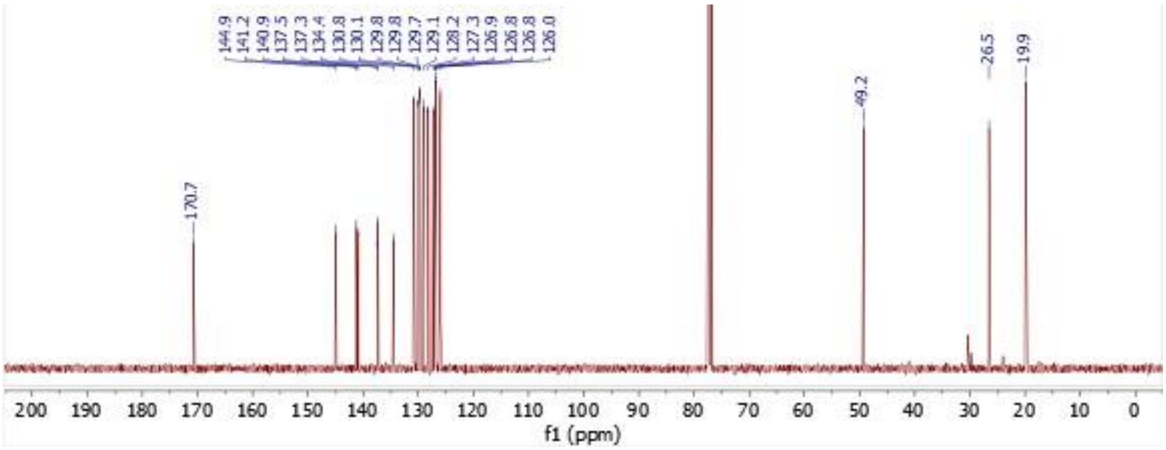

HSQC of **6e**

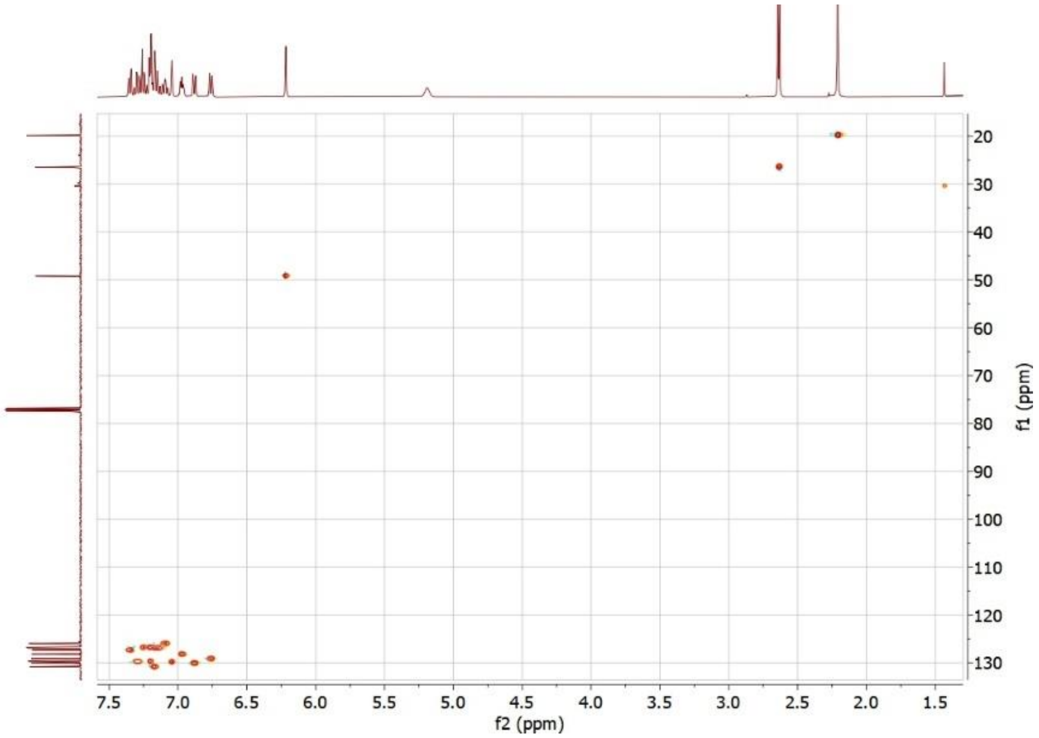

^{1}H NMR of **6f**

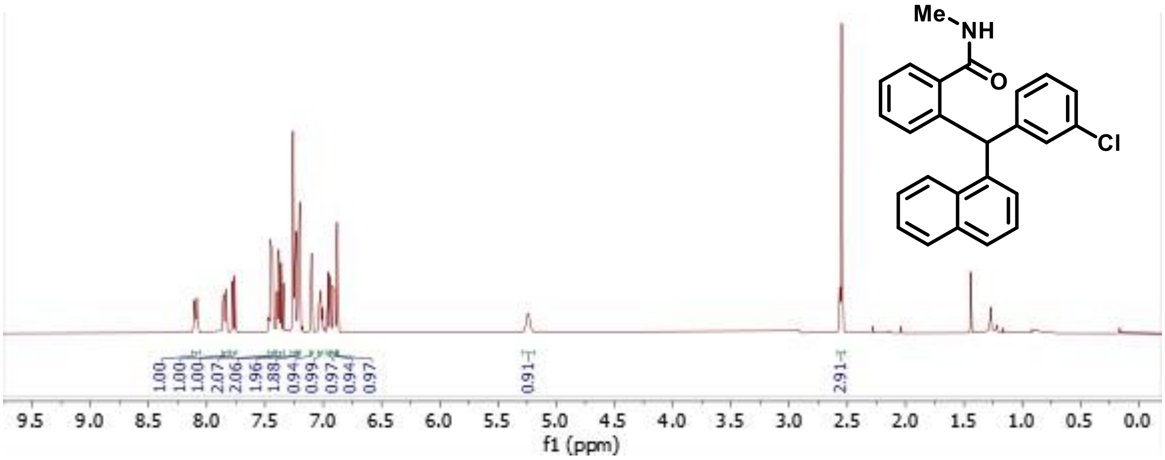

^{13}C NMR of **6f**

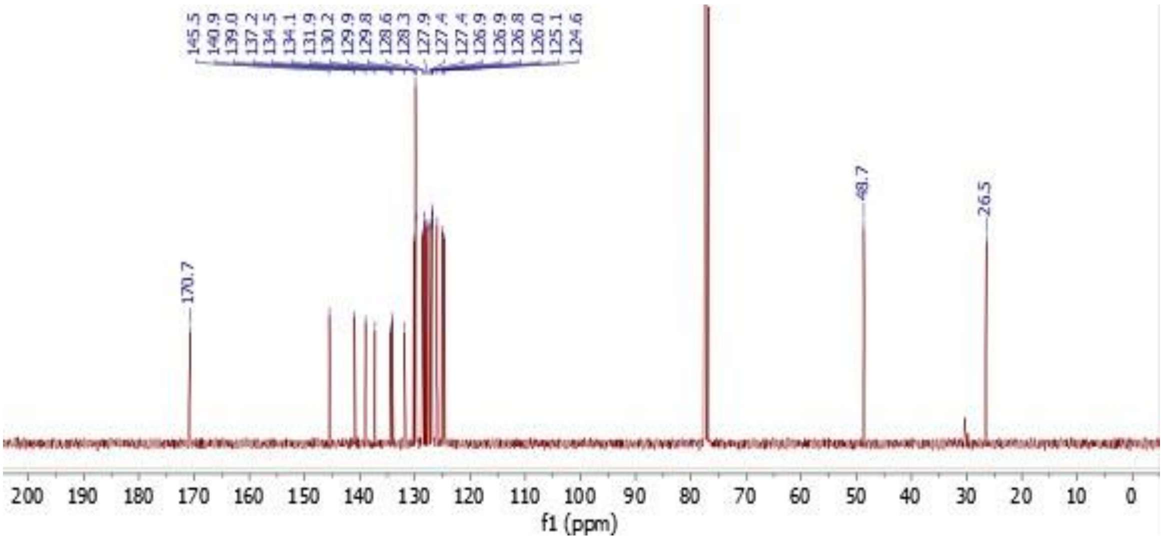

HSQC of **6f**

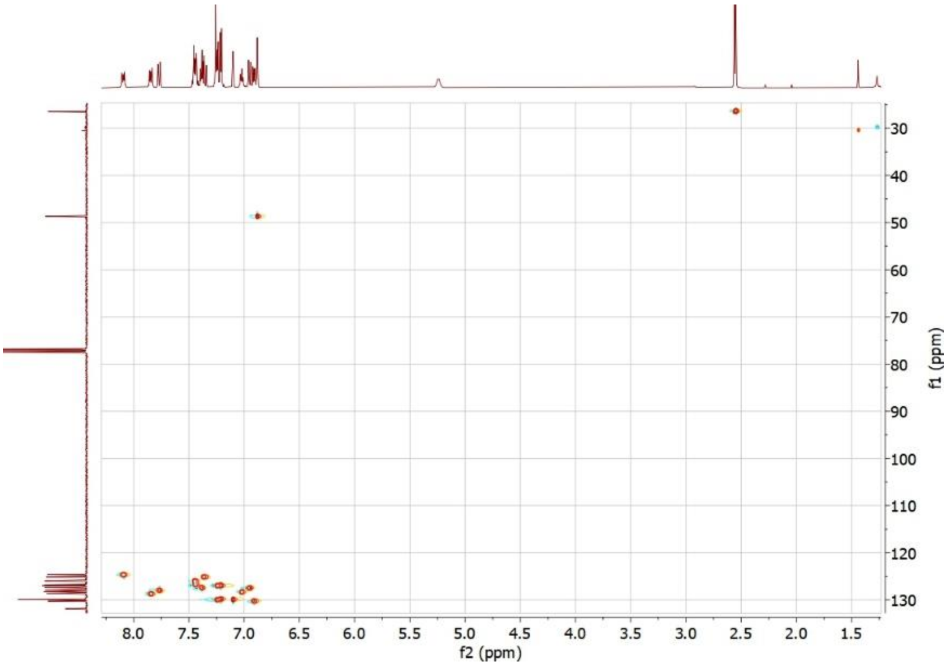

^{1}H NMR of **6g**

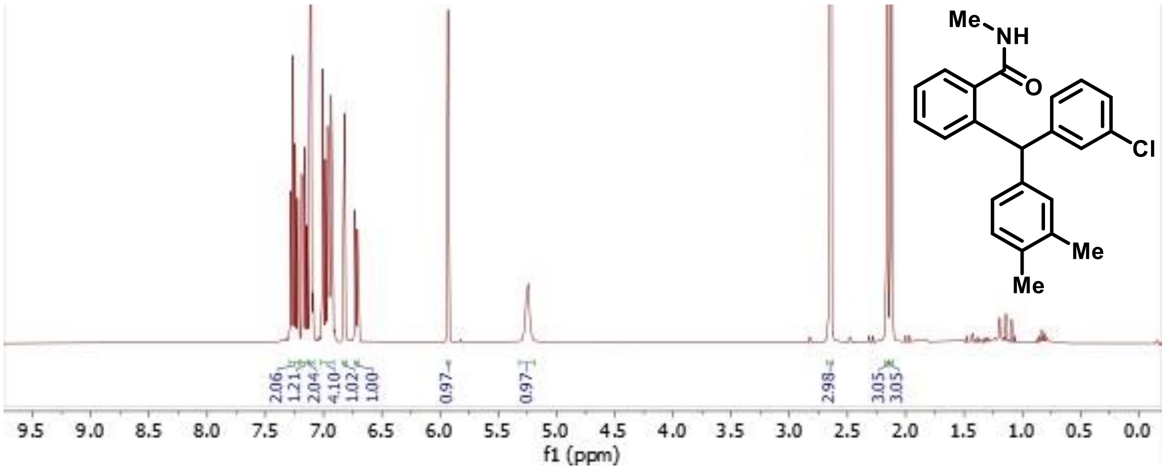

^{13}C NMR of **6g**

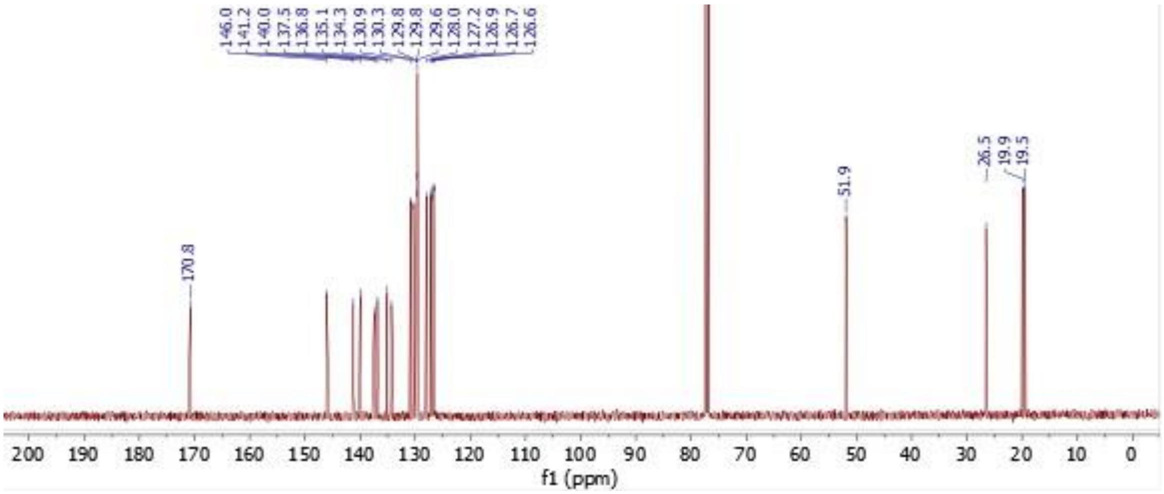

HSQC of **6g**

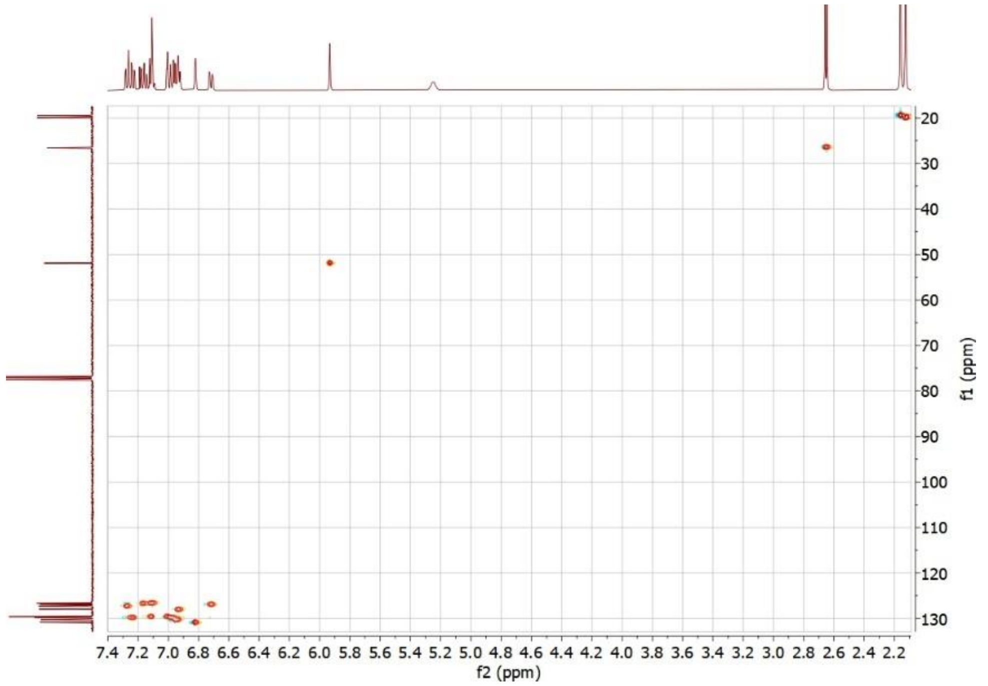

^{1}H NMR of **6h**

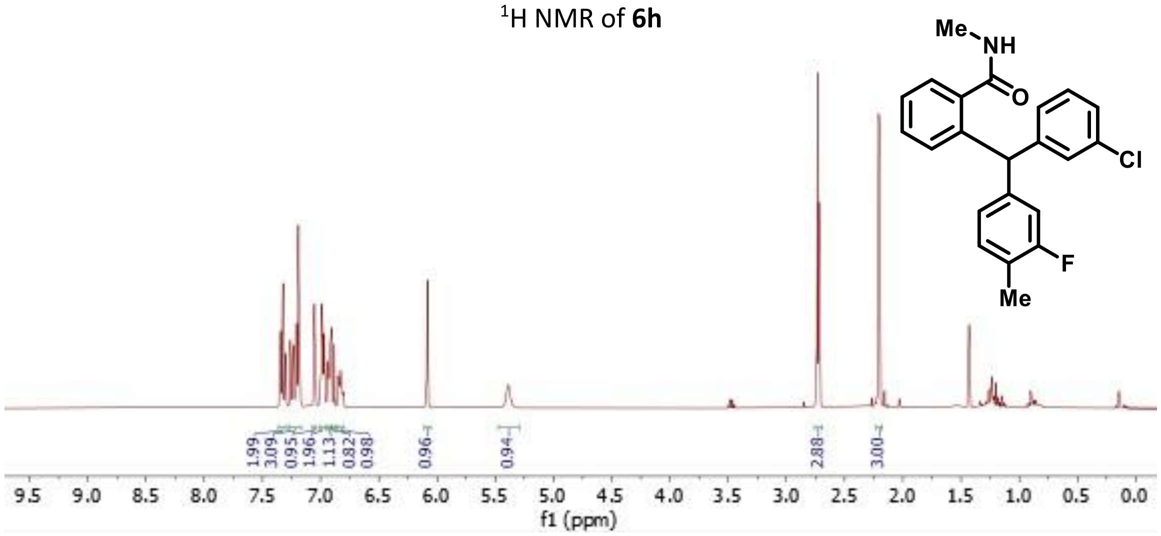

^{13}C NMR of **6h**

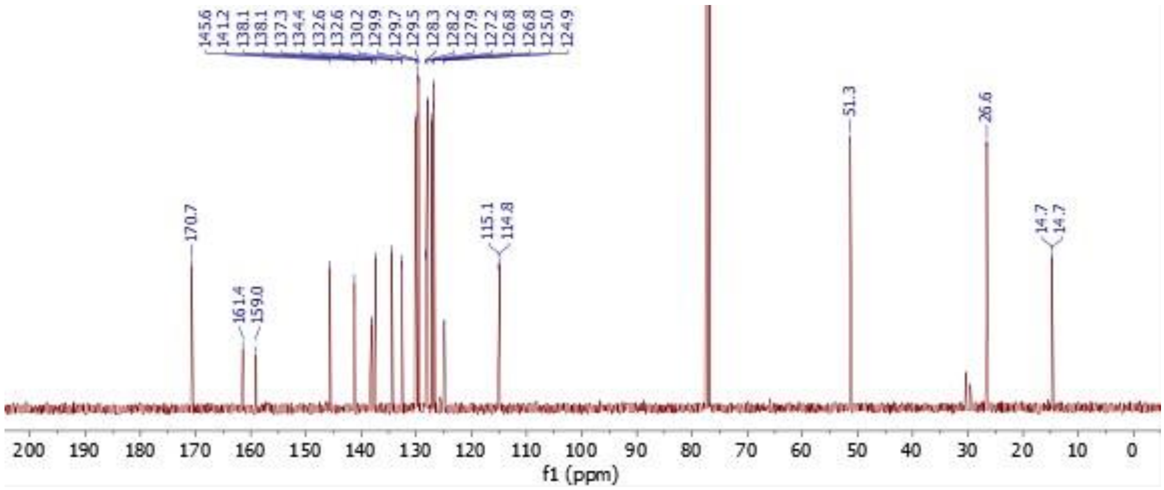

HSQC of **6h**

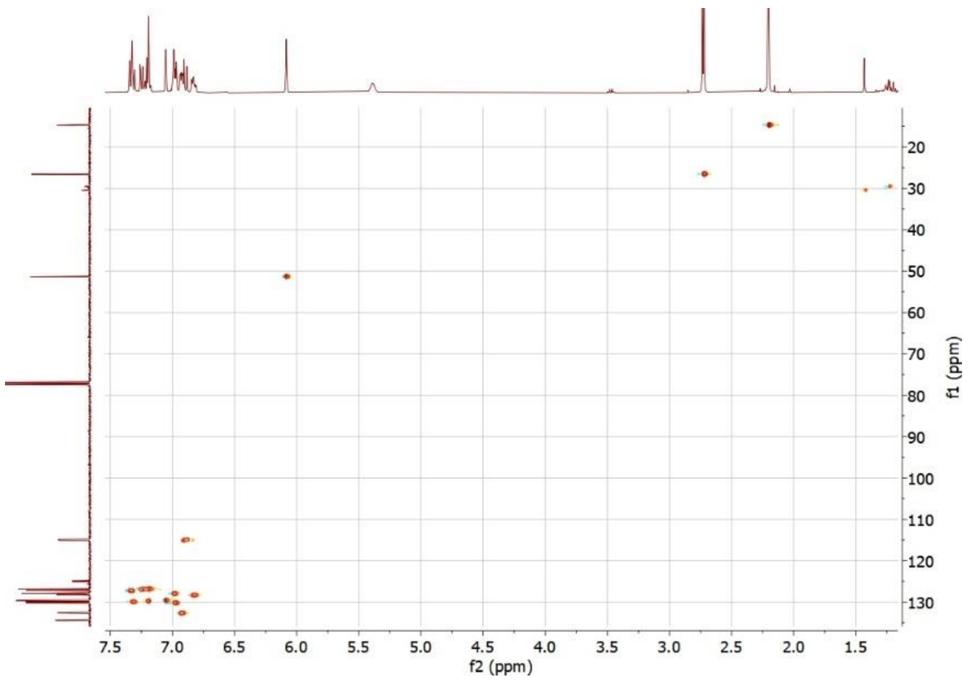

^{1}H NMR of **6i**

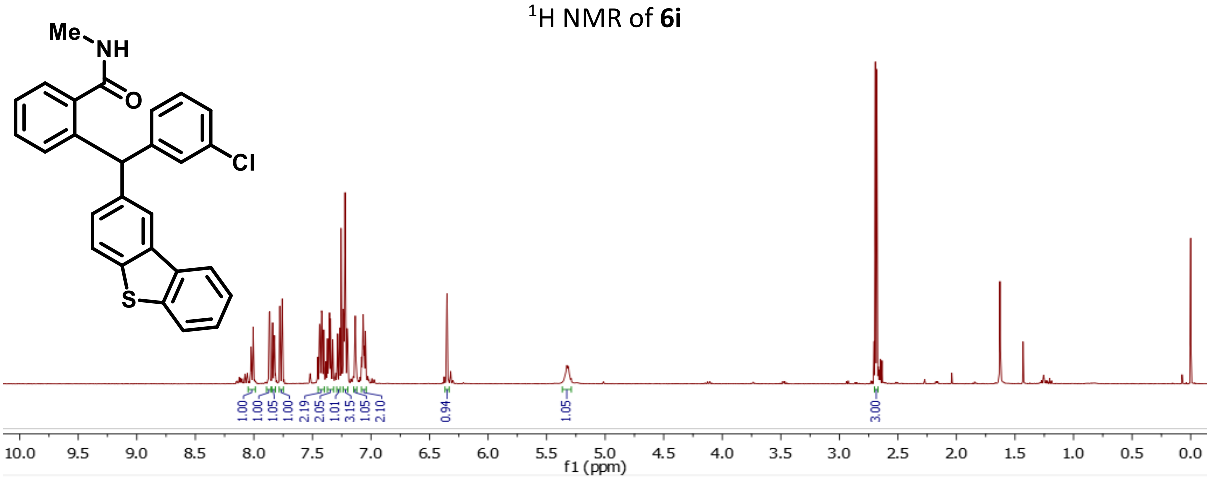

^{13}C NMR of **6i**

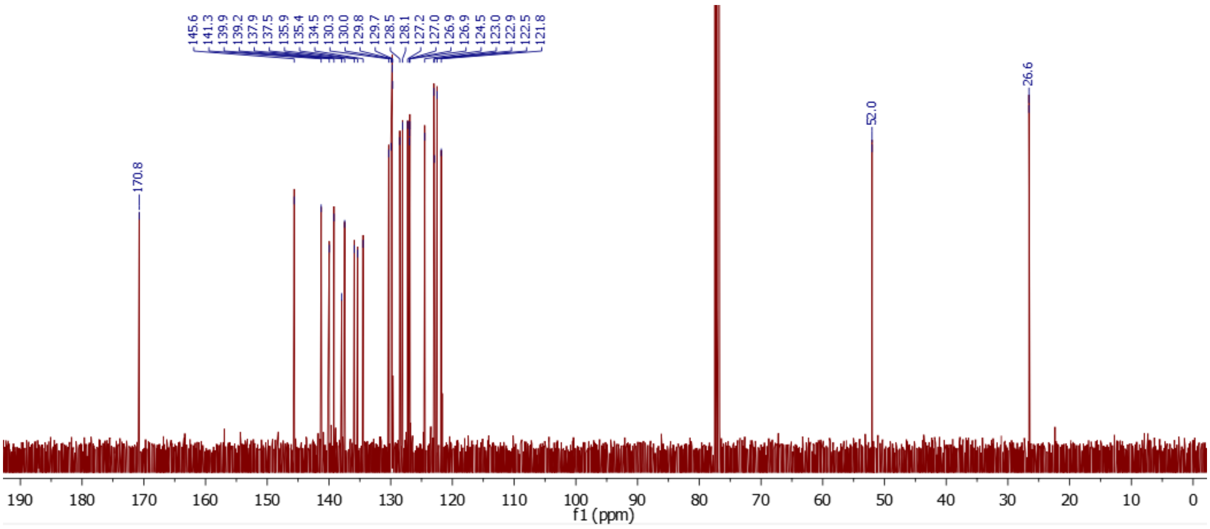

HSQC of **6i**

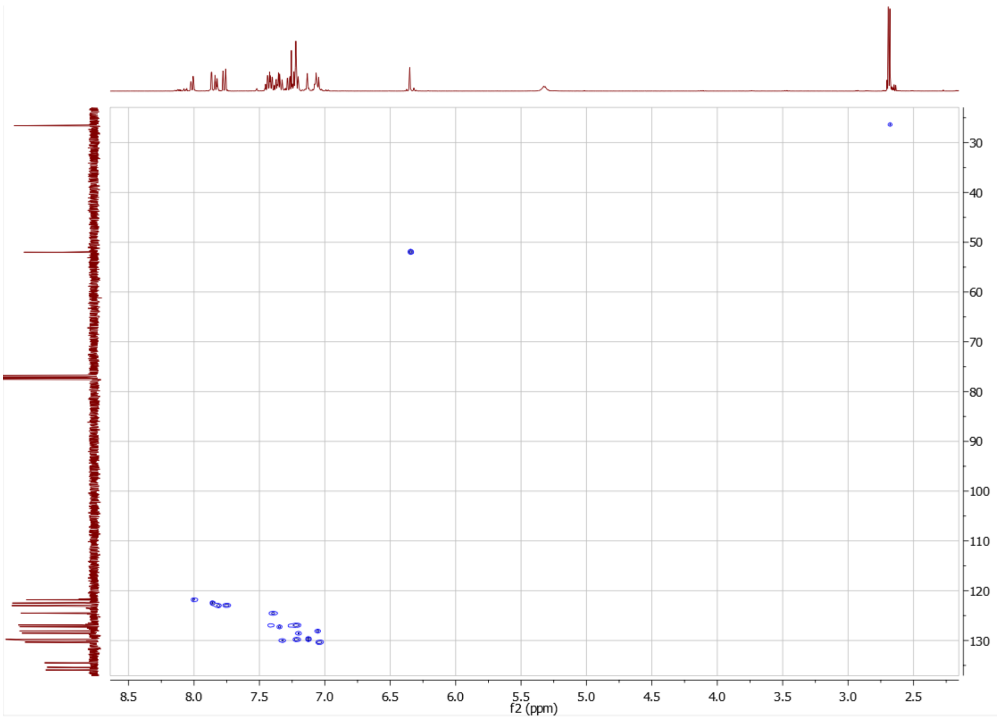

^{1}H NMR of **6j**

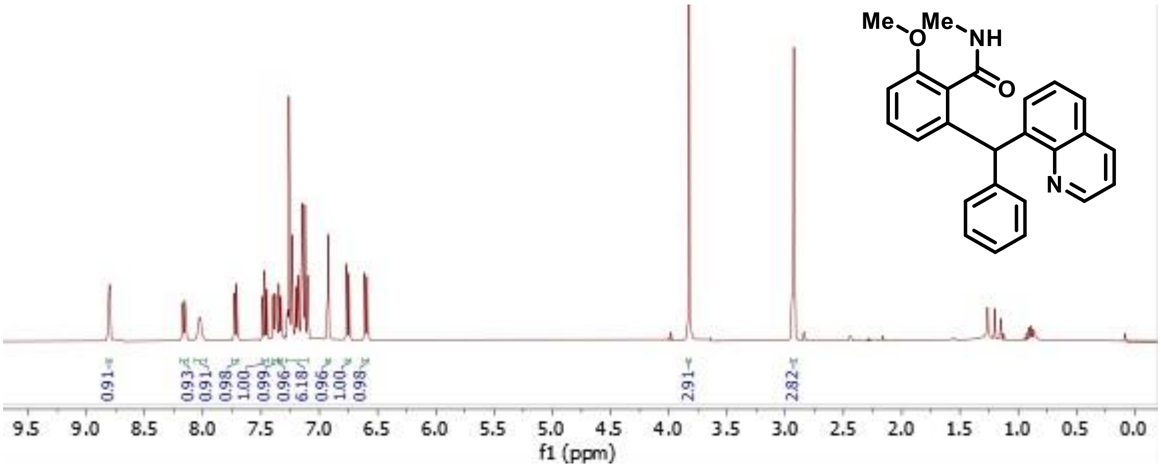

^{13}C NMR of **6j**

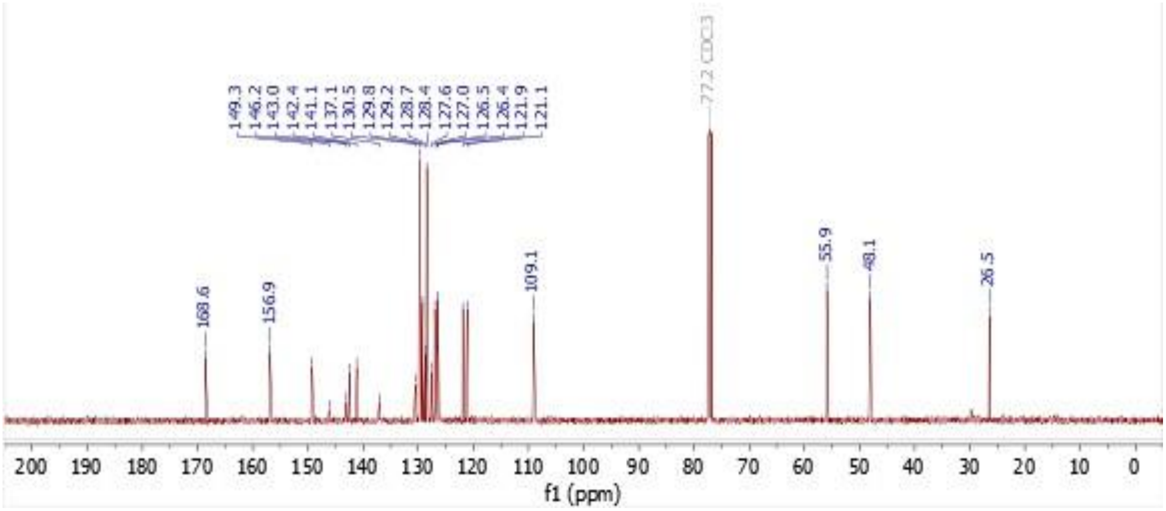

HSQC of **6j**

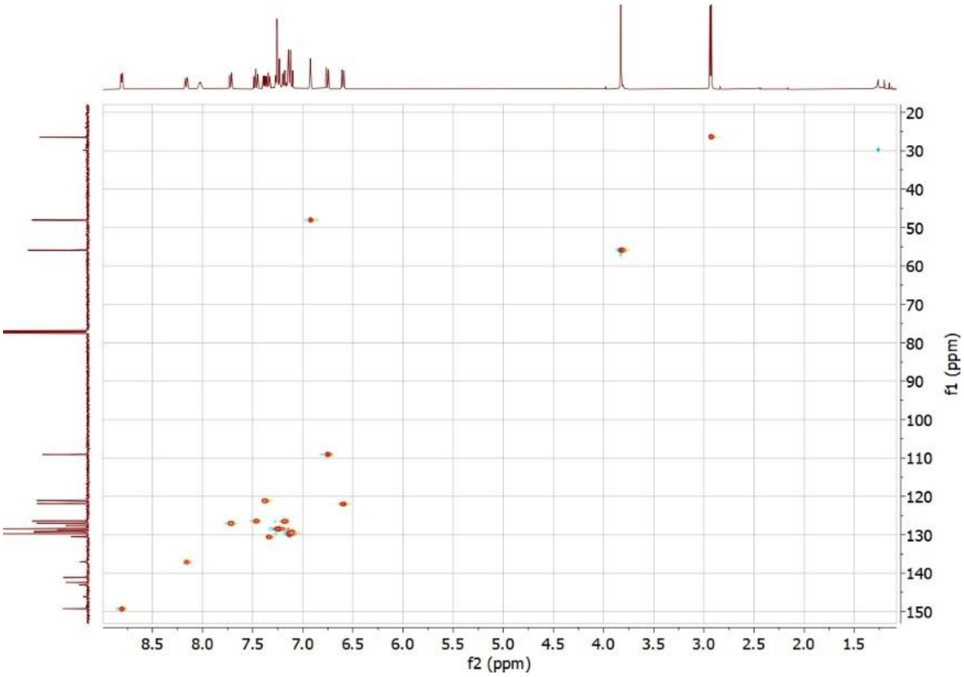

^{1}H NMR of **7a**

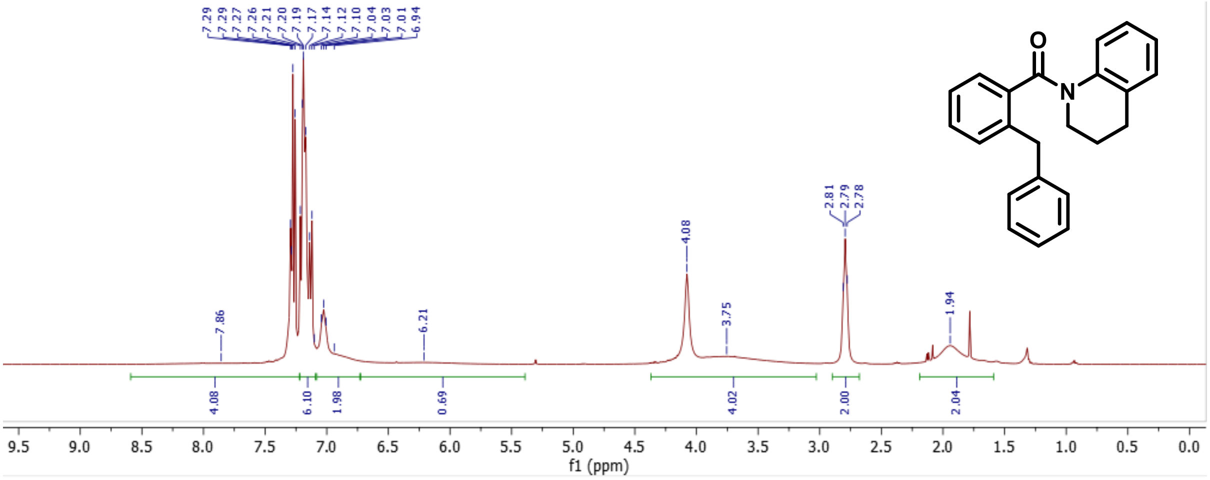

^{13}C NMR of **7a** at 100 °C

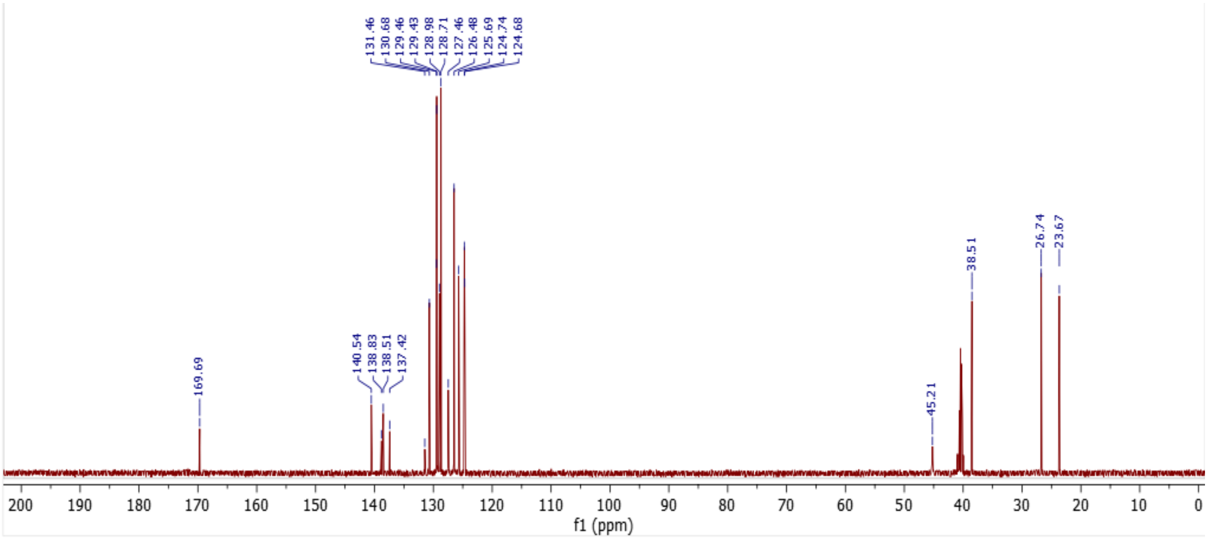

HSQC of **7a**

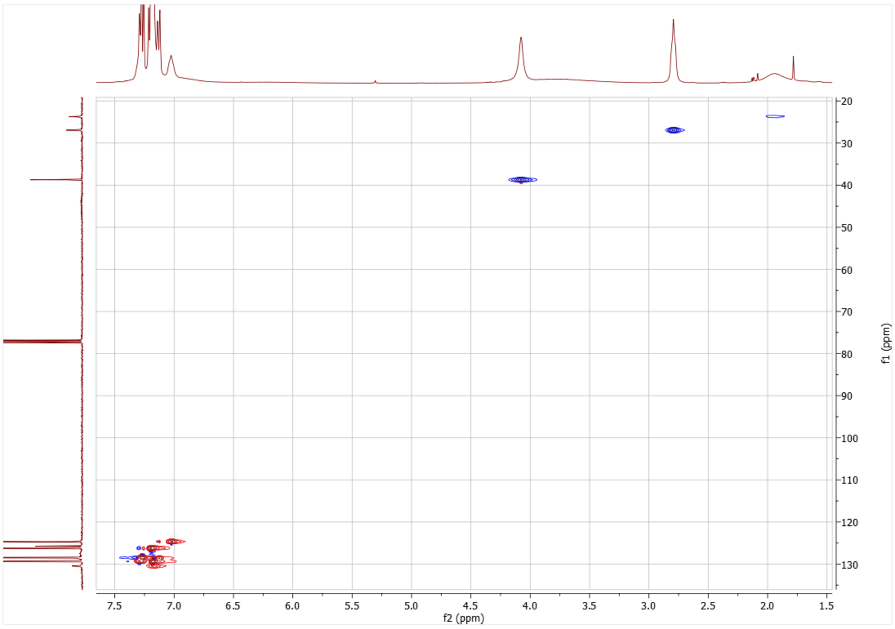

^{1}H NMR of **7b**

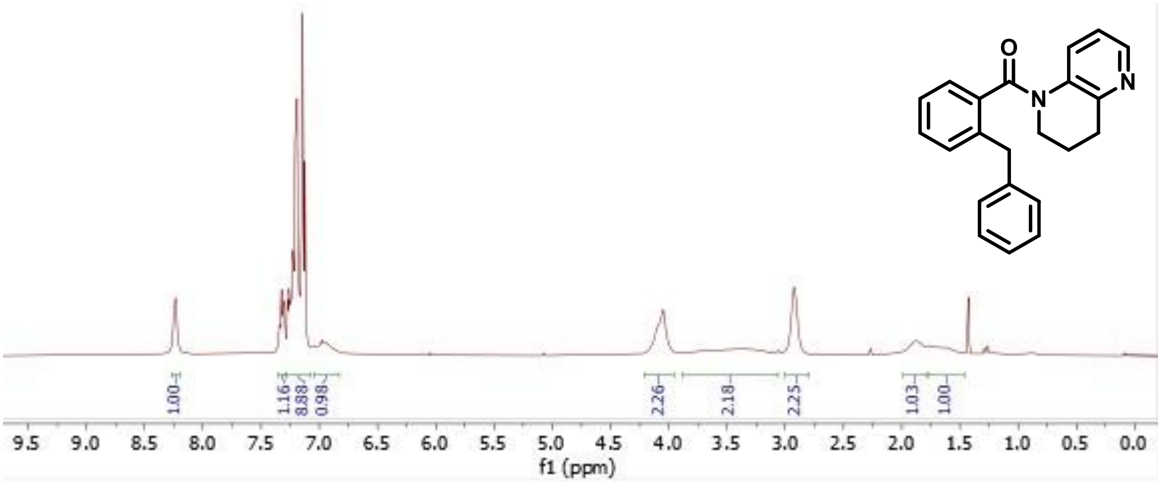

^{13}C NMR of **7b**

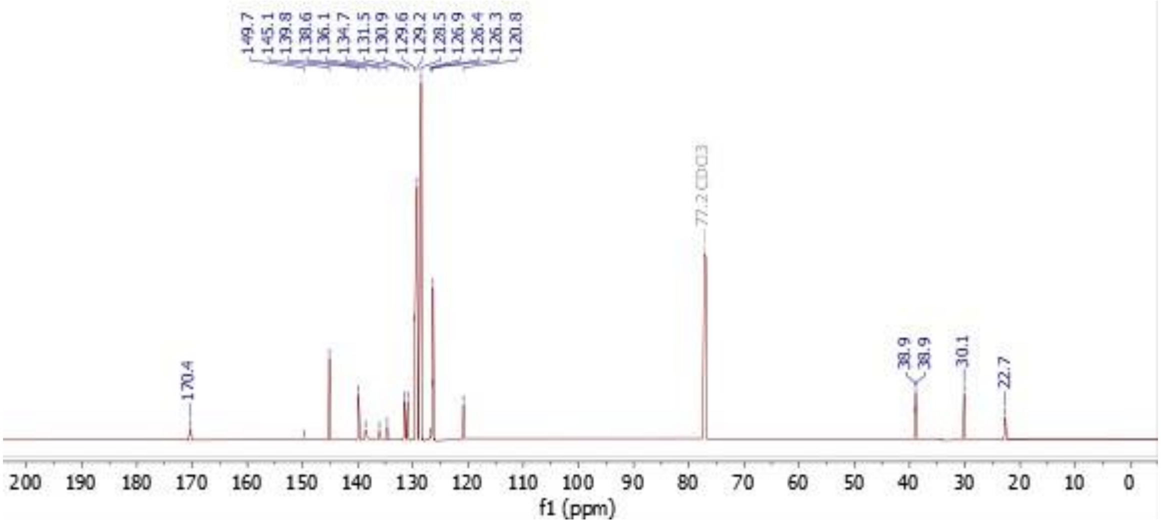

HSQC of **7b**

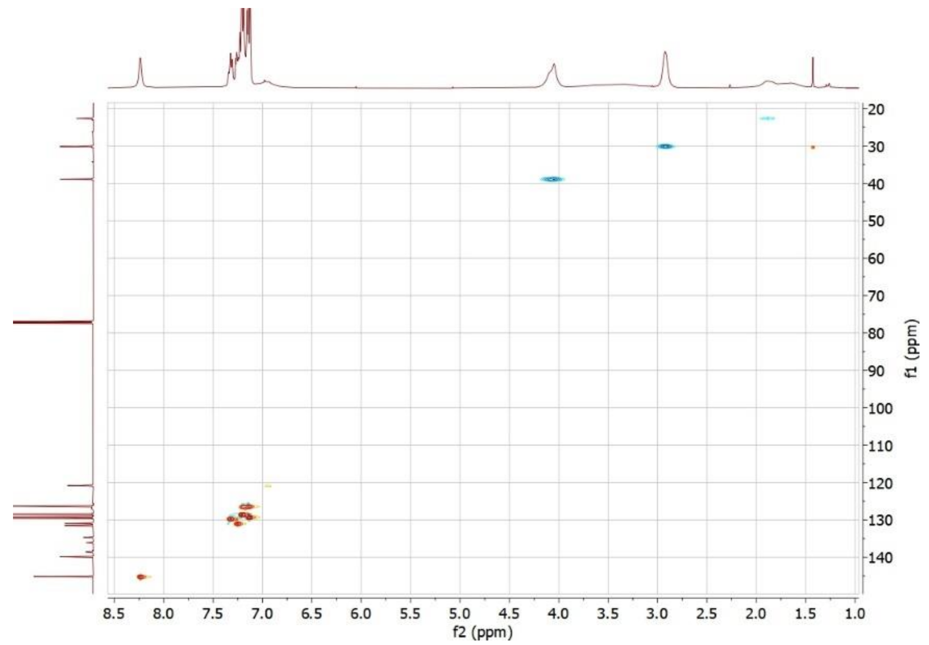

^{1}H NMR of **7c**

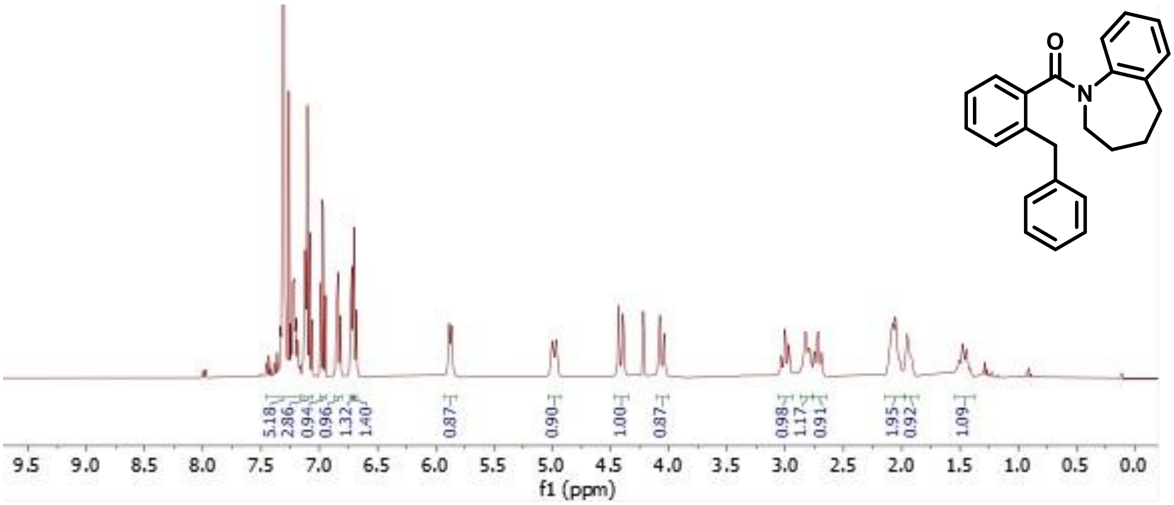

^{13}C NMR of **7c**

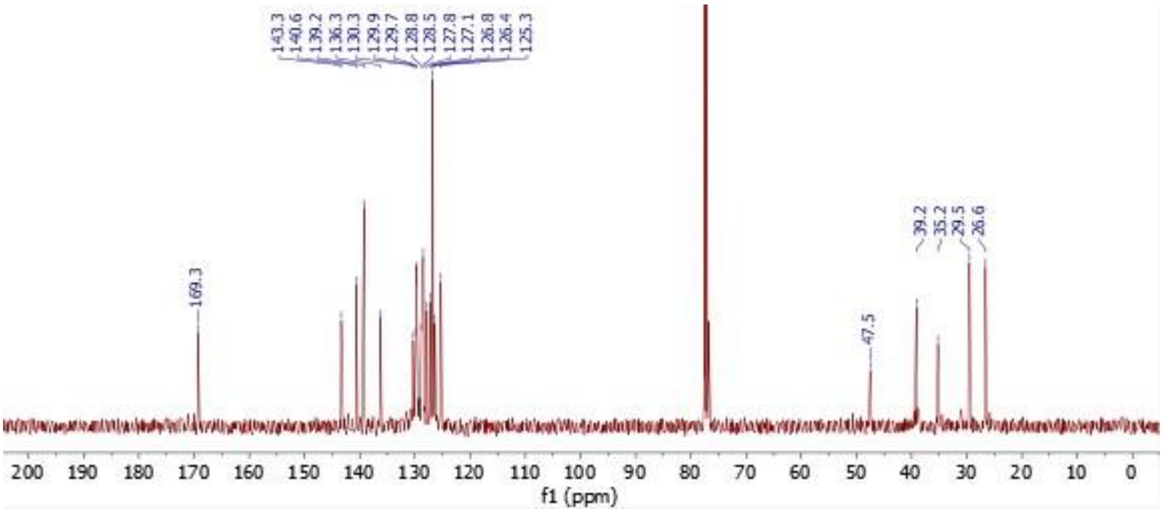

HSQC of **7c**

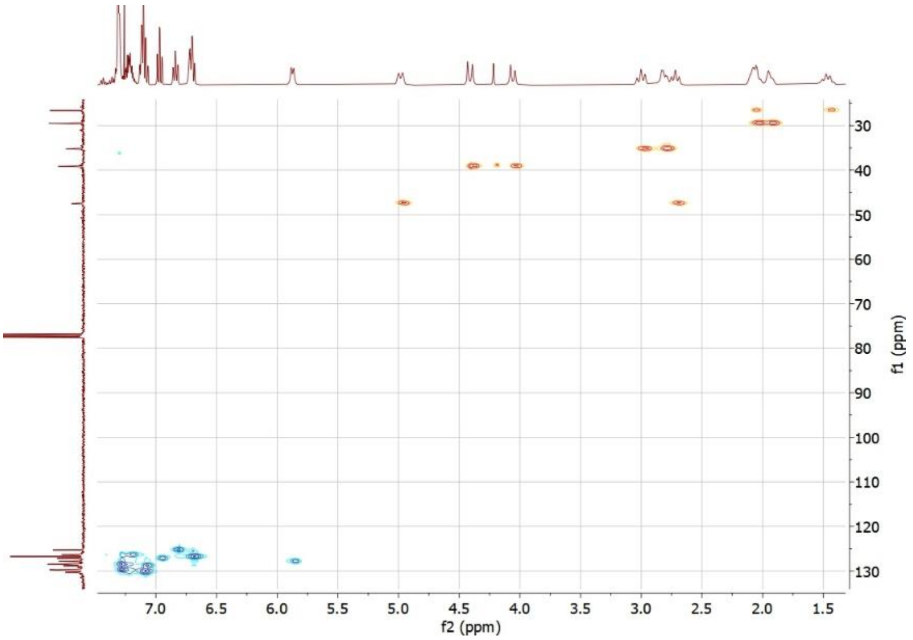

^{1}H NMR of **7d**

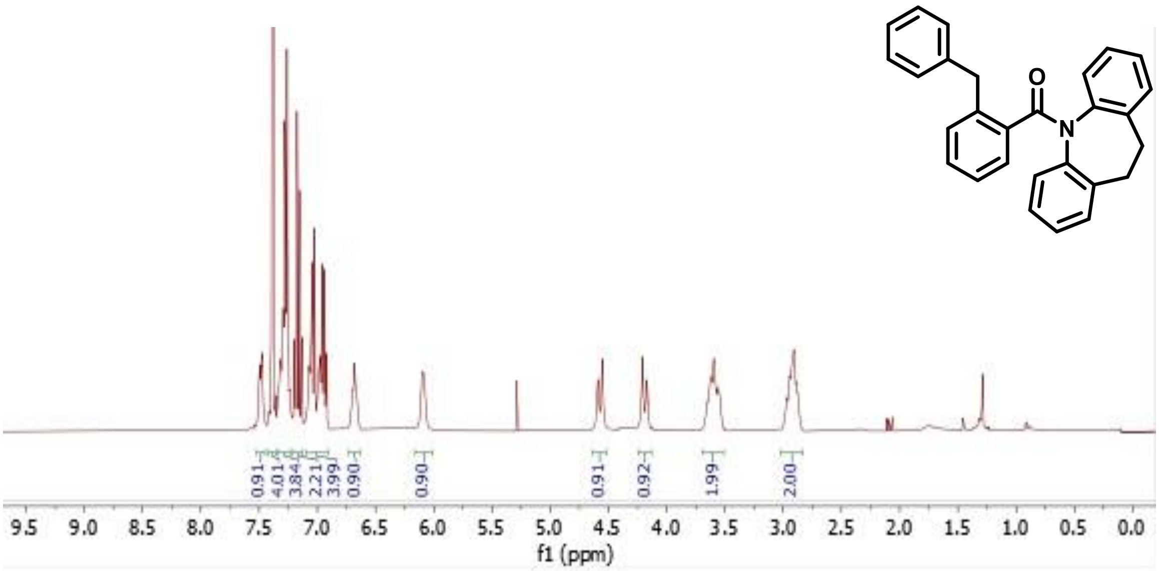

^{13}C NMR of **7d**

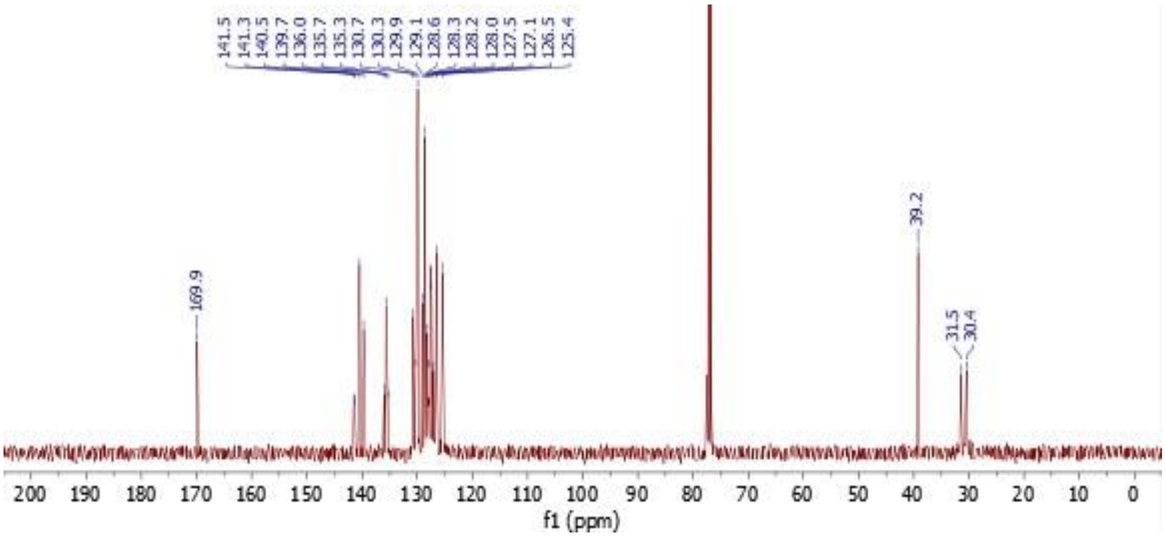

HSQC of **7d**

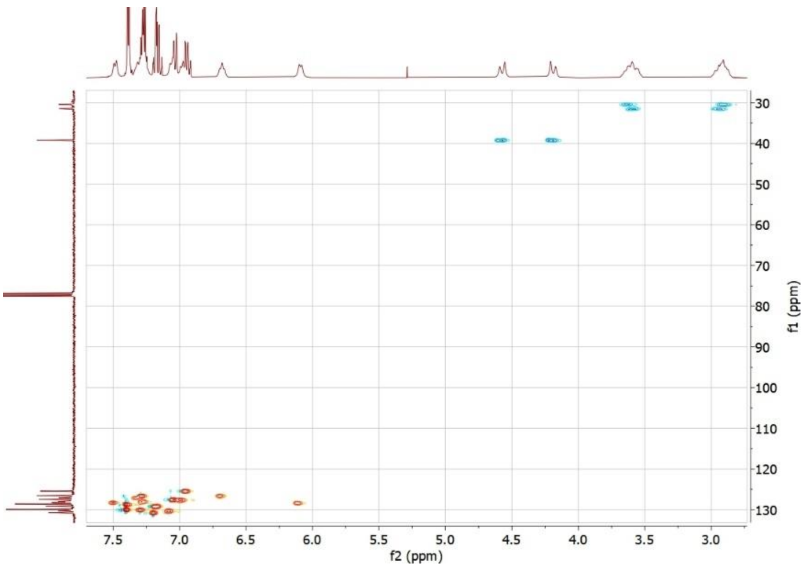

^{1}H NMR of **8a**

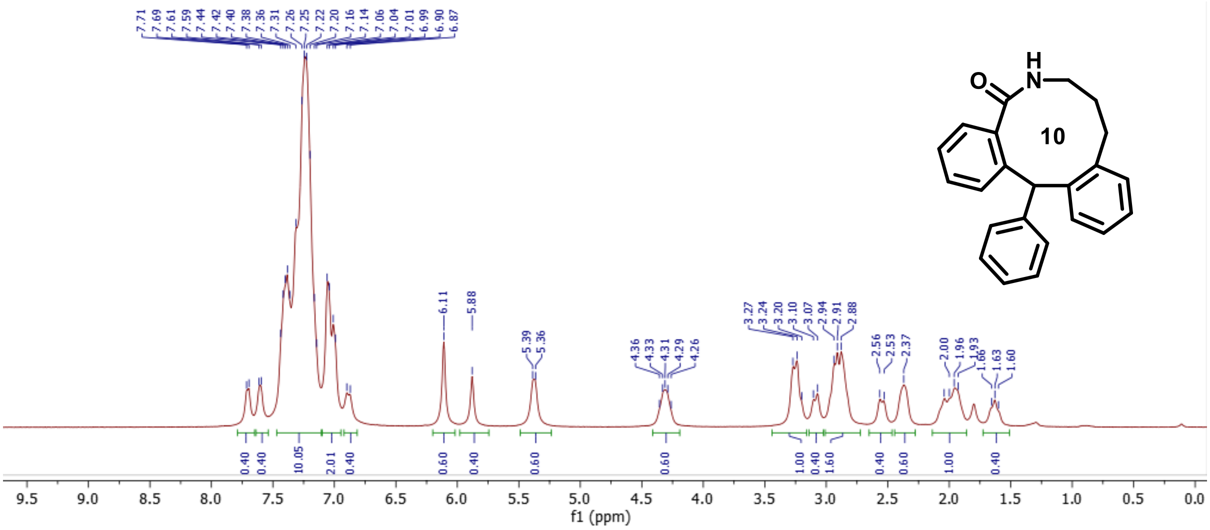

^{13}C NMR of **8a**

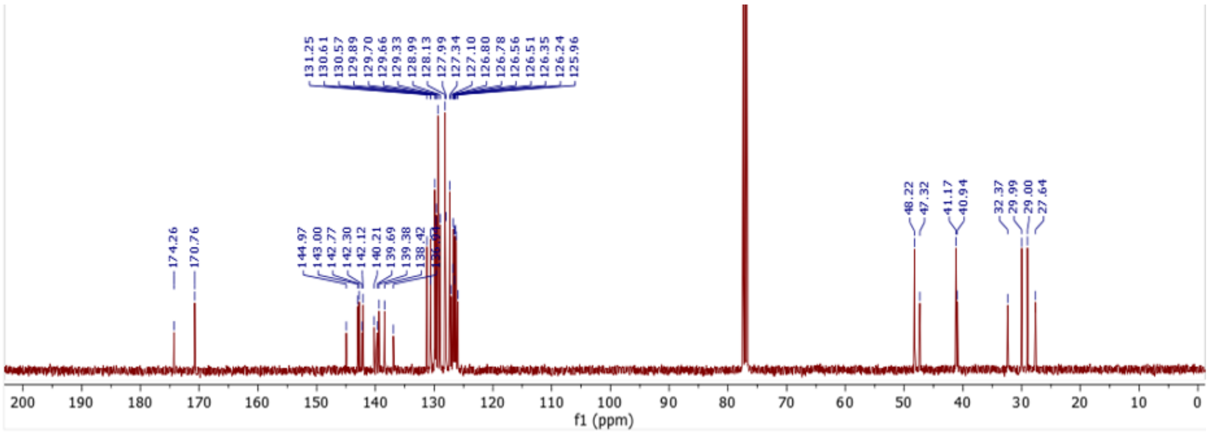

HSQC of **8a**

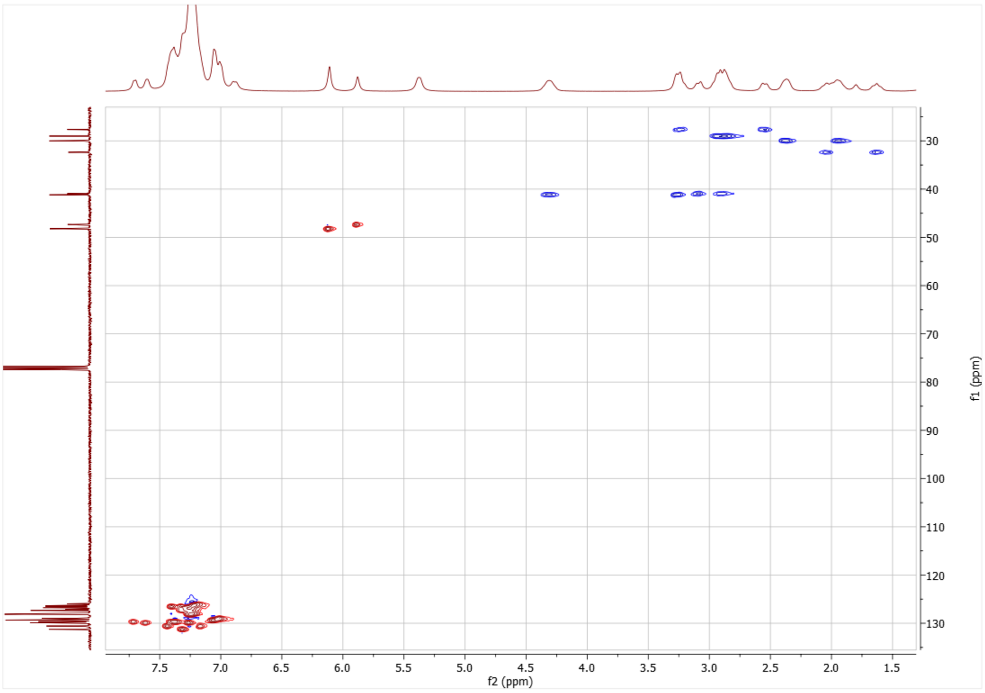

^{1}H NMR of **8b**

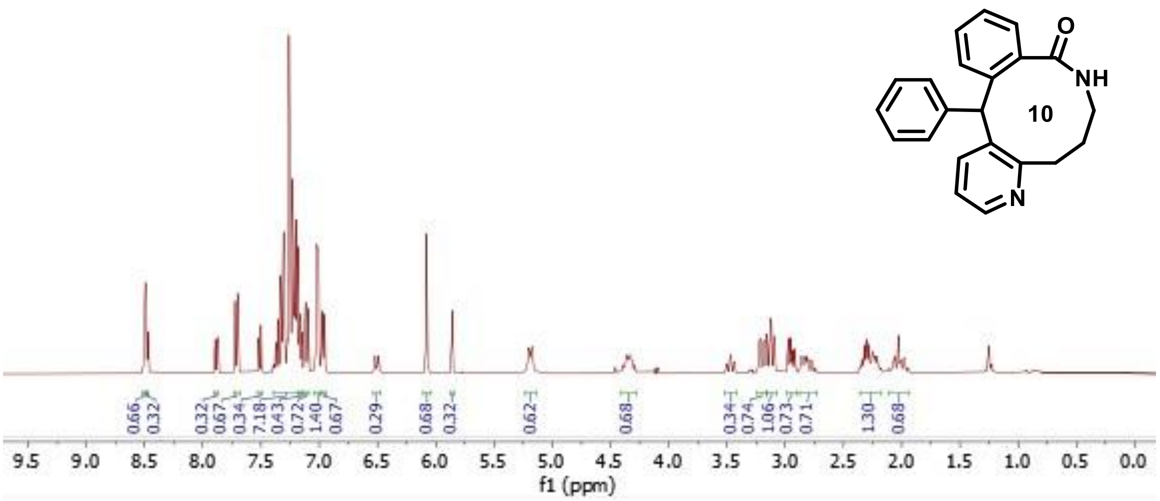

^{13}C NMR of **8b**

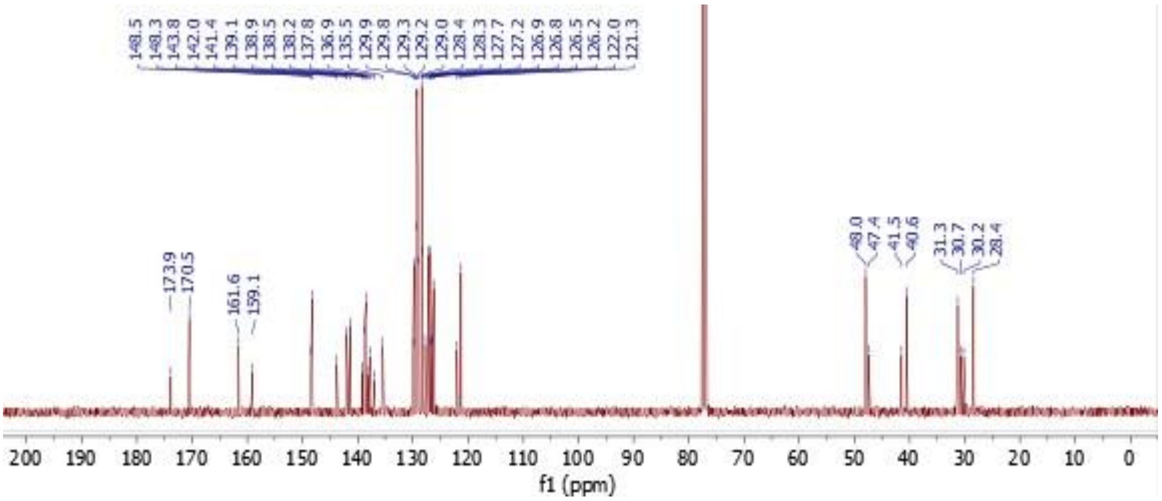

HSQC of **8b**

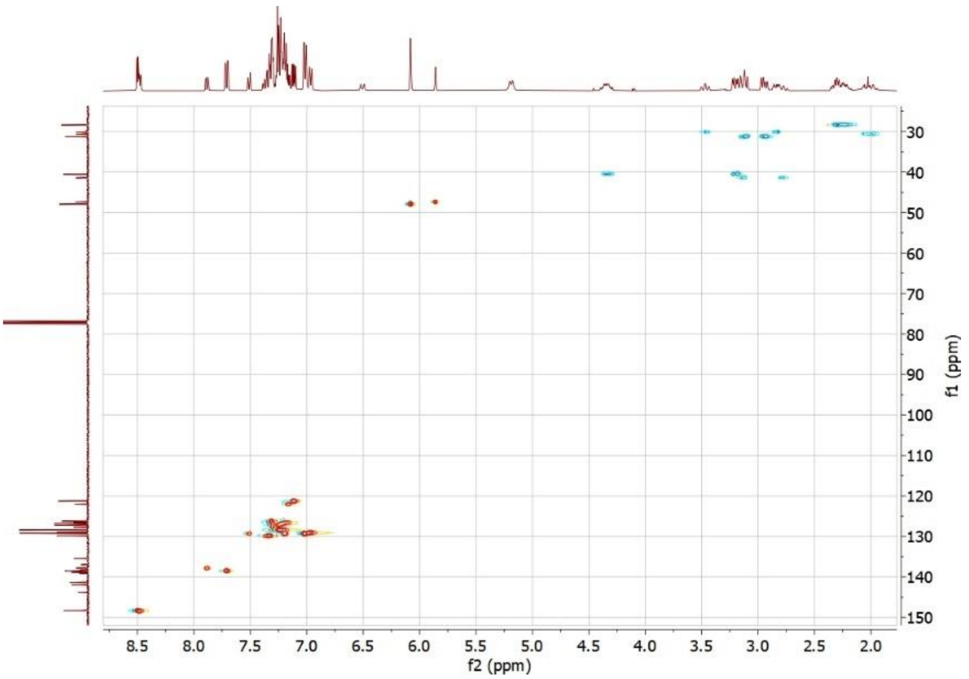

^{1}H NMR of **8c**

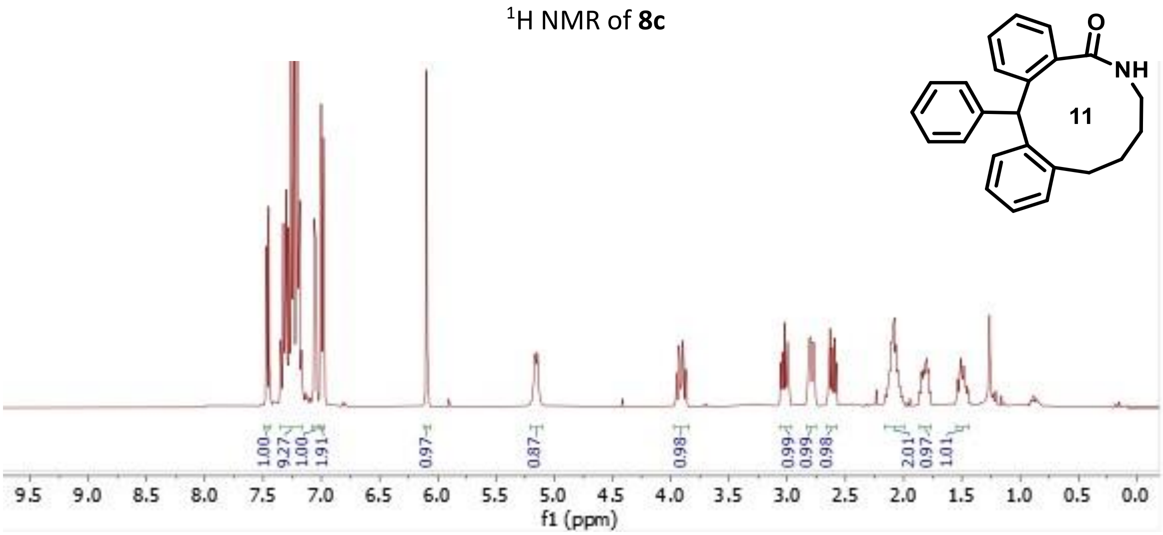

^{13}C NMR of **8c**

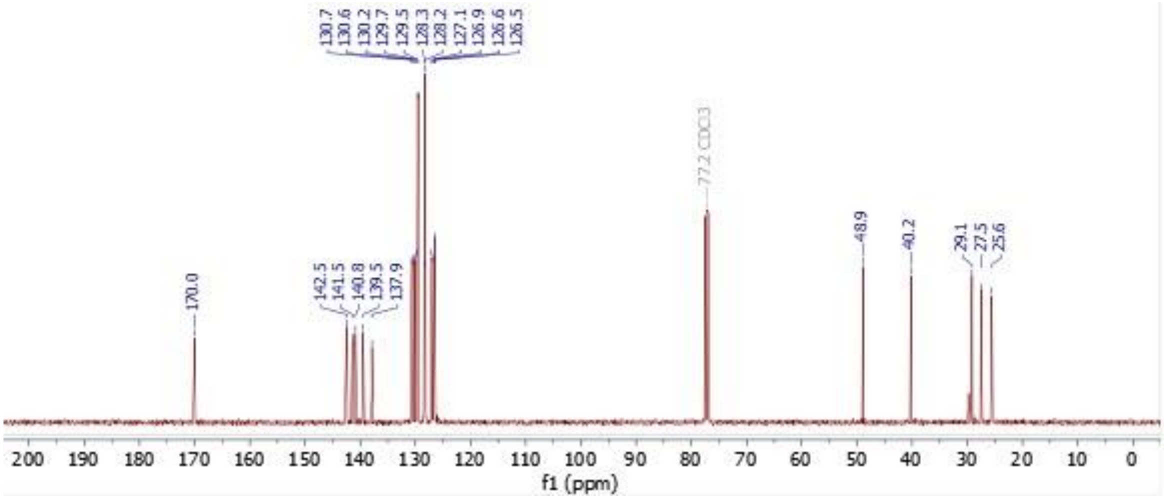

HSQC of **8c**

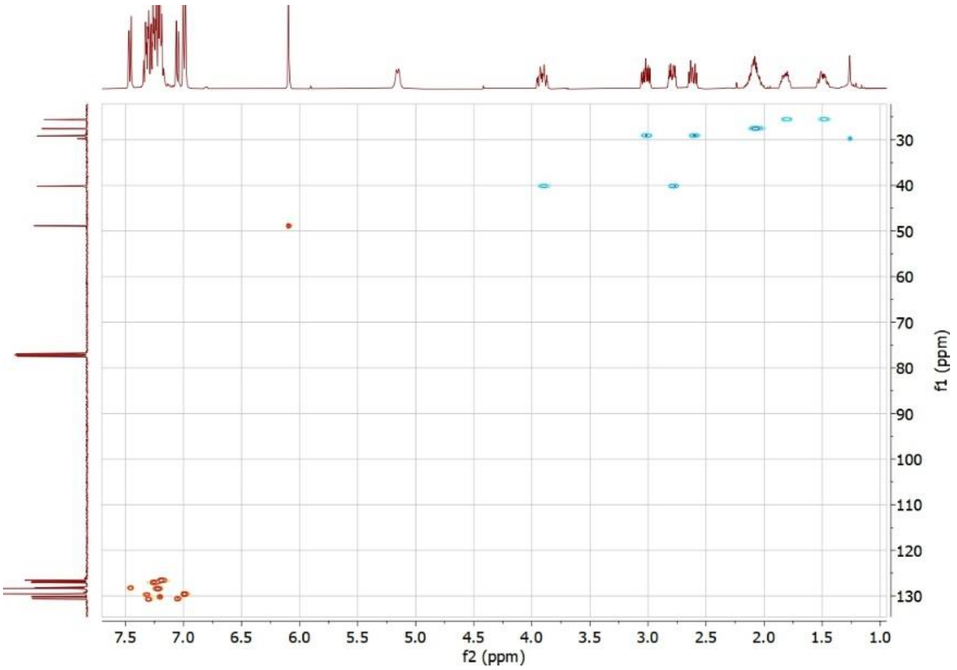

^{1}H NMR of **8d**

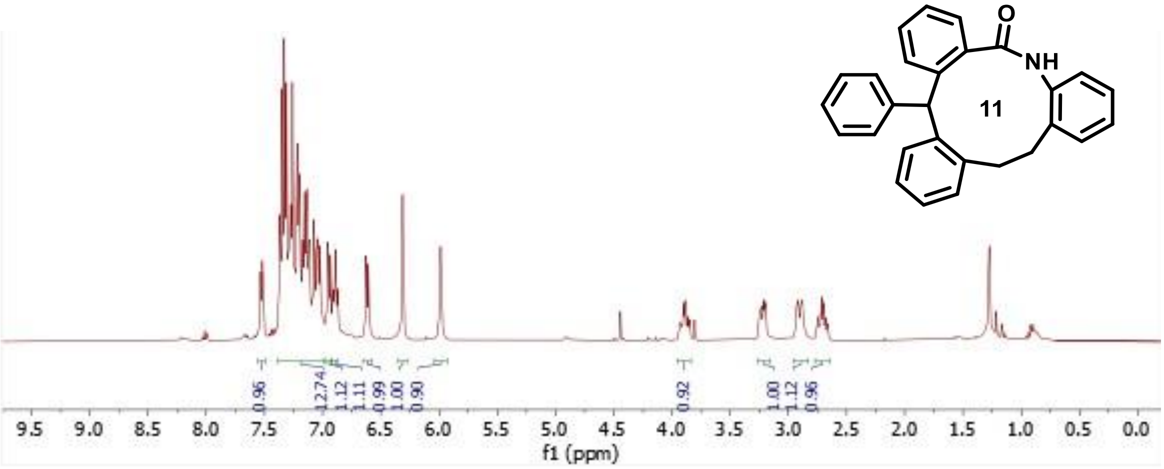

^{13}C NMR of **8d**

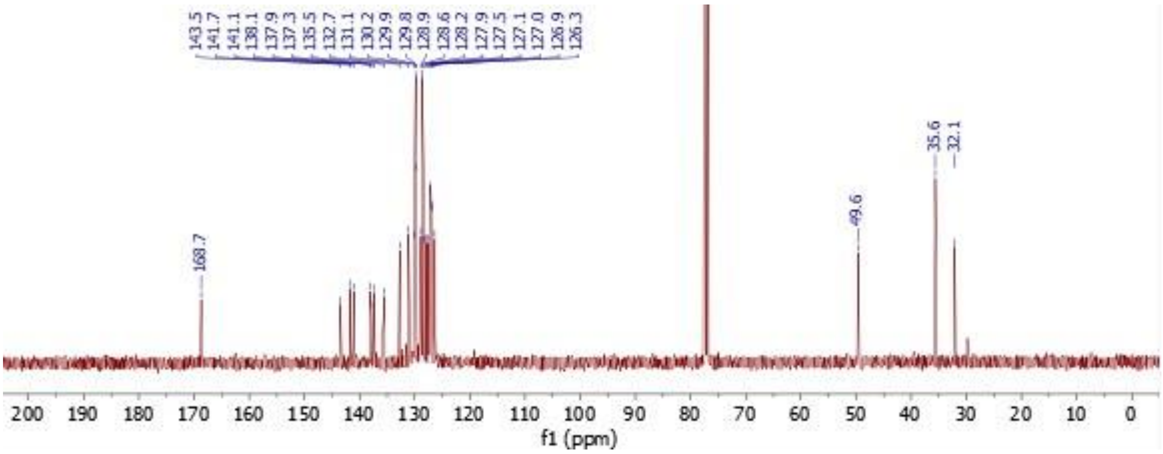

HSQC of **8d**

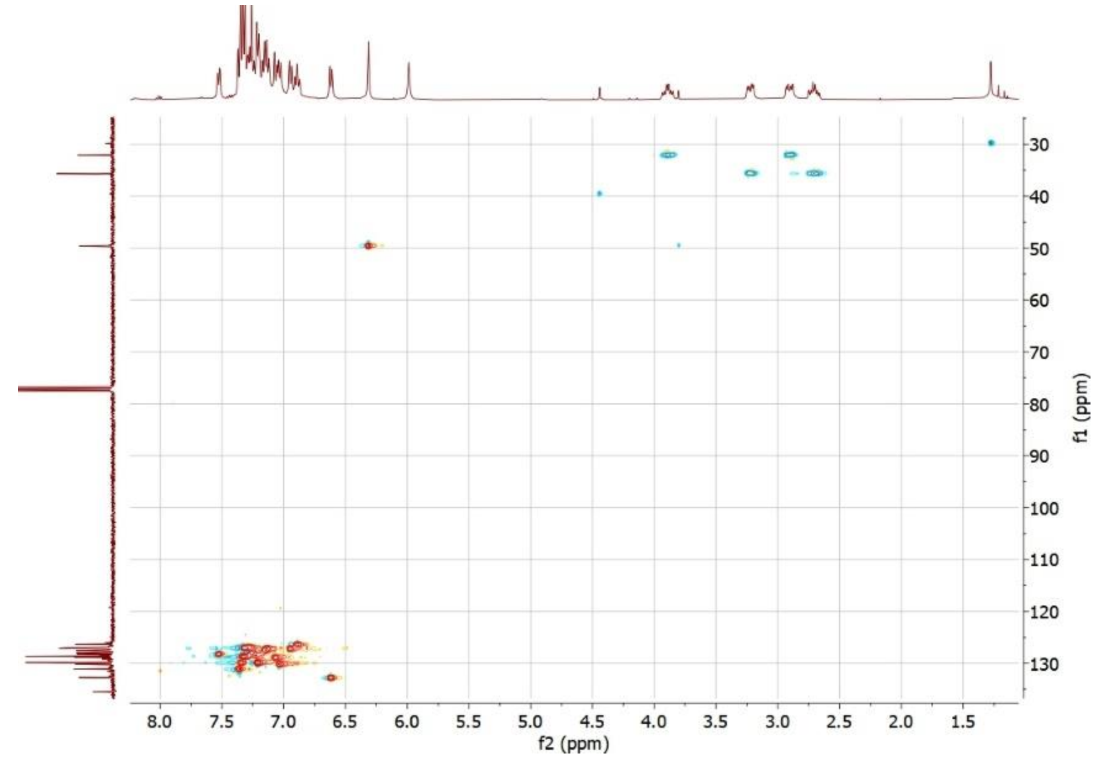

^{1}H NMR of **9**

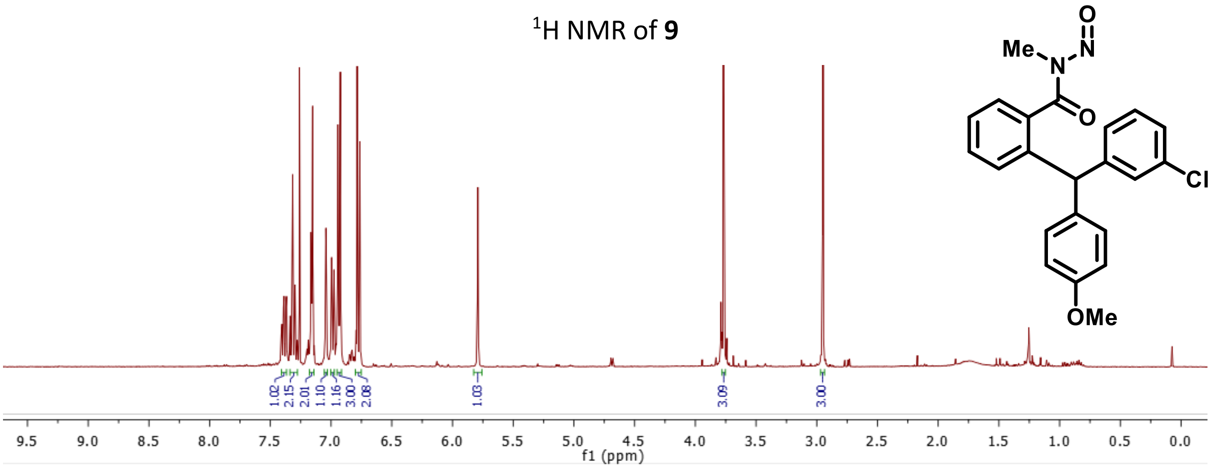

^{13}C NMR of **9**

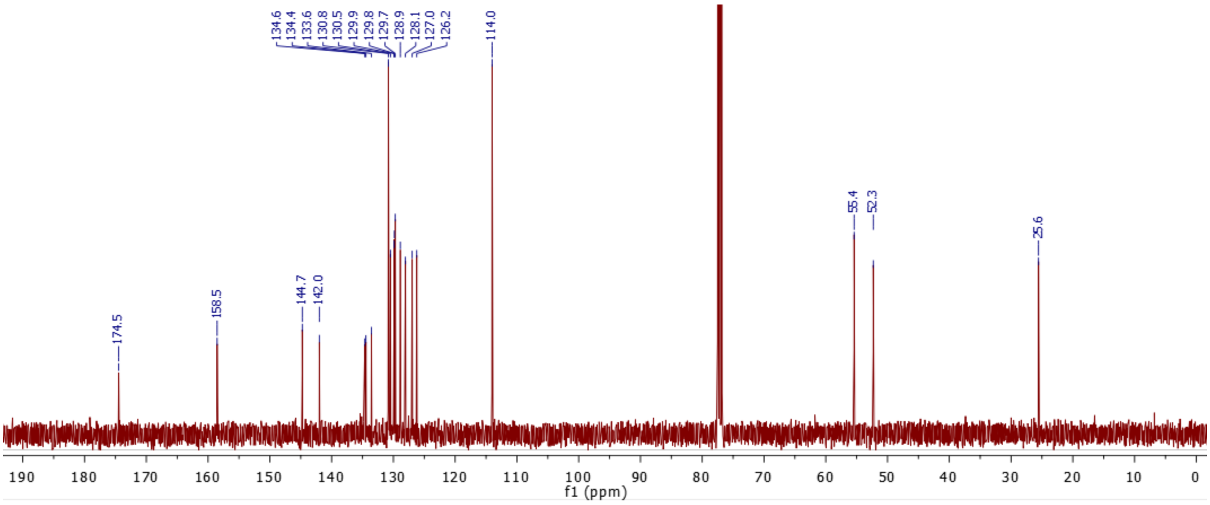

HSQC of **9**

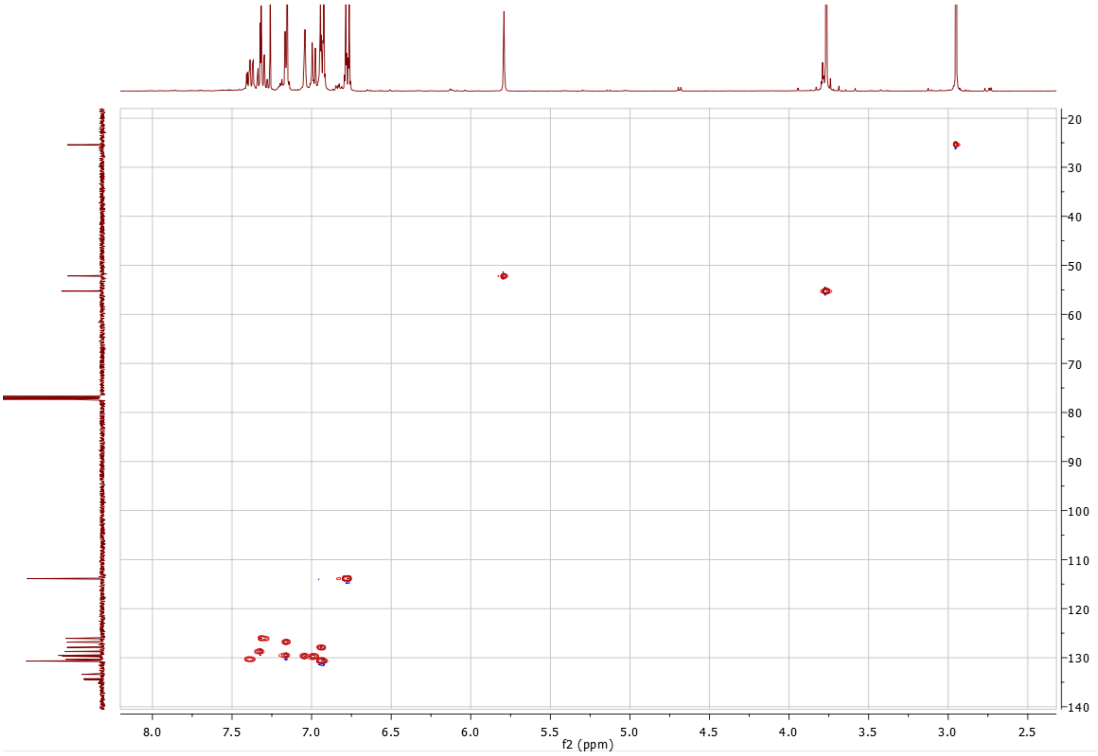

^{1}H NMR of **10**

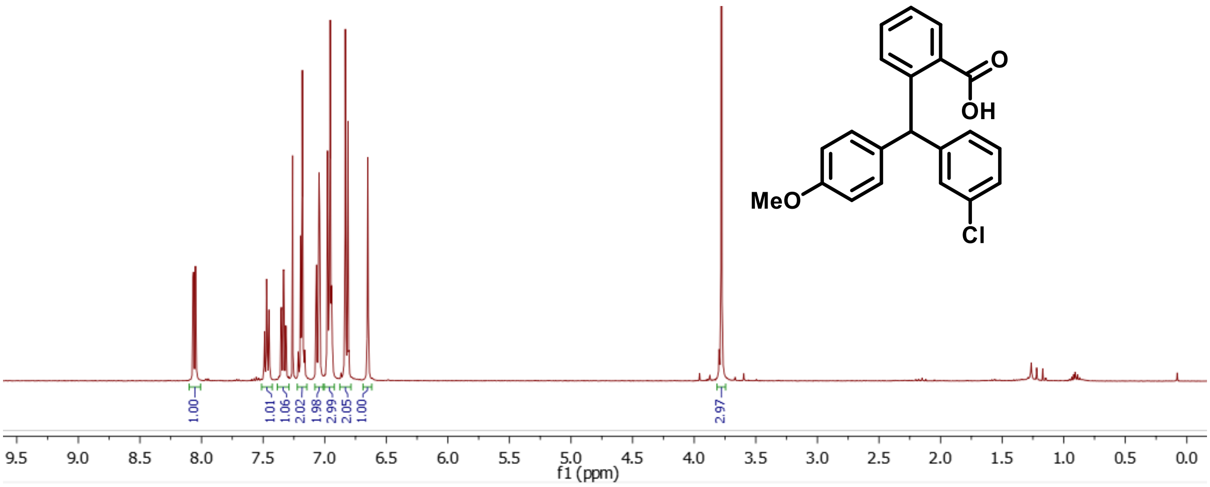

^{13}C NMR of **10**

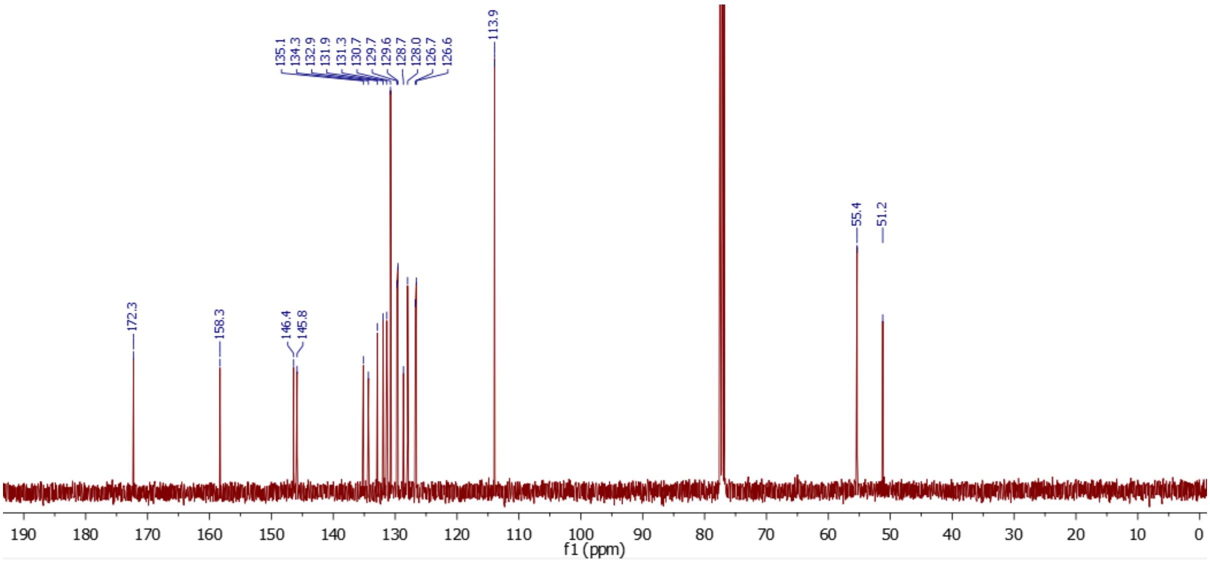

HSQC of **10**

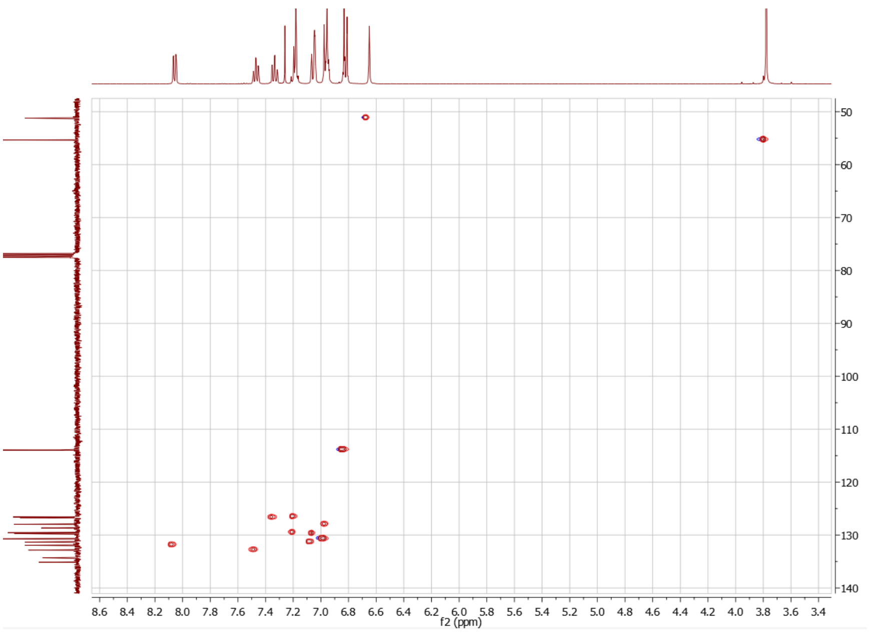

^{1}H NMR of **11**

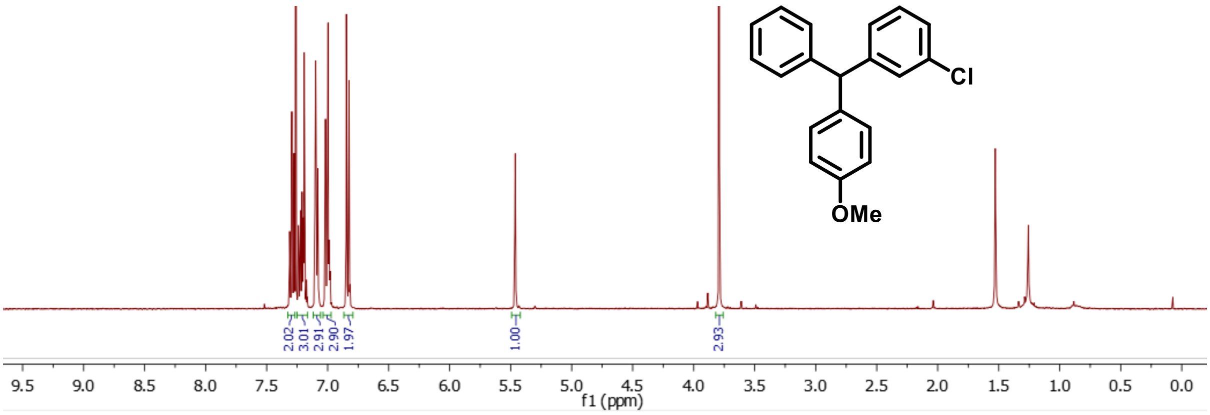

^{13}C NMR of **11**

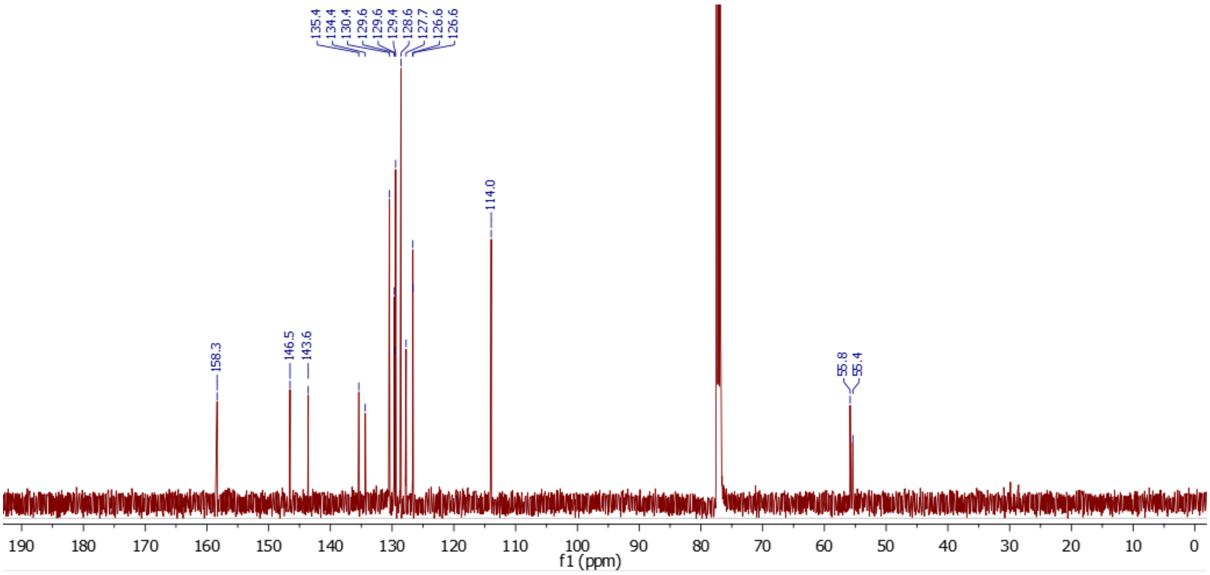

HSQC of **11**

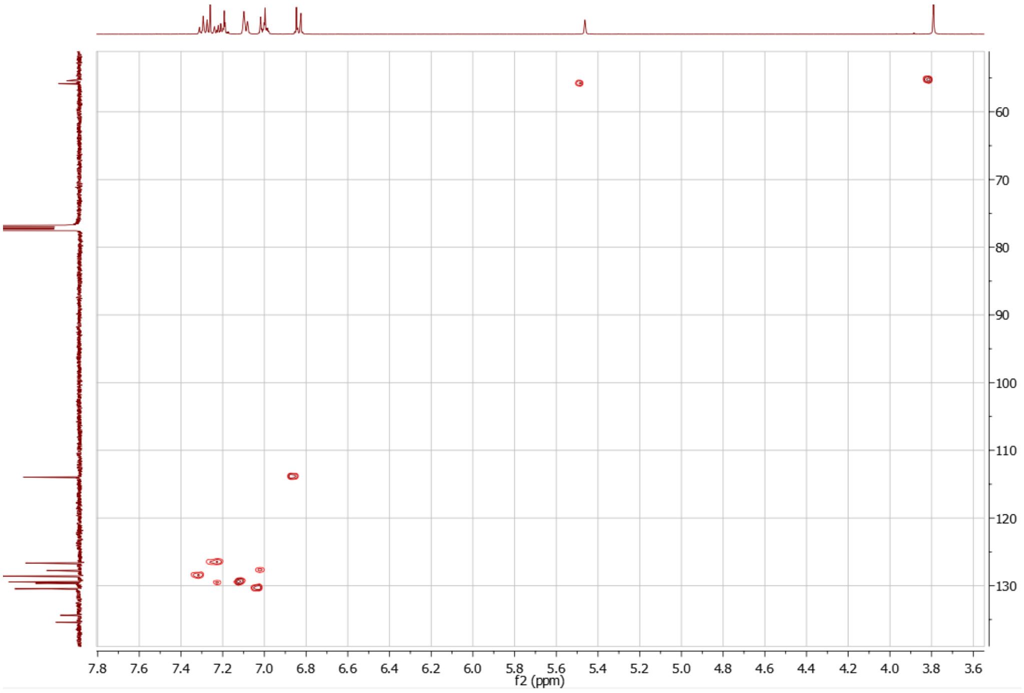

^{1}H NMR of **12**

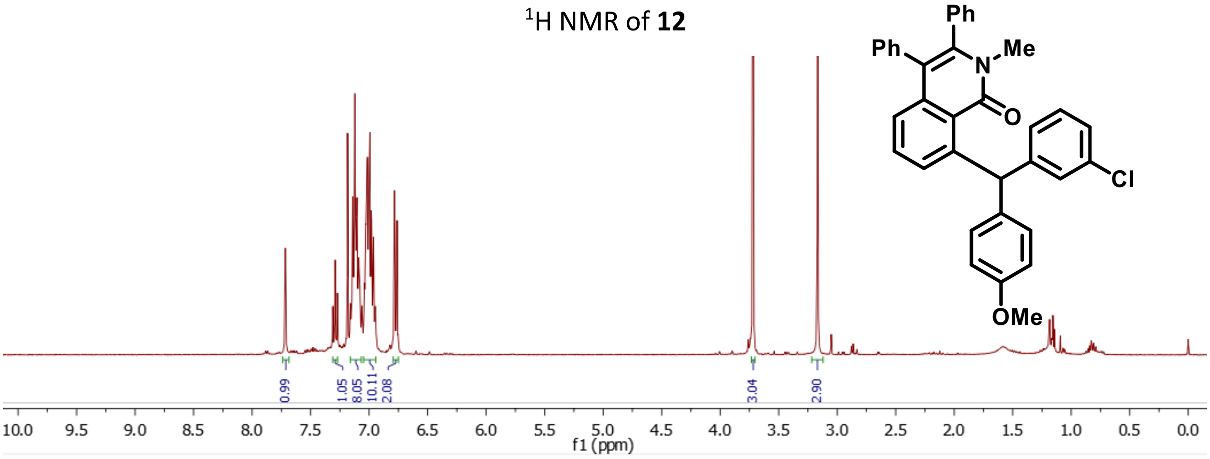

^{13}C NMR of **12**

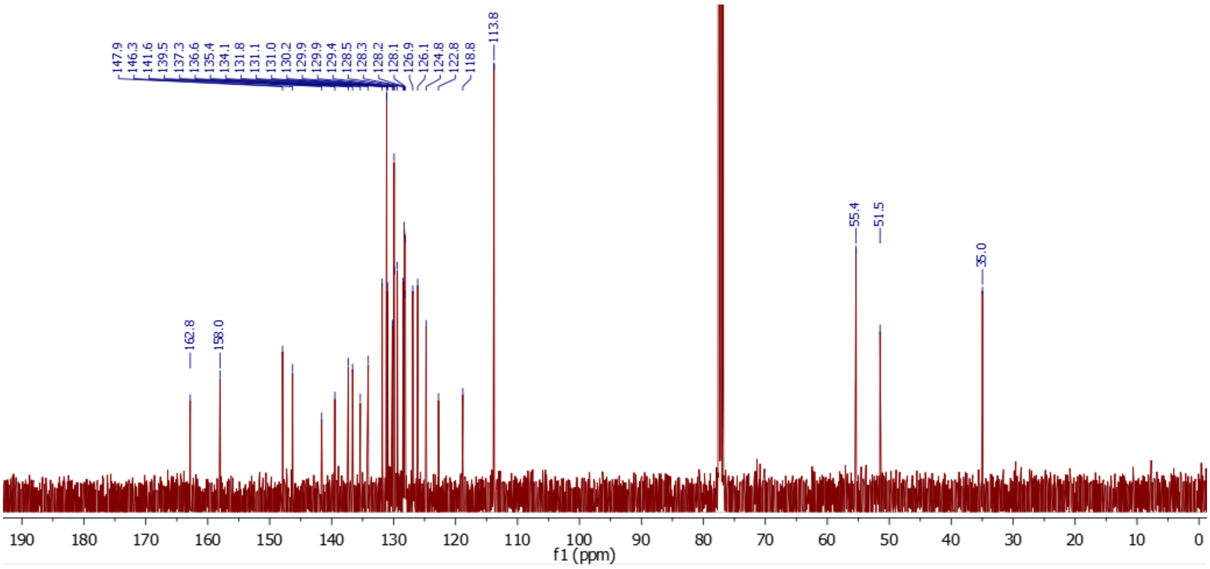

HSQC of **12**

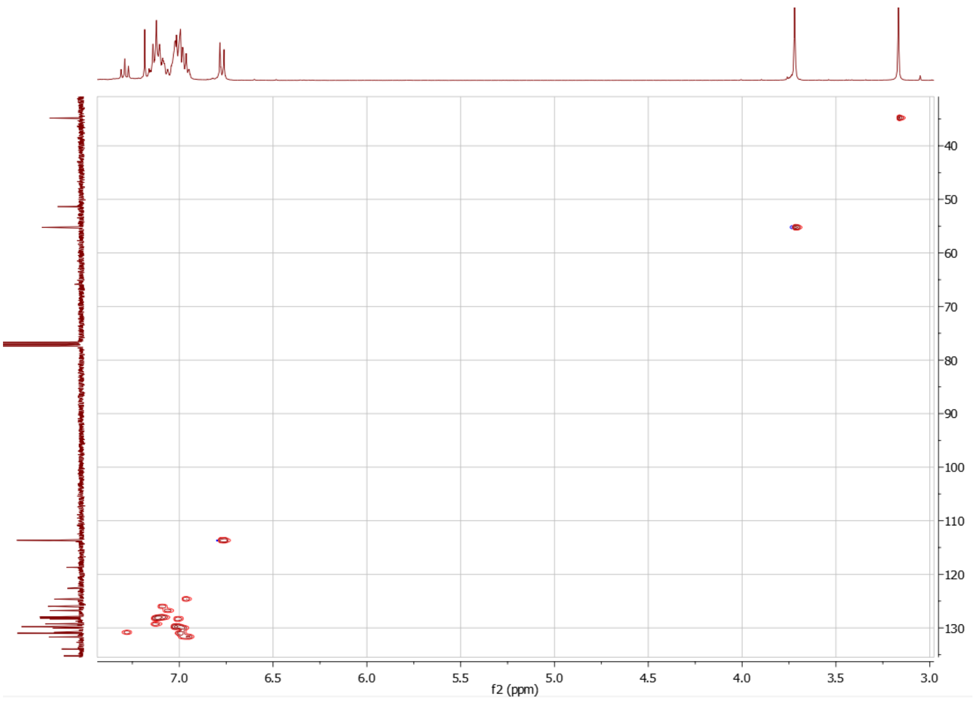

Supplement: Supplementary file 1 — Supplementary [file ANIE-60-11272-s001.pdf]
